# Non-enzymatic catalytic asymmetric cyanation of acylsilanes

**DOI:** 10.1038/s42004-022-00662-y

**Published:** 2022-03-31

**Authors:** Tagui Nagano, Akira Matsumoto, Ryotaro Yoshizaki, Keisuke Asano, Seijiro Matsubara

**Affiliations:** 1 Department of Material Chemistry, Graduate School of Engineering, Kyoto University, Kyotodaigaku-Katsura, Nishikyo, Kyoto, 615-8510 Japan; 2 Present Address: Graduate School of Pharmaceutical Sciences, Kyoto University, Yoshida-Shimoadachi, Sakyo, Kyoto, 606-8501 Japan

**Keywords:** Organocatalysis, Asymmetric catalysis, Synthetic chemistry methodology

## Abstract

The asymmetric cyanation of acylsilanes affords densely functionalized tetrasubstituted chiral carbon centers bearing silyl, cyano, and hydroxy groups, which are of particular interest in synthetic and medicinal chemistry. However, this method has been limited to a few enzymatic approaches, which employ only one substrate because of substrate specificity. Here we show the non-enzymatic catalytic asymmetric cyanation of acylsilanes using a chiral Lewis base as an enantioselective catalyst, trimethylsilyl cyanide as a cyanating reagent, and isopropyl alcohol as an additive to drive catalyst turnover. High enantio- and site-selectivities are achieved in a catalytic manner, and a variety of functional groups are installed in optically active acylsilane cyanohydrins, thus overcoming the limitations imposed by substrate specificity in conventional enzymatic methods. A handle for the synthetic application of the products is also established through the development of a catalyst for protecting acylsilane cyanohydrins, which are unstable and difficult to protect alcohols.

## Abstract (web summary)

Catalytic asymmetric cyanation of ketones is a straightforward method to construct tetrasubstituted chiral carbon centers, but for acylsilanes this method has been limited to enzymatic approaches. Here, non-enzymatic catalytic asymmetric cyanation of acylsilanes is accomplished using a chiral Lewis base as an enantioselective catalyst.

## Introduction

**Fig. 1:**
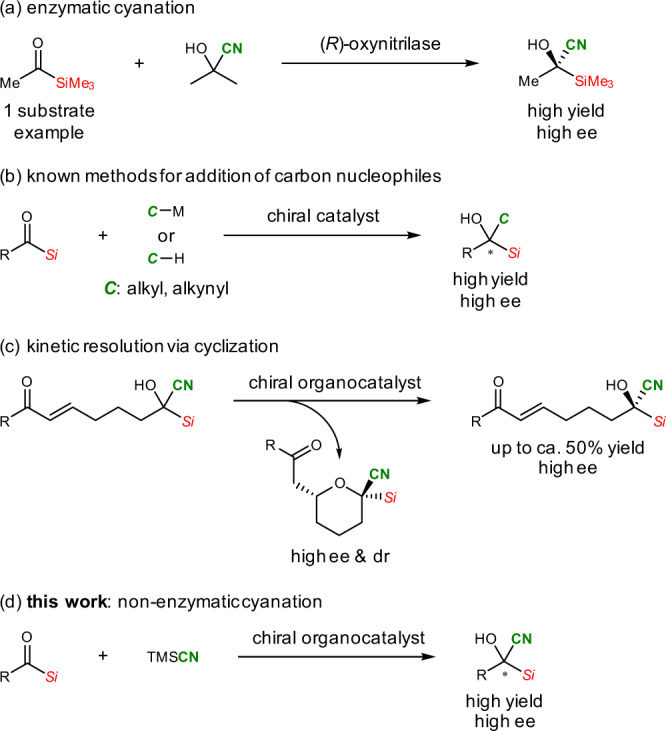
Catalytic asymmetric nucleophilic addition to acylsilanes. **a** Enzymatic cyanation^33,34^. **b** Known methods for the addition of carbon nucleophiles^46–51^. **c** Kinetic resolution via cyclization^59^. **d** Non-enzymatic cyanation.

The catalytic asymmetric cyanation of ketones constitutes a straightforward method for the construction of tetrasubstituted chiral carbon centers^1–15^, which are of particular interest in synthetic^16–23^ and medicinal^24–26^ chemistry. Indeed, owing to the utility of optically active tertiary alcohols bearing cyano groups^27–32^, significant advances have been made in their asymmetric synthesis^1–18^. The asymmetric cyanation of acylsilanes affords densely functionalized tetrasubstituted chiral carbon centers bearing silyl, cyano, and hydroxy groups. However, this method has been limited to a few enzymatic approaches, which employ only one substrate because of substrate specificity (Fig. 1a)^33,34^.

Owing to the increasing interest in chiral silicon-containing molecules, such as silicon isosteres in drug design and development^35–41^ and synthetic building blocks in stereocontrolled C–C bond formation and rearrangements^42–45^, there have been some recent reports on the catalytic asymmetric addition of carbon and heteroatom nucleophiles to acylsilanes^46–52^. However, among carbon nucleophiles, only alkylation and alkynylation have been reported to date (Fig. 1b)^46–51^. It is plausible that cyanation is hindered by the competing Brook rearrangement, which takes place rapidly in basic media^44,53–58^. As an alternative synthetic approach to optically active acylsilane cyanohydrins, we recently reported the kinetic resolution of chiral cyanohydrins generated in situ from acylsilanes involving organocatalytic asymmetric cyclization under nearly neutral conditions which prevent the occurrence of the Brook rearrangement (Fig. 1c)^59^. This reaction is to the best of our knowledge the first non-enzymatic catalytic asymmetric approach to the synthesis of optically active acylsilane cyanohydrins. However, the following problems remain: (1) the maximum yield is ca. 50% due to the principle of kinetic resolution; (2) the substrate structures are limited as they are required to undergo a 6-membered ring formation via intramolecular oxy-Michael addition. To solve these issues, it is necessary to develop a non-enzymatic approach to the asymmetric cyanation of acylsilanes via enantioselective 1,2-addition reactions. Organocatalysis is an effective method not only for achieving high enantioselectivity but also for preventing side reactions. Therefore, we designed a chiral amine-catalyzed cyanation using a silyl cyanide (Fig. 1d)^8,11^. In addition, it is important to establish protocols for transforming the resulting cyanohydrin products, because they remain susceptible to the Brook rearrangement and are difficult to protect because of the adjacent bulky silyl group. In this study, we developed a novel Lewis-base-catalyzed enantioselective cyanation of acylsilanes, which is to the best of our knowledge the first non-enzymatic catalytic method that leads to quantitative yields of optically active acylsilane cyanohydrins. This method does not necessitate any specific substrate structure, and various functional groups are tolerated. Furthermore, the newly developed catalytic method for the silylation of the product alcohols constitutes a valuable handle for their synthetic applications.

## Results

### Optimization of reaction conditions

**Fig. 2:**
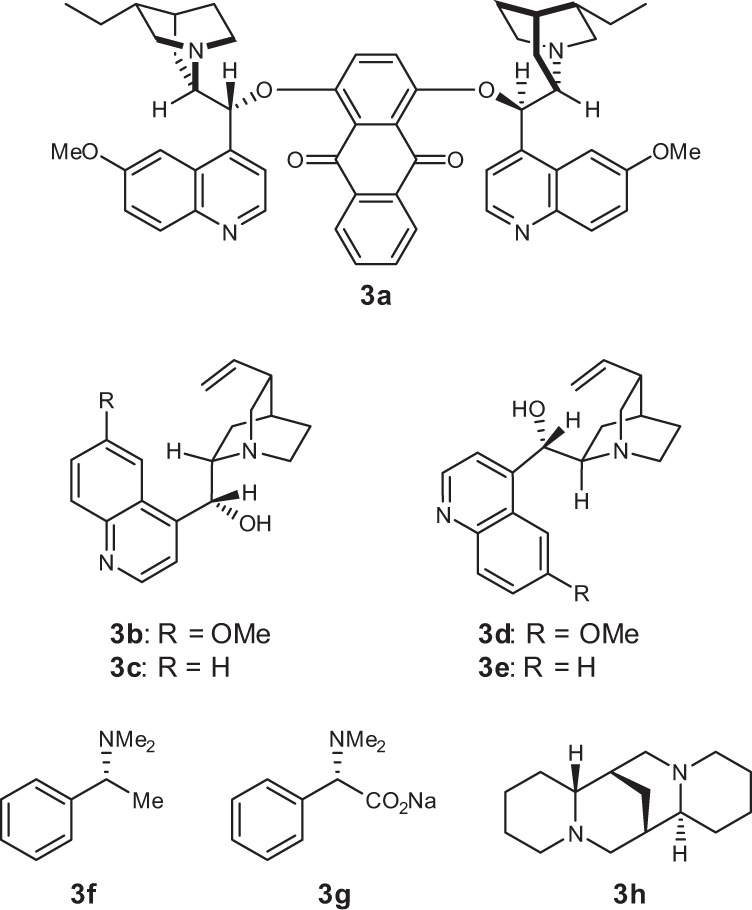
Organocatalysts. **3a** (DHQ)_2_AQN. **3b** Quinine. **3c** Cinchonidine. **3d** Quinidine. **3e** Cinchonine. **3f** (*R*)-*N*,*N*-Dimethyl-1-phenylethan-1-amine. **3g** Sodium (*S*)-2-(dimethylamino)-2-phenylacetate. **3h** (–)-Sparteine.

We initiated our investigations using 4-phenyl-1-(trimethylsilyl)butan-1-one (**1a**) and 2.0 equivalents of trimethylsilyl cyanide (TMSCN) with 20 mol % of the chiral amine catalysts **3a**–**3h** (Fig. 2) in CHCl_3_ at –78 °C (Table 1, entries 1–8; see also Supplementary Table 1). Catalyst **3a** yielded the corresponding product **2a** with high enantioselectivity (Table 1, entry 1), while the cinchona alkaloids **3b**–**3e** and other amine catalysts **3f** and **3g** were less active and exhibited lower enantioselectivity (Table 1, entries 2–7). The use of catalyst **3h** resulted in a high yield but low enantioselectivity. In addition, while the catalysts **3a**–**3g** afforded the alcohol product **2a**, only catalyst **3h** yielded the trimethylsilyl ether of **2a**. Various solvents were also investigated using **3a** as the catalyst (Table 1, entries 9–14). CHCl_3_ proved to be the most efficient solvent (Table 1, entry 1). At a lower catalyst loading of 10 mol %, a significantly low yield was obtained, while a high enantioselectivity was maintained (Table 1, entry 15). Lengthening the reaction time did not improve the yield (Table 1, entry 16), which implied that the catalyst was deactivated during the reaction.

**Table 1:** Optimization of reaction conditions^a^.


Entry	Catalyst (mol%)	Solvent	Yield (%)^b^	ee (%)
1	**3a** (20)	CHCl_3_	88	94
2	**3b** (20)	CHCl_3_	42	83
3	**3c** (20)	CHCl_3_	22	49
4	**3d** (20)	CHCl_3_	31	–61
5	**3e** (20)	CHCl_3_	23	19
6	**3f** (20)	CHCl_3_	24	41
7	**3g** (20)	CHCl_3_	<5	—
8^c^	**3h** (20)	CHCl_3_	93	21
9	**3a** (20)	CH_2_Cl_2_	84	94
10	**3a** (20)	EtOAc	61	95
11	**3a** (20)	acetone	57	95
12	**3a** (20)	THF	39	89
13	**3a** (20)	toluene	48	93
14	**3a** (20)	hexane	15	60
15	**3a** (10)	CHCl_3_	37	95
16^d^	**3a** (10)	CHCl_3_	36	96

^a^Reactions were run using **1a** (0.20 mmol), TMSCN (0.40 mmol), and the catalyst in the solvent (0.20 mL).

^b^Isolated yields.

^c^The trimethylsilyl ether of **2a** was obtained as the product.

^d^Reaction was run for 48 h.

As the product **2a** was obtained as the alcohol, it was postulated that the silyl group derived from TMSCN remained attached to the Lewis basic moiety of **3a** after the cyanation of **1a**, which disturbed the turnover of **3a**. Therefore, to improve the catalytic efficiency, the use of an additive was investigated (Table 2; see also Supplementary Table 2). Alcohols were used as additives for scavenging the silyl group and protonating the alkoxide resulting from the cyanation (in the absence of any additive, the dissolved water was probably involved in the catalytic cycle to yield the product, albeit in low yields). In the presence of methanol (MeOH), although the yield significantly increased as expected, the enantioselectivity decreased (Table 2, entry 2). A bulkier alcohol, isopropyl alcohol (*i*-PrOH), provided a higher enantioselectivity than MeOH, while the yield was similarly high (Table 2, entry 3). However, an even bulkier alcohol, tertiary butyl alcohol, exhibited negligible effects as an additive (Table 2, entry 4). Further investigations revealed that the use of 1.0 equivalent of *i*-PrOH with 5.0 mol % of **3a** provided a high yield with appreciable enantioselectivity (Table 2, entry 5). A 1.0 mmol scale reaction also resulted in comparable results (see Supplementary Scheme 1 for details). Higher temperatures resulted in lower enantioselectivities (see Supplementary Table 2 for details).

**Table 2:** Effects of alcohol additives^a^.


Entry	Additive (equiv)	Yield (%)^b^	ee (%)
1	None	37	95
2	MeOH (2.0)	90	82
3	*i*-PrOH (2.0)	91	87
4	*t*-BuOH (2.0)	33	95
5^*c*^	*i*-PrOH (1.0)	88	91

^a^Reactions were run using **1a** (0.20 mmol), TMSCN (0.40 mmol), the additive, and **3a** (0.020 mmol) in CHCl_3_ (0.20 mL).

^b^Isolated yields.

^c^Reaction was run using 5.0 mol % (0.010 mmol) of **3a**.

### Mechanistic investigations

**Fig. 3:**
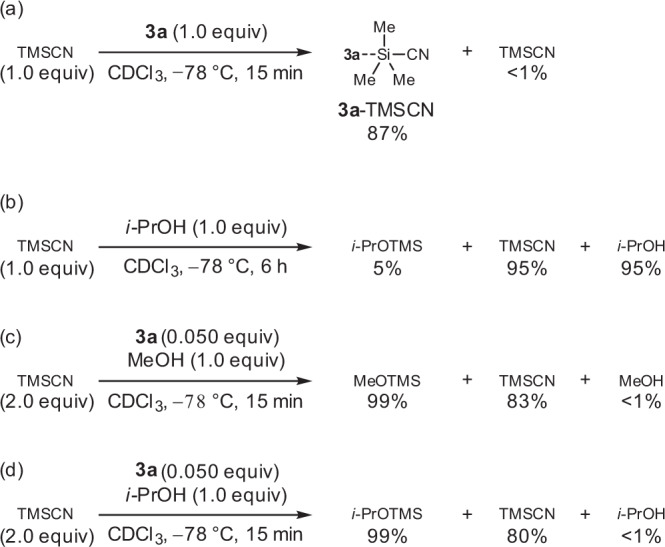
Results of NMR studies. **a** TMSCN:**3a** = 1:1. **b** TMSCN:*i*-PrOH = 1:1. **c** TMSCN:MeOH:**3a** = 2:1:0.05; the generation of TMSOTMS was also observed in 12% yield. **d** TMSCN:*i*-PrOH:**3a** = 2:1:0.05; the generation of TMSOTMS was also observed in 18% yield. The reaction mixtures were stirred in CDCl_3_ at –78 °C, and NMR analyses were carried out at –60 °C to prevent the solutions from freezing in the NMR sample tubes. Yields are values calculated with 1.0 equivalent of starting material, identified as 100%, and the theoretical maximum yield of TMSCN recovered under the conditions described in equations **c** and **d** is 200%.

The alcohol additives play two possible roles. First, *i*-PrOH scavenges the silyl group remaining on the Lewis basic moiety of **3a** after the cyanation of **1a**, which involves the formation of a **3a**-TMSCN complex as the cyanating species. Second, *i*-PrOH reacts with TMSCN to supply hydrogen cyanide (HCN), which is subsequently activated by **3a** for the cyanation of **1a**. The formation of these species was verified by performing nuclear magnetic resonance (NMR) analyses of the reaction mixtures (Fig. 3). NMR analysis of the solution of **3a** and TMSCN in CDCl_3_ (reaction time: 15 min) indicated that the signal associated with the protons of the TMS group was shifted upfield, suggesting the coordination of **3a** to the silyl group of TMSCN (Fig. 3a; see also Supplementary Scheme 2). The solution of *i*-PrOH and TMSCN in CDCl_3_ (reaction time: 6 h) exhibited only a small signal associated with *i*-PrOTMS, which appeared along with the generation of HCN, suggesting that in the absence of **3a**, insignificant amounts of HCN were generated at low temperatures even after a long time (Fig. 3b; see also Supplementary Scheme 3). On the other hand, the solutions of MeOH and *i*-PrOH with TMSCN and **3a** in CDCl_3_ (reaction time: 15 min) exhibited signals associated with MeOTMS and *i*-PrOTMS, respectively, while those corresponding to the alcohols disappeared, suggesting that in the presence of **3a**, HCN was generated in both the cases after a certain time (Fig. 3c and d; see also Supplementary Scheme 4).

**Fig. 4:**
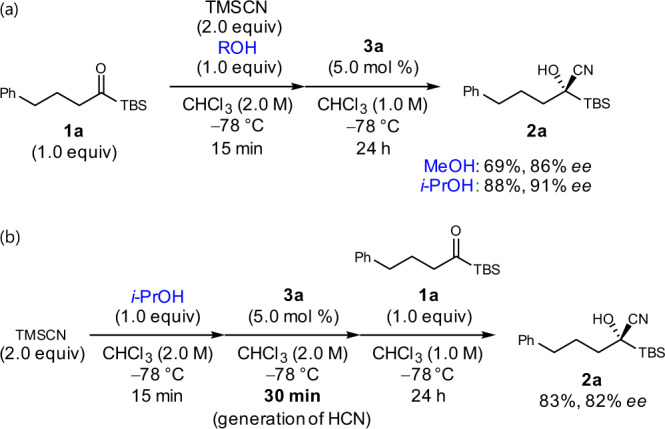
Reactions with different procedures. **a** Optimized procedures. **b** Modified procedures.

Notably, the enantioselectivity was affected by the alcohol additive (Table 2, entries 2 and 3), and we supposed the existence of two competing catalytic pathways, which involved the **3a**-TMSCN complex and the **3a**-HCN complex as the cyanating species, in a parallel manner. We hypothesized that the pathway involving **3a**-TMSCN afforded a higher enantioselectivity than the one involving **3a**-HCN, and *i*-PrOH produced HCN at a slower rate than MeOH. Thus, we tested a modified procedure, in which a mixture of *i*-PrOH, TMSCN, and **3a** was stirred in CHCl_3_ at –78 °C for 30 min, which was sufficient to generate HCN, before **1a** was added (Fig. 4). Under these conditions, the enantioselectivity decreased to 82% enantiomeric excess (*ee*), which was even less than that of the reaction using MeOH with the optimized procedures (86% *ee*). These results support the hypothesis mentioned above.

**Fig. 5:**
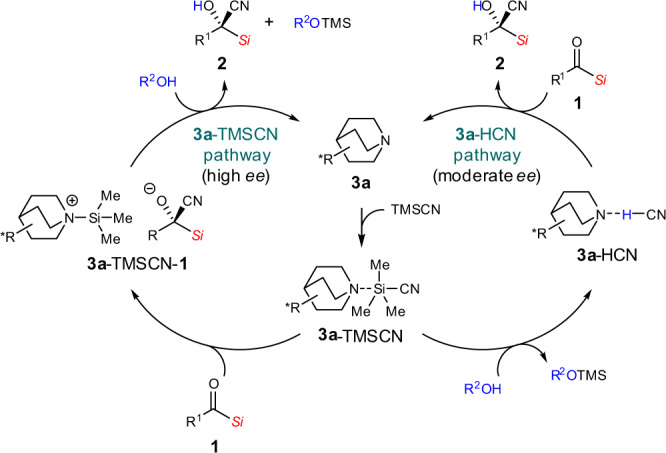
Proposed catalytic pathways. A plausible mechanism consists of **3a**-TMSCN and **3a**-HCN pathways.

The catalytic pathways are proposed based on the experimental results (Fig. 5; see also Supplementary Scheme 5). The **3a**-TMSCN complex is generated as a common intermediate. It may react rapidly with less bulky alcohols (e.g. MeOH) to provide HCN, which is involved in the less enantioselective catalytic cycle (**3a**-HCN pathway). On the other hand, the reaction of *i*-PrOH with the **3a**-TMSCN complex is slow, which is probably due to the bulkiness of *i*-PrOH, and the **3a**-TMSCN complex is involved in the cyanation (**3a**-TMSCN pathway), thereby leading to a higher enantioselectivity. In the **3a**-TMSCN pathway, *i*-PrOH scavenges the TMS group from **3a**-TMSCN-**1** to regenerate **3a** and protonates the resulting alkoxide to provide **2**. Experiments using *i*-PrOH-*d*_8_ are also mentioned in Supplementary Scheme 6.

### Substrate scope and site-selectivity

**Fig. 6:**
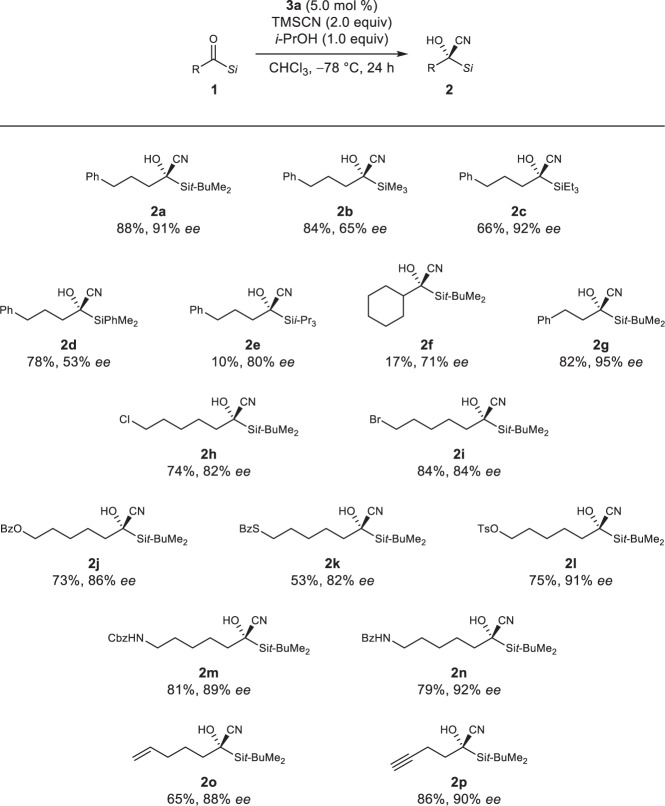
Substrate scope. Reactions were run using **1** (0.20 mmol), TMSCN (0.40 mmol), *i*-PrOH (0.20 mmol), and **3a** (0.010 mmol) in CHCl_3_ (0.20 mL). Yields represent material isolated after silica gel column chromatography.

**Fig. 7:**
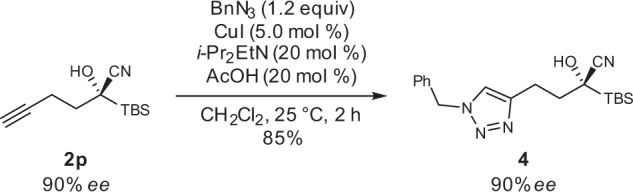
CuAAC of 2p. The alkynyl group of **2p** was amenable to click ligation.

We explored the substrate scope under the optimized conditions using 5.0 mol % of **3a** and 1.0 equivalent of *i*-PrOH (Fig. 6). Various silyl groups were investigated (Figs. 6, **2a**–**e**). Bulky silyl groups were found to provide higher enantioselectivities but lower yields. A bulky alkyl group was also tolerated, providing moderate enantioselectivity, albeit with a low yield (Figs. 6, **2f**). A shorter alkyl group was well tolerated, affording the product in good yield with high enantioselectivity (Figs. 6, **2g**). We also investigated substrates bearing various functional groups on the alkyl group (Figs. 6, **2h**–**p**). Halogenated substrates resulted in good yields and enantioselectivities (Figs. 6, **2h** and **i**). Ester, thioester, and sulfonic ester functionalities, which are useful for further transformations, were also tolerated to afford the corresponding products with high enantioselectivities (Figs. 6, **2j**, **k**, and **l**). In addition, amino and amide group-bearing substrates afforded good yields with high enantioselectivities (Figs. 6, **2m** and **n**). Terminal alkenyl and alkynyl groups, which are useful not only as tags for imaging^60,61^ and ligation^62,63^ for chemical biology studies but also as platforms for further functionalization^64,65^, also participated in the reaction to afford the products in high yields and with high enantioselectivities (Figs. 6, **2o** and **p**). Actually, **2p** was transformed via copper(I)-catalyzed azide-alkyne cycloaddition (CuAAC)^62,63,66^ in high yield without erosion of its optical purity, which demonstrated the utility of click ligation for the accumulation of functional structures on the obtained products (Fig. 7). Other substrates we investigated are also mentioned in Supplementary Scheme 7.

**Fig. 8:**
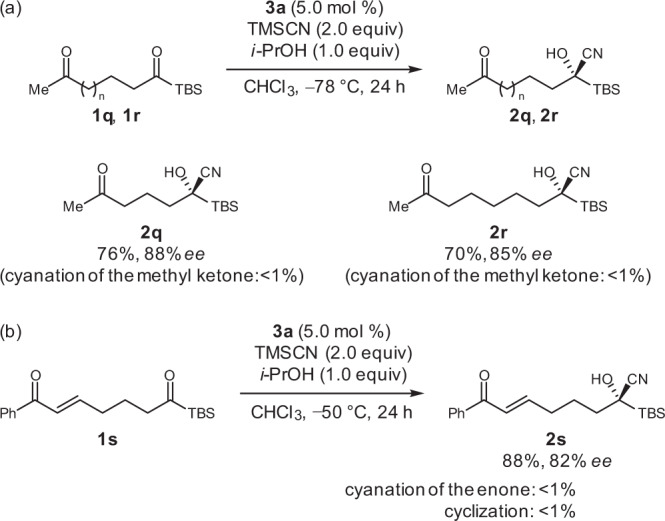
Site- and enantioselective cyanation. **a** Cyanation of acylsilanes bearing methyl ketone moieties. **b** Cyanation of an acylsilane bearing an enone moiety.

Furthermore, the site-selectivity between different carbonyl groups was demonstrated (Fig. 8). The acylsilane cyanohydrins **2q** and **2r** were obtained in high yields and with good enantioselectivities with intact methyl ketone moieties, which are difficult to achieve using conventional catalysis for the cyanation of ketones (Fig. 8a)^1–15^. This was attributed to the high electrophilicity of the acylsilanes^67–69^ in combination with the non-enzymatic but enzyme-like characteristics of the mild organocatalysts^70^. Moreover, a substrate bearing an enone moiety, which was used in our kinetic resolution approach (Fig. 1c)^59^, was site- and enantioselectively cyanated without cyclization as well as cyanation at the enone moiety to provide the optically active cyanohydrin **2s** in a yield exceeding 50%, which demonstrates the utility of the current cyanation process involving enantioselective 1,2-addition (Fig. 8b). The absolute configuration of **2a** was determined by X-ray crystallography (see Supplementary Fig. 129 and Supplementary Data 1 for details), and the configurations of all the other products were assigned analogously.

### Catalytic silylation and transformations of the acylsilane cyanohydrin products

To transform acylsilane cyanohydrins, it is important to establish a method for protecting the alcohol moiety. As acylsilane cyanohydrins are unstable against the Brook rearrangement, mild conditions are required, and strongly basic conditions should be avoided. Additionally, as the bulky silyl groups of acylsilane cyanohydrins retard the protection (some conventional methods for alcohol protection were ineffective when applied on **2a**; see Supplementary Scheme 8 for details), which is consistent with the formation of the non-silylated product **2a** when cyanation was performed using the catalysts **3a**–**3g** (Table 1, entries 1–7), an active catalyst is necessary for protection. According to entry 8 of Table 1, only sparteine (**3h**) afforded the cyanated product as a silyl ether. Inspired by these results, the sparteine-catalyzed silylation of the optically active acylsilane cyanohydrin was investigated (Table 3). As expected, the **3h**-catalyzed silylation of **2a** proceeded smoothly (Table 3, entry 1). Other diamines were also examined. Tetramethylethylenediamine (**6**) was inactive as the catalyst (Table 3, entry 2), and 1,8-bis(dimethylamino)naphthalene (**7**) provided a lower yield (Table 3, entry 3). In addition, other silylation reagents did not react in the presence of the catalyst **3h** (Table 3, entries 4–6). Thus, the use of TMSCN in conjunction with the catalyst **3h** was established as a reliable method for the protection of the acylsilane cyanohydrins. The product **5a** was obtained in 97% yield upon lowering the catalyst loading at –40 °C (Table 3, entry 7). Moreover, the optical purity of the optically active compound **2a** was maintained during the reaction (Table 3, entry 8; see also Supplementary Scheme 9). This method proved to be effective not only at –40 °C (Table 3, entry 8), but also at an ambient temperature (Table 3, entry 9) and produced **5a** in a quantitative yield without any side-reaction while maintaining the enantiomeric purity. Therefore, the method is synthetically versatile depending on the stability and reactivity of the alcohol substrates (see also Supplementary Scheme 10).

**Table 3:** Catalytic silylation of 2a^a^.


Entry	Catalyst (mol%)	TMSX	Yield (%)^b^
1^c^	**3h** (20)	TMSCN	78
2^c^	**6** (20)	TMSCN	< 1
3^c^	**7** (20)	TMSCN	15
4^c^	**3h** (20)	TMSCl	<1
5^c^	**3h** (20)	TMSBr	<1
6^c^	**3h** (20)	TMSOTf	<1
7^c,d^	**3h** (5.0)	TMSCN	97
8^d,e^	**3h** (5.0)	TMSCN	99 (92% *ee*)
9^f,g^	**3h** (5.0)	TMSCN	99 (91% *ee*)

^a^Reactions were run using **2a** (0.20 mmol), TMSX (0.40 mmol), and the catalyst in CHCl_3_ (0.20 mL).

^b^Isolated yields.

^c^Reactions were run using racemic **2a**.

^d^Reactions were run at –40 °C.

^e^Reaction was run using **2a** with 93% *ee*.

^f^Reaction was run at 25 °C.

^g^Reaction was run using **2a** with 91% *ee*.

**Fig. 9:**
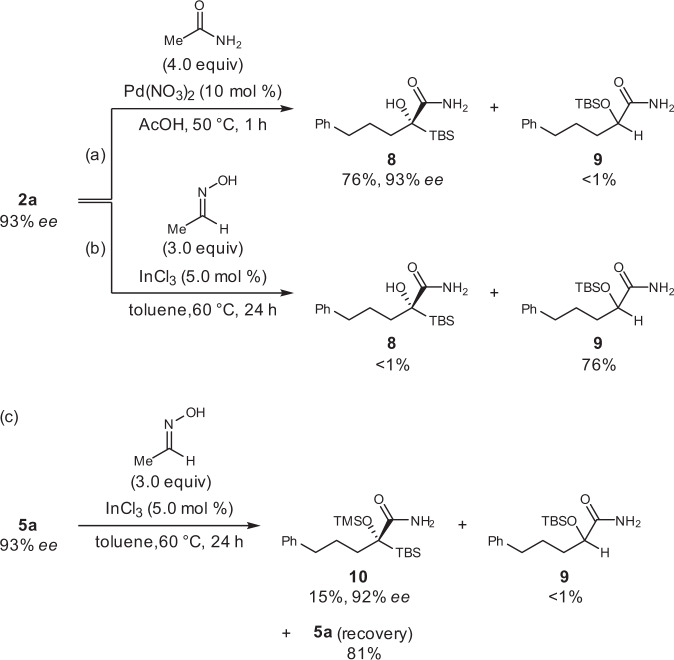
Hydration of 2a and 5a. **a** Hydration of **2a** using acetamide with palladium nitrate. **b** Hydration of **2a** using acetaldoxime with indium chloride. **c** Hydration of **5a** using acetaldoxime with indium chloride.

The transformation of the unprotected cyanohydrin **2a** and protected cyanohydrin **5a** was demonstrated through the synthesis of the corresponding amides using two established hydration methods (Fig. 9)^71,72^. When the method using acetamide with palladium nitrate as a catalyst was employed^71^, the nitrile **2a** was transformed to the amide **8** in 76% yield while maintaining the enantiomeric purity without the formation of the Brook side-product **9** due to the acidic conditions (Fig. 9a). On the other hand, when **2a** was subjected to less acidic conditions, which is desirable for the synthesis of some multifunctional molecules, using acetaldoxime with indium chloride as a catalyst^72^, the Brook side-product **9** was obtained in 76% yield without the formation of **8** (Fig. 9b). The latter method also allowed the transformation of the nitrile **5a** to the amide **10** without the loss of enantiomeric purity, albeit with a low conversion ratio under the current conditions, and the Brook rearrangement was completely suppressed (Fig. 9c). These facts indicate that the sparteine-catalyzed silylation outlined in Table 3 further expands the synthetic utility of the optically active acylsilane cyanohydrins, a variety of which are now available through the **3a**-catalyzed cyanation developed in this study.

## Conclusion

In summary, the non-enzymatic catalytic asymmetric cyanation of acylsilanes was accomplished using the chiral Lewis base as the catalyst, TMSCN as the cyanating reagent, and *i*-PrOH as the additive to drive catalyst turnover. High enantio- and site-selectivities were achieved in a catalytic manner, and a variety of functional groups were installed in the optically active acylsilane cyanohydrins, which overcame the limitations imposed by substrate specificity in conventional enzymatic methods. Moreover, a handle for the synthetic application of the products was established through the development of catalytic methods for the silylation of unstable and difficult to protect alcohols. These synthetic methods provide tetrasubstituted chiral carbon centers integrating multiple functional groups, including silyl, cyano, hydroxy, and functionalized alkyl groups. An efficient catalytic approach was thus developed for the preparation of potential building blocks for the synthesis of pharmaceutically relevant chiral organosilanes.

## Methods

### General procedure for aymmetric cyanation of acylsilanes 1

To a 5-mL vial were sequentially added acylsilane **1** (0.20 mmol), CHCl_3_ (0.10 mL), TMSCN (50 μL, 0.40 mmol), and *i*-PrOH (15 μL, 0.20 mmol). After the reaction mixture was stirred at –78 °C for 15 min, a solution of **3a** (8.6 mg, 0.010 mmol) in CHCl_3_ (0.10 mL) was added. The mixture was stirred for 24 h. The reaction mixture was subsequently diluted with EtOAc, passed through a short silica gel pad, and concentrated in vacuo. Purification of the crude product by flash silica gel column chromatography using hexane/EtOAc (v/v = 2:1–20:1) as an eluent afforded the corresponding acylsilane cyanohydrin **2**.

### Procedure for trimetylsilylation of acylsilane cyanohydrin 2a

To a 5-mL vial were sequentially added acylsilane cyanohydrin **2a** (58 mg, 0.20 mmol), CHCl_3_ (0.20 mL), and (–)-sparteine (2.3 mg, 0.010 mmol). After the reaction mixture was stirred at –40 °C for 30 min, TMSCN (50 μL, 0.40 mmol) was added. The mixture was stirred for 24 h. The reaction mixture was subsequently diluted with EtOAc, passed through a short silica gel pad, and concentrated in vacuo. Purification of the crude product by flash silica gel column chromatography using hexane/EtOAc (v/v = 20:1) as an eluent afforded the corresponding trimethylsilyl ether **5a**.

## Supplementary information

Supplementary Information

Supplementary Data 1

Description of Additional Supplementary Files

## Data Availability

Additional data supporting the findings described in this manuscript are available in the Supplementary Information. For full characterization data of new compounds and experimental details, see Supplementary Methods. For the ^1^H and ^13^C NMR spectra of new compounds, see Supplementary Figs. 1–104. For HPLC chromatogram profiles of the reaction products, see Supplementary Figs. 105–128. For an ORTEP drawing of **2a**, see Supplementary Fig. 129. For the cif, see Supplementary Data 1. X-ray crystallographic data have also been deposited in Cambridge Crystallographic Data Centre (http://www.ccdc.cam.ac.uk/) with the accession code CCDC 2112913. All other data are available from the authors upon reasonable request.
