# Supplementary material for: Non-enzymatic catalytic asymmetric cyanation of acylsilanes
Source: Commun Chem. 2022 Mar 31;5:45. doi: 10.1038/s42004-022-00662-y (PMC9814240; doi:10.1038/s42004-022-00662-y)
Supplement: Supplementary file 1 — Supplementary Information [file 42004_2022_662_MOESM1_ESM.pdf]

Supplementary Information

Non-enzymatic catalytic asymmetric cyanation of acylsilanes

Tagui Nagano, Akira Matsumoto, Ryotaro Yoshizaki, Keisuke Asano,* and Seijiro Matsubara*

Department of Material Chemistry, Graduate School of Engineering, Kyoto University, Kyotodaigaku-Katsura, Nishikyo, Kyoto 615-8510, Japan

Contents

Supplementary Methods **S2**

Supplementary Discussion **S43**

Charactarization Data of Products **S58**

NMR Spectra (^{1}H, ^{13}C) of Substrates **S71**

NMR Spectra (^{1}H, ^{13}C) of Products **S113**

HPLC Chromatogram Profiles **S175**

ORTEP Drawing of 2a **S199**

Supplementary References **S200**

Supplementary Methods

Instrumentation and Chemicals

^{1}H and ^{13}C Nuclear magnetic resonance spectra were taken on a Varian UNITY INOVA 500 (^{1}H, 500 MHz; ^{13}C, 125.7 MHz) spectrometer using tetramethylsilane as an internal standard for ^{1}H NMR (δ = 0 ppm) and $CDCl_3$ as an internal standard for ^{13}C NMR (δ = 77.0 ppm). When a ^{13}C NMR spectrum was measured using C_6D_6 as a solvent, C_6D_6 was used as an internal standard (δ =128.06 ppm). ^{1}H NMR data are reported as follows: chemical shift, multiplicity (s = singlet, d = doublet, t = triplet, q = quartet, quint = quintet, sext = sextet, sept = septet, br = broad, m = multiplet), coupling constants (Hz), integration. ^{19}F NMR spectra were measured on a Varian Mercury 200 (^{19}F, 188 MHz) spectrometer with hexafluorobenzene as an internal standard (δ = 0 ppm). Mass spectra were recorded on a SHIMADZU GCMS-QP2010 Plus (EI) and a Thermo Scientific Exactive (ESI, APCI) spectrometers (analyzer type: TOF). High performance liquid chromatography (HPLC) was performed with a SHIMADZU Prominence. Infrared (IR) spectra were determined on a SHIMADZU IR Affinity-1 spectrometer. Melting points were determined using a YANAKO MP-500D. Optical rotations were measured on a HORIBA SEPA-200. X-ray data were taken on a Rigaku XtaLAB mini diffractometer equipped with a CCD detector. TLC analyses were performed by means of Merck Kieselgel 60 F_{254} (0.25 mm) Plates. Visualization was accomplished with UV light (254 nm) and/or such as an aqueous alkaline $KMnO_4$ solution followed by heating.

Flash column chromatography was carried out using Kanto Chemical silica gel (spherical, 40–50 μm). Unless otherwise noted, commercially available reagents were used without purification.

Experimental Procedure

General procedure for asymmetric cyanation of acylsilanes 1

To a 5-mL vial were sequentially added acylsilane **1** (0.20 mmol), $CHCl_3$ (0.10 mL), TMSCN (50 μL, 0.40 mmol), and *i*-PrOH (15 μL, 0.20 mmol). After the reaction mixture was stirred at –78 °C for 15 min, a solution of **3a** (8.6 mg, 0.010 mmol) in $CHCl_3$ (0.10 mL) was added. The mixture was stirred for 24 h. The reaction mixture was subsequently diluted with EtOAc, passed through a short silica gel pad, and concentrated in vacuo. Purification of the crude product by flash silica gel column chromatography using hexane/EtOAc (v/v = 2:1–20:1) as an eluent afforded the corresponding acylsilane cyanohydrin **2**.

The enantiomeric excesses of **2i**, **2o**, **2p**, **2q**, and **2r** were determined by analyzing those of products **2'** after addition to *p*-toluenesulfonyl isocyanate.

General procedure for synthesis of 2'[1]

To a 5-mL vial were sequentially added **2** (1.0 equiv), $CHCl_3$ (1.0 M), and *p*-toluenesulfonyl isocyanate (2.0 equiv). The mixture was stirred at ambient temperature for 24 h. The reaction mixture was subsequently diluted with EtOAc, passed through a short silica gel pad, and concentrated in vacuo. Purification of the crude product by flash silica gel column chromatography using hexane/EtOAc (v/v = 3:1–10:1) as an eluent afforded **2'**.

Procedure for trimetylsilylation of acylsilane cyanohydrin 2a

To a 5-mL vial were sequentially added acylsilane cyanohydrin **2a** (58 mg, 0.20 mmol), $CHCl_3$ (0.20 mL), and (–)-sparteine (2.3 mg, 0.010 mmol). After the reaction mixture was stirred at –40 °C for 30 min, TMSCN (50 μL, 0.40 mmol) was added. The mixture was stirred for 24 h. The reaction mixture was subsequently diluted with EtOAc, passed through a short silica gel pad, and concentrated in vacuo. Purification of the crude product by flash silica gel column chromatography using hexane/EtOAc (v/v = 20:1) as an eluent afforded the corresponding trimethylsilyl ether **5a**.

The enantiomeric excess of **5a** was determined by analyzing that of **2a** after desilylation.

Procedure for desilylation of 5a

To a 5-mL vial were sequentially added **5a** (1.0 equiv), THF (0.1 M), H_2O (several drops), and trimethylsilyl trifluoromethanesulfonate (3.0 equiv). The mixture was stirred at ambient temperature for 2 h. The reaction mixture was subsequently diluted with EtOAc, passed through a short silica gel pad, and concentrated in vacuo. Purification of the crude product by flash silica gel column chromatography using hexane/EtOAc (v/v = 10:1) as an eluent afforded **2a**.

General procedure for preparation of acylsilanes 1a–1d[1]

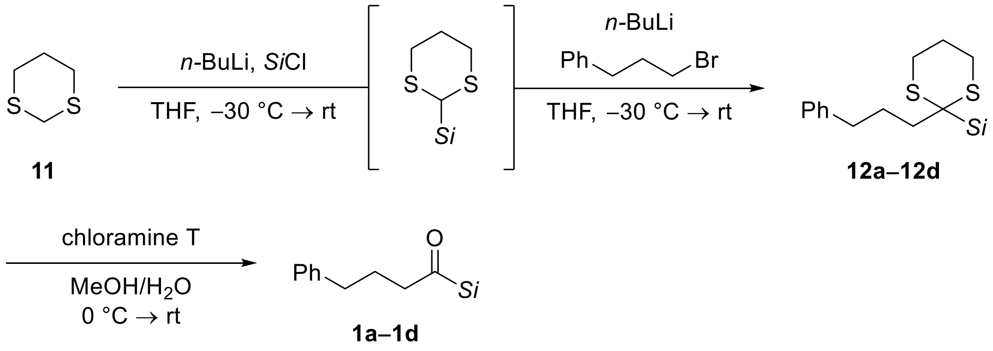

<u>General procedure for preparation of **12a**–**12d**</u>

To a solution of **11** (1.0 equiv) in dry THF (0.50 M) was slowly added *n*-butyllithium (1.1 equiv, 1.60 M in hexane) at –30 °C. After the mixture was stirred for 30 min, a solution of a chlorosilane (1.2 equiv) in dry THF (1.10 M) was added (30 mmol) in dry THF (20 mL) was added, and the reaction mixture was allowed to warm to ambient temperature. After being stirred overnight, the reaction mixture was cooled to –30 °C, and then *n*-butyllithium (1.1 equiv, 1.60 M in hexane) was slowly added. After the mixture was stirred for 30 min, 1-bromo-3-phenylpropane (1.2 equiv) was added at –30 °C, and the reaction mixture was allowed to warm to ambient temperature. After being stirred for 5 h, the reaction was quenched with H_2O (0.10 L). The aqueous layers were extracted with Et_2O (0.10 L × 3), and the combined organic layers were washed with brine, dried over Na_2SO_4, and concentrated in vacuo. Purification by flash silica gel column chromatography using hexane/EtOAc (v/v = 20:1–10:1) as an eluent gave **12**.

***tert*-Butyldimethyl(2-(3-phenylpropyl)-1,3-dithian-2-yl)silane (12a)**.

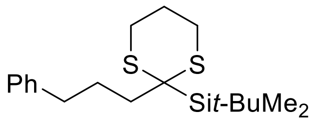

Colorless oil; 78% yield (8.3 g).
^{1}H NMR ($CDCl_3$) δ 7.29 (dd, *J* = 8.0, 7.5 Hz, 2H), 7.21 (d, *J* = 8.0 Hz, 2H), 7.19 (t, *J* = 7.5 Hz, 1H), 2.89 (m, 2H), 2.67 (t, *J* = 7.5 Hz, 2H), 2.36–2.32 (m, 4H), 1.97–1.82 (m, 4H), 1.02 (s, 9H), 0.19 (s, 6H). ^{13}C NMR ($CDCl_3$) δ 142.1, 128.5, 128.3, 125.8, 40.9, 37.2, 36.1, 29.6, 28.3, 24.9, 23.4, 19.8, –5.3. TLC: R_f 0.63 (hexane/EtOAc = 10:1). IR (neat): 2950, 1604, 1471, 1423, 1257, 1013, 913, 823, 699, 487 cm^{-1}. HRMS (ESI) Calcd for $C_{19}H_{32}S_2SiNa$: [M+Na]$^+$, 375.1607. Found: *m*/*z* 375.1610.

Trimethyl(2-(3-phenylpropyl)-1,3-dithian-2-yl)silane (12b): CAS RN [2409952-74-3].

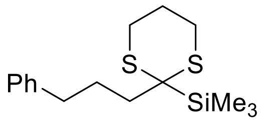

White solid; 84% yield (1.3 g).

^{1}H NMR ($CDCl_3$) δ 7.29 (t, *J* = 7.5 Hz, 2H), 7.22–7.17 (m, 3H), 2.88 (m, 2H), 2.68 (t, *J* = 7.5 Hz, 2H), 2.38 (m, 2H), 2.22 (m, 2H), 1.99 (m, 1H), 1.89–1.81 (m, 3H), 0.16 (s, 9H). ^{13}C NMR ($CDCl_3$) δ 142.1, 128.5, 128.3, 125.8, 38.7, 36.4, 36.1, 29.1, 25.1, 23.3, –2.6. TLC: R_f 0.15 (hexane/EtOAc = 20:1).

Triethyl(2-(3-phenylpropyl)-1,3-dithian-2-yl)silane (12c).

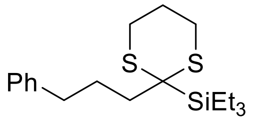

White solid; 68% yield (1.2 g).

^{1}H NMR ($CDCl_3$) δ 7.29 (dd, *J* = 7.5, 7.0 Hz, 2H), 7.23 (d, *J* = 7.0 Hz, 2H), 7.19 (t, *J* = 7.5 Hz, 1H), 2.89 (m, 2H), 2.68 (t, *J* = 7.5 Hz, 2H), 2.32 (m, 2H), 2.25 (m, 2H), 1.99–1.82 (m, 4H), 1.03 (t, *J* = 8.0 Hz, 9H), 0.76 (q, *J* = 8.0 Hz, 6H). ^{13}C NMR ($CDCl_3$) δ 142.1, 128.5, 128.3, 125.8, 39.7, 36.7, 36.1, 29.5, 25.2, 23.4, 8.2, 2.9. TLC: R_f 0.50 (hexane/EtOAc = 10:1). Mp. 31.0–31.5 °C. IR (KBr): 2958, 1495, 1455, 1422, 1239, 1015, 817, 701, 665, 540 cm^{-1}. HRMS (ESI) Calcd for $C_{19}H_{32}S_2SiNa$: $[M+Na]^+$, 375.1607. Found: *m*/*z* 375.1614.

Dimethyl(phenyl)(2-(3-phenylpropyl)-1,3-dithian-2-yl)silane (12d).

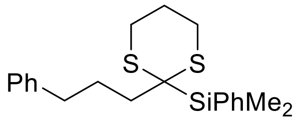

Colorless oil; 73% yield (1.4 g).

^{1}H NMR ($CDCl_3$) δ 7.61 (d, *J* = 8.0 Hz, 2H), 7.42–7.34 (m, 3H), 7.26 (dd, *J* = 7.5, 7.0 Hz, 2H), 7.17 (t, *J* = 7.0 Hz, 1H), 7.11 (d, *J* = 7.5 Hz, 2H), 2.83 (m, 2H), 2.54 (t, *J* = 7.5 Hz, 2H), 2.38 (m, 2H), 2.10 (m, 2H), 1.94 (m, 1H), 1.86 (m, 1H), 1.73 (m, 2H), 0.49 (s, 6H). ^{13}C NMR ($CDCl_3$) δ 142.0, 135.5, 134.8, 129.6, 128.5, 128.2, 127.6, 125.7, 39.0, 36.4, 35.9, 28.9, 24.8, 23.6, –4.0. TLC: R_f 0.50 (hexane/EtOAc = 10:1). IR (neat): 3068, 2910, 1959, 1603, 1427, 1249, 1115, 827, 700, 490 cm^{-1}. HRMS (ESI) Calcd for $C_{21}H_{28}S_2SiNa$: $[M+Na]^+$, 395.1294. Found: *m*/*z* 395.1299.

General procedure for preparation of **1a**–**1d**

To a solution of **12** (1.0 equiv) in MeOH/H_2O (v/v = 4:1, 1.0 M) was slowly added a solution of chloramine T trihydrate (4.0 equiv) in MeOH/H_2O (v/v = 4:1, 0.25 M) at 0 °C, and the reaction mixture was allowed to warm to ambient temperature. After being stirred for 2 h, the reaction was quenched with H_2O (0.10 L). The aqueous layers were extracted with $CHCl_3$ (0.10 L × 3), and the combined organic layers were dried over Na_2SO_4 and concentrated in vacuo. Purification by flash silica gel column chromatography using hexane/EtOAc (v/v = 20:1–10:1) as an eluent gave **1**.

1-(*tert*-Butyldimethylsilyl)-4-phenylbutan-1-one (1a).

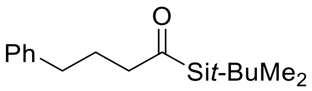

Colorless oil; 57% yield (3.4 g).

^{1}H NMR ($CDCl_3$) δ 7.27 (m, 2H), 7.19–7.12 (m, 3H), 2.62 (t, *J* = 7.0 Hz, 2H), 2.58 (t, *J* = 7.5 Hz, 2H), 1.85 (tt, *J* = 7.5, 7.0 Hz, 2H), 0.91 (s, 9H), 0.16 (s, 6H). ^{13}C NMR ($CDCl_3$) δ 247.4, 141.9, 128.4, 128.3, 125.8, 49.4, 35.2, 26.4, 23.5, 16.5, –7.0. TLC: R_f 0.25 (hexane/$CHCl_3$ = 3:1). IR (neat): 2932, 1641, 1474, 1364, 1249, 1007, 837, 775, 699, 484 cm^{-1}. HRMS (ESI) Calcd for $C_{16}H_{26}OSiNa$: [M+Na]$^+$, 285.1645. Found: *m*/*z* 285.1641.

4-Phenyl-1-(trimethylsilyl)butan-1-one (1b): CAS RN [142981-60-0].

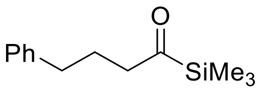

Colorless oil; 23% yield (0.22 g).

^{1}H NMR ($CDCl_3$) δ 7.28 (m, 2H), 7.20–7.15 (m, 3H), 2.62 (t, *J* = 7.5 Hz, 2H), 2.59 (t, *J* = 8.0 Hz, 2H), 1.85 (tt, *J* = 8.0, 7.5 Hz, 2H), 0.17 (s, 9H). ^{13}C NMR ($CDCl_3$) δ 248.1, 141.8, 128.4, 128.3, 125.8, 47.5, 35.2, 23.7, –3.2. TLC: R_f 0.48 (hexane/EtOAc = 10:1).

4-Phenyl-1-(triethylsilyl)butan-1-one (1c).

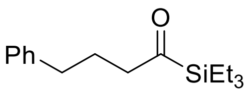

Colorless oil; 35% yield (0.27 g).

^{1}H NMR ($CDCl_3$) δ 7.27 (m, 2H), 7.19–7.15 (m, 3H), 2.60 (t, *J* = 8.0 Hz, 2H), 2.57 (t, *J* = 7.0 Hz, 2H), 1.85 (tt, *J* = 8.0, 7.0 Hz, 2H), 0.95 (t, *J* = 7.5 Hz, 9H), 0.71 (q, *J* = 7.5 Hz, 6H). ^{13}C NMR ($CDCl_3$) δ 247.9, 141.9, 128.4, 128.3, 125.8, 49.2, 35.2, 23.5, 7.2, 2.1.

TLC: R_f 0.43 (hexane/EtOAc = 10:1). IR (neat): 2955, 1641, 1497, 1457, 1415, 1237, 1019, 734, 699, 495 cm^{-1}. HRMS (EI) Calcd for $C_{16}H_{25}OSi$: $[M–H]^+$, 261.1674. Found: *m*/*z* 261.1673.

1-(Dimethyl(phenyl)silyl)-4-phenylbutan-1-one (1d): CAS RN [205752-93-8].

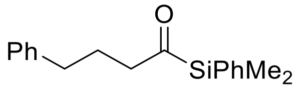

Colorless oil; 49% yield (0.41 g).

^1H NMR ($CDCl_3$) δ 7.53 (d, *J* = 7.0 Hz, 2H), 7.42–7.37 (m, 3H), 7.25 (m, 2H), 7.16 (t, *J* = 7.0 Hz, 1H), 7.05 (d, *J* = 7.5 Hz, 2H), 2.59 (t, *J* = 7.0 Hz, 2H), 2.49 (t, *J* = 8.0 Hz, 2H), 1.78 (tt, *J* = 8.0, 7.0 Hz, 2H), 0.67 (s, 6H). ^{13}C NMR ($CDCl_3$) δ 246.1, 141.8, 134.5, 134.0, 129.9, 128.4, 128.3, 128.2, 125.8, 47.9, 35.1, 23.7, –4.8. TLC: R_f 0.30 (hexane/Et_2O = 10:1).

Procedure for preparation of acylsilane 1e

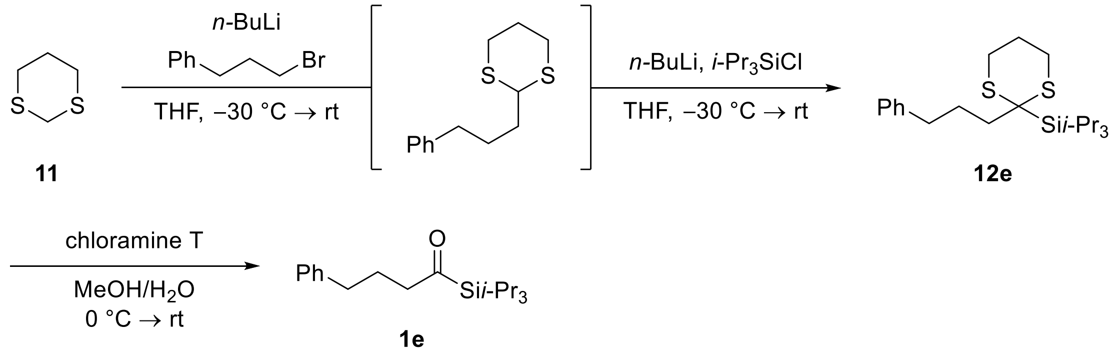

<u>Procedure for preparation of **12e**</u>

To a solution of **11** (0.60 g, 5.0 mmol) in dry THF (15 mL) was slowly added *n*-butyllithium (3.6 mL, 1.60 M in hexane, 5.5 mmol) at –30 °C. After the mixture was stirred for 30 min, a solution of 1-bromo-3-phenylpropane (0.91 mL, 6.0 mmol) was added, and the reaction mixture was allowed to warm to ambient temperature. After being stirred for 5 h, the mixture was cooled to –30 °C, and then *n*-butyllithium (3.6 mL, 1.60 M in hexane, 5.5 mmol) was slowly added. After the mixture was stirred for 30 min, chlorotriisopropylsilane (1.1 mL, 5.0 mmol) was added at –30 °C, and the reaction mixture was allowed to warm to ambient temperature. After being stirred overnight, the reaction was quenched with H_2O (0.10 L). The aqueous layers were extracted with Et_2O (0.10 L × 3), and the combined organic layers were washed

with brine, dried over Na_2SO_4, and concentrated in vacuo. Purification by flash silica gel column chromatography using hexane/EtOAc (v/v = 20:1) as an eluent gave **12e**.

Triisopropyl(2-(3-phenylpropyl)-1,3-dithian-2-yl)silane (12e).

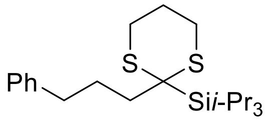

White solid; 79% yield (1.6 g).
^{1}H NMR ($CDCl_3$) δ 7.29 (dd, J = 7.0, 7.0 Hz, 2H), 7.22 (d, J = 7.0 Hz, 2H), 7.19 (t, J = 7.0 Hz, 1H), 2.91 (m, 2H), 2.69 (t, J = 7.5 Hz, 2H), 2.37–2.31 (m, 4H), 2.00–1.88 (m, 4H), 1.40 (sept, J = 7.0 Hz, 3H), 1.20 (d, J = 7.0 Hz, 18H). ^{13}C NMR ($CDCl_3$) δ 142.1, 128.6, 128.3, 125.8, 41.4, 38.5, 36.2, 29.9, 24.7, 24.3, 20.0, 12.4. TLC: R_f 0.55 (hexane/EtOAc = 10:1). Mp. 46.0–46.5 °C. IR (KBr): 2947, 1463, 1246, 1023, 882, 700, 658, 616, 569, 486 cm^{-1}. HRMS (ESI) Calcd for $C_{22}H_{38}S_2SiNa$: $[M+Na]^+$, 417.2076. Found: m/z 417.2083.

<u>Procedure for preparation of **1e**</u>

To a solution of **12e** (1.4 g, 3.5 mmol) in MeOH/H_2O (v/v = 4:1, 7.0 mL) was slowly added a solution of chloramine T trihydrate (3.9 g, 14 mmol) in MeOH/H_2O (v/v = 4:1, 28 mL) at 0 °C, and the reaction mixture was allowed to warm to ambient temperature. After being stirred for 2 h, the reaction was quenched with H_2O (0.10 L). The aqueous layers were extracted with $CHCl_3$ (0.10 L× 3), and the combined organic layers were dried over Na_2SO_4 and concentrated in vacuo. Purification by flash silica gel column chromatography using hexane/EtOAc (v/v = 30:1) as an eluent gave **1e**.

4-Phenyl-1-(triisopropylsilyl)butan-1-one (1e).

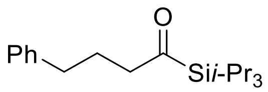

Colorless oil; 24% yield (0.26 g).
^{1}H NMR ($CDCl_3$) δ 7.27 (m, 2H), 7.18–7.16 (m, 3H), 2.60 (t, J = 7.0 Hz, 2H), 2.58 (t, J = 7.5 Hz, 2H), 1.86 (tt, J = 7.5, 7.0 Hz, 2H), 1.26 (sept, J = 7.5 Hz, 3H), 1.08 (d, J = 7.5 Hz, 18H). ^{13}C NMR ($CDCl_3$) δ 247.3, 142.0, 128.4, 128.3, 125.8, 50.5, 35.3, 23.3, 18.5, 10.7. TLC: R_f 0.23 (hexane/EtOAc = 30:1). IR (neat): 2945, 1636, 1455, 1265, 1072, 1018, 882, 740, 653, 506 cm^{-1}. HRMS (EI) Calcd for $C_{19}H_{31}OSi$: $[M–H]^+$, 303.2144. Found: m/z 303.2140.

Procedure for preparation of acylsilane 1f

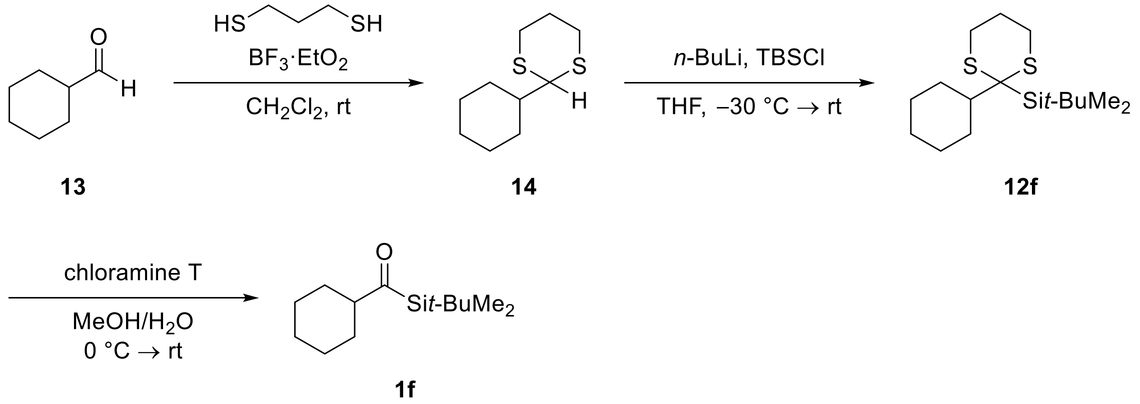

Procedure for preparation of **14**

To a solution of cyclohexanecarboxaldehyde (**13**, 1.8 mL, 15 mmol) in dry THF (45 mL) were added 1,3-propanedithiol (1.6 mL, 16 mmol) and boron trifluoride diethyl etherate (0.94 mL, 7.5 mmol) at 0 °C. After being stierred at ambient temperature overnight, the reaction was quenched with H_2O (50 mL). The aqueous layers were extracted with CH_2Cl_2 (50 mL × 3), and the combined organic layers were washed with brine, dried over Na_2SO_4, and concentrated in vacuo. The residue was recrystallized from CH_2Cl_2/hexane to give **14**.

2-Cyclohexyl-1,3-dithiane (14): CAS RN [56698-00-1].

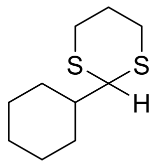

White solid; 95% yield (2.9 g).

^{1}H NMR ($CDCl_3$) δ 4.04 (d, J = 5.5 Hz, 1H), 2.91–2.83 (m, 4H), 2.10 (m, 1H), 1.90–1.84 (m, 3H), 1.76 (m, 2H), 1.68 (m, 2H), 1.30–1.13 (m, 5H). ^{13}C NMR ($CDCl_3$) δ 55.3, 43.1, 30.9, 30.4, 26.4, 26.23, 26.17. TLC: R_f 0.63 (hexane/EtOAc = 10:1).

Procedure for preparation of **12f**

To a solution of **14** (1.4 g, 7.0 mmol) in dry THF (14 mL) was slowly added *n*-butyllithium (5.0 mL, 1.60 M in hexane, 7.7 mmol) at –30 °C. After being stirred at –30 °C for 8 h, the reaction mixture was cooled to –78 °C, and a solution of *tert*-butylchlorodimethylsilane (1.2 g, 7.7 mmol) in dry THF (7.0 mL) was added. Subsequently, the reaction mixture was allowed to warm to ambient temperature.

After being stirred overnight, the reaction was quenched with H_2O (0.10 L). The aqueous layers were extracted with Et_2O (0.10 L × 3), and the combined organic layers were washed with brine, dried over Na_2SO_4, and concentrated in vacuo. Purification by flash silica gel column chromatography using hexane/$CHCl_3$ (v/v = 5:1) as an eluent gave **12f**.

***tert*-Butyl(2-cyclohexyl-1,3-dithian-2-yl)dimethylsilane (12f)**.

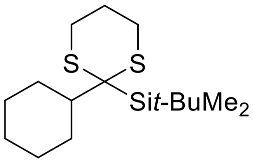

Colorless oil; 27% yield (0.61 g).
^{1}H NMR ($CDCl_3$) δ 2.92 (m, 2H), 2.44 (ddd, J = 14.0, 4.5, 3.5 Hz, 2H), 2.22–2.17 (m, 3H), 2.06 (m, 1H), 1.89–1.83 (m, 3H), 1.66 (m, 1H), 1.36 (m, 2H), 1.28–1.14 (m, 3H), 1.04 (s, 9H), 0.26 (s, 6H). ^{13}C NMR ($CDCl_3$) δ 49.7, 31.4, 28.6, 27.4, 26.3, 26.1 (2C), 24.3, 20.4, –2.9. TLC: R_f 0.40 (hexane/$CHCl_3$ = 5:1). IR (neat): 2939, 1472, 1424, 1364, 1257, 1006, 914, 827, 670, 500 cm^{-1}. HRMS (EI) Calcd for $C_{16}H_{32}S_2Si$: $[M]^+$, 316.1715. Found: m/z 316.1710.

<u>Procedure for preparation of **1f**</u>

To a solution of **12f** (0.57 g, 1.8 mmol) in MeOH/H_2O (v/v = 4:1, 4.0 mL) was slowly added a solution of chloramine T trihydrate (2.0 g, 7.2 mmol) in MeOH/H_2O (v/v = 4:1, 14 mL) at 0 °C, and the reaction mixture was allowed to warm to ambient temperature. After being stirred for 2 h, the reaction was quenched with H_2O (0.10 L). The aqueous layers were extracted with $CHCl_3$ (0.10 L × 3), and the combined organic layers were washed with brine, dried over Na_2SO_4, and concentrated in vacuo. Purification by flash silica gel column chromatography using $CHCl_3$ as an eluent gave **1f**.

(*tert*-Butyldimethylsilyl)(cyclohexyl)methanone (1f).

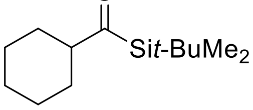

Colorless oil; 89% yield (0.36 g).
^{1}H NMR ($CDCl_3$) δ 2.76 (tt, J = 11.5, 3.0 Hz, 1H), 1.77 (m, 2H), 1.72–1.65 (m, 3H), 1.30–1.13 (m, 5H), 0.92 (s, 9H), 0.19 (s, 6H). ^{13}C NMR ($CDCl_3$) δ 250.7, 56.0, 26.63,

26.56, 26.0, 25.8, 16.8, –6.4. TLC: R_f 0.80 ($CHCl_3$). IR (neat): 2935, 1637, 1464, 1364, 1249, 1139, 979, 838, 767, 492 cm^{-1}. HRMS (ESI) Calcd for $C_{13}H_{26}OSiNa$: $[M+Na]^+$, 249.1645. Found: *m/z* 249.1645.

Procedure for preparation of acylsilane 1g

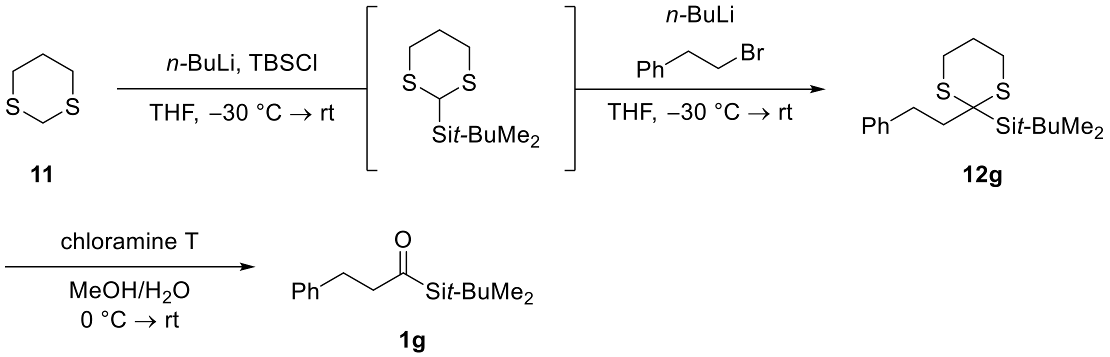

<u>Procedure for preparation of **12g**</u>

To a solution of **11** (2.4 g, 20 mmol) in dry THF (40 mL) was slowly added *n*-butyllithium (14 mL, 1.60 M in hexane, 22 mmol) at –30 °C. After the mixture was stirred for 30 min, a solution of *tert*-butylchlorodimethylsilane (3.3 g, 22 mmol) in dry THF (20 mL) was added, and the reaction mixture was allowed to warm to ambient temperature. After being stirred for 24 h, the reaction mixture was cooled to –30 °C, and then *n*-butyllithium (14 mL, 1.60 M in hexane, 22 mmol) was slowly added. After the mixture was stirred for 30 min, a solution of (2-bromoethyl)benzene (3.2 mL, 24 mmol) in dry THF (20 mL) was added at –30 °C, and the reaction mixture was allowed to warm to ambient temperature. After being stirred for 5 h, the reaction was quenched with H_2O (0.20 L). The aqueous layers were extracted with Et_2O (0.20 L × 3), and the combined organic layers were washed with H_2O and brine, dried over Na_2SO_4, and concentrated in vacuo. Purification by flash silica gel column chromatography using hexane/EtOAc (v/v = 20:1) as an eluent gave **12g**.

***tert*-Butyldimethyl(2-phenethyl-1,3-dithian-2-yl)silane (12g)**: CAS RN [129806-69-5].

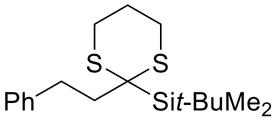

Colorless oil; 99% yield (6.7 g).

^{1}H NMR ($CDCl_3$) δ 7.32 (m, 2H), 7.24–7.20 (m, 3H), 3.08 (m, 2H), 2.88 (m, 2H), 2.61 (m, 2H), 2.43 (m, 2H), 2.03 (m, 1H), 1.93 (m, 1H), 1.10 (s, 9H), 0.28 (m, 6H). ^{13}C NMR ($CDCl_3$) δ 142.4, 128.5, 128.4, 125.9, 41.0, 40.1, 34.5, 28.4, 24.9, 23.3, 19.9, –5.2. TLC: R_f 0.25 (hexane/EtOAc = 20:1).

<u>Procedure for preparation of **1g**</u>

To a solution of **12g** (6.7 g, 20 mmol) in MeOH/H_2O (v/v = 4:1, 0.20 L) was slowly added a solution of chloramine T trihydrate (23 g, 80 mmol) in MeOH/H_2O (v/v = 4:1, 0.32 L) at 0 °C, and the reaction mixture was allowed to warm to ambient temperature. After being stirred for 2 h, the reaction was quenched with H_2O (0.20 L). The aqueous layers were extracted with Et_2O (0.20 L × 3), and the combined organic layers were washed with H_2O and brine, dried over Na_2SO_4, and concentrated in vacuo. Purification by flash silica gel column chromatography using hexane/EtOAc (v/v = 20:1) as an eluent gave **1g**.

1-(*tert*-Butyldimethylsilyl)-3-phenylpropan-1-one (1g): CAS RN [128084-24-2].

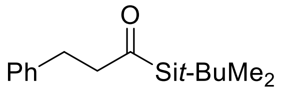

White solid; 11% yield (0.54 g).

^{1}H NMR ($CDCl_3$) δ 7.27 (m, 2H), 7.19–7.16 (m, 3H), 2.92 (t, J = 7.5 Hz, 2H), 2.83 (t, J = 7.5 Hz, 2H), 0.91 (s, 9H), 0.16 (s, 6H). ^{13}C NMR ($CDCl_3$) δ 246.4, 141.8, 128.42, 128.37, 125.9, 52.0, 28.1, 26.4, 16.5, –7.1. TLC: R_f 0.50 (hexane/EtOAc = 20:1).

General procedure for preparation of acylsilanes 1h and 1i

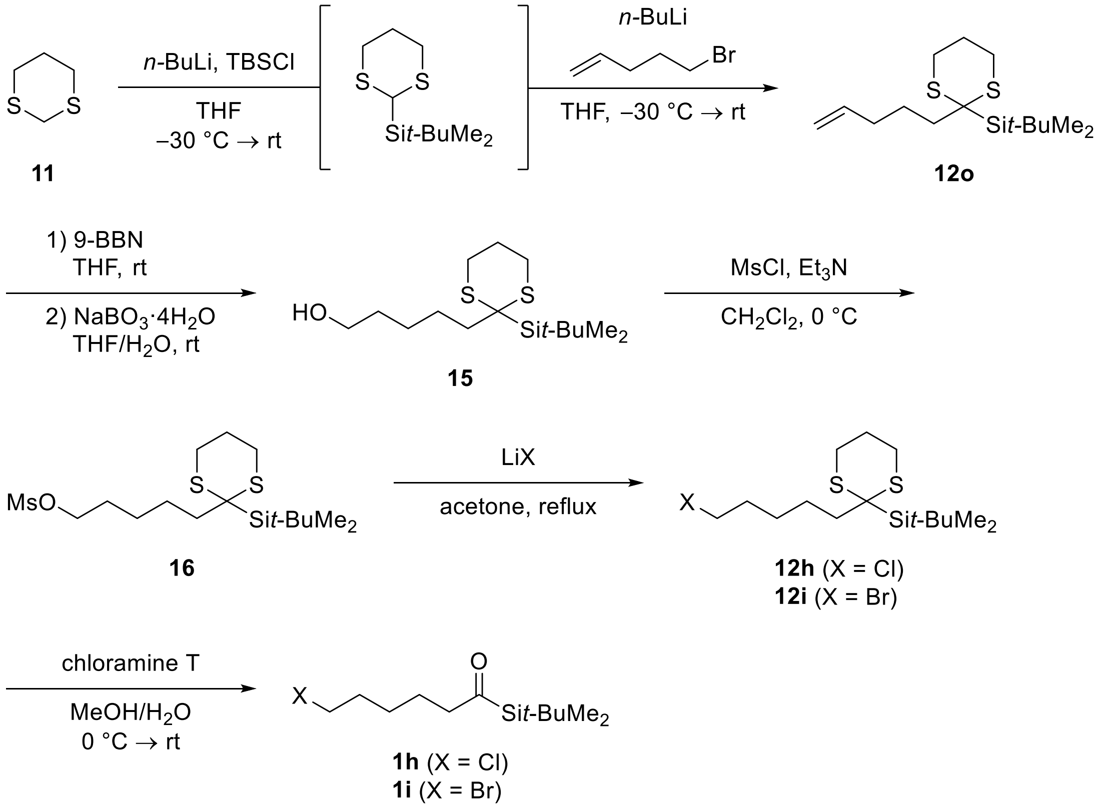

<u>Procedure for preparation of **12o**</u>

To a solution of **11** (3.6 g, 30 mmol) in dry THF (60 mL) was slowly added *n*-butyllithium (21 mL, 1.60 M in hexane, 33 mmol) at –30 °C. After the mixture was stirred for 30 min, a solution of *tert*-butylchlorodimethylsilane (5.0 g, 33 mmol) in dry THF (30 mL) was added, and the reaction mixture was allowed to warm to ambient temperature. After being stirred for 18 h, the reaction mixture was cooled to –30 °C, and then *n*-butyllithium (21 mL, 1.60 M in hexane, 33 mmol) was slowly added. After the mixture was stirred for 30 min, 5-bromo-1-pentene (4.3 mL, 36 mmol) was added at –30 °C, and the reaction mixture was allowed to warm to ambient temperature. After being stirred for 5 h, the reaction was quenched with H_2O (0.10 L). The aqueous layers were extracted with Et_2O (0.10 L × 3), and the combined organic layers were washed with brine, dried over Na_2SO_4, and concentrated in vacuo. Purification by flash silica gel column chromatography using hexane/EtOAc (v/v = 30:1) as an eluent gave **12o**.

***tert*-Butyldimethyl(2-(pent-4-en-1-yl)-1,3-dithian-2-yl)silane (12o)**: CAS RN [2254446-88-1].

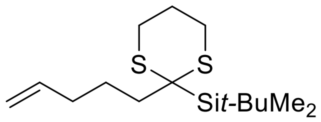

Colorless oil; 75% yield (6.9 g).

^{1}H NMR ($CDCl_3$) δ 5.84 (ddt, *J* = 17.0, 10.0, 7.0 Hz, 1H), 5.05 (dd, *J* = 17.0, 1.5 Hz, 1H), 4.99 (dd, *J* = 10.0, 1.5 Hz, 1H), 3.05 (m, 2H), 2.40 (m, 2H), 2.32 (m, 2H), 2.09 (m, 2H), 2.02 (m, 1H), 1.90 (m, 1H), 1.66 (m, 2H), 1.03 (s, 9H), 0.21 (s, 6H). ^{13}C NMR ($CDCl_3$) δ 138.5, 114.9, 41.0, 37.4, 34.1, 28.3, 27.2, 25.0, 23.5, 19.8, –5.3. TLC: R_f 0.20 (hexane/EtOAc = 30:1).

Procedure for preparation of **15**

To a solution of **12o** (6.1 g, 20 mmol) in dry THF (50 mL) was added 9-borabicyclo[3.3.1]nonane (44 mL, 0.50 M in THF, 22 mmol) at ambient temperature. After the reaction mixture was stirred for 1.5 h, H_2O (50 mL) and sodium perborate tetrahydrate were slowly added. After being stirred for 1.5 h, the reaction was quenched with saturated aqueous NH_4Cl (50 mL). The aqueous layers were extracted with Et_2O (0.10 L × 3), and the combined organic layers were washed with brine, dried over Na_2SO_4, and concentrated in vacuo. Purification by flash silica gel column chromatography using hexane/EtOAc (v/v = 2:1) as an eluent gave **15**.

5-(2-(*tert*-Butyldimethylsilyl)-1,3-dithian-2-yl)pentan-1-ol (15).

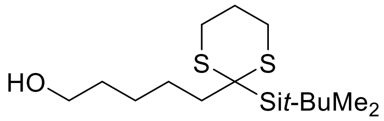

Colorless oil; 99% yield (6.4 g).

^{1}H NMR ($CDCl_3$) δ 3.67 (t, *J* = 6.5 Hz, 2H), 3.05 (m, 2H), 2.40 (m, 2H), 2.33 (m, 2H), 2.03 (m, 1H), 1.90 (m, 1H), 1.62 (m, 4H), 1.42 (m, 2H), 1.36 (s, 1H), 1.04 (s, 9H), 0.21 (s, 6H). ^{13}C NMR ($CDCl_3$) δ 63.0, 40.9, 38.0, 32.7, 28.3, 27.8, 26.3, 25.0, 23.5, 19.8, –5.3. TLC: R_f 0.43 (hexane/EtOAc = 2:1). IR (neat): 3364, 2939, 1474, 1364, 1257, 1055, 911, 822, 691, 464 cm^{-1}. HRMS (ESI) Calcd for $C_{15}H_{32}OS_2SiNa$: $[M+Na]^+$, 343.1556. Found: *m*/*z* 343.1560.

Procedure for preparation of **16**

To a solution of **15** (5.0 g, 16 mmol) in dry CH_2Cl_2 (30 mL) were added triethylamine (2.4 mL, 17 mmol) and methanesulfonyl chloride (1.3 mL, 17 mmol) at 0 °C. After being stirred for 5 h, the reaction was quenched with H_2O (50 mL). The aqueous layers were extracted with CH_2Cl_2 (0.10 L × 3), and the combined organic layers were dried over Na_2SO_4 and concentrated in vacuo. Purification by flash silica gel column chromatography using hexane/EtOAc (v/v = 5:1) as an eluent gave **16**.

5-(2-(*tert*-Butyldimethylsilyl)-1,3-dithian-2-yl)pentyl methanesulfonate (16).

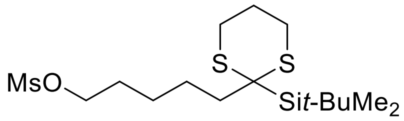

Colorless oil; 89% yield (5.6 g).

^{1}H NMR ($CDCl_3$) δ 4.25 (t, J = 6.5 Hz, 2H), 3.03 (m, 2H), 3.01 (s, 3H), 2.41 (m, 2H), 2.33 (m, 2H), 2.03 (m, 1H), 1.90 (m, 1H), 1.82 (m, 2H), 1.60 (m, 2H), 1.47 (m, 2H), 1.04 (s, 9H), 0.20 (s, 6H). ^{13}C NMR ($CDCl_3$) δ 70.0, 40.8, 37.9, 37.4, 29.2, 28.3, 27.5, 26.0, 24.9, 23.5, 19.8, –5.3. TLC: R_f 0.63 (hexane/EtOAc = 1:1). IR (neat): 2936, 1471, 1360, 1250, 1178, 1016, 952, 824, 694, 492 cm^{-1}. HRMS (ESI) Calcd for $C_{16}H_{34}O_3S_3SiNa$: [M+Na]$^+$, 421.1332. Found: m/z 421.1334.

General procedure for preparation of **12h** and **12i**

To a solution of **16** (1.0 equiv) in dry acetone (0.50 M) was added a lithium halide (3.0 equiv). After being refluxed for 6 h, the reaction was cooled to ambient temperature and quenched with H_2O (50 mL). The aqueous layers were extracted with Et_2O (0.10 L × 3), and the combined organic layers were washed with brine, dried over Na_2SO_4, and concentrated in vacuo. Purification by flash silica gel column chromatography using hexane/EtOAc (v/v = 2:1–5:1) as an eluent gave **12**.

***tert*-Butyl(2-(5-chloropentyl)-1,3-dithian-2-yl)dimethylsilane (12h)**.

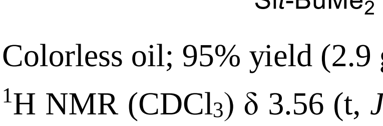

Colorless oil; 95% yield (2.9 g).

^{1}H NMR ($CDCl_3$) δ 3.56 (t, J = 7.0 Hz, 2H), 3.04 (m, 2H), 2.41 (m, 2H), 2.33 (m, 2H), 2.03 (m, 1H), 1.90 (m, 1H), 1.83 (m, 2H), 1.58 (m, 2H), 1.50 (m, 2H), 1.04 (s, 9H), 0.21 (s, 6H). ^{13}C NMR ($CDCl_3$) δ 45.0, 40.9, 37.9, 32.6, 28.3, 27.4, 27.3, 25.0, 23.5, 19.8,

–5.3. TLC: R_f 0.38 (hexane/EtOAc = 10:1). IR (neat): 2941, 1471, 1364, 1250, 1177, 1014, 909, 831, 668, 490 cm^{-1}. HRMS (EI) Calcd for $C_{15}H_{31}ClS_2Si$: $[M]^+$, 338.1325. Found: *m*/*z* 338.1320.

(2-(5-Bromopentyl)-1,3-dithian-2-yl)(*tert*-butyl)dimethylsilane (12i): CAS RN [174710-27-1].

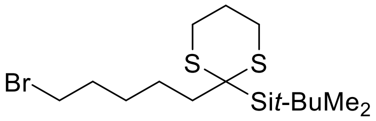

Colorless oil; 99% yield (1.9 g).

^{1}H NMR ($CDCl_3$) δ 3.43 (t, *J* = 6.5 Hz, 2H), 3.04 (m, 2H), 2.41 (m, 2H), 2.33 (m, 2H), 2.02 (m, 1H), 1.95–1.85 (m, 3H), 1.58 (m, 2H), 1.50 (m, 2H), 1.04 (s, 9H), 0.21 (s, 6H). ^{13}C NMR ($CDCl_3$) δ 40.8, 37.9, 33.8, 32.7, 28.7, 28.3, 27.2, 25.0, 23.5, 19.8, –5.3. TLC: R_f 0.35 (hexane/EtOAc = 10:1).

<u>General procedure for preparation of **1h** and **1i**</u>

To a solution of **12** (1.0 equiv) in MeOH/H_2O (v/v = 4:1, 0.50 M) was slowly added a solution of chloramine T trihydrate (4.0 equiv) in MeOH/H_2O (v/v = 4:1, 0.50 M) at 0 °C, and the reaction mixture was allowed to warm to ambient temperature. After being stirred for 2 h, the reaction was quenched with H_2O (0.10 L). The aqueous layers were extracted with Et_2O (0.10 L × 3), and the combined organic layers were dried over Na_2SO_4 and concentrated in vacuo. Purification by flash silica gel column chromatography using hexane/EtOAc (v/v = 10:1) as an eluent gave **1**.

1-(*tert*-Butyldimethylsilyl)-6-chlorohexan-1-one (1h).

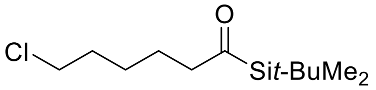

Colorless oil; 53% yield (0.40 g).

^{1}H NMR ($CDCl_3$) δ 3.53 (t, *J* = 7.0 Hz, 2H), 2.61 (t, *J* = 7.0 Hz, 2H), 1.76 (m, 2H), 1.54 (m, 2H), 1.39 (m, 2H), 0.93 (s, 9H), 0.18 (s, 6H). ^{13}C NMR ($CDCl_3$) δ 247.3, 50.0, 44.9, 32.5, 26.6, 26.4, 21.1, 16.5, −7.0. TLC: R_f 0.50 (hexane/EtOAc = 10:1). IR (neat): 2951, 1732, 1641, 1464, 1364, 1250, 1012, 834, 776, 487 cm^{-1}. HRMS (ESI) Calcd for $C_{12}H_{25}ClOSiNa$: $[M+Na]^+$, 271.1255. Found: *m*/*z* 271.1249.

6-Bromo-1-(*tert*-butyldimethylsilyl)hexan-1-one (1i): CAS RN [147752-75-8].

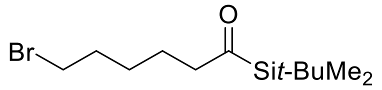

Colorless oil; 29% yield (0.38 g).

^{1}H NMR ($CDCl_3$) δ 3.40 (t, J = 6.5 Hz, 2H), 2.61 (t, J = 7.0 Hz, 2H), 1.85 (m, 2H), 1.54 (m, 2H), 1.39 (m, 2H), 0.93 (s, 9H), 0.18 (s, 6H). ^{13}C NMR ($CDCl_3$) δ 247.3, 50.0, 33.6, 32.7, 27.8, 26.4, 21.0, 16.5, −7.0. TLC: R_f 0.30 (hexane/EtOAc = 10:1).

Procedure for preparation of acylsilane 1j

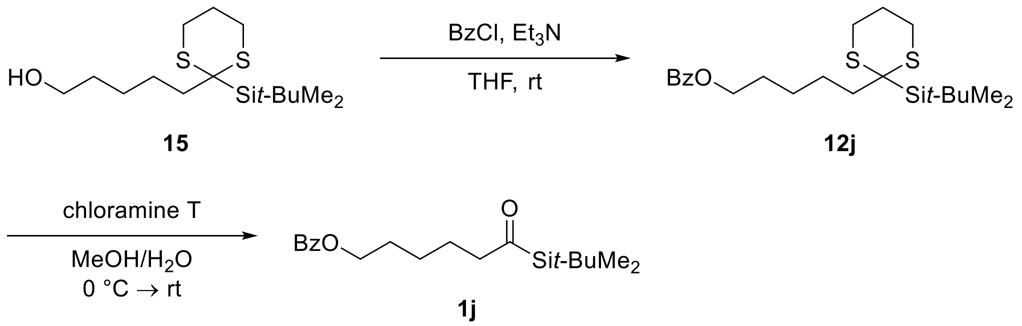

<u>Procedure for preparation of **12j**</u>

To a solution of **15** (1.6 g, 5.0 mmol) in dry THF (25 mL) were slowly added triethylamine (3.5 mL, 25 mmol) and benzoyl chloride (0.87 mL, 7.5 mmol) at 0 °C, and the reaction mixture was allowed to warm to ambient temperature. After being stirred for 14 h, the reaction was quenched with saturated aqueous Na_2CO_3 (50 mL). The aqueous layers were extracted with CH_2Cl_2 (0.10 L × 3), and the combined organic layers were washed with brine, dried over Na_2SO_4, and concentrated in vacuo. Purification by flash silica gel column chromatography using hexane/EtOAc (v/v = 10:1) as an eluent gave **12j**.

5-(2-(*tert*-Butyldimethylsilyl)-1,3-dithian-2-yl)pentyl benzoate (12j).

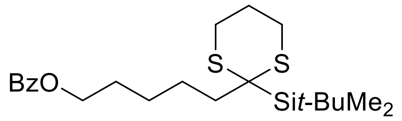

Colorless oil; 98% yield (2.1 g).

^{1}H NMR ($CDCl_3$) δ 8.05 (d, J = 7.0 Hz, 2H), 7.56 (t, J = 7.5 Hz, 1H), 7.45 (dd, J = 7.5, 7.0 Hz, 2H), 4.35 (t, J = 7.0 Hz, 2H), 3.04 (m, 2H), 2.40 (m, 2H), 2.34 (m, 2H), 2.03 (m, 1H), 1.93–1.81 (m, 3H), 1.64 (m, 2H), 1.51 (m, 2H), 1.03 (s, 9H), 0.21 (s, 6H). ^{13}C

NMR ($CDCl_3$) δ 166.7, 132.8, 130.5, 129.5, 128.3, 65.0, 40.9, 38.0, 28.8, 28.3, 27.8, 26.6, 25.0, 23.5, 19.8, –5.3. TLC: R_f 0.33 (hexane/EtOAc = 10:1). IR (neat): 2950, 1720, 1603, 1471, 1273, 1118, 1027, 823, 712, 478 cm^{-1}. HRMS (ESI) Calcd for $C_{22}H_{36}O_2S_2SiNa$: $[M+Na]^+$, 447.1818. Found: *m*/*z* 447.1819.

<u>Procedure for preparation of **1j**</u>

To a solution of **12j** (1.7 g, 4.1 mmol) in MeOH/H_2O (v/v = 4:1, 8.0 mL) was slowly added a solution of chloramine T trihydrate (4.6 g, 16 mmol) in MeOH/H_2O (v/v = 4:1, 32 mL) at 0 °C, and the reaction mixture was allowed to warm to ambient temperature. After being stirred for 2 h, the reaction was quenched with H_2O (0.10 L). The aqueous layers were extracted with Et_2O (0.10 L × 3), and the combined organic layers were washed with brine, dried over Na_2SO_4, and concentrated in vacuo. Purification by flash silica gel column chromatography using hexane/EtOAc (v/v = 10:1) as an eluent gave **1j**.

6-(*tert*-Butyldimethylsilyl)-6-oxohexyl benzoate (1j).

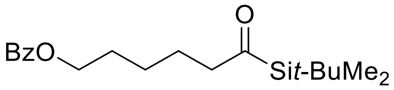

Colorless oil; 76% yield (1.0 g).

^{1}H NMR ($CDCl_3$) δ 8.04 (d, *J* = 7.0 Hz, 2H), 7.55 (t, *J* = 7.5 Hz, 1H), 7.44 (dd, *J* = 7.5, 7.0 Hz, 2H), 4.31 (t, *J* = 6.5 Hz, 2H), 2.62 (t, *J* = 7.5 Hz, 2H), 1.76 (m, 2H), 1.59 (m, 2H), 1.41 (m, 2H), 0.92 (s, 9H), 0.18 (s, 6H). ^{13}C NMR ($CDCl_3$) δ 247.4, 166.6, 132.8, 130.5, 129.5, 128.3, 64.9, 50.1, 28.7, 26.4, 25.8, 21.6, 16.5, –7.0. TLC: R_f 0.33 (hexane/EtOAc = 10:1). IR (neat): 2943, 2860, 1724, 1641, 1452, 1274, 1115, 839, 712, 476 cm^{-1}. HRMS (ESI) Calcd for $C_{19}H_{30}O_3SiNa$: $[M+Na]^+$, 357.1856. Found: *m*/*z* 357.1855.

Procedure for preparation of acylsilane 1k

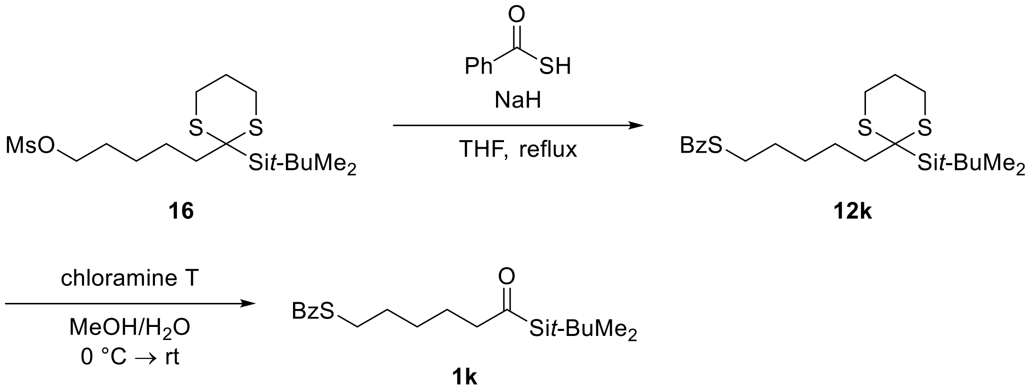

Procedure for preparation of **12k**

To a two-neck round-bottom flask was added sodium hydride (0.28 g, 55% dispersion in oil, 6.3 mmol), and it was washed with hexane (10 mL × 3). Subsequently, dry THF (3.0 mL) was added. To the resulting suspension was added thiobenzoic acid (0.70 mL, 6.0 mmol) at 0 °C. After the reaction mixture was stirred for 30 min, a solution of **16** (1.2 g, 3.0 mmol) in dry THF (5.0 mL) was added. After being refluxed overnight, the reaction was quenched with saturated aqueous $NaHCO_3$ (50 mL). The aqueous layers were extracted with EtOAc (0.10 L × 3), and the combined organic layers were washed with H_2O (0.10 L), dried over Na_2SO_4, and concentrated in vacuo. Purification by flash silica gel column chromatography using hexane/EtOAc (v/v = 10:1) as an eluent gave **12k**.

***S*-(5-(2-(*tert*-Butyldimethylsilyl)-1,3-dithian-2-yl)pentyl) benzothioate (12k)**.

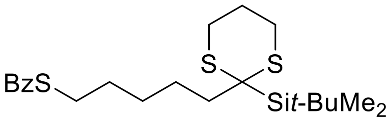

Colorless oil; 76% yield (1.0 g).
^{1}H NMR ($CDCl_3$) δ 7.98 (d, J = 7.0 Hz, 2H), 7.57 (t, J = 7.5 Hz, 1H), 7.45 (dd, J = 7.5, 7.0 Hz, 2H), 3.10 (t, J = 7.5 Hz, 2H), 3.06 (m, 2H), 2.40 (m, 2H), 2.32 (m, 2H), 2.03 (m, 1H), 1.90 (m, 1H), 1.74 (m, 2H), 1.59 (m, 2H), 1.49 (m, 2H), 1.04 (s, 9H), 0.21 (s, 6H). ^{13}C NMR ($CDCl_3$) δ 192.0, 137.2, 133.2, 128.6, 127.2, 40.9, 37.9, 29.6, 29.4, 29.0, 28.3, 27.6, 25.0, 23.5, 19.8, –5.3. TLC: R_f 0.43 (hexane/Et_2O = 10:1). IR (neat): 2928, 1667, 1582, 1459, 1255, 1209, 911, 832, 687, 476 cm^{-1}. HRMS (ESI) Calcd for $C_{22}H_{36}OS_3SiNa$: $[M+Na]^+$, 463.1590. Found: m/z 463.1589.

Procedure for preparation of **1k**

To a solution of **12k** (0.88 g, 2.0 mmol) in MeOH/H_2O (v/v = 4:1, 4.0 mL) was slowly added a solution of chloramine T trihydrate (2.3 g, 8.0 mmol) in MeOH/H_2O (v/v = 4:1, 16 mL) at 0 °C, and the reaction mixture was allowed to warm to ambient temperature. After being stirred for 2 h, the reaction was quenched with H_2O (50 mL). The aqueous layers were extracted with Et_2O (50 mL × 3), and the combined organic layers were washed with brine, dried over Na_2SO_4, and concentrated in vacuo. Purification by flash silica gel column chromatography using hexane/Et_2O (v/v = 10:1) as an eluent gave **1k**.

***S*-(6-(*tert*-Butyldimethylsilyl)-6-oxohexyl) benzothioate (1k)**.

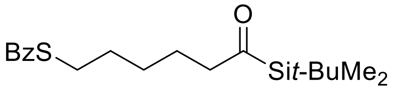

Colorless oil; 26% yield (0.18 g).

^{1}H NMR ($CDCl_3$) δ 7.96 (d, *J* = 7.0 Hz, 2H), 7.56 (t, *J* = 7.5 Hz, 1H), 7.44 (dd, *J* = 7.5, 7.0 Hz, 2H), 3.06 (t, *J* = 7.0 Hz, 2H), 2.61 (t, *J* = 7.0 Hz, 2H), 1.67 (m, 2H), 1.56 (m, 2H), 1.39 (m, 2H), 0.92 (s, 9H), 0.18 (s, 6H). ^{13}C NMR ($CDCl_3$) δ 247.5, 192.0, 137.2, 133.2, 128.6, 127.2, 50.0, 29.5, 28.8, 28.6, 26.4, 21.4, 16.5, –7.0. TLC: R_f 0.28 (hexane/Et_2O = 10:1). IR (neat): 2934, 1665, 1640, 1463, 1207, 916, 839, 776, 689, 459 cm^{-1}. HRMS (ESI) Calcd for $C_{19}H_{30}O_2SSiNa$: $[M+Na]^+$, 373.1628. Found: *m*/*z* 373.1627.

Procedure for preparation of acylsilane 1l

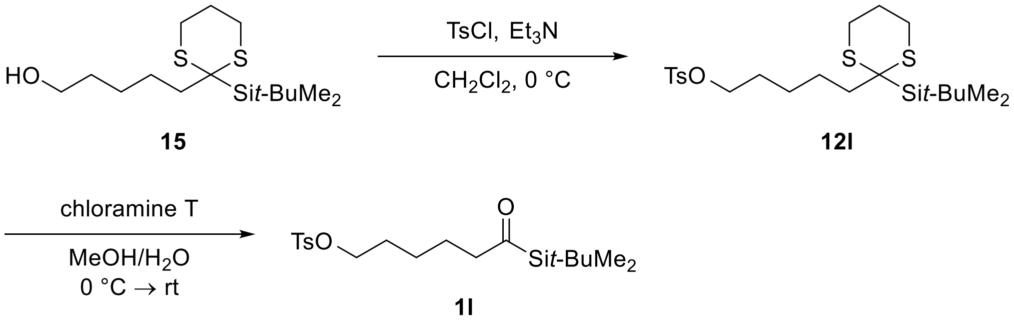

Procedure for preparation of **12l**

To a solution of **15** (6.1 g, 19 mmol) in dry CH_2Cl_2 (10 mL) were added triethylamine (2.9 mL, 21 mmol) and a solution of *p*-toluenesulfonyl chloride (1.3 mL,

17 mmol) in dry CH_2Cl_2 (30 mL) at 0 °C. After being stirred for 5 h, the reaction mixture was quenched with H_2O (50 mL). The aqueous layers were extracted with CH_2Cl_2 (0.10 L × 3), and the combined organic layers were washed with brine, dried over Na_2SO_4, and concentrated in vacuo. Purification by flash silica gel column chromatography using hexane/EtOAc (v/v = 10:1) as an eluent gave **12l**.

5-(2-(*tert*-Butyldimethylsilyl)-1,3-dithian-2-yl)pentyl 4-methylbenzenesulfonate (12l).

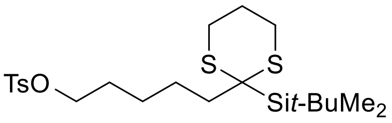

Colorless oil; 62% yield (3.6 g).
^{1}H NMR ($CDCl_3$) δ 7.80 (d, *J* = 8.0 Hz, 2H), 7.35 (d, *J* = 8.0 Hz, 2H), 4.04 (t, *J* = 6.5 Hz, 2H), 3.00 (m, 2H), 2.45 (s, 3H), 2.39 (m, 2H), 2.26 (m, 2H), 2.01 (m, 1H), 1.88 (m, 1H), 1.70 (m, 2H), 1.51 (m, 2H), 1.37 (m, 2H), 1.01 (s, 9H), 0.18 (s, 6H). ^{13}C NMR ($CDCl_3$) δ 144.7, 133.2, 129.8, 127.9, 70.6, 40.8, 37.8, 28.9, 28.3, 27.4, 25.9, 24.9, 23.5, 21.6, 19.8, –5.3. TLC: R_f 0.20 (hexane/EtOAc = 10:1). IR (neat): 2935, 1471, 1364, 1249, 1178, 1098, 957, 914, 821, 488 cm^{-1}. HRMS (ESI) Calcd for $C_{22}H_{38}O_3S_3SiNa$: $[M+Na]^+$, 497.1645. Found: *m*/*z* 497.1646.

<u>Procedure for preparation of **1l**</u>

To a solution of **12l** (2.4 g, 5.0 mmol) in MeOH/H_2O (v/v = 4:1, 10 mL) was slowly added a solution of chloramine T trihydrate (4.3 g, 15 mmol) in MeOH/H_2O (v/v = 4:1, 40 mL) at 0 °C, and the reaction mixture was allowed to warm to ambient temperature. After being stirred for 2 h, the reaction was quenched with H_2O (50 mL). The aqueous layers were extracted with Et_2O (50 mL × 3), and the combined organic layers were washed with brine, dried over Na_2SO_4, and concentrated in vacuo. Purification by flash silica gel column chromatography using $CHCl_3$ as an eluent gave **1l**.

6-(*tert*-Butyldimethylsilyl)-6-oxohexyl 4-methylbenzenesulfonate (1l).

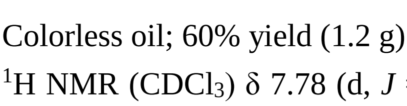

Colorless oil; 60% yield (1.2 g).
^{1}H NMR ($CDCl_3$) δ 7.78 (d, *J* = 8.0 Hz, 2H), 7.34 (d, *J* = 8.0 Hz, 2H), 4.00 (t, *J* = 6.5 Hz, 2H), 2.55 (t, *J* = 7.0 Hz, 2H), 2.45 (s, 3H), 1.63 (m, 2H), 1.45 (m, 2H), 1.27 (m, 2H),

0.91 (s, 9H), 0.16 (s, 6H). ^{13}C NMR ($CDCl_3$) δ 247.2, 144.6, 133.2, 129.8, 127.9, 70.4, 49.9, 28.8, 26.4, 25.1, 21.6, 21.2, 16.5, −7.0. TLC: R_f 0.43 ($CHCl_3$). IR (neat): 2933, 1636, 1471, 1364, 1189, 1098, 946, 831, 665, 462 cm^{-1}. HRMS (ESI) Calcd for $C_{19}H_{32}O_4SSiNa$: $[M+Na]^+$, 407.1683. Found: *m*/*z* 407.1687.

Procedure for preparation of acylsilane 1m

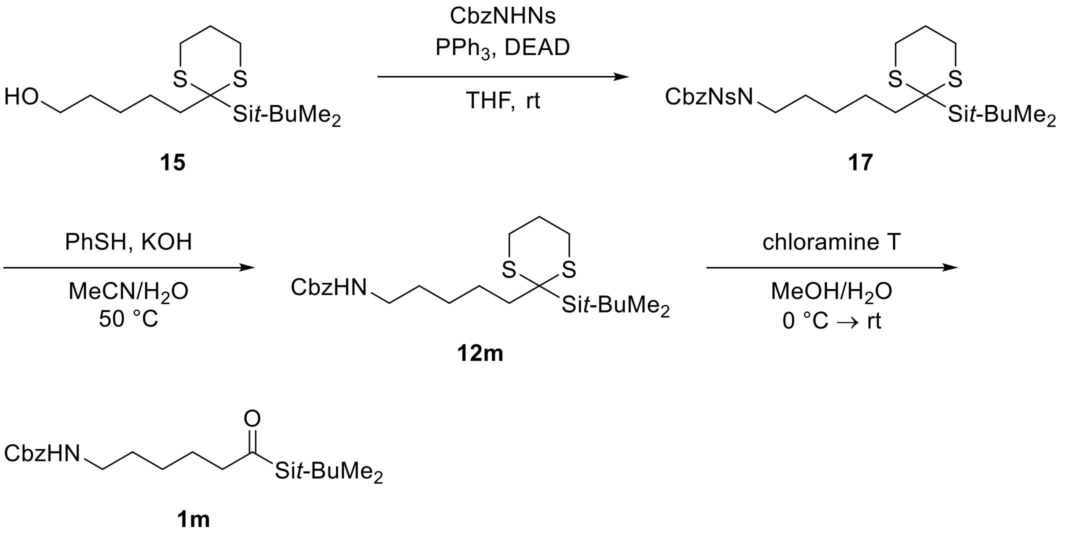

Procedure for preparation of **17**[2]

To a solution of **15** (1.6 g, 5.0 mmol) in dry THF (50 mL) were added *N*-benzyloxycarbonyl-2-nitrobenzenesulfonamide (3.4 g, 10 mmol), triphenylphosphine (2.6 g, 10 mmol), and diethyl azodicarboxylate (4.6 mL, 2.2 M in toluene, 10 mmol) at 0 °C, and the reaction mixture was allowed to warm to ambient temperature. After being stirred for 3 h, the reaction was quenched with saturated aqueous NH_4Cl (50 mL). The aqueous layers were extracted with EtOAc (50 mL × 3), and the combined organic layers were washed with brine, dried over Na_2SO_4, and concentrated in vacuo. Purification by flash silica gel column chromatography using $CHCl_3$ as an eluent gave **17**.

Benzyl (5-(2-(*tert*-butyldimethylsilyl)-1,3-dithian-2-yl)pentyl)((2-nitrophenyl)sulfonyl)carbamate (17).

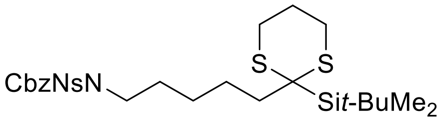

White solid; 77% yield (2.5 g).

^{1}H NMR ($CDCl_3$) δ 8.12 (d, *J* = 8.0 Hz, 1H), 7.72–7.65 (m, 2H), 7.47 (m, 1H), 7.36–7.33 (m, 3H), 7.23 (m, 2H), 6.13 (s, 2H), 3.88 (t, *J* = 7.5 Hz, 2H), 3.06 (m, 2H), 2.39 (m, 2H), 2.33 (m, 2H), 2.02 (m, 1H), 1.87 (m, 1H), 1.82 (m, 2H), 1.59 (m, 2H), 1.44 (m, 2H), 1.05 (s, 9H), 0.20 (s, 6H). ^{13}C NMR ($CDCl_3$) δ 151.8, 147.8, 134.25, 134.18, 132.9, 131.5, 128.8 (2C), 128.7, 128.5, 124.3, 69.3, 48.4, 40.9, 37.7, 30.1, 28.3, 27.6, 27.1, 25.0, 23.4, 19.8, –5.3. TLC: R_f 0.33 ($CHCl_3$). Mp. 72.5–73.0 °C. IR (KBr): 2951, 1732, 1535, 1363, 1243, 1169, 827, 738, 592, 427 cm^{-1}. HRMS (ESI) Calcd for $C_{29}H_{42}N_2O_6S_3SiNa$: $[M+Na]^+$, 661.1866. Found: *m*/*z* 661.1863.

Procedure for preparation of **12m**[3]

To a solution of **17** (2.3 g, 3.6 mmol) in MeCN (3.6 mL) were added a solution of benzenethiol (0.92 mL, 9.0 mmol) in H_2O (3.6 mL) at 0 °C. After the reaction mixture was stirred for 5 min, a solution of potassium hydroxide (0.52 g, 9.0 mmol) in MeCN (7.2 mL) was added at 0 °C. After being stirred at 50 °C for 1 h, the reaction was quenched with H_2O (50 mL). The aqueous layers were extracted with CH_2Cl_2 (50 mL × 3), and the combined organic layers were washed with brine, dried over Na_2SO_4, and concentrated in vacuo. Purification by flash silica gel column chromatography using hexane/EtOAc (v/v = 10:1) as an eluent gave **12m**.

Benzyl (5-(2-(*tert*-butyldimethylsilyl)-1,3-dithian-2-yl)pentyl)carbamate (12m).

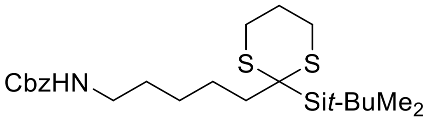

Colorless oil; 67% yield (1.1 g).

^{1}H NMR ($CDCl_3$) δ 7.37–7.36 (m, 4H), 7.32 (m, 1H), 5.10 (s, 2H), 4.73 (s, 1H), 3.21 (m, 2H), 3.03 (m, 2H), 2.40 (m, 2H), 2.30 (m, 2H), 2.03 (m, 1H), 1.88 (m, 1H), 1.57–1.53 (m, 4H), 1.37 (m, 2H), 1.03 (s, 9H), 0.20 (s, 6H). ^{13}C NMR ($CDCl_3$) δ 156.4, 136.6, 128.5, 128.1 (2C), 66.6, 41.1, 40.9, 37.9, 30.0, 28.3, 27.7, 27.3, 25.0, 23.5, 19.8, –5.3. TLC: R_f 0.20 (hexane/EtOAc = 10:1). IR (neat): 3350, 2935, 1720, 1538, 1464, 1240, 1137, 1020, 823, 477 cm^{-1}. HRMS (ESI) Calcd for $C_{23}H_{39}NO_2S_2SiNa$: $[M+Na]^+$,

476.2084. Found: *m*/*z* 476.2086.

Procedure for preparation of **1m**

To a solution of **12m** (0.91 g, 2.0 mmol) in MeOH/H_2O (v/v = 4:1, 4.0 mL) was slowly added a solution of chloramine T trihydrate (2.3 g, 8.0 mmol) in MeOH/H_2O (v/v = 4:1, 16 mL) at 0 °C, and the reaction mixture was allowed to warm to ambient temperature. After being stirred for 2 h, the reaction was quenched with H_2O (20 mL). The aqueous layers were extracted with $CHCl_3$ (20 mL × 3), and the combined organic layers were dried over Na_2SO_4 and concentrated in vacuo. Purification by flash silica gel column chromatography using $CHCl_3$ as an eluent gave **1m**.

Benzyl (6-(*tert*-butyldimethylsilyl)-6-oxohexyl)carbamate (1m).

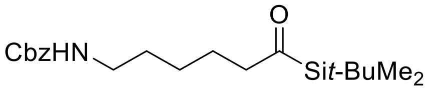

White solid; 74% yield (0.54 g).

^{1}H NMR ($CDCl_3$) δ 7.36–7.35 (m, 4H), 7.32 (m, 1H), 5.09 (s, 2H), 4.76 (s, 1H), 3.18 (m, 2H), 2.59 (t, *J* = 7.0 Hz, 2H), 1.54–1.46 (m, 4H), 1.28 (m, 2H), 0.92 (s, 9H), 0.17 (s, 6H). ^{13}C NMR ($CDCl_3$) δ 247.5, 156.4, 136.6, 128.5, 128.10, 128.07, 66.6, 50.0, 40.9, 29.9, 26.44, 26.40, 21.4, 16.5, –7.0. TLC: R_f 0.18 ($CHCl_3$). Mp. 35.5–36.0 °C. IR (KBr): 3358, 2944, 1714, 1531, 1459, 1254, 1143, 838, 778, 426 cm^{-1}. HRMS (ESI) Calcd for $C_{20}H_{33}NO_3SiNa$: $[M+Na]^+$, 386.2122. Found: *m*/*z* 386.2127.

Procedure for preparation of acylsilane 1n

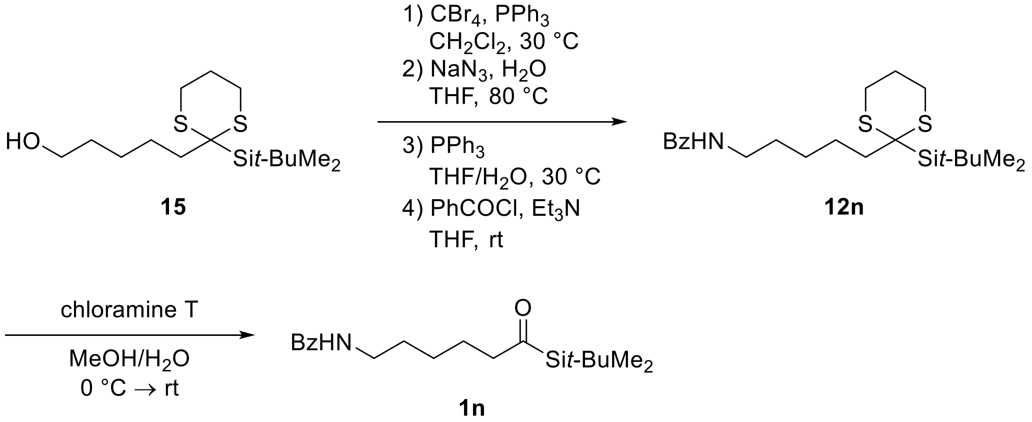

Procedure for preparation of **12n**

To a solution of **15** (10 g, 33 mmol) and carbon tetrabromide (13 g, 40 mmol) in dry

CH_2Cl_2 (35 mL) was slowly added a solution of triphenylphosphine (13 g, 50 mmol) in dry CH_2Cl_2 (35 mL) at 0 °C. After being stirred at 30 °C for 1 h, hexane (50 mL) and CH_2Cl_2 (5.0 mL) was added, and precipitates were filtrated. The filtrate was concentrated in vacuo, and the crude product was used for the next step without further purification.

To a solution of the crude product in THF (80 mL) was slowly added a solution of sodium azide (4.3 g, 66 mmol) in H_2O (10 mL). After being refluxed for 24 h, the reaction was quenched with H_2O (0.10 L). The aqueous layers were extracted with Et_2O (0.10 L × 3), and the combined organic layers were washed with H_2O and brine, dried over Na_2SO_4, and concentrated in vacuo. The crude product was used for the next step without further purification.

To a solution of the crude product in THF/H_2O (v/v = 10:1, 70 mL) was slowly added triphenylphosphine (11 g, 33 mmol). After being stirred at 30 °C for 5 h, the reaction was quenched with H_2O (0.10 L). The aqueous layers were extracted with Et_2O (50 mL × 3), and the combined organic layers were washed with brine, dried over Na_2SO_4, and concentrated in vacuo. To the crude product was added hexane (50 mL) and CH_2Cl_2 (5.0 mL), and precipitates were filtrated. The filtrate was concentrated in vacuo, and the crude product was used for the next step without further purification.

To a solution of the crude product in CH_2Cl_2 (70 mL) was slowly added triethylamine (9.7 mL, 70 mmol) and benzoyl chloride (3.9 mL, 40 mmol). After being stirred at ambient temperature for 24 h, the reaction was quenched with saturated aqueous $NaHCO_3$ (0.20 L). The aqueous layers were extracted with CH_2Cl_2 (0.10 L × 3), and the combined organic layers were washed with H_2O and brine, dried over Na_2SO_4, and concentrated in vacuo. Purification by flash silica gel column chromatography using hexane/EtOAc (v/v = 3:1) as an eluent gave **12n**.

***N*-(5-(2-(*tert*-Butyldimethylsilyl)-1,3-dithian-2-yl)pentyl)benzamide (12n)**.

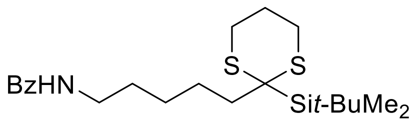

Colorless oil; 29% yield (for 4 steps, 4.1 g).

^{1}H NMR ($CDCl_3$) δ 7.76 (d, *J* = 7.0 Hz, 2H), 7.50 (t, *J* = 7.0 Hz, 1H), 7.44 (dd, *J* = 7.0, 7.0 Hz, 2H), 6.12 (s, 1H), 3.48 (m, 2H), 3.04 (m, 2H), 2.41 (m, 2H), 2.33 (m, 2H), 2.02 (m, 1H), 1.89 (m, 1H), 1.68 (m, 2H), 1.60 (m, 2H), 1.45 (m, 2H), 1.03 (s, 9H), 0.20 (s, 6H). ^{13}C NMR ($CDCl_3$) δ 167.5, 134.8, 131.4, 128.6, 126.8, 40.9, 40.1, 37.9, 29.8, 28.3, 27.8, 27.6, 25.0, 23.5, 19.8, –5.3. TLC: R_f 0.25 (hexane/EtOAc = 3:1). IR

(neat): 3329, 2936, 1638, 1542, 1465, 1310, 1257, 824, 702, 481 cm^{-1}. HRMS (ESI) Calcd for $C_{22}H_{37}NOS_2SiNa$: $[M+Na]^+$, 446.1978. Found: *m*/*z* 446.1979.

Procedure for preparation of **1n**

To a solution of **12n** (4.1 g, 9.7 mmol) in MeOH/H_2O (v/v = 4:1, 0.10 L) was slowly added a solution of chloramine T trihydrate (11 g, 39 mmol) in MeOH/H_2O (v/v = 4:1, 0.15 L) at 0 °C, and the reaction mixture was allowed to warm to ambient temperature. After being stirred for 2 h, the reaction was quenched with H_2O (0.20 L). The aqueous layers were extracted with Et_2O (0.20 L × 3), and the combined organic layers were washed with H_2O and brine, dried over Na_2SO_4, and concentrated in vacuo. Purification by flash silica gel column chromatography using hexane/EtOAc (v/v = 2:1) as an eluent gave **1n**.

***N*-(6-(*tert*-Butyldimethylsilyl)-6-oxohexyl)benzamide (1n)**.

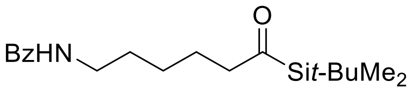

White solid; 63% yield (2.0 g).

^{1}H NMR ($CDCl_3$) δ 7.79 (d, *J* = 7.5 Hz, 2H), 7.49 (t, *J* = 7.5 Hz, 1H), 7.43 (dd, *J* = 7.5, 7.5 Hz, 2H), 6.31 (s, 1H), 3.48 (m, 2H), 2.63 (t, *J* = 7.0 Hz, 2H), 1.64–1.53 (m, 4H), 1.35 (m, 2H), 0.92 (s, 9H), 0.18 (s, 6H). ^{13}C NMR ($CDCl_3$) δ 247.8, 167.5, 134.8, 131.3, 128.5, 126.9, 50.0, 39.5, 29.4, 26.5, 26.4, 21.1, 16.5, –7.0. Mp. 65.5–66.0 °C. TLC: R_f 0.25 (hexane/EtOAc = 2:1). IR (KBr): 3313, 2935, 1634, 1550, 1306, 1248, 1159, 826, 695, 422 cm^{-1}. HRMS (ESI) Calcd for $C_{19}H_{31}NO_2SiNa$: $[M+Na]^+$, 356.2016. Found: *m*/*z* 356.2014.

Procedure for preparation of acylsilane 1o

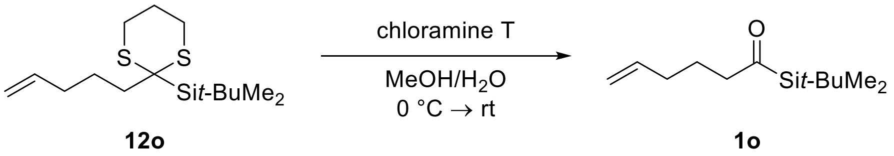

Procedure for preparation of **1o**

To a solution of **12o** (15 g, 50 mmol) in MeOH/H_2O (v/v = 4:1, 0.10 L) was slowly added a solution of chloramine T trihydrate (56 g, 0.20 mol) in MeOH/H_2O (v/v = 4:1, 0.40 L) at 0 °C, and the reaction mixture was allowed to warm to ambient temperature.

After being stirred for 2 h, the reaction was quenched with H_2O (0.20 L). The aqueous layers were extracted with $CHCl_3$ (0.20 L × 3), and the combined organic layers were dried over Na_2SO_4 and concentrated in vacuo. Purification by flash silica gel column chromatography using hexane/EtOAc (v/v = 30:1) as an eluent gave **1o**.

1-(*tert*-Butyldimethylsilyl)hex-5-en-1-one (1o): CAS RN [144668-16-6].

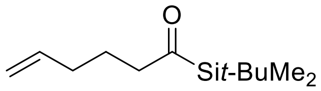

Colorless oil; 31% yield (3.3 g).
^{1}H NMR ($CDCl_3$) δ 5.75 (ddt, J = 17.0, 10.0, 7.0 Hz, 1H), 4.98 (ddt, J = 17.0, 2.0, 1.5 Hz, 1H), 4.95 (ddt, J = 10.0, 2.0, 1.0 Hz, 1H), 2.60 (t, J = 7.0 Hz, 2H), 2.02 (dddt, J = 7.5, 1.5, 1.0, 7.0 Hz, 2H), 1.62 (tt, J = 7.0, 7.0 Hz, 2H), 0.92 (s, 9H), 0.17 (s, 6H). ^{13}C NMR ($CDCl_3$) δ 247.5, 138.3, 115.0, 49.3, 33.2, 26.4, 21.0, 16.5, –7.0. TLC: R_f 0.25 (hexane/EtOAc = 30:1).

Procedure for preparation of acylsilane 1p

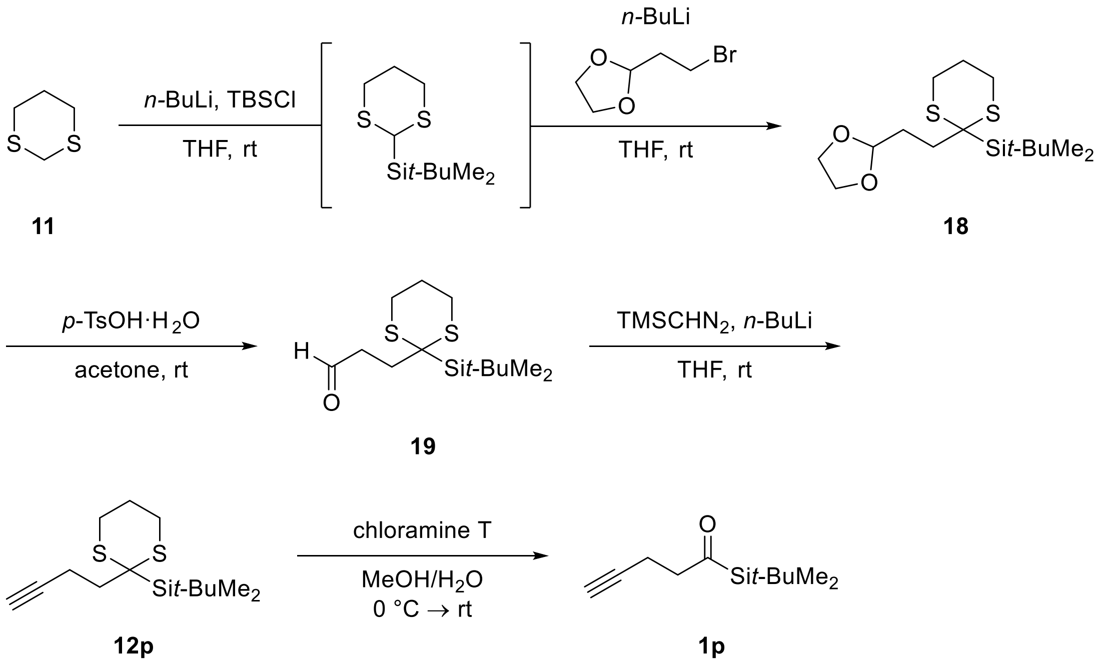

<u>Procedure for preparation of **18**</u>

To a solution of **11** (3.6 g, 30 mmol) in dry THF (90 mL) was slowly added *n*-butyllithium (21 mL, 1.60 M in hexane, 33 mmol) at –30 °C. After the mixture was stirred for 30 min, a solution of *tert*-butylchlorodimethylsilane (5.0 g, 33 mmol) in dry

THF (30 mL) was added, and the reaction mixture was allowed to warm to ambient temperature. After being stirred for 18 h, the reaction mixture was cooled to –30 °C, and then *n*-butyllithium (21 mL, 1.60 M in hexane, 33 mmol) was slowly added. After the mixture was stirred for 30 min, 2-(2-bromoethyl)-1,3-dioxolane (4.3 mL, 36 mmol) was added at –30 °C, and the reaction mixture was allowed to warm to ambient temperature. After being stirred for 5 h, the reaction was quenched with H_2O (0.10 L). The aqueous layers were extracted with Et_2O (0.10 L × 3), and the combined organic layers were washed with brine, dried over Na_2SO_4, and concentrated in vacuo. Purification by flash silica gel column chromatography using hexane/EtOAc (v/v = 10:1) as an eluent gave **18**.

(2-(2-(1,3-Dioxolan-2-yl)ethyl)-1,3-dithian-2-yl)(*tert*-butyl)dimethylsilane (18).

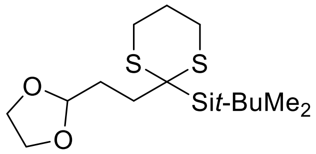

Colorless oil; 92% yield (9.2 g).

^{1}H NMR ($CDCl_3$) δ 4.93 (t, J = 4.0 Hz, 1H), 3.94 (m, 4H), 3.09 (ddd, J = 14.5, 13.0, 3.0 Hz, 2H), 2.45 (m, 2H), 2.35 (ddd, J = 14.5, 4.5, 3.0 Hz, 2H), 2.05–1.85 (m, 4H), 1.05 (s, 9H), 0.21 (s, 6H). ^{13}C NMR ($CDCl_3$) δ 104.5, 64.9, 40.6, 32.3, 31.2, 28.3, 25.0, 23.2, 19.8, –5.4. TLC: R_f 0.25 (Hexane/EtOAc = 10:1). IR (neat): 2934, 1474, 1418, 1251, 1141, 1018, 911, 824, 735, 491 cm^{-1}. HRMS (ESI) Calcd for $C_{15}H_{31}O_2S_2Si$: [M+H]$^+$, 335.1529. Found: *m*/*z* 335.1528.

<u>Procedure for preparation of **19**</u>

To a solution of **18** (9.2 g, 27 mmol) in acetone/H_2O (v/v = 1:1, 0.40 L) were added *p*-toluenesulfonic acid monohydrate (0.51 g, 2.7 mmol). After being stirred at 70 °C overnight, the reaction was quenched with saturated aqueous $NaHCO_3$ (0.10 L). The aqueous layers were extracted with Et_2O (0.10 L × 3), and the combined organic layers were washed with brine, dried over Na_2SO_4, and concentrated in vacuo. Purification by flash silica gel column chromatography using hexane/EtOAc (v/v = 10:1) as an eluent gave **19**.

3-(2-(*tert*-Butyldimethylsilyl)-1,3-dithian-2-yl)propanal (19): CAS RN [617693-14-8].

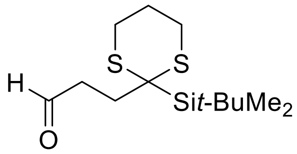

Colorless oil; 82% yield (6.4 g).

^{1}H NMR ($CDCl_3$) δ 9.87 (t, J = 1.0 Hz, 1H), 3.01 (ddd, J = 15.0, 12.5, 3.0 Hz, 2H), 2.75–2.68 (m, 4H), 2.40 (ddd, J = 15.0, 4.5, 3.0 Hz, 2H), 1.96 (m, 2H), 1.04 (s, 9H), 0.23 (s, 6H). ^{13}C NMR ($CDCl_3$) δ 201.4, 42.7, 40.0, 30.0, 28.2, 24.6, 23.4, 19.8, –5.3. TLC: R_f 0.38 (Hexane/EtOAc = 10:1).

Procedure for preparation of **12p**[4]

To a solution of trimethylsilyldiazomethane (10 mL, 0.60 M in hexane, 6.0 mmol) in dry THF (15 mL) was slowly added *n*-butyllithium (3.9 mL, 1.60 M in hexane, 6.0 mmol) at –78 °C. After the mixture was stirred for 1 h, a solution of **19** (1.5 g, 5.0 mmol) in dry THF (5.0 mL) was added. After being stirred at –78 °C for 1 h, the reaction mixture was allowed to warm to 0 °C. After being stirred for 30 min, the reaction mixture was allowed to warm to ambient temperature. After being stirred for 1 h, the reaction was quenched with saturated aqueous NH_4Cl (20 mL). The aqueous layers were extracted with Et_2O (30 mL × 3), and the combined organic layers were washed with brine, dried over Na_2SO_4, and concentrated in vacuo. Purification by flash silica gel column chromatography using hexane/EtOAc (v/v = 20:1) as an eluent gave **12p**.

(2-(But-3-yn-1-yl)-1,3-dithian-2-yl)(*tert*-butyl)dimethylsilane (12p).

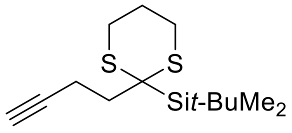

Colorless oil; 80% yield (1.2 g).

^{1}H NMR ($CDCl_3$) δ 3.05 (ddd, J = 15.0, 13.0, 3.0 Hz, 2H), 2.62 (m, 2H), 2.46–2.59 (m, 4H), 2.01 (t, J = 2.5 Hz, 1H), 1.95 (m, 2H), 1.05 (s, 9H), 0.20 (s, 6H). ^{13}C NMR ($CDCl_3$) δ 84.2, 68.6, 40.1, 36.8, 28.2, 24.8, 23.4, 19.7, 17.5, –5.4. TLC: R_f 0.50 (Hexane/EtOAc = 20:1). IR (neat): 3310, 2936, 1472, 1424, 1365, 1251, 1010, 824, 623, 492 cm^{-1}. HRMS (ESI) Calcd for $C_{14}H_{27}S_2Si$: $[M+H]^+$, 287.1318. Found: m/z 287.1315.

Procedure for preparation of **1p**

To a solution of **12p** (0.72 g, 2.5 mmol) in MeOH/H_2O (v/v = 4:1, 5.0 mL) was slowly added a solution of chloramine T trihydrate (2.8 g, 10 mmol) in MeOH/H_2O (v/v = 4:1, 20 mL) at 0 °C, and the reaction mixture was allowed to warm to ambient temperature. After being stirred for 2 h, the reaction was quenched with H_2O (0.10 L). The aqueous layers were extracted with $CHCl_3$ (200 mL × 3), and the combined organic layers were dried over Na_2SO_4 and concentrated in vacuo. Purification by flash silica gel column chromatography using hexane/EtOAc (v/v = 20:1) as an eluent gave **1p**.

1-(*tert*-Butyldimethylsilyl)pent-4-yn-1-one (1p).

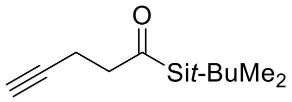

Colorless oil; 53% yield (0.26 g).
^{1}H NMR ($CDCl_3$) δ 2.85 (t, J = 7.0 Hz, 2H), 2.39 (dt, J = 7.0, 3.5 Hz, 2H), 1.92 (t, J = 3.5 Hz, 1H), 0.93 (s, 9H), 0.20 (s, 6H). ^{13}C NMR ($CDCl_3$) δ 244.3, 83.8, 68.3, 48.7, 26.4, 16.6, 11.1, –7.1. TLC: R_f 0.25 (Hexane/EtOAc = 20:1). IR (neat): 3313, 2943, 1643, 1465, 1365, 1251, 1008, 838, 634, 487 cm^{-1}. HRMS (ESI) Calcd for $C_{11}H_{20}OSiNa$: [M+Na]$^+$, 219.1176. Found: *m*/*z* 219.1174.

Procedure for preparation of 21q[5]

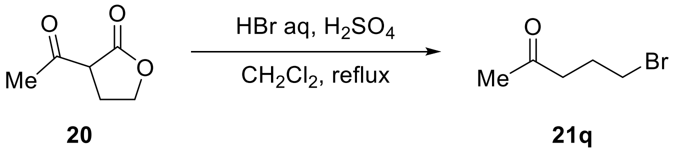

To a solution of **20** (3.2 mL, 30 mmol) in dry CH_2Cl_2 (30 mL) were added hydrobromic acid (34 mL, 48% in H_2O, 0.30 mol) and sulfuric acid (0.16 mL, 3.0 mmol) at 0 °C. The reaction mixture was refluxed for 3 h. After being cooled to ambient temperature, the reaction was quenched with H_2O (0.30 L). The aqueous layers were extracted with CH_2Cl_2 (200 mL × 3), and the combined organic layers were washed with brine, dried over Na_2SO_4, and concentrated in vacuo. Purification by flash silica gel column chromatography using hexane/EtOAc (v/v = 10:1) as an eluent gave **21q**.

5-Bromopentan-2-one (21q): CAS RN [3884-71-7].

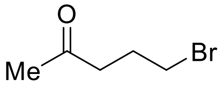

Colorless oil; 70% yield (3.5 g).

^{1}H NMR ($CDCl_3$) δ 3.45 (t, J = 6.5 Hz, 2H), 2.64 (t, J = 6.5 Hz, 2H), 2.17 (s, 3H), 2.12 (tt, J = 6.5, 6.5 Hz, 2H). ^{13}C NMR ($CDCl_3$) δ 207.4, 41.4, 33.3, 30.1, 26.3. TLC: R_f 0.25 (hexane/EtOAc = 10:1).

Procedure for preparation of 21r[6]

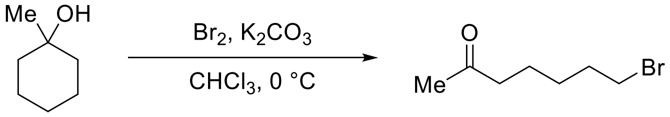

22 **21r**

To a solution of 1-methylcyclohexan-1-ol (**22**, 3.4 g, 30 mmol) in $CHCl_3$ (90 mL) were added potassium carbonate (21 g, 0.15 mol). After the reaction mixture was cooled to 0 °C, bromine (9.3 mL, 0.18 mol) was slowly added. After being stirred for 5 h, the reaction was quenched with saturated aqueous $Na_2S_2O_3$ (200 mL). The aqueous layers were extracted with Et_2O (0.30 L × 3), and the combined organic layers were washed with H_2O and brine, dried over Na_2SO_4, and concentrated in vacuo. Purification by flash silica gel column chromatography using hexane/EtOAc (v/v = 10:1) as an eluent gave **21r**.

7-Bromoheptan-2-one (21r): CAS RN [50775-02-5].

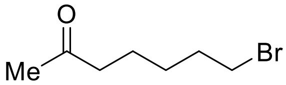

Colorless oil; 72% yield (4.1 g).

^{1}H NMR ($CDCl_3$) δ 3.41 (t, J = 7.0 Hz, 2H), 2.45 (t, J = 7.0 Hz, 2H), 2.14 (s, 3H), 1.87 (m, 2H), 1.60 (m, 2H), 1.44 (m, 2H). ^{13}C NMR ($CDCl_3$) δ 208.7, 43.4, 33.5, 32.5, 29.9, 27.6, 22.8. TLC: R_f 0.35 (hexane/EtOAc = 10:1).

General procedure for preparation of 23q and 23r

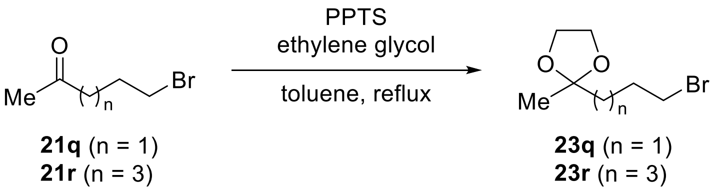

To a solution of **21** (1.0 equiv) in toluene (0.20 M) were added pyridinium *p*-toluenesulfonate (0.30 equiv) and ethylene glycol (4.0 equiv). The reaction mixture was refluxed for 2 h to remove H_2O. After being cooled to ambient temperature, the reaction was quenched with saturated aqueous $NaHCO_3$ (0.20 L). The aqueous layers were extracted with Et_2O (0.10 L × 3), and the combined organic layers were washed with brine, dried over Na_2SO_4, and concentrated in vacuo. Purification by flash silica gel column chromatography using hexane/EtOAc (v/v = 10:1) as an eluent gave **23**.

2-(3-Bromopropyl)-2-methyl-1,3-dioxolane (23q): CAS RN [24400-75-7].

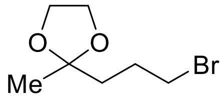

Colorless oil; 66% yield (2.9 g).
^{1}H NMR ($CDCl_3$) δ 3.94 (m, 4H), 3.44 (t, J = 6.5 Hz, 2H), 1.98 (m, 2H), 1.79 (m, 2H), 1.33 (s, 3H). ^{13}C NMR ($CDCl_3$) δ 109.5, 64.7, 37.6, 34.0, 27.5, 24.0. TLC: R_f 0.38 (hexane/EtOAc = 10:1).

2-(5-Bromopentyl)-2-methyl-1,3-dioxolane (23r): CAS RN [37865-98-8].

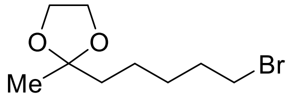

Colorless oil; 79% yield (3.9 g).
^{1}H NMR ($CDCl_3$) δ 3.93 (m, 4H), 3.41 (t, J = 6.5 Hz, 2H), 1.87 (m, 2H), 1.65 (m, 2H), 1.46–1.41 (m, 4H), 1.31 (s, 3H). ^{13}C NMR ($CDCl_3$) δ 110.0, 64.6, 39.0, 33.8, 32.8, 28.3, 23.7, 23.2. TLC: R_f 0.33 (hexane/EtOAc = 10:1).

General procedure for preparation of acylsilanes 1q and 1r

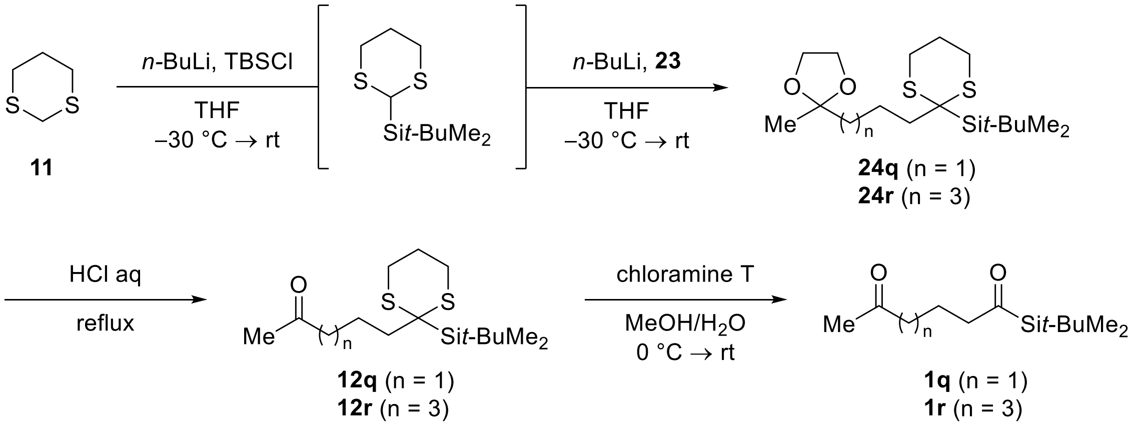

General procedure for preparation of **24q** and **24r**

To a solution of **11** (1.0 equiv) in dry THF (0.25 M) was slowly added *n*-butyllithium (1.1 equiv, 1.60 M in hexane) at –30 °C. After the mixture was stirred for 30 min, a solution of *tert*-butylchlorodimethylsilane (1.1 equiv) in dry THF (1.0 M) was added, and the reaction mixture was allowed to warm to ambient temperature. After being stirred overnight, the reaction mixture was cooled to –30 °C, and then *n*-butyllithium (1.1 equiv, 1.60 M in hexane) was slowly added. After the mixture was stirred for 30 min, a solution of **23** (1.2 equiv) in dry THF (1.0 M) was added at –30 °C, and the reaction mixture was allowed to warm to ambient temperature. After being stirred for 5 h, the reaction was quenched with H_2O (0.10 L). The aqueous layers were extracted with Et_2O (0.10 L × 3), and the combined organic layers were washed with brine, dried over Na_2SO_4, and concentrated in vacuo. Purification by flash silica gel column chromatography using hexane/EtOAc (v/v = 10:1) as an eluent gave **24**.

***tert*-Butyldimethyl(2-(3-(2-methyl-1,3-dioxolan-2-yl)propyl)-1,3-dithian-2-yl)silane (24q)**.

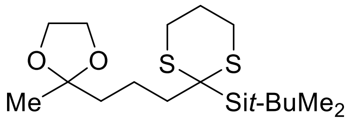

Colorless oil; 96% yield (3.5 g).

^{1}H NMR ($CDCl_3$) δ 3.94 (m, 4H), 3.05 (m, 2H), 2.40 (m, 2H), 2.32 (m, 2H), 1.99 (m, 2H), 1.68 (m, 4H), 1.34 (s, 3H), 1.04 (s, 9H), 0.22 (s, 6H). ^{13}C NMR ($CDCl_3$) δ 110.0, 64.7, 41.0, 39.4, 38.2, 28.3, 25.0, 24.0, 23.6, 22.7, 19.8, –5.3. TLC: R_f 0.38 (hexane/EtOAc = 10:1). IR (neat): 2921, 1471, 1376, 1251, 1118, 1067, 911, 822, 688, 502 cm^{-1}. HRMS (ESI) Calcd for $C_{17}H_{34}O_2S_2SiNa$: $[M+Na]^+$, 385.1662. Found: *m*/*z*

385.1671.

***tert*-Butyldimethyl(2-(5-(2-methyl-1,3-dioxolan-2-yl)pentyl)-1,3-dithian-2-yl)silane (24r)**.

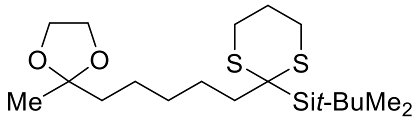

Colorless oil; 79% yield (3.1 g).

^{1}H NMR ($CDCl_3$) δ 3.95 (m, 4H), 3.05 (m, 2H), 2.39 (m, 2H), 2.31 (m, 2H), 2.02 (m, 1H), 1.89 (m, 1H), 1.65 (m, 2H), 1.57 (m, 2H), 1.45 (m, 2H), 1.35 (m, 2H), 1.32 (s, 3H), 1.03 (s, 9H), 0.20 (s, 6H). ^{13}C NMR ($CDCl_3$) δ 110.1, 64.6, 41.0, 39.3, 37.9, 30.4, 28.3, 28.0, 25.0, 24.1, 23.7, 23.5, 19.8, –5.3. TLC: R_f 0.25 (hexane/EtOAc = 10:1). IR (neat): 2943, 1471, 1376, 1249, 1136, 1077, 824, 763, 672, 503 cm^{-1}. HRMS (ESI) Calcd for $C_{19}H_{38}O_2S_2SiNa$: [M+Na]$^+$, 413.1975. Found: *m*/*z* 413.1977.

General procedure for preparation of **12q** and **12r**

The mixture of **24** (1.0 equiv) and aqueous HCl (4.0 equiv, 1.0 M in H_2O) was refluxed for 1 h. After being cooled to ambient temperature, the reaction was quenched with saturated aqueous $NaHCO_3$ (0.10 L). The aqueous layers were extracted with Et_2O (0.10 L × 3), and the combined organic layers were washed with brine, dried over Na_2SO_4, and concentrated in vacuo. Purification by flash silica gel column chromatography using hexane/EtOAc (v/v = 5:1–20:1) as an eluent gave **12**.

5-(2-(*tert*-Butyldimethylsilyl)-1,3-dithian-2-yl)pentan-2-one (12q).

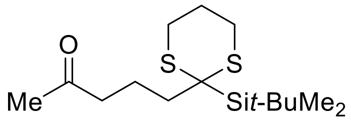

Colorless oil; 39% yield (0.63 g).

^{1}H NMR ($CDCl_3$) δ 3.07 (m, 2H), 2.49 (t, J = 7.0 Hz, 2H), 2.41 (ddd, J = 14.5, 4.5, 3.5 Hz, 2H), 2.30 (m, 2H), 2.16 (s, 3H), 2.03–1.93 (m, 4H), 1.04 (s, 9H), 0.22 (s, 6H). ^{13}C NMR ($CDCl_3$) δ 208.5, 43.7, 40.7, 37.4, 30.0, 28.3, 24.9, 23.5, 22.3, 19.8, –5.3. TLC: R_f 0.13 (hexane/EtOAc = 20:1). IR (neat): 2930, 1716, 1471, 1418, 1363, 1250, 1159, 822, 689, 460 cm^{-1}. HRMS (ESI) Calcd for $C_{15}H_{30}OS_2SiNa$: [M+Na]$^+$, 341.1400. Found: *m*/*z* 341.1407.

7-(2-(*tert*-Butyldimethylsilyl)-1,3-dithian-2-yl)heptan-2-one (12r).

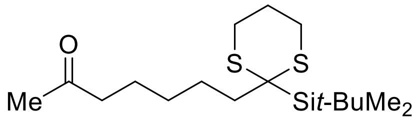

Colorless oil; 83% yield (2.0 g).

^{1}H NMR ($CDCl_3$) δ 3.03 (m, 2H), 2.44 (t, *J* = 7.0 Hz, 2H), 2.39 (m, 2H), 2.30 (m, 2H), 2.14 (s, 3H), 2.01 (m, 1H), 1.90 (m, 1H), 1.63 (m, 2H), 1.54 (m, 2H), 1.34 (m, 2H), 1.03 (s, 9H), 0.20 (s, 6H). ^{13}C NMR ($CDCl_3$) δ 209.1, 43.7, 40.9, 37.9, 29.9, 29.7, 28.3, 27.9, 25.0, 23.8, 23.5, 19.8, –5.3. TLC: R_f 0.33 (hexane/EtOAc = 5:1). IR (neat): 2948, 1720, 1474, 1418, 1360, 1249, 1167, 1013, 823, 479 cm^{-1}. HRMS (ESI) Calcd for $C_{17}H_{34}OS_2SiNa$: [M+Na]$^+$, 369.1713. Found: *m*/*z* 369.1710.

General procedure for preparation of **1q** and **1r**

To a solution of **12** (1.0 equiv) in MeOH/H_2O (v/v = 4:1, 0.50 M) was slowly added a solution of chloramine T trihydrate (8.0 equiv) in MeOH/H_2O (v/v = 4:1, 0.5 M) at 0 °C, and the reaction mixture was allowed to warm to ambient temperature. After being stirred for 2 h, the reaction was quenched with H_2O (50 mL). The aqueous layers were extracted with $CHCl_3$ (50 mL × 3), and the combined organic layers were dried over Na_2SO_4 and concentrated in vacuo. Purification by flash silica gel column chromatography using $CHCl_3$/MeOH (v/v = 50:1, for **1q**) and hexane/EtOAc (v/v = 2:1, for **1r**), respectively, as an eluent gave **1**.

1-(*tert*-Butyldimethylsilyl)hexane-1,5-dione (1q).

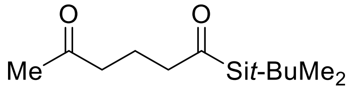

Colorless oil; 61% yield (1.5 g).

^{1}H NMR ($CDCl_3$) δ 2.63 (t, *J* = 7.0 Hz, 2H), 2.42 (t, *J* = 7.5 Hz, 2H), 2.12 (s, 3H), 1.78 (tt, *J* = 7.5, 7.0 Hz, 2H), 0.92 (s, 9H), 0.17 (s, 6H). ^{13}C NMR ($CDCl_3$) δ 247.0, 208.6, 49.0, 42.7, 29.8, 26.4, 16.5, 16.1, –7.0. TLC: R_f 0.30 ($CHCl_3$/MeOH = 50:1). IR (neat): 2935, 1719, 1641, 1465, 1365, 1259, 1176, 1008, 835, 470 cm^{-1}. HRMS (ESI) Calcd for $C_{12}H_{24}O_2SiNa$: [M+Na]$^+$, 251.1438. Found: *m*/*z* 251.1434.

1-(*tert*-Butyldimethylsilyl)octane-1,7-dione (1r).

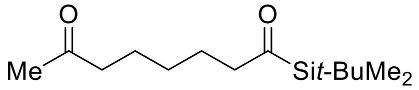

Colorless oil; 52% yield (0.73 g).

^{1}H NMR ($CDCl_3$) δ 2.59 (t, *J* = 7.0 Hz, 2H), 2.42 (t, *J* = 7.0 Hz, 2H), 2.12 (s, 3H), 1.59–1.48 (m, 4H), 1.24 (m, 2H), 0.92 (s, 9H), 0.17 (s, 6H). ^{13}C NMR ($CDCl_3$) δ 247.6, 209.1, 50.0, 43.5, 29.9, 28.8, 26.4, 23.6, 21.5, 16.5, –7.0. TLC: R_f 0.38 (hexane/EtOAc = 2:1). IR (neat): 2932, 1720, 1641, 1471, 1364, 1250, 1162, 1007, 839, 459 cm^{-1}. HRMS (ESI) Calcd for $C_{14}H_{28}O_2SiNa$: $[M+Na]^+$, 279.1751. Found: *m*/*z* 279.1750.

Procedure for preparation of acylsilane 1s

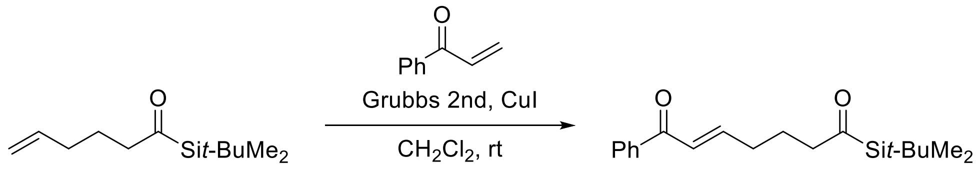

1o **1s**

To a solution of **1o** (3.2 g, 15 mmol) and 1-phenylprop-2-en-1-one (7.9 g, 60 mmol) in dry CH_2Cl_2 (0.60 L) were added Grubbs 2nd generation catalyst (0.64 g, 0.75 mmol) and copper(I) iodide (0.17 g, 0.90 mmol). After being stirred at ambient temperature overnight, the reaction mixture was concentrated in vacuo. Purification by flash silica gel column chromatography using hexane/EtOAc (v/v = 20:1) as an eluent gave **1s**.

(*E*)-7-(*tert*-Butyldimethylsilyl)-1-phenylhept-2-ene-1,7-dione (1s): CAS RN [2254446-63-2].

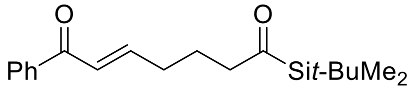

Colorless oil; 27% yield (1.2 g).

^{1}H NMR ($CDCl_3$) δ 7.92 (m, 2H), 7.55 (m, 1H), 7.46 (m, 2H), 7.01 (dt, *J* = 15.5, 7.0 Hz, 1H), 6.88 (dt, *J* = 15.5, 1.0 Hz, 1H), 2.67 (t, *J* = 7.0 Hz, 2H), 2.29 (ddt, *J* = 7.0, 1.0, 7.0 Hz, 2H), 1.77 (tt, *J* = 7.0, 7.0 Hz, 2H), 0.93 (s, 9H), 0.18 (s, 6H). ^{13}C NMR ($CDCl_3$) δ 246.8, 190.8, 149.0, 137.9, 132.6, 128.5 (2C), 126.3, 49.2, 32.1, 26.4, 20.3, 16.5, –7.0. TLC: R_f 0.13 (hexane/EtOAc = 20:1).

Procedure for preparation of acylsilane 1t

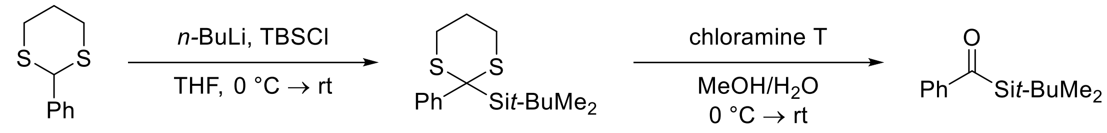

25 **12t** **1t**

<u>Procedure for preparation of **12t**</u>

To a solution of **25** (5.0 g, 26 mmol) in dry THF (40 mL) was slowly added *n*-butyllithium (19 mL, 1.60 M in hexane, 29 mmol) at –30 °C. After the mixture was stirred for 30 min, a solution of *tert*-butylchlorodimethylsilane (4.5 g, 30 mmol) in dry THF (10 mL) was added, and the reaction mixture was allowed to warm to ambient temperature. After being stirred for 24 h, the reaction was quenched with H_2O (0.10 L). The aqueous layers were extracted with Et_2O (0.10 L × 3), and the combined organic layers were washed with brine, dried over Na_2SO_4, and concentrated in vacuo. Purification by flash silica gel column chromatography using hexane/EtOAc (v/v = 20:1) as an eluent gave **12t**.

***tert*-Butyldimethyl(2-phenyl-1,3-dithian-2-yl)silane (12t)**: CAS RN [461051-83-2].

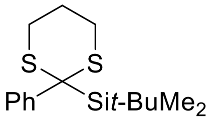

White solid; 99% yield (8.1 g).

^{1}H NMR ($CDCl_3$) δ 7.97 (d, *J* = 7.5 Hz, 2H), 7.37 (dd, *J* = 7.5, 7.5 Hz, 2H), 7.18 (t, *J* = 7.5 Hz, 1H), 2.78 (m, 2H), 2.39 (m, 2H), 2.00 (m, 1H), 1.86 (m, 1H), 0.80 (s, 9H), 0.15 (s, 6H). ^{13}C NMR ($CDCl_3$) δ 140.7, 130.2, 128.3, 125.3, 48.7, 27.8, 25.21, 25.16, 19.7, −7.0. TLC: R_f 0.60 (hexane/EtOAc = 20:1).

<u>Procedure for preparation of **1t**</u>

To a solution of **12t** (8.1 g, 26 mmol) in MeOH/H_2O (v/v = 4:1, 50 mL) was slowly added a solution of chloramine T trihydrate (28 g, 100 mmol) in MeOH/H_2O (v/v = 4:1, 0.20 L) at 0 °C, and the reaction mixture was allowed to warm to ambient temperature. After being stirred for 2 h, the reaction was quenched with H_2O (0.20 L). The aqueous layers were extracted with Et_2O (0.20 L × 3), and the combined organic layers were washed with brine, dried over Na_2SO_4, and concentrated in vacuo. Purification by flash silica gel column chromatography using hexane/EtOAc (v/v = 20:1) as an eluent gave **1t**.

(*tert*-Butyldimethylsilyl)(phenyl)methanone (1t): CAS RN [132868-67-8].

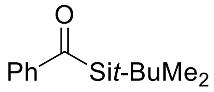

Yellow oil; 86% yield (4.8 g).
^{1}H NMR ($CDCl_3$) δ 7.80 (m, 2H), 7.52 (m, 1H), 7.46 (m, 2H), 0.96 (s, 9H), 0.37 (s, 6H). ^{13}C NMR ($CDCl_3$) δ 235.9, 142.8, 132.5, 128.5, 127.6, 26.7, 16.9, −4.7. TLC: R_f 0.25 (hexane/EtOAc = 20:1).

Procedure for preparation of acylsilane 1u[7]

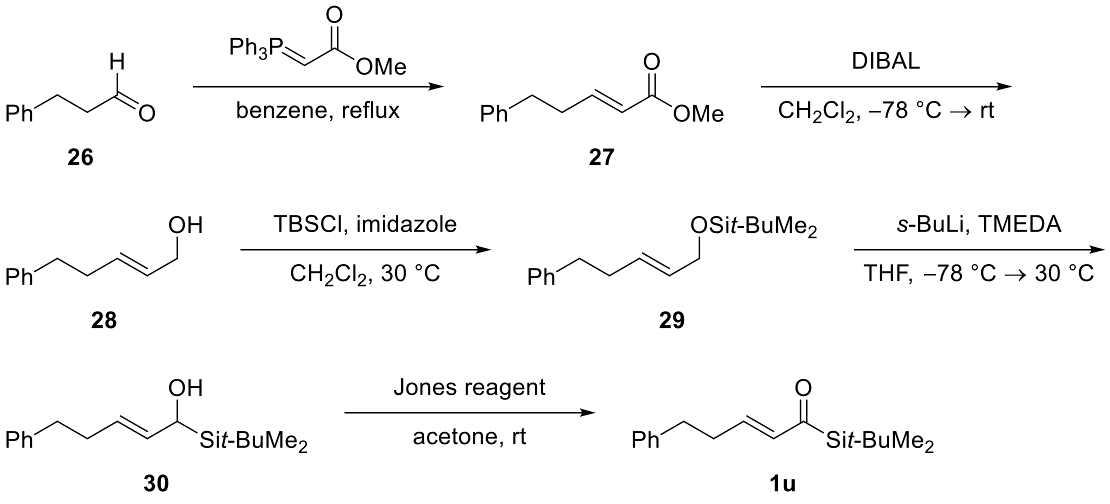

Procedure for preparation of **27**

To a solution of 3-phenylpropionaldehyde (**26**, 13 mL, 100 mmol) in benzene (0.10 L) was added methyl (triphenylphosphoranylidene)acetate (50 g, 150 mmol). After the mixture was refluxed for 17 h, the solvent was removed. To the crude product was added hexane (0.20 L) and Et_2O (20 mL), and precipitates were filtrated. The filtrate was concentrated in vacuo. Purification by flash silica gel column chromatography using hexane/EtOAc (v/v = 2:1) as an eluent gave **27**.

Methyl (*E*)-5-phenylpent-2-enoate (27): CAS RN [26429-97-0].

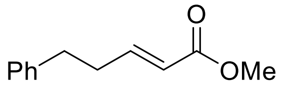

Colorless oil; 99% yield (19 g).
^{1}H NMR ($CDCl_3$) δ 7.30 (m, 2H), 7.22–7.17 (m, 3H), 7.01 (dt, J = 15.5, 7.0 Hz, 1H),

5.85 (dt, J = 15.5, 1.5 Hz, 1H), 3.72 (s, 3H), 2.78 (t, J = 7.5 Hz, 2H), 2.53 (ddt, J = 7.0, 1.5, 7.5 Hz, 2H). ^{13}C NMR ($CDCl_3$) δ 167.0, 148.4, 140.7, 128.5, 128.3, 126.2, 121.4, 51.4, 34.3, 33.9. TLC: R_f 0.50 (hexane/EtOAc = 2:1).

Procedure for preparation of **28**

To a solution of **27** (19 g, 99 mmol) in dry CH_2Cl_2 (0.20 L) was slowly added DIBAL (0.25 L, 1.03 M in hexane, 0.25 mol) at –78 °C, and the reaction mixture was allowed to warm to ambient temperature. After the mixture was stirred for 1 h, MeOH (0.10 L) and aqueous potassium sodium tartrate (0.30 L, 30w/v%) were added at 0 °C. After the mixture was stirred at ambient temperature for 1 h, the aqueous layers were extracted with CH_2Cl_2 (0.20 L × 3), and the combined organic layers were washed with H_2O and brine, dried over Na_2SO_4, and concentrated in vacuo. Purification by flash silica gel column chromatography using hexane/EtOAc (v/v = 2:1) as an eluent gave **28**.

(*E*)-5-Phenylpent-2-en-1-ol (28): CAS RN [75553-23-0].

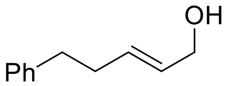

Colorless oil; 70% yield (11 g).

^1H NMR ($CDCl_3$) δ 7.28 (m, 2H), 7.21–7.18 (m, 3H), 5.77–5.64 (m, 2H), 4.12 (m, 2H), 2.71 (t, J = 7.5 Hz, 2H), 2.39 (m, 2H). ^{13}C NMR ($CDCl_3$) δ 141.7, 132.3, 129.6, 128.4, 128.3, 125.9, 63.7, 35.5, 34.0. TLC: R_f 0.38 (hexane/EtOAc = 2:1).

Procedure for preparation of **29**

To a solution of **28** (11 g, 70 mmol) in dry CH_2Cl_2 (0.14 L) was added imidazole (7.1 g, 105 mmol) and *tert*-butylchlorodimethylsilane (16 g, 0.11 mol). After being stirred at 30 °C for 18 h, the reaction was quenched with H_2O (0.20 L). The aqueous layers were extracted with CH_2Cl_2 (0.10 L × 3), and the combined organic layers were washed with H_2O and brine, dried over Na_2SO_4, and concentrated in vacuo. Purification by flash silica gel column chromatography using hexane/EtOAc (v/v = 10:1) as an eluent gave **29**.

(*E*)-*tert*-Butyldimethyl((5-phenylpent-2-en-1-yl)oxy)silane (29): CAS RN [160805-54-9].

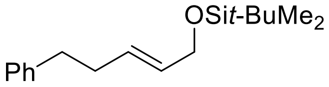

Colorless oil; 74% yield (14 g).

^{1}H NMR ($CDCl_3$) δ 7.28 (m, 2H), 7.19–7.14 (m, 3H), 5.69 (dt, *J* = 15.5, 6.0 Hz, 1H), 5.58 (dt, *J* = 15.5, 7.5 Hz, 1H), 4.12 (d, *J* = 6.0 Hz, 2H), 2.70 (t, *J* = 7.5 Hz, 2H), 2.36 (dt, *J* = 7.5, 7.5 Hz, 2H), 0.90 (s, 9H), 0.06 (s, 6H). ^{13}C NMR ($CDCl_3$) δ 141.9, 130.3, 129.8, 128.4, 128.3, 125.8, 63.9, 35.6, 34.0, 26.0, 18.4, –5.1. TLC: R_f 0.50 (hexane/EtOAc = 10:1).

Procedure for preparation of **30**

To a solution of **29** (1.4 g, 5.0 mmol) in dry THF (20 mL) was slowly added *N*,*N*,*N'*,*N'*-tetramethylethylenediamine (3.3 mL, 22 mmol) and *sec*-butyllithium (17 mL, 1.2 M in hexane, 20 mmol) at –78 °C, and the reaction mixture was allowed to warm to 25 °C. After being stirred for 1 h, the reaction was quenched with saturated aqueous NH_4Cl (20 mL). The aqueous layers were extracted with Et_2O (20 mL × 3), and the combined organic layers were washed with H_2O and brine, dried over Na_2SO_4, and concentrated in vacuo. Purification by flash silica gel column chromatography using hexane/EtOAc (v/v = 20:1) as an eluent gave **30**.

(*E*)-1-(*tert*-Butyldimethylsilyl)-5-phenylpent-2-en-1-ol (30): CAS RN [749926-98-5].

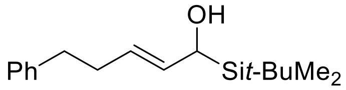

Colorless oil; 37% yield (0.51 g).

^{1}H NMR ($CDCl_3$) δ 7.27 (dd, *J* = 7.5, 7.5 Hz, 2H), 7.19–7.16 (m, 3H), 5.67 (dd, *J* = 15.0, 6.0 Hz, 1H), 5.52 (dt, *J* = 15.0, 7.0 Hz, 1H), 4.06 (d, *J* = 6.0 Hz, 1H), 2.70 (t, *J* = 7.0 Hz, 2H), 2.38 (dt, *J* = 7.0, 7.0 Hz, 2H), 0.93 (s, 9H), –0.03 (s, 3H), –0.10 (s, 3H). ^{13}C NMR ($CDCl_3$) δ 141.8, 132.9, 128.4, 128.3, 126.4, 125.8, 66.8, 36.0, 34.2, 26.9, 16.9, –7.7, –9.0. TLC: R_f 0.19 (hexane/EtOAc = 20:1).

Procedure for preparation of **1u**

To a solution of **30** (0.55 g, 2.0 mmol) in dry acetone (20 mL) was slowly added Jones reagent (1.0 mL, ca. 2.5 M in aqueous H_2SO_4, ca. 2.5 mmol) dropwise at ambient temperature. After being stirred for 30 min, the reaction was quenched with H_2O (40 mL). The aqueous layers were extracted with Et_2O (20 mL × 3), and the combined organic layers were washed with H_2O and brine, dried over Na_2SO_4, and concentrated in vacuo. Purification by flash silica gel column chromatography using hexane/EtOAc (v/v = 5:1) as an eluent gave **1u**.

(*E*)-1-(*tert*-Butyldimethylsilyl)-5-phenylpent-2-en-1-one (1u): CAS RN [749926-99-6].

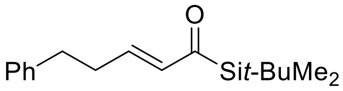

Pale yellow oil; 26% yield (0.14 g).

^{1}H NMR ($CDCl_3$) δ 7.29 (dd, *J* = 7.0, 7.0 Hz, 2H), 7.22–7.17 (m, 3H), 6.66 (dt, *J* = 16.0, 6.5 Hz, 1H), 6.35 (d, *J* = 16.0 Hz, 1H), 2.79 (t, *J* = 6.5 Hz, 2H), 2.54 (dt, *J* = 6.5, 6.5 Hz, 2H), 0.90 (s, 9H), 0.18 (s, 6H). ^{13}C NMR ($CDCl_3$) δ 235.8, 145.2, 140.7, 136.8, 128.5, 128.4, 126.2, 34.5, 34.2, 26.6, 16.6, –6.0. TLC: R_f 0.50 (hexane/EtOAc = 5:1).

Supplementary Discussion

Supplementary Table 1. Screening of Catalysts[a]

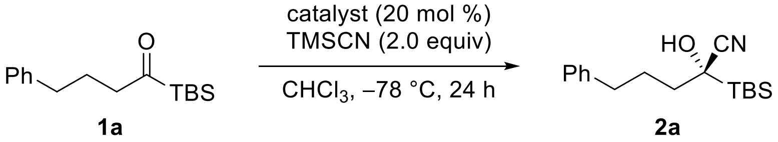

entry	catalyst	yield (%)[b]	*ee* (%)
1	**3a**	88	94
2	**3b**	42	83
3	**3c**	22	49
4	**3d**	31	–61
5	**3e**	23	19
6	**3f**	24	41
7	**3g**	<5	—
8[c]	**3h**	93	21
9	**3i**	79	–68
10	**3j**	20	80
11	**3k**	53	–69
12	**3l**	27	97
13	**3m**	35	–98
14	none	<1	—

[a] Reactions were run using **1a** (0.20 mmol), TMSCN (0.40 mmol), and the catalyst (0.040 mmol) in $CHCl_3$ (0.20 mL). [b] Isolated yields. [c] The trimethylsilyl ether of **2a** was obtained as the product.

(Supplementary Table 1)

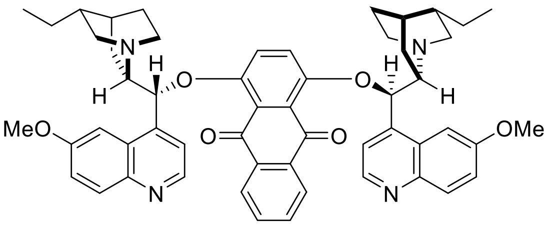

3a

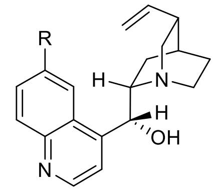

3b: R = OMe
3c: R = H

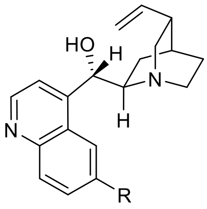

3d: R = OMe
3e: R = H

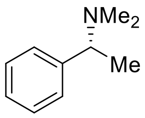

3f

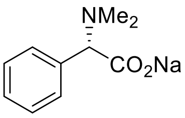

3g

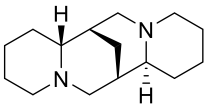

3h

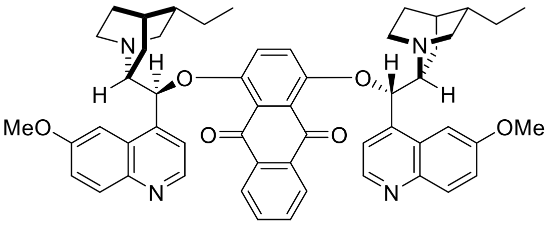

3i

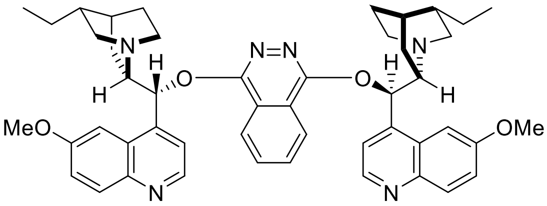

3j

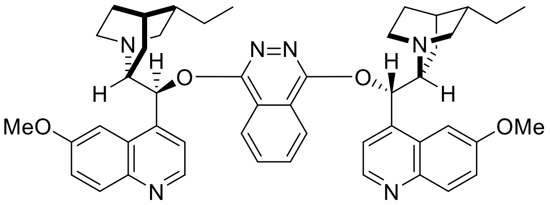

3k

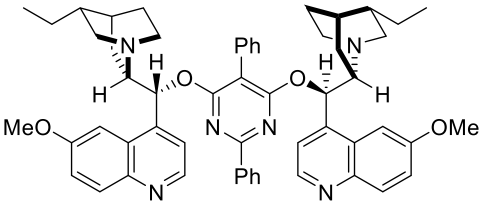

3l

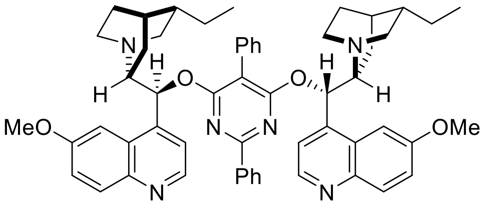

3m

Supplementary Table 2. Optimization Studies[a]

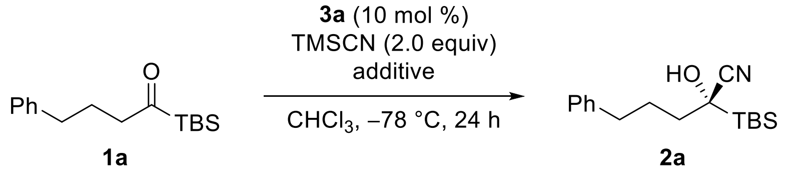

entry	additive (equiv)	yield (%)[b]	*ee* (%)
1	none	37	95
2	MeOH (2.0)	90	82
3	*i*-PrOH (2.0)	91	87
4	*t*-BuOH (2.0)	33	95
5	*s*-BuOH (2.0)	86	88
6	PhOH (2.0)	77	78
7[c]	*i*-PrOH (1.0)	88	91
8[d]	*i*-PrOH (1.0)	38	89
9[e]	*i*-PrOH (1.0)	81	78
10[f]	*i*-PrOH (1.0)	85	91
11[g]	*i*-PrOH (1.0)	54	89
12[h]	*i*-PrOH (1.0)	65	87
13[c,i]	*i*-PrOH (1.0)	72	87
14[c,j]	*i*-PrOH (1.0)	80	81

[a] Reactions were run using **1a** (0.20 mmol), TMSCN (0.40 mmol), the additive, and **3a** (0.020 mmol) in $CHCl_3$ (0.20 mL). [b] Isolated yields. [c] Reaction was run using 5.0 mol % (0.010 mmol) of **3a**. [d] Reaction was run using 1.0 mol % (0.0020 mmol) of **3a**. [e] Reaction was run using MS3A (150 mg). [f] Reaction was run using MS4A (150 mg). [g] Reaction was run using MS5A (150 mg). [h] Reaction was run using MS13X (150 mg). [i] Reaction was run at –40 °C. [j] Reaction was run at 0 °C.

Supplementary Scheme 1. Reaction of **1a** on a 1.0 mmol Scale

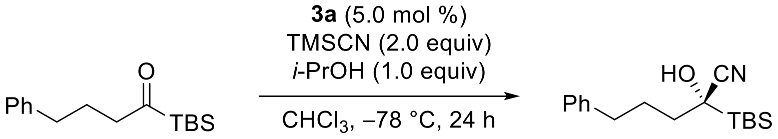

1a
(1.0 mmol)

2a
75%, 90% *ee*

Supplementary Scheme 2. ^{1}H NMR Study of the Solution of TMSCN and **3a**

(a) TMSCN:**3a** = 1:1

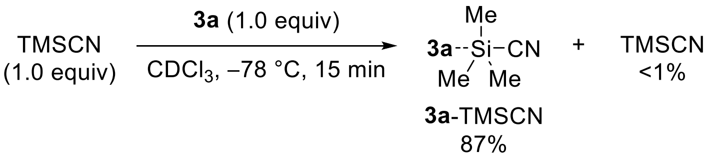

Procedure

A solution of TMSCN (2.5 μL, 0.010 mmol) and **3a** (8.6 mg, 0.010 mmol) in $CDCl_3$ (0.10 mL) was stirred in an NMR sample tube at –78 °C for 15 min under argon atomsphere. Subsequently, the solution was diluted with $CDCl_3$ (0.40 mL) to carry out the ^{1}H NMR analyses at –60 °C.

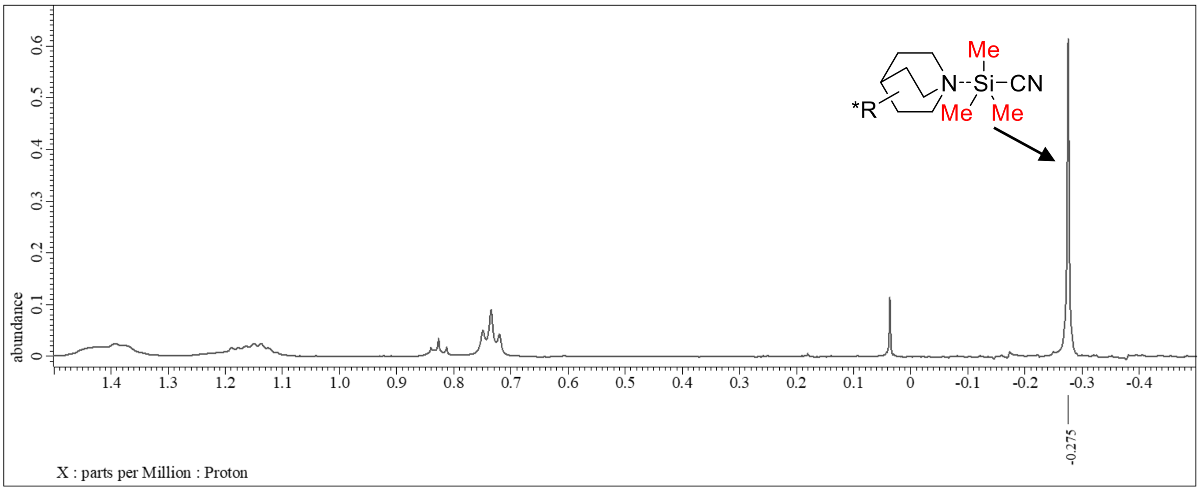

(b) TMSCN (a material prepared for the reference)

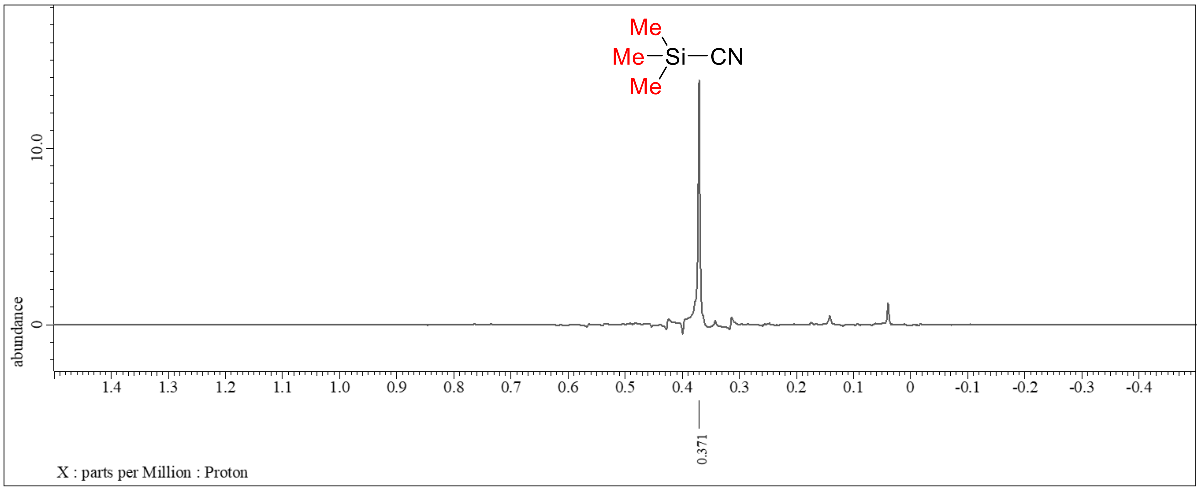

Supplementary Scheme 3. ^{1}H NMR Study of the Solution of TMSCN and *i*-PrOH

(a) TMSCN:*i*-PrOH = 1:1

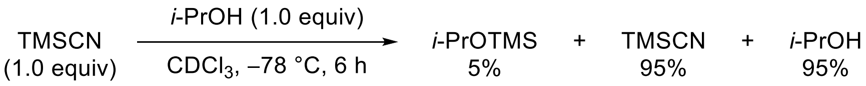

Procedure

A solution of TMSCN (25 μL, 0.20 mmol) and *i*-PrOH (15 μL, 0.20 mmol) in $CDCl_3$ (0.10 mL) was stirred in an NMR sample tube at –78 °C for 6 h under argon atomsphere. Subsequently, the solution was diluted with $CDCl_3$ (0.40 mL) to carry out the ^{1}H NMR analyses at –60 °C.

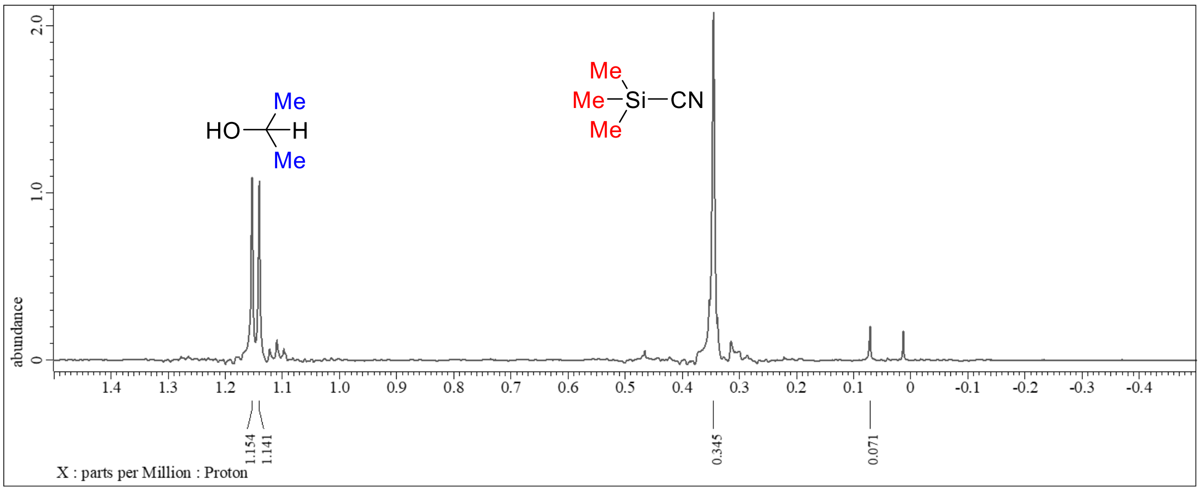

(b) *i*-PrOTMS, TMSOTMS (materials prepared for the reference)

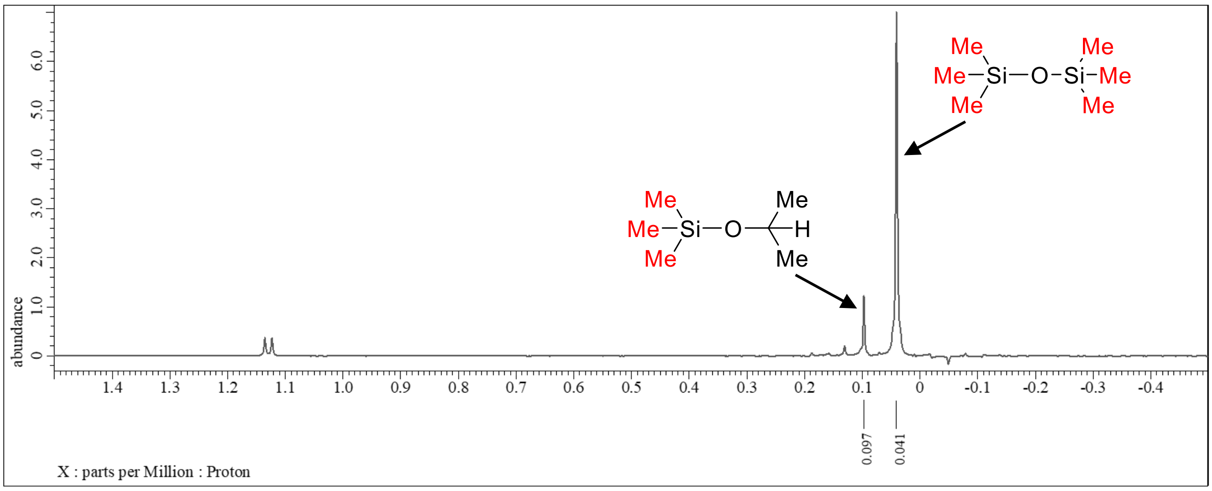

Supplementary Scheme 4. ^{1}H NMR Studies of the Solutions of TMSCN, Alcohols, and **3a**

(a) TMSCN:MeOH:**3a** = 2:1:0.05

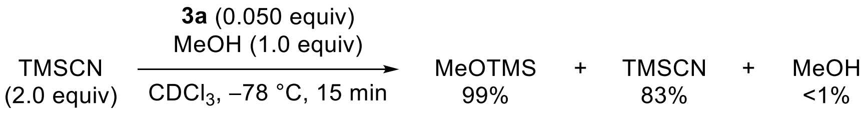

Procedure

A solution of TMSCN (50 µL, 0.40 mmol), MeOH (8.1 µL, 0.20 mmol), and **3a** (8.6 mg, 0.010 mmol) in $CDCl_3$ (0.10 mL) was stirred in an NMR sample tube at –78 °C for 15 min under argon atomsphere. Subsequently, the solution was diluted with $CDCl_3$ (0.40 mL) to carry out the ^{1}H NMR analyses at –60 °C.

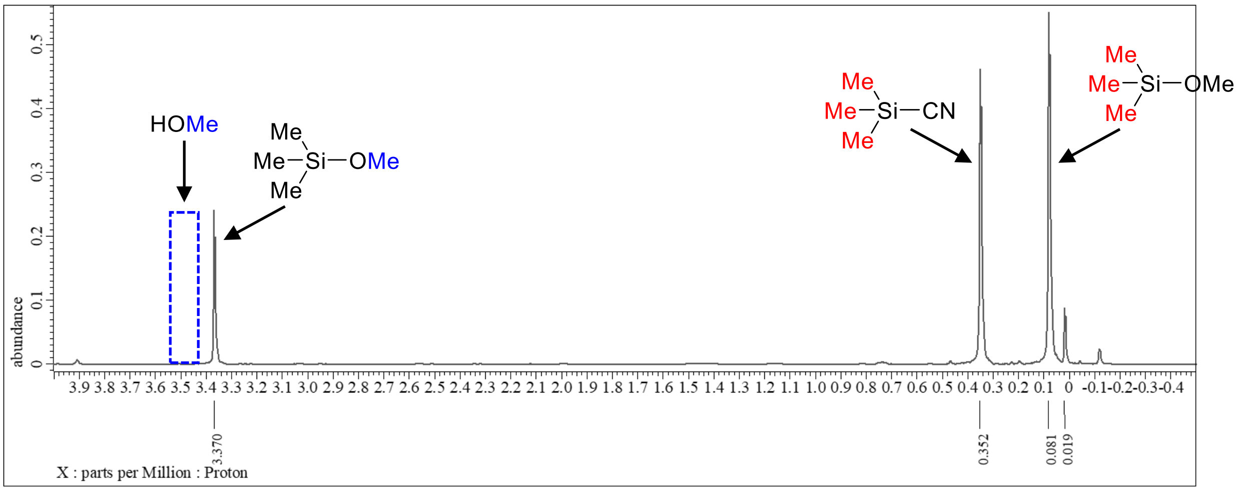

(b) TMSCN:*i*-PrOH:**3a** = 2:1:0.05

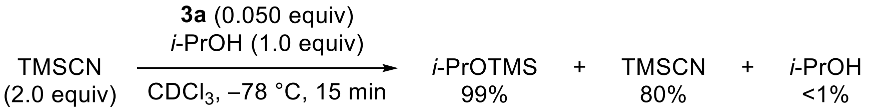

Procedure

A solution of TMSCN (50 μL, 0.40 mmol), *i*-PrOH (15 μL, 0.20 mmol), and **3a** (8.6 mg, 0.010 mmol) in $CDCl_3$ (0.10 mL) was stirred in an NMR sample tube at –78 °C for 15 min under argon atomsphere. Subsequently, the solution was diluted with $CDCl_3$ (0.40 mL) to carry out the ^{1}H NMR analyses at –60 °C.

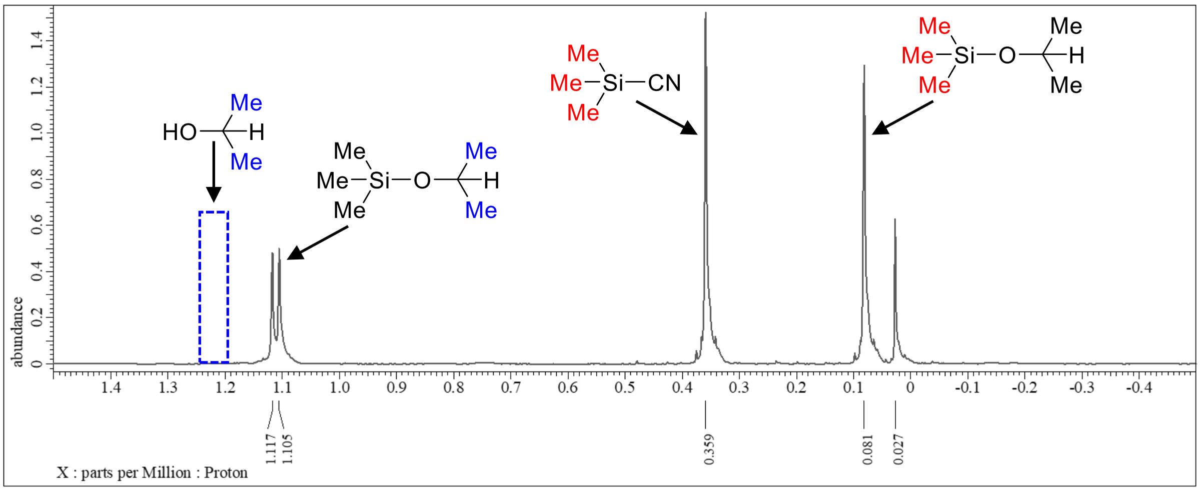

Supplementary Scheme 5. Studies of **3a**-TMSCN Complexes

(a) coodination of **3a** to TMSCN

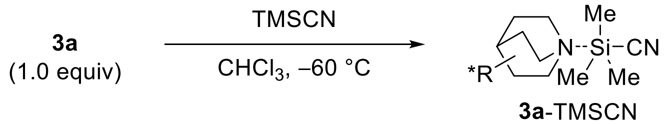

entry	TMSCN (equiv)	**3a**-TMSCN (%)[a]
1	1.0	78
2	2.0	127

[a] Yields were determined by ^{1}H NMR spectroscopy using 1,1,2,2-tetrachloroethane as an internal standard. They are values calculated with 1.0 equivalent of starting material identified as 100%, and the theoretical maximum yield of **3a**-TMSCN is 100% (entry 1) and 200% (entry 2), respectively.

Procedure

A solution of **3a** (8.6 mg, 0.010 mmol), TMSCN (1.2 μL, 0.010 mmol), and 1,1,2,2-tetrachloroethane (5.0 μL, 0.048 mmol) in $CDCl_3$ (0.70 mL) was stirred in an NMR sample tube at –78 °C for 30 min under argon atomsphere, and the ^{1}H NMR analysis was carried out at –60 °C. Subsequently, additional TMSCN (1.2 μL, 0.010 mmol) was added. After the solution was stirred at –78 °C for 30 min, the ^{1}H NMR analyses were carried out at –60 °C.

(b) effects of TMSCN equivalents on enantioselectivities[a]

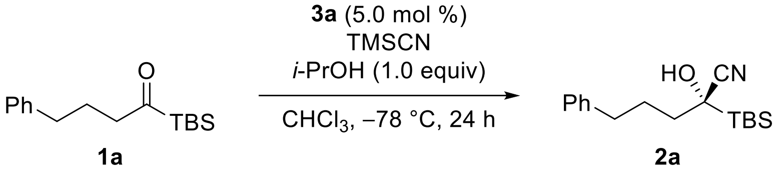

entry	TMSCN (equiv)	yield (%)[b]	*ee* (%)
1	1.0	33	96
2	2.0	88	91

[a] Reactions were run using **1a** (0.20 mmol), TMSCN, *i*-PrOH (0.20 mmol), and **3a** (0.010 mmol) in $CHCl_3$ (0.20 mL). [b] Isolated yields.

(c) reaction profiles of asymmetric cyanation of **1a**[a]

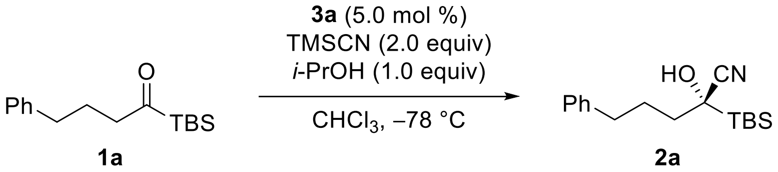

entry	reaction time (h)	yield (%)[b]	*ee* (%)
1	6	22	94
2	12	66	93
3	18	69	93
4	24	88	91

[a] Reactions were run using **1a** (0.20 mmol), TMSCN (0.40 mmol), *i*-PrOH (0.20 mmol), **3a** (0.010 mmol) in $CHCl_3$ (0.20 mL). [b] Isolated yields.

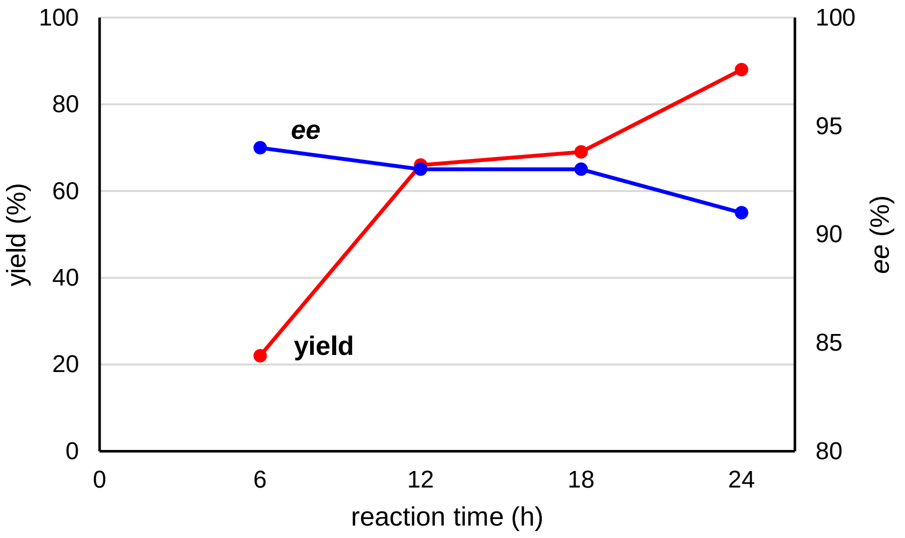

Supplementary Scheme 6. Reaction of **1a** with *i*-PrOH-d_8

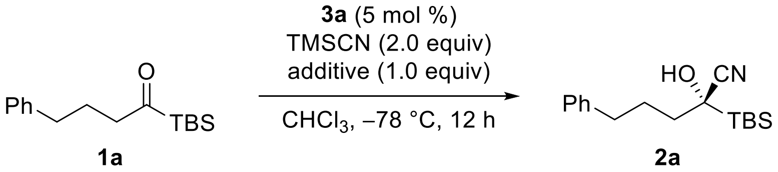

entry	additive	yield (%)[b]	*ee* (%)
1	*i*-PrOH	66	93
2	*i*-PrOH-d_8	54	94

[a] Reactions were run using **1a** (0.20 mmol), TMSCN (0.40 mmol), the additive (0.20 mmol), and **3a** (0.010 mmol) in $CHCl_3$ (0.20 mL). [b] Isolated yields.

Supplementary Scheme 7. Reactions of Conjugated Acylsilanes

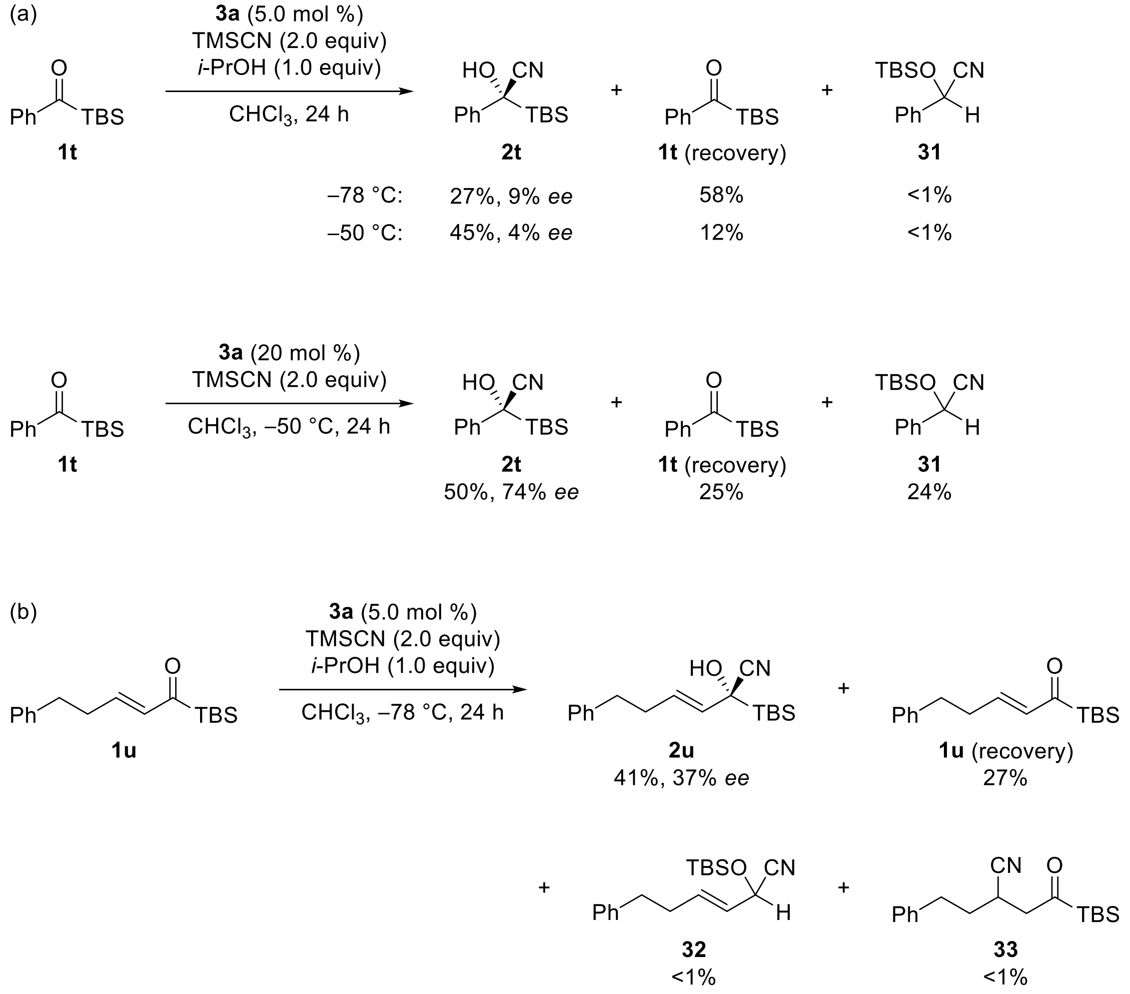

Supplementary Scheme 8. Protection of **2a** with Conventional Methods

(a) benzylation of **2a**

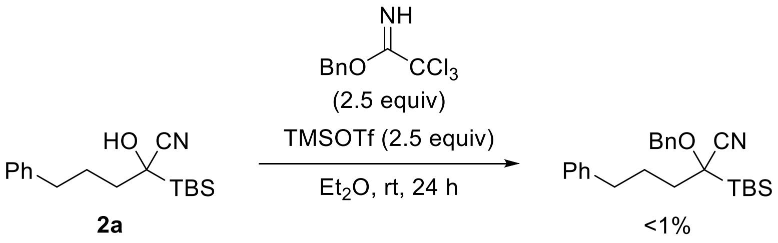

Procedure[8]

To a 5-mL vial were sequentially added acylsilane cyanohydrin **2a** (29 mg, 0.10 mmol), benzyl 2,2,2-trichloroacetimidate (50 μL, 0.25 mmol), and Et_2O (0.50 mL). After the reaction mixture was stirred at 0 °C for 30 min, trimethylsilyl trifluoromethanesulfonate (45 μL, 0.25 mmol) was added. The mixture was stirred at ambient temperature for 24 h. The reaction mixture was subsequently diluted with EtOAc, passed through a short silica gel pad, and concentrated in vacuo.

(b) trimethylsilylation of **2a**

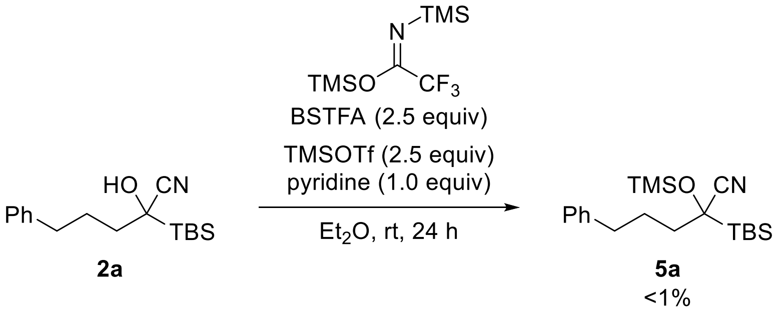

Procedure[9]

To a 5-mL vial were sequentially added acylsilane cyanohydrin **2a** (29 mg, 0.10 mmol), *N*,*O*-bis(trimethylsilyl)trifluoroacetamide (66 μL, 0.25 mmol), Et_2O (0.50 mL), trimethylsilyl trifluoromethanesulfonate (45 μL, 0.25 mmol), and pyridine (8.0 μL, 0.10 mmol). The mixture was stirred at ambient temperature for 24 h. The reaction mixture was subsequently diluted with EtOAc, passed through a short silica gel pad, and concentrated in vacuo.

Supplementary Scheme 9. 3h-Catalyzed Silylation of Rcemic **2a** with 0.5 equivalent of TMSCN

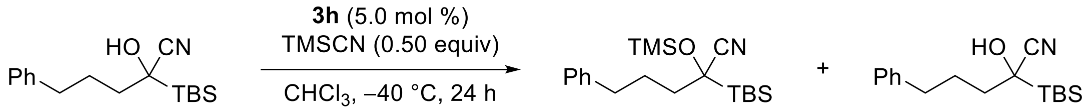

2a
(racemate)

5a
22%, <1% *ee*

2a (recovery)
65%, <1% *ee*

Supplementary Scheme 10. Trimethylsilylation of **2a** with HMDS and I_2

(a) 25 °C

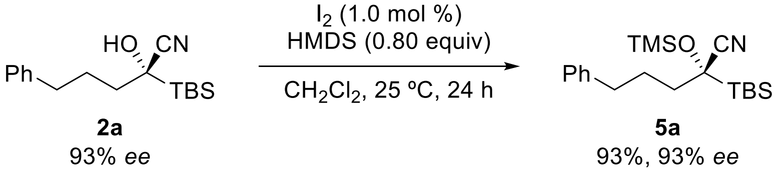

(b) –40 °C

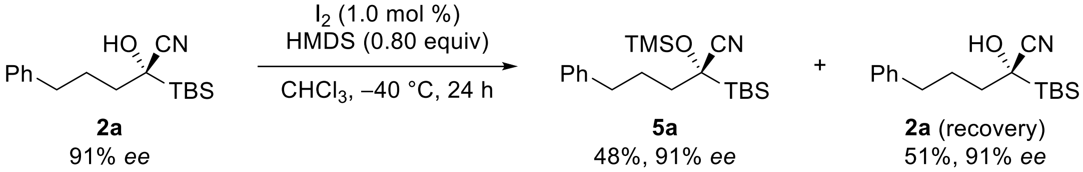

Procedure[10]

To a 5-mL vial were sequentially added **2a** (58 mg, 0.20 mmol), CH_2Cl_2 (0.80 mL), iodine (0.51 mg, 0.0020 mmol), and 1,1,1,3,3,3-hexamethyldisilazane (34 μL, 0.16 mmol). The mixture was stirred at the reaction temperatures for 24 h. The reaction mixture was subsequently diluted with EtOAc, passed through a short silica gel pad, and concentrated in vacuo. Purification of the crude product by flash silica gel column chromatography using hexane/EtOAc (v/v = 20:1) as an eluent afforded **5a**.

Characterization Data of Products

(*S*)-2-(*tert*-Butyldimethylsilyl)-2-hydroxy-5-phenylpentanenitrile (2a).

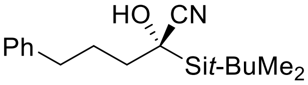

Yield: 88% yield (51 mg), 91% *ee*, white solid. $[\alpha]_D^{20}$ −11.0 (*c* 0.86, $CHCl_3$). ^1H NMR ($CDCl_3$) δ 7.30 (dd, *J* = 8.0, 6.5 Hz, 2H), 7.22−7.17 (m, 3H), 2.70 (m, 2H), 1.94 (m, 2H), 1.91 (s, 1H), 1.83 (m, 2H), 1.04 (s, 9H), 0.17 (s, 3H), 0.16 (s, 3H). ^{13}C NMR ($CDCl_3$) δ 141.2, 128.5, 128.3, 126.1, 122.3, 64.5, 36.3, 35.5, 27.3, 25.5, 18.1, −7.7, –8.2. Mp. 89.1−89.9 °C. TLC: R_f 0.18 (hexane/EtOAc = 10:1). IR (KBr): 3371, 2949, 2229, 1460, 1382, 1250, 1064, 841, 777, 566 cm^{-1}. HRMS (ESI) Calcd for $C_{17}H_{27}NOSiNa$: $[M+Na]^+$, 312.1754. Found: *m*/*z* 312.1757. HPLC (Daicel Chiralpak IB, hexane/*i*-PrOH = 98/2, flow rate = 0.5 mL/min, λ = 215 nm, 30 °C): t_{major} = 15.4 min, t_{minor} = 16.5 min.

2-Hydroxy-5-phenyl-2-(trimethylsilyl)pentanenitrile (2b).

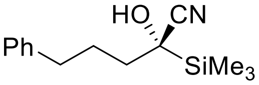

Yield: 84% yield (42 mg), 65% *ee*, colorless oil. $[\alpha]_D^{20}$ –8.9 (*c* 1.35, $CHCl_3$). ^1H NMR ($CDCl_3$) δ 7.30 (dd, *J* = 8.0, 7.0 Hz, 2H), 7.21 (t, *J* = 7.0 Hz, 1H), 7.20 (d, *J* = 8.0 Hz, 2H), 2.70 (m, 2H), 1.94 (m, 2H), 1.91 (s, 1H), 1.77 (t, *J* = 8.0 Hz, 2H), 0.21 (s, 9H). ^{13}C NMR ($CDCl_3$) δ 141.3, 128.5, 128.3, 126.1, 121.8, 64.3, 35.6, 35.2, 25.9, –4.6. TLC: R_f 0.13 (hexane/EtOAc = 10:1). IR (neat): 3434, 2957, 2218, 1604, 1454, 1254, 1059, 850, 756, 501 cm^{-1}. HRMS (ESI) Calcd for $C_{14}H_{21}NOSiNa$: $[M+Na]^+$, 270.1285. Found: *m*/*z* 270.1286. HPLC (Daicel Chiralpak IA, hexane/*i*-PrOH = 98/2, flow rate = 1.0 mL/min, λ = 215 nm, 30 °C): t_{major} = 13.5 min, t_{minor} = 14.7 min.

2-Hydroxy-5-phenyl-2-(triethylsilyl)pentanenitrile (2c).

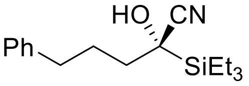

Yield: 66% yield (38 mg), 92% *ee*, colorless oil. $[\alpha]_D^{20}$ –13.3 (*c* 0.65, $CHCl_3$). ^1H NMR ($CDCl_3$) δ 7.29 (dd, *J* = 8.0, 6.0 Hz, 2H), 7.21 (t, *J* = 6.0 Hz, 1H), 7.19 (d, *J* = 8.0 Hz, 2H), 2.70 (m, 2H), 1.94 (m, 2H), 1.89 (s, 1H), 1.80 (m, 2H), 1.05 (t, *J* = 8.0 Hz, 9H), 0.78 (q, *J* = 8.0 Hz, 6H). ^{13}C NMR ($CDCl_3$) δ 141.3, 128.5, 128.3, 126.1, 122.2, 64.1, 35.9, 35.5, 25.6, 7.3, 1.4. TLC: R_f 0.20 (hexane/EtOAc = 10:1). IR (neat): 3419, 2920, 2219, 1604, 1455, 1420, 1242, 1016, 722, 481 cm^{-1}. HRMS (ESI) Calcd for $C_{17}H_{27}NOSiNa$: $[M+Na]^+$, 312.1754. Found: *m*/*z* 312.1756. HPLC (Daicel Chiralpak

IA, hexane/*i*-PrOH = 98/2, flow rate = 1.0 mL/min, λ = 215 nm, 30 °C): t_{major} = 12.5 min, t_{minor} = 13.4 min.

2-(Dimethyl(phenyl)silyl)-2-hydroxy-5-phenylpentanenitrile (2d).

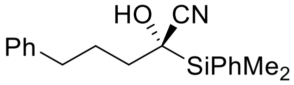

Yield: 78% yield (48 mg), 53% *ee*, white solid. $[\alpha]_D^{20}$ –3.2 (*c* 0.83, $CHCl_3$). ^1H NMR ($CDCl_3$) δ 7.62 (m, 2H), 7.47 (m, 1H), 7.42 (m, 2H), 7.27 (m, 2H), 7.19 (m, 1H), 7.14 (d, *J* = 8.0 Hz, 2H), 2.63 (t, *J* = 8.0 Hz, 2H), 1.90 (m, 2H), 1.82 (s, 1H), 1.73 (t, *J* = 7.5 Hz, 2H), 0.53 (s, 3H), 0.51 (s, 3H). ^{13}C NMR ($CDCl_3$) δ 141.3, 134.6, 132.2, 130.6, 128.4, 128.3 (2C), 126.0, 121.7, 64.2, 35.5, 35.3, 25.9, –6.1, –6.5. Mp. 54.2–54.8 °C. TLC: R_f 0.20 (hexane/EtOAc = 10:1). IR (KBr): 3454, 2936, 2211, 1429, 1260, 1116, 959, 889, 695, 467 cm^{-1}. HRMS (ESI) Calcd for $C_{19}H_{23}NOSiNa$: $[M+Na]^+$, 332.1441. Found: *m*/*z* 332.1443. HPLC (Daicel Chiralpak IB, hexane/*i*-PrOH = 98/2, flow rate = 1.0 mL/min, λ = 215 nm, 30 °C): t_{minor} = 11.2 min, t_{major} = 12.1 min.

2-Hydroxy-5-phenyl-2-(triisopropylsilyl)pentanenitrile (2e).

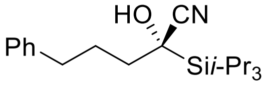

Yield: 10% yield (6.6 mg), 80% *ee*, white solid. $[\alpha]_D^{20}$ –17.4 (*c* 0.33, $CHCl_3$). ^1H NMR ($CDCl_3$) δ 7.29 (dd, *J* = 6.0, 4.0 Hz, 2H), 7.20 (d, *J* = 6.0 Hz, 2H), 7.19 (t, *J* = 4.0 Hz, 1H), 2.71 (m, 2H), 2.01–1.92 (m, 3H), 1.95 (s, 1H), 1.84 (m, 1H), 1.37 (sept, *J* = 7.5 Hz, 3H), 1.17 (d, *J* = 7.5 Hz, 18H). ^{13}C NMR ($CDCl_3$) δ 141.3, 128.5, 128.4, 126.1, 122.7, 64.5, 36.6, 35.5, 25.2, 18.9, 10.7. Mp. 44.5–45.0 °C. TLC: R_f 0.25 (hexane/EtOAc = 10:1). IR (KBr): 3397, 2945, 2218, 1461, 1296, 1069, 887, 675, 581, 465 cm^{-1}. HRMS (ESI) Calcd for $C_{20}H_{33}NOSiNa$: $[M+Na]^+$, 354.2224. Found: *m*/*z* 354.2227. HPLC (Daicel Chiralpak IB, hexane/*i*-PrOH = 98/2, flow rate = 1.0 mL/min, λ = 215 nm, 30 °C): t_{major} = 6.4 min, t_{minor} = 7.5 min.

2-(*tert*-Butyldimethylsilyl)-2-cyclohexyl-2-hydroxyacetonitrile (2f).

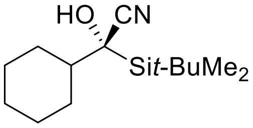

Yield: 17% yield (8.6 mg), 71% *ee*, white solid. $[\alpha]_D^{20}$ –11.4 (*c* 0.39, $CHCl_3$). ^1H NMR ($CDCl_3$) δ 2.05 (s, 1H), 1.97 (m, 1H), 1.87–1.80 (m, 4H), 1.69 (m, 1H), 1.29–1.17

(m, 5H), 1.05 (s, 9H), 0.25 (s, 3H), 0.22 (s, 3H). ^{13}C NMR ($CDCl_3$) δ 121.7, 69.3, 44.4, 28.7, 27.3, 26.9, 26.0, 25.9, 25.8, 18.6, –5.5, –6.6. Mp. 52.3–52.8 °C. TLC: R_f 0.35 (hexane/EtOAc = 10:1). IR (KBr): 3411, 2920, 2221, 1450, 1264, 1075, 941, 832, 584, 461 cm^{-1}. HRMS (ESI) Calcd for $C_{14}H_{27}NOSiNa$: $[M+Na]^+$, 276.1754. Found: *m*/*z* 276.1753. HPLC (Daicel Chiralpak IC, hexane/*i*-PrOH = 98/2, flow rate = 0.5 mL/min, λ = 220 nm, 30 °C): t_{minor} = 14.3 min, t_{major} = 15.0 min.

2-(*tert*-Butyldimethylsilyl)-2-hydroxy-4-phenylbutanenitrile (2g).

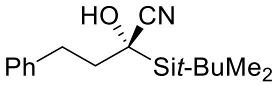

Yield: 82% yield (45 mg), 95% *ee*, white solid. $[\alpha]_D^{20}$ –19.3 (*c* 1.50, $CHCl_3$). ^{1}H NMR ($CDCl_3$) δ 7.33 (m, 2H), 7.28–7.23 (m, 3H), 2.97 (m, 2H), 2.08 (m, 2H), 1.05 (s, 9H), 0.19 (s, 3H), 0.18 (s, 3H). ^{13}C NMR ($CDCl_3$) δ 140.5, 128.9, 128.6, 126.5, 122.1, 64.9, 38.5, 30.6, 27.3, 18.2, –7.7, –8.2. Mp. 67.5–68.0 °C. TLC: R_f 0.25 (hexane/EtOAc = 10:1). IR (KBr): 3426, 2929, 2214, 1456, 1330, 1255, 1043, 838, 694, 533 cm^{-1}. HRMS (ESI) Calcd for $C_{16}H_{25}NOSiNa$: $[M+Na]^+$, 298.1598. Found: *m*/*z* 298.1599. HPLC (Daicel Chiralpak IC, hexane/*i*-PrOH = 98/2, flow rate = 0.5 mL/min, λ = 220 nm, 30 °C): t_{minor} = 14.1 min, t_{major} = 17.5 min.

2-(*tert*-Butyldimethylsilyl)-7-chloro-2-hydroxyheptanenitrile (2h).

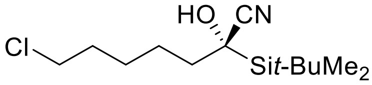

Yield: 74% yield (41 mg), 82% *ee*, white solid. $[\alpha]_D^{20}$ –11.6 (*c* 0.83, $CHCl_3$). ^{1}H NMR ($CDCl_3$) δ 3.56 (t, *J* = 6.5 Hz, 2H), 1.94 (s, 1H), 1.86–1.75 (m, 4H), 1.65 (m, 2H), 1.54 (m, 2H), 1.06 (s, 9H), 0.19 (s, 3H), 0.18 (s, 3H). ^{13}C NMR ($CDCl_3$) δ 122.3, 64.5, 44.8, 36.5, 32.3, 27.3, 26.7, 23.1, 18.2, –7.7, –8.2. Mp. 64.1–64.9 °C. TLC: R_f 0.20 (hexane/EtOAc = 10:1). IR (KBr): 3369, 2957, 2227, 1459, 1252, 1062, 841, 777, 577, 421 cm^{-1}. HRMS (ESI) Calcd for $C_{13}H_{26}ClNOSiNa$: $[M+Na]^+$, 298.1364. Found: *m*/*z* 298.1365. HPLC (Daicel Chiralpak IC, hexane/*i*-PrOH = 98/2, flow rate = 0.5 mL/min, λ = 215 nm, 30 °C): t_{major} = 2.6 min, t_{minor} = 3.4 min.

7-Bromo-2-(*tert*-butyldimethylsilyl)-2-hydroxyheptanenitrile (2i).

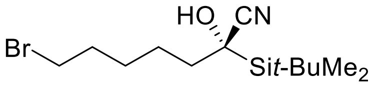

Yield: 84% yield (54 mg), 84% *ee*, white solid. $[\alpha]_D^{20}$ –7.8 (*c* 2.19, $CHCl_3$). ^{1}H NMR ($CDCl_3$) δ 3.43 (t, *J* = 6.5 Hz, 2H), 1.94 (s, 1H), 1.92 (m, 2H), 1.78 (m, 2H), 1.65

(m, 2H), 1.55 (m, 2H), 1.06 (s, 9H), 0.19 (s, 3H), 0.18 (s, 3H). ^{13}C NMR ($CDCl_3$) δ 122.3, 64.5, 36.5, 33.5, 32.5, 28.0, 27.3, 23.0, 18.2, –7.7, –8.2. Mp. 65.3–65.9 °C. TLC: R_f 0.23 (hexane/EtOAc = 10:1). IR (KBr): 3378, 2936, 2226, 1459, 1363, 1252, 1058, 776, 562, 422 cm^{-1}. HRMS (ESI) Calcd for $C_{13}H_{26}BrNOSiNa$: [M+Na]$^+$, 342.0859. Found: *m*/*z* 342.0852.

6-Bromo-1-(*tert*-butyldimethylsilyl)-1-cyanohexyl tosylcarbamate (2i').

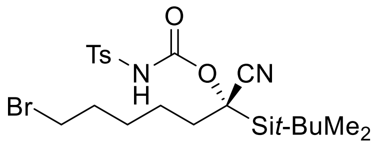

Yield: 73% yield (38 mg), 84% *ee*, colorless oil. $[\alpha]_D^{20}$ –27.0 (*c* 1.25, $CHCl_3$). ^{1}H NMR ($CDCl_3$) δ 9.16 (s, 1H), 7.95 (d, *J* = 8.0 Hz, 2H), 7.83 (d, *J* = 8.0 Hz, 2H), 3.28 (t, *J* = 7.0 Hz, 2H), 2.47 (s, 3H), 1.97 (m, 2H), 1.68 (m, 2H), 1.33 (m, 2H), 1.08 (m, 1H), 0.96 (m, 1H), 0.89 (s, 9H), 0.12 (m, 3H), 0.02 (m, 3H). ^{13}C NMR ($CDCl_3$) δ 157.4, 150.6, 147.0, 134.0, 130.0, 128.4, 86.7, 35.2, 33.2, 32.4, 27.7, 27.3, 21.8, 20.7, 18.2, –7.93, –7.99. TLC: R_f 0.20 (hexane/EtOAc = 10:1). IR (neat): 3327, 2935, 1800, 1682, 1311, 1178, 1089, 841, 757, 511 cm^{-1}. HRMS (ESI) Calcd for $C_{21}H_{33}BrN_2O_4SSiNa$: [M+Na]$^+$, 539.1006. Found: *m*/*z* 539.1003. HPLC (Daicel Chiralpak IC, hexane/*i*-PrOH = 90/10, flow rate = 1.0 mL/min, λ = 230 nm, 30 °C): t_{major} = 7.3 min, t_{minor} = 8.0 min.

6-(*tert*-Butyldimethylsilyl)-6-cyano-6-hydroxyhexyl benzoate (2j).

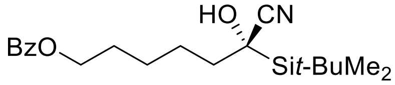

Yield: 73% yield (53 mg), 86% *ee*, white solid. $[\alpha]_D^{20}$ –9.87 (*c* 1.00, $CHCl_3$). ^{1}H NMR ($CDCl_3$) δ 8.05 (d, *J* = 8.0 Hz, 2H), 7.56 (t, *J* = 7.5 Hz, 1H), 7.45 (m, 2H), 4.35 (t, *J* = 6.5 Hz, 2H), 1.99 (s, 1H), 1.86–1.78 (m, 4H), 1.69 (m, 2H), 1.55 (m, 2H), 1.06 (s, 9H), 0.19 (s, 3H), 0.18 (s, 3H). ^{13}C NMR ($CDCl_3$) δ 166.7, 132.9, 130.3, 129.6, 128.4, 122.3, 64.7, 64.5, 36.6, 28.7, 27.4, 26.1, 23.5, 18.2, –7.7, –8.2. Mp. 35.1–35.8 °C. TLC: R_f 0.13 (hexane/EtOAc = 10:1). IR (KBr): 3412, 2952, 2216, 1695, 1281, 1129, 840, 720, 585, 419 cm^{-1}. HRMS (ESI) Calcd for $C_{20}H_{31}NO_3SiNa$: [M+Na]$^+$, 384.1965. Found: *m*/*z* 384.1956. HPLC (Daicel Chiralpak IB, hexane/*i*-PrOH = 95/5, flow rate = 2.0 mL/min, λ = 215 nm, 30 °C): t_{major} = 3.5 min, t_{minor} = 7.6 min.

S-(6-(*tert*-Butyldimethylsilyl)-6-cyano-6-hydroxyhexyl) benzothioate (2k).

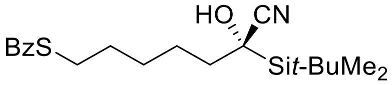

Yield: 53% yield (40 mg), 82% *ee*, white solid. $[\alpha]_D^{20}$ –10.2 (*c* 1.01, $CHCl_3$). ^1H NMR ($CDCl_3$) δ 7.97 (d, *J* = 8.0 Hz, 2H), 7.57 (t, *J* = 7.5 Hz, 1H), 7.45 (dd, *J* = 8.0, 7.5 Hz, 2H), 3.09 (m, 2H), 2.02 (s, 1H), 1.81–1.64 (m, 6H), 1.52 (m, 2H), 1.06 (s, 9H), 0.19 (s, 3H), 0.18 (s, 3H). ^{13}C NMR ($CDCl_3$) δ 192.0, 137.1, 133.3, 128.6, 127.2, 122.3, 64.5, 36.5, 29.4, 28.7, 28.5, 27.4, 23.2, 18.2, –7.7, –8.2. Mp. 41.3–41.7 °C. TLC: R_f 0.15 (hexane/EtOAc = 10:1). IR (KBr): 3457, 2950, 2217, 1665, 1465, 1206, 916, 838, 680, 422 cm^{-1}. HRMS (ESI) Calcd for $C_{20}H_{31}NO_2SSiNa$: $[M+Na]^+$, 400.1737. Found: *m*/*z* 400.1740. HPLC (Daicel Chiralpak IB, hexane/*i*-PrOH = 95/5, flow rate = 2.0 mL/min, λ = 230 nm, 30 °C): t_{major} = 4.7 min, t_{minor} = 8.7 min.

6-(*tert*-Butyldimethylsilyl)-6-cyano-6-hydroxyhexyl 4-methylbenzenesulfonate (2l).

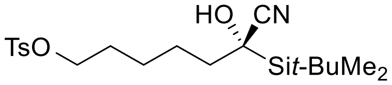

Yield: 75% yield (62 mg), 91% *ee*, colorless oil. $[\alpha]_D^{20}$ –9.4 (*c* 1.40, $CHCl_3$). ^1H NMR ($CDCl_3$) δ 7.79 (d, *J* = 8.5 Hz, 2H), 7.35 (d, *J* = 8.5 Hz, 2H), 4.04 (t, *J* = 6.0 Hz, 2H), 2.45 (s, 3H), 2.07 (s, 1H), 1.75–1.67 (m, 4H), 1.57 (m, 2H), 1.42 (m, 2H), 1.04 (s, 9H), 0.17 (s, 3H), 0.16 (s, 3H). ^{13}C NMR ($CDCl_3$) δ 144.8, 133.1, 129.9, 127.9, 122.2, 70.2, 64.3, 36.4, 28.7, 27.3, 25.3, 23.1, 21.6, 18.1, –7.7, –8.2. TLC: R_f 0.13 (hexane/EtOAc = 5:1). IR (neat): 3502, 2951, 1599, 1471, 1354, 1177, 940, 820, 668, 488 cm^{-1}. HRMS (ESI) Calcd for $C_{20}H_{33}NO_4SSiNa$: $[M+Na]^+$, 434.1792. Found: *m*/*z* 434.1788. HPLC (Daicel Chiralpak IC, hexane/*i*-PrOH = 80/20, flow rate = 2.0 mL/min, λ = 220 nm, 30 °C): t_{major} = 6.8 min, t_{minor} = 11.5 min.

Benzyl (6-(*tert*-butyldimethylsilyl)-6-cyano-6-hydroxyhexyl)carbamate (2m).

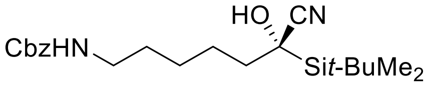

Yield: 81% yield (63 mg), 89% *ee*, colorless oil. $[\alpha]_D^{20}$ –8.5 (*c* 1.85, $CHCl_3$). ^1H NMR ($CDCl_3$) δ 7.36–7.35 (m, 4H), 7.32 (m, 1H), 5.10 (s, 2H), 4.81 (s, 1H), 3.22 (m, 2H), 2.54 (s, 1H), 1.76 (m, 2H), 1.64 (m, 2H), 1.55 (m, 2H), 1.40 (m, 2H), 1.05 (s, 9H), 0.18 (s, 3H), 0.17 (s, 3H). ^{13}C NMR ($CDCl_3$) δ 156.5, 136.5, 128.5, 128.12, 128.09, 122.5, 66.7, 64.2, 40.6, 36.5, 29.9, 27.3, 26.4, 23.3, 18.2, –7.7, –8.2. TLC: R_f 0.23 (hexane/EtOAc = 5:1). IR (neat): 3381, 2944, 1713, 1535, 1254, 1141, 1016, 839, 755, 504 cm^{-1}. HRMS (ESI) Calcd for $C_{21}H_{34}N_2O_3SiNa$: $[M+Na]^+$, 413.2231. Found: *m*/*z*

413.2231. HPLC (Daicel Chiralpak IE, hexane/*i*-PrOH = 90/10, flow rate = 1.0 mL/min, λ = 215 nm, 30 °C): t_{major} = 13.8 min, t_{minor} = 14.9 min.

***N*-(6-(*tert*-Butyldimethylsilyl)-6-cyano-6-hydroxyhexyl)benzamide (2n)**.

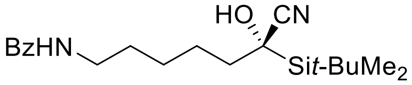

Yield: 79% yield (57 mg), 92% *ee*, white solid. $[\alpha]_D^{20}$ –10.7 (*c* 1.89, $CHCl_3$). ^1H NMR ($CDCl_3$) δ 7.77 (d, *J* = 7.5 Hz, 2H), 7.50 (t, *J* = 7.5 Hz, 1H), 7.43 (dd, *J* = 7.5, 7.5 Hz, 2H), 6.18 (s, 1H), 3.50 (m, 2H), 1.85–1.63 (m, 6H), 1.48 (m, 2H), 1.05 (s, 9H), 0.18 (s, 3H), 0.17 (s, 3H). ^{13}C NMR ($CDCl_3$) δ 167.7, 134.6, 131.4, 128.6, 126.8, 122.5, 64.2, 39.6, 36.4, 29.7, 27.4, 26.6, 23.4, 18.2, –7.7, –8.2. Mp. 148.5–149.0 °C. TLC: R_f 0.18 (hexane/EtOAc = 2:1). IR (KBr): 3373, 2944, 2219, 1644, 1539, 1251, 1076, 839, 711, 430 cm^{-1}. HRMS (ESI) Calcd for $C_{20}H_{32}N_2O_2SiNa$: $[M+Na]^+$, 383.2125. Found: *m*/*z* 383.2125. HPLC (Daicel Chiralpak ID, hexane/*i*-PrOH = 90/10, flow rate = 1.0 mL/min, λ = 230 nm, 30 °C): t_{major} = 18.9 min, t_{minor} = 21.5 min.

2-(*tert*-Butyldimethylsilyl)-2-hydroxyhept-6-enenitrile (2o).

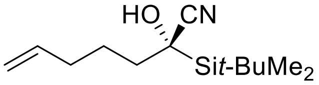

Yield: 65% yield (31 mg), 88% *ee*, colorless oil. $[\alpha]_D^{20}$ –19.1 (*c* 1.04, $CHCl_3$). ^1H NMR ($CDCl_3$) δ 5.81 (ddt, *J* = 17.0, 10.0, 7.5 Hz, 1H), 5.06 (ddt, *J* = 17.0, 1.5, 1.5 Hz, 1H), 5.01 (ddt, *J* = 10.0, 1.5, 3.5 Hz, 1H), 2.16 (m, 2H), 1.98 (s, 1H), 1.81–1.69 (m, 4H), 1.05 (s, 9H), 0.19 (s, 3H), 0.18 (s, 3H). ^{13}C NMR ($CDCl_3$) δ 137.6, 122.3, 115.5, 64.5, 36.1, 33.3, 27.3, 22.9, 18.2, –7.66, –8.17. TLC: R_f 0.13 (hexane/EtOAc = 20:1). IR (neat): 3427, 2932, 2219, 1648, 1476, 1254, 913, 839, 737, 525 cm^{-1}. HRMS (ESI) Calcd for $C_{13}H_{25}NOSiNa$: $[M+Na]^+$, 262.1598. Found: *m*/*z* 262.1601.

1-(*tert*-Butyldimethylsilyl)-1-cyanohex-5-en-1-yl tosylcarbamate (2o').

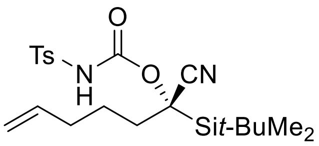

Yield: 21% yield (12 mg), 88% *ee*, white solid. $[\alpha]_D^{20}$ –31.0 (*c* 0.40, $CHCl_3$). ^1H NMR ($CDCl_3$) δ 9.16 (s, 1H), 7.96 (d, *J* = 8.0 Hz, 2H), 7.37 (d, *J* = 8.0 Hz, 2H), 5.75 (ddt, *J* = 17.0, 10.0, 6.5 Hz, 1H), 5.02–4.94 (m, 2H), 2.46 (s, 3H), 2.00–1.92 (m, 4H), 1.11 (m, 2H), 0.89 (s, 9H), 0.12 (s, 3H), 0.03 (s, 3H). ^{13}C NMR ($CDCl_3$) δ 150.6,

146.9, 137.5, 134.0, 130.0, 128.4, 115.1, 86.8, 34.8, 33.1 (2C), 27.3, 21.8, 20.6, 18.2, –7.9, –8.0. Mp. 64.5–65.0 °C. TLC: R_f 0.38 (hexane/EtOAc = 3:1). IR (KBr): 3329, 2936, 1796, 1683, 1311, 1176, 1086, 878, 835, 575 cm^{-1}. HRMS (ESI) Calcd for $C_{21}H_{32}N_2O_4SSiNa$: $[M+Na]^+$, 459.1744. Found: *m*/*z* 459.1738. HPLC (Daicel Chiralpak IA, hexane/*i*-PrOH = 97/3, flow rate = 1.0 mL/min, λ = 220 nm, 30 °C): t_{major} = 8.4 min, t_{minor} = 9.5 min.

2-(*tert*-Butyldimethylsilyl)-2-hydroxyhex-5-ynenitrile (2p).

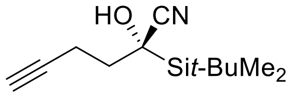

Yield: 86% yield (38 mg), 90% *ee*, colorless oil. $[\alpha]_D^{20}$ +10.3 (*c* 1.28, $CHCl_3$). ^1H NMR ($CDCl_3$) δ 3.12 (s, 1H), 2.69 (dddd, *J* = 14.5, 7.5, 4.5, 3.0 Hz, 1H), 2.53 (dddd, *J* = 14.5, 7.5, 4.5, 3.0 Hz, 1H), 2.14 (dd, *J* = 3.0, 3.0 Hz, 1H), 2.12 (ddd, *J* = 14.5, 7.5, 4.5 Hz, 1H), 1.95 (ddd, *J* = 14.5, 7.5, 4.5 Hz, 1H), 1.06 (s, 9H), 0.190 (s, 3H), 0.187 (s, 3H). ^{13}C NMR ($CDCl_3$) δ 121.5, 83.4, 71.0, 65.4, 34.4, 27.3, 18.2, 14.4, –7.8, –8.3. TLC: R_f 0.13 (hexane/EtOAc = 20:1). IR (neat): 3434, 2934, 2220, 1471, 1367, 1255, 1066, 840, 672, 462 cm^{-1}. HRMS (ESI) Calcd for $C_{12}H_{21}NOSiNa$: $[M+Na]^+$, 246.1285. Found: *m*/*z* 246.1284.

1-(*tert*-Butyldimethylsilyl)-1-cyanopent-4-yn-1-yl tosylcarbamate (2p').

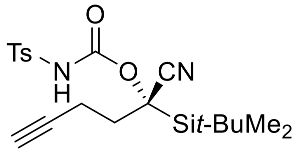

Yield: 25% yield (18 mg), 90% *ee*, white solid. $[\alpha]_D^{20}$ –37.0 (*c* 0.61, $CHCl_3$). ^1H NMR ($CDCl_3$) δ 9.19 (s, 1H), 7.96 (d, *J* = 8.0 Hz, 2H), 7.38 (d, *J* = 8.0 Hz, 2H), 2.47 (s, 3H), 2.30–2.27 (m, 2H), 2.13 (m, 1H), 2.02 (m, 1H), 1.76 (dd, *J* = 2.5, 2.5 Hz, 1H), 0.91 (s, 9H), 0.14 (s, 3H), 0.03 (s, 3H). ^{13}C NMR ($CDCl_3$) δ 156.6, 150.3, 147.0, 133.8, 130.0, 128.6, 85.9, 81.8, 69.5, 33.5, 27.3, 21.8, 18.3, 12.0, –8.05, –8.14. Mp. 72.5–73.0 °C. TLC: R_f 0.25 (hexane/EtOAc = 5:1). IR (KBr): 3337, 2959, 1793, 1685, 1381, 1302, 1164, 1095, 827, 572 cm^{-1}. HRMS (ESI) Calcd for $C_{20}H_{28}N_2O_4SSiNa$: $[M+Na]^+$, 443.1431. Found: *m*/*z* 443.1433. HPLC (Daicel Chiralpak IC, hexane/*i*-PrOH = 97/3, flow rate = 1.0 mL/min, λ = 230 nm, 30 °C): t_{major} = 14.9 min, t_{minor} = 16.9 min.

2-(*tert*-Butyldimethylsilyl)-2-hydroxy-6-oxoheptanenitrile (2q); 2-(*tert*-butyldimethylsilyl)-6-hydroxy-6-methyltetrahydro-2*H*-pyran-2-carbonitrile (34).

There is an equilibrium between **2q** and **34**.

Yield: 76% yield (39 mg), 88% *ee*, colorless oil. $[\alpha]_D^{20}$ –8.0 (*c* 1.18, $CHCl_3$). TLC: R_f 0.50 (hexane/EtOAc = 2:1). IR (neat): 3427, 2939, 2253, 2215, 1712, 1465, 1253, 911, 736, 469 cm^{-1}. HRMS (ESI) Calcd for $C_{13}H_{25}NO_2SiNa$: $[M+Na]^+$, 278.1547. Found: *m*/*z* 278.1544.

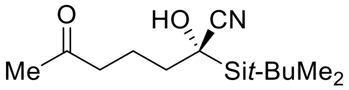

2q. ^{1}H NMR ($CDCl_3$) δ 3.37 (s, 1H), 2.62 (m, 2H), 2.19 (s, 3H), 1.96–1.71 (m, 4H), 1.04 (s, 9H), 0.160 (s, 3H), 0.156 (s, 3H). ^{13}C NMR ($CDCl_3$) δ 209.9, 122.7, 53.1, 42.7, 35.8, 29.9, 27.5, 18.2, 16.2, –7.8, –8.2.

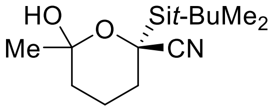

34. ^{1}H NMR ($CDCl_3$) δ 2.54 (s, 1H), 1.96–1.73 (m, 6H), 1.37 (s, 3H), 1.03 (s, 9H), 0.15 (s, 3H), 0.14 (s, 3H). ^{13}C NMR ($CDCl_3$) δ 123.3, 96.7, 62.4, 34.3, 30.7, 29.9, 27.4, 18.4, 17.3, –8.1, –8.3.

1-(*tert*-Butyldimethylsilyl)-1-cyano-5-oxohexyl tosylcarbamate (2q').

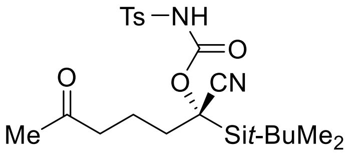

Yield: 39% yield (36 mg), 88% *ee*, colorless oil. $[\alpha]_D^{20}$ –25.1 (*c* 1.19, $CHCl_3$). ^{1}H NMR ($CDCl_3$) δ 9.18 (s, 1H), 7.96 (d, *J* = 8.0 Hz, 2H), 7.39 (d, *J* = 8.0 Hz, 2H), 2.47 (s, 3H), 2.34 (m, 2H), 2.06 (s, 3H), 1.99 (m, 2H), 1.34 (m, 2H), 0.89 (s, 9H), 0.11 (s, 3H), 0.01 (s, 3H). ^{13}C NMR ($CDCl_3$) δ 207.2, 157.2, 150.5, 147.0, 133.9, 130.1, 128.5, 86.7, 42.8, 34.5, 29.7, 27.3, 21.8, 18.2, 16.1, –7.9, –8.0. TLC: R_f 0.25 (hexane/EtOAc = 2:1). IR (neat): 3328, 2933, 1794, 1683, 1315, 1090, 841, 752, 680, 481 cm^{-1}. HRMS (ESI) Calcd for $C_{21}H_{32}N_2O_5SSiNa$: $[M+Na]^+$, 475.1693. Found: *m*/*z* 475.1685. HPLC (Daicel Chiralpak IA, hexane/*i*-PrOH = 60/40, flow rate = 2.0 mL/min, λ = 230 nm, 30 °C): t_{major} = 2.5 min, t_{minor} = 2.8 min.

2-(*tert*-Butyldimethylsilyl)-2-hydroxy-8-oxononanenitrile (2r).

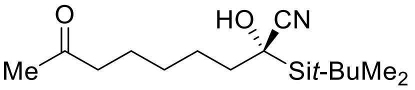

Yield: 70% yield (40 mg), 85% *ee*, colorless oil. $[\alpha]_D^{20}$ –11.4 (*c* 0.61, $CHCl_3$). ^1H NMR ($CDCl_3$) δ 2.46 (t, *J* = 7.0 Hz, 2H), 2.15 (s, 3H), 2.05 (s, 1H), 1.76 (m, 2H), 1.66–1.57 (m, 4H), 1.38 (m, 2H), 1.05 (s, 9H), 0.18 (s, 3H), 0.17 (s, 3H). ^{13}C NMR ($CDCl_3$) δ 208.9, 122.3, 64.5, 43.4, 36.4, 29.9, 28.9, 27.3, 23.5, 23.4, 18.2, –7.7, –8.2. TLC: R_f 0.13 (hexane/EtOAc = 5:1). IR (neat): 3449, 2954, 1706, 1466, 1366, 1252, 1160, 839, 733, 510 cm^{-1}. HRMS (ESI) Calcd for $C_{15}H_{29}NO_2SiNa$: $[M+Na]^+$, 306.1860. Found: *m*/*z* 306.1858.

1-(*tert*-Butyldimethylsilyl)-1-cyano-7-oxooctyl tosylcarbamate (2r').

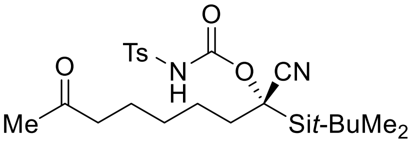

Yield: 88% yield (4.2 mg), 85% *ee*, colorless oil. $[\alpha]_D^{20}$ –21.9 (*c* 1.40, $CHCl_3$). ^1H NMR ($CDCl_3$) δ 9.14 (s, 1H), 7.95 (d, *J* = 8.0 Hz, 2H), 7.37 (d, *J* = 8.0 Hz, 2H), 2.46 (s, 3H), 2.33 (t, *J* = 7.0 Hz, 2H), 2.10 (s, 3H), 1.94 (t, *J* = 8.5 Hz, 2H), 1.38 (m, 2H), 1.17 (m, 2H), 1.07 (m, 1H), 0.93 (m, 1H), 0.87 (s, 9H), 0.10 (s, 3H), 0.01 (s, 3H). ^{13}C NMR ($CDCl_3$) δ 208.6, 157.4, 150.6, 147.0, 133.9, 130.0, 128.4, 86.8, 43.3, 35.2, 29.9, 28.7, 27.3, 23.4, 21.8, 21.2, 18.1, –7.9, –8.0. TLC: R_f 0.25 (hexane/EtOAc = 3:1). IR (neat): 3328, 2931, 1799, 1687, 1312, 1191, 1090, 839, 755, 487 cm^{-1}. HRMS (ESI) Calcd for $C_{23}H_{37}N_2O_5SSi$: $[M+H]^+$, 481.2187. Found: *m*/*z* 481.2196. HPLC (Daicel Chiralpak IA, hexane/*i*-PrOH = 60/40, flow rate = 2.0 mL/min, λ = 230 nm, 30 °C): t_{minor} = 2.5 min, t_{major} = 2.9 min.

(*E*)-2-(*tert*-Butyldimethylsilyl)-2-hydroxy-8-oxo-8-phenyloct-6-enenitrile (2s): CAS RN [2307487-25-6].

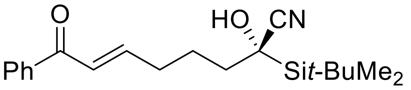

Yield: 88% yield (60 mg), 82% *ee*, white solid. ^1H NMR (C_6D_6) δ 7.94 (m, 2H), 7.13–7.08 (m, 3H), 7.00 (m, 1H), 6.71 (m, 1H), 1.80 (m, 2H), 1.53–1.46 (m, 3H), 1.34 (m, 1H), 0.99 (s, 9H), 0.04 (s, 3H), 0.00 (s, 3H). ^{13}C NMR (C_6D_6) δ 189.7, 147.9, 138.7, 132.8, 129.0, 128.9, 126.7, 122.4, 64.4, 36.9, 32.4, 27.7, 22.7, 18.5, –7.4, –7.9. TLC: R_f 0.25 (hexane/EtOAc = 5:1). HPLC (Daicel Chiralpak IA, hexane/*i*-PrOH =

90/10, flow rate = 1.0 mL/min, λ = 254 nm, 30 °C): t_{major} = 7.7 min, t_{minor} = 8.7 min.

2-(*tert*-Butyldimethylsilyl)-2-hydroxy-2-phenylacetonitrile (2t).

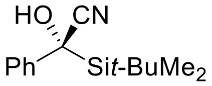

Yield: 50% yield (25 mg), 74% *ee*, white solid. $[\alpha]_D^{20}$ –3.7 (*c* 0.54, $CHCl_3$). ^1H NMR ($CDCl_3$) δ 7.43 (d, *J* = 7.0 Hz, 2H), 7.39 (dd, *J* = 7.5, 7.0 Hz, 2H), 7.30 (t, *J* = 7.5 Hz, 1H), 0.97 (s, 9H), 0.13 (s, 3H), 0.06 (s, 3H). ^{13}C NMR ($CDCl_3$) δ 139.2, 128.5, 127.7, 124.6, 122.1, 68.3, 27.1, 18.6, –7.9, –8.2. Mp. 84.5–85.0 °C. TLC: R_f 0.25 (hexane/EtOAc = 10:1). IR (KBr): 3364, 2930, 2226, 1465, 1392, 1254, 1040, 837, 615, 502 cm^{-1}. HRMS (ESI) Calcd for $C_{14}H_{21}NOSiNa$: $[M+Na]^+$, 270.1285. Found: *m*/*z* 270.1282. HPLC (Daicel Chiralpak IC, hexane/*i*-PrOH = 90/10, flow rate = 0.5 mL/min, λ = 230 nm, 30 °C): t_{minor} = 7.5 min, t_{major} = 8.1 min.

(*E*)-2-(*tert*-Butyldimethylsilyl)-2-hydroxy-6-phenylhex-3-enenitrile (2u).

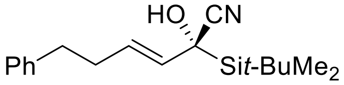

Yield: 41% yield (25 mg), 37% *ee*, colorless oil. $[\alpha]_D^{20}$ +6.4 (*c* 0.83, $CHCl_3$). ^1H NMR ($CDCl_3$) δ 7.29 (dd, *J* = 7.5, 7.5 Hz, 2H), 7.21–7.17 (m, 3H), 5.89 (dt, *J* = 15.0, 7.0 Hz, 1H), 5.58 (d, *J* = 15.0 Hz, 1H), 2.74 (t, *J* = 7.0 Hz, 2H), 2.46 (dt, *J* = 7.0, 7.0 Hz, 2H), 1.01 (s, 9H), 0.10 (s, 6H). ^{13}C NMR ($CDCl_3$) δ 141.0, 130.6, 128.42, 128.37, 128.30, 126.1, 121.1, 65.9, 35.2, 33.7, 27.2, 18.3, –8.1, –8.5. TLC: R_f 0.25 (hexane/EtOAc = 10:1). IR (neat): 3404, 2931, 2860, 2222, 1471, 1252, 970, 910, 756, 473 cm^{-1}. HRMS (ESI) Calcd for $C_{18}H_{27}NOSiNa$: $[M+Na]^+$, 324.1754. Found: *m*/*z* 324.1757. HPLC (Daicel Chiralpak IB, hexane/*i*-PrOH = 98/2, flow rate = 0.5 mL/min, λ = 220 nm, 30 °C): t_{minor} = 15.5 min, t_{major} = 17.0 min.

2-(*tert*-Butyldimethylsilyl)-5-phenyl-2-((trimethylsilyl)oxy)pentanenitrile (5a).

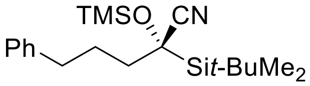

Yield: 99% yield (72 mg), 92% *ee*, colorless oil. $[\alpha]_D^{20}$ –11.3 (*c* 0.79, $CHCl_3$). ^1H NMR ($CDCl_3$) δ 7.29 (dd, *J* = 7.5, 6.0 Hz, 2H), 7.21–7.17 (m, 3H), 2.66 (t, *J* = 7.0 Hz, 2H), 1.92–1.79 (m, 4H), 0.97 (s, 9H), 0.20 (s, 9H), 0.15 (s, 3H), 0.12 (s, 3H). ^{13}C NMR ($CDCl_3$) δ 141.3, 128.4, 128.3, 126.0, 122.3, 66.3, 38.0, 35.7, 27.5, 26.9, 18.4, 1.8, –6.9, –7.1. TLC: R_f 0.50 (hexane/EtOAc = 10:1). IR (neat): 2943, 2211, 1604, 1465, 1253, 1096, 1008, 846, 699, 451 cm^{-1}. HRMS (ESI) Calcd for $C_{20}H_{35}NOSi_2Na$:

[M+Na]$^+$, 384.2149. Found: *m*/*z* 384.2151.

Procedure for copper(I)-catalyzed azide-alkyne cycloaddition of 2p[11]

To a 5-mL vial were sequentially added copper(I) iodide (1.9 mg, 0.010 mmol), CH_2Cl_2 (0.10 mL), *N*,*N*-diisopropylethylamine (7.0 µL, 0.040 mmol), and AcOH (2.0 µL, 0.040 mmol). After the reaction mixture was stirred at ambient temperature for 15 min, a solution of **2p** (45 mg, 0.20 mmol) in CH_2Cl_2 (0.10 mL) and benzyl azide (30 µL, 0.24 mmol) was added. The mixture was stirred at ambient temperature for 2 h. The reaction mixture was subsequently diluted with EtOAc, passed through a short silica gel pad, and concentrated in vacuo. Purification of the crude product by flash silica gel column chromatography using hexane/EtOAc (v/v = 2:1) as an eluent afforded the corresponding triazole **4**.

4-(1-Benzyl-1*H*-1,2,3-triazol-4-yl)-2-(*tert*-butyldimethylsilyl)-2-hydroxybutanenitrile (4).

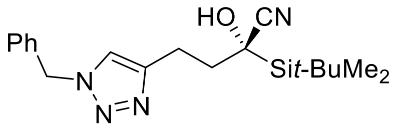

Yield: 85% yield (60 mg), 90% *ee*, colorless oil. $[\alpha]_D^{20}$ –9.4 (*c* 2.01, $CHCl_3$). ^{1}H NMR ($CDCl_3$) δ 7.41–7.36 (m, 3H), 7.27 (m, 2H), 6.25 (s, 1H), 5.50 (s, 2H), 3.17 (ddd, *J* = 16.0, 8.5, 6.0 Hz, 1H), 3.00 (ddd, *J* = 16.0, 6.0, 4.5 Hz, 1H), 2.17 (m, 2H), 1.07 (s, 9H), 0.20 (s, 3H), 0.17 (s, 3H). ^{13}C NMR ($CDCl_3$) δ 146.3, 134.3, 129.2, 128.9, 128.1, 122.5, 121.2, 63.8, 54.3, 34.2, 27.4, 22.2, 18.3, –7.6, –8.3. TLC: R_f 0.50 (hexane/EtOAc = 2:1). IR (neat): 3230, 2953, 2213, 1724, 1471, 1253, 1065, 839, 729, 474 cm^{-1}. HRMS (ESI) Calcd for $C_{19}H_{29}N_4OSi$: [M+H]$^+$, 357.2105. Found: *m*/*z* 357.2098. HPLC (Daicel Chiralpak IC, hexane/*i*-PrOH = 85/15, flow rate = 1.0 mL/min, λ = 210 nm, 30 °C): t_{major} = 13.1 min, t_{minor} = 16.1 min.

Procedure for hydration of acylsilane cyanohydrin 2a[12]

To a 5-mL vial were sequentially added palladium(II) nitrate (4.6 mg, 0.020 mmol), AcOH (0.40 mL), acetamide (47 mg, 0.80 mmol), and **2a** (58 g, 0.20 mmol) in a glovebox. The mixture was stirred at 50 °C for 1 h. The reaction mixture was subsequently diluted with EtOAc, passed through a short silica gel pad, and concentrated in vacuo. Purification of the crude product by flash silica gel column

chromatography using hexane/EtOAc (v/v = 4:1) as an eluent afforded the corresponding amide **8**.

2-(*tert*-Butyldimethylsilyl)-2-hydroxy-5-phenylpentanamide (8).

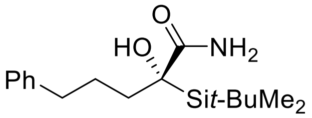

Yield: 76% yield (47 mg), 93% *ee*, white solid. $[\alpha]_D^{20}$ –18.1 (*c* 1.57, $CHCl_3$). ^1H NMR ($CDCl_3$) δ 7.27 (m, 2H), 7.19–7.16 (m, 3H), 6.16 (s, 1H), 5.45 (s, 1H), 2.61 (m, 2H), 2.24 (s, 1H), 2.13 (m, 1H), 1.79 (m, 2H), 1.53 (m, 1H), 0.91 (s, 9H), 0.12 (s, 3H), 0.11 (s, 3H). ^{13}C NMR ($CDCl_3$) δ 178.6, 142.1, 128.4, 128.3, 125.8, 75.0, 36.1, 35.9, 27.4, 24.3, 18.3, –6.9, –7.6. Mp. 52.5–53.0 °C. TLC: R_f 0.13 (hexane/EtOAc = 5:1). IR (KBr): 3464, 3155, 2950, 1655, 1581, 1401, 1249, 1084, 782, 578 cm^{-1}. HRMS (ESI) Calcd for $C_{17}H_{29}NO_2SiNa$: $[M+Na]^+$, 330.1860. Found: *m*/*z* 330.1865. HPLC (Daicel Chiralpak IB, hexane/*i*-PrOH = 95/5, flow rate = 1.0 mL/min, λ = 220 nm, 30 °C): t_{minor} = 10.5 min, t_{major} = 14.4 min.

Procedure for hydration of trimethylsilyl ether 5a[13]

To a 5-mL vial were sequentially added **5a** (72 mg, 0.20 mmol), indium(III) chloride (2.2 mg, 0.010 mmol), acetaldoxime (37 μL, 0.60 mmol), and toluene (0.20 mL) in a glovebox. The mixture was stirred at 60 °C for 24 h. The reaction mixture was subsequently diluted with EtOAc, passed through a short silica gel pad, and concentrated in vacuo. Purification of the crude product by flash silica gel column chromatography using hexane/EtOAc (v/v = 3:1) as an eluent afforded the corresponding amide **10**.

2-(*tert*-Butyldimethylsilyl)-5-phenyl-2-((trimethylsilyl)oxy)pentanamide (10).

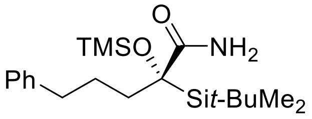

Yield: 15% yield (12 mg), 92% *ee*, white solid. $[\alpha]_D^{20}$ –42.8 (*c* 0.34, $CHCl_3$). ^1H NMR ($CDCl_3$) δ 7.27 (t, *J* = 8.0 Hz, 2H), 7.18–7.16 (m, 3H), 6.50 (s, 1H), 5.49 (s, 1H), 2.64 (m, 1H), 2.55 (m, 1H), 2.20 (m, 1H), 1.77–1.65 (m, 3H), 0.94 (s, 9H), 0.19 (s, 9H), 0.16 (s, 3H), 0.07 (s, 3H). ^{13}C NMR ($CDCl_3$) δ 178.8, 142.1, 128.3 (2C), 125.8, 80.9, 36.1, 35.4, 27.6, 25.9, 18.5, 2.8, –6.1, –7.0. Mp. 99.0–99.5 °C. TLC: R_f 0.45

(hexane/EtOAc = 3:1). IR (KBr): 3492, 3260, 2935, 1657, 1381, 1253, 1104, 838, 761, 528 cm^{-1}. HRMS (ESI) Calcd for $C_{20}H_{37}NO_2Si_2Na$: $[M+Na]^+$, 402.2255. Found: *m*/*z* 402.2258. HPLC (Daicel Chiralpak IB, hexane/*i*-PrOH = 99/1, flow rate = 2.0 mL/min, λ = 220 nm, 30 °C): t_{major} = 5.3 min, t_{minor} = 6.4 min.

NMR Spectra (^{1}H, ^{13}C) of Substrates

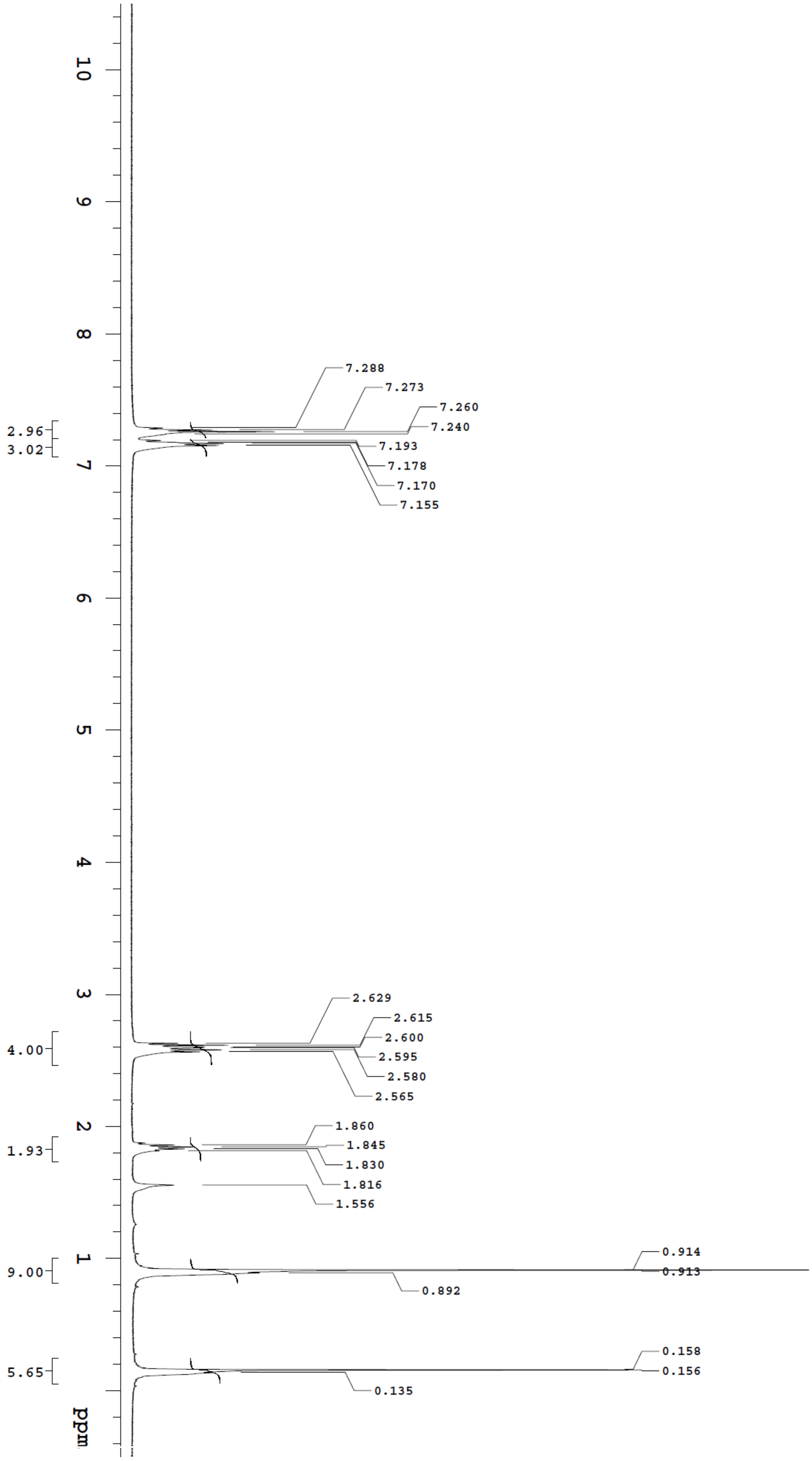

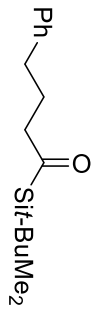

Supplementary Figure 1. ^{1}H NMR Spectrum of 1-(*tert*-Butyldimethylsilyl)-4-phenylbutan-1-one (**1a**)

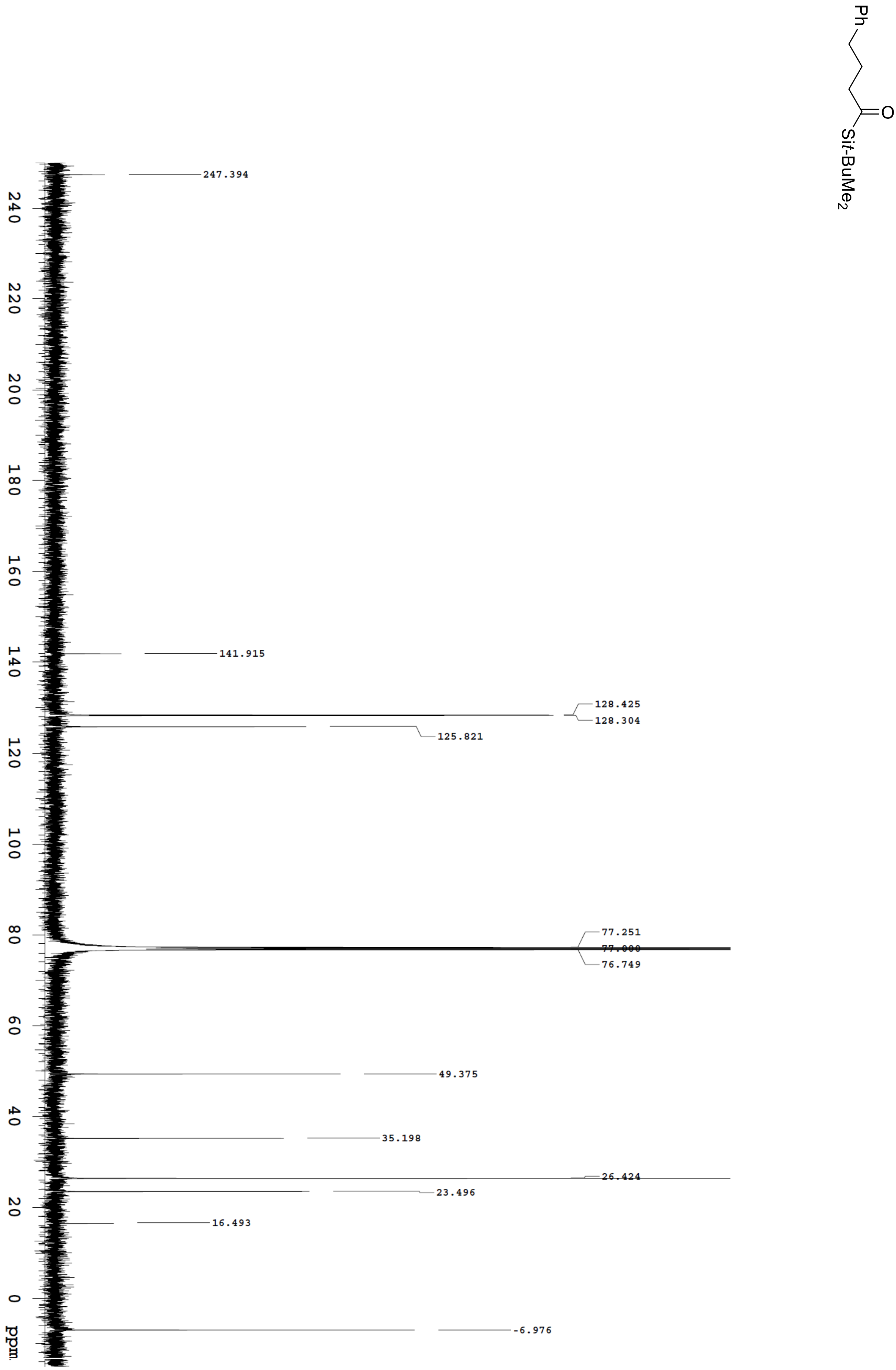

Supplementary Figure 2. ^{13}C NMR Spectrum of 1-(*tert*-Butyldimethylsilyl)-4-phenylbutan-1-one (**1a**)

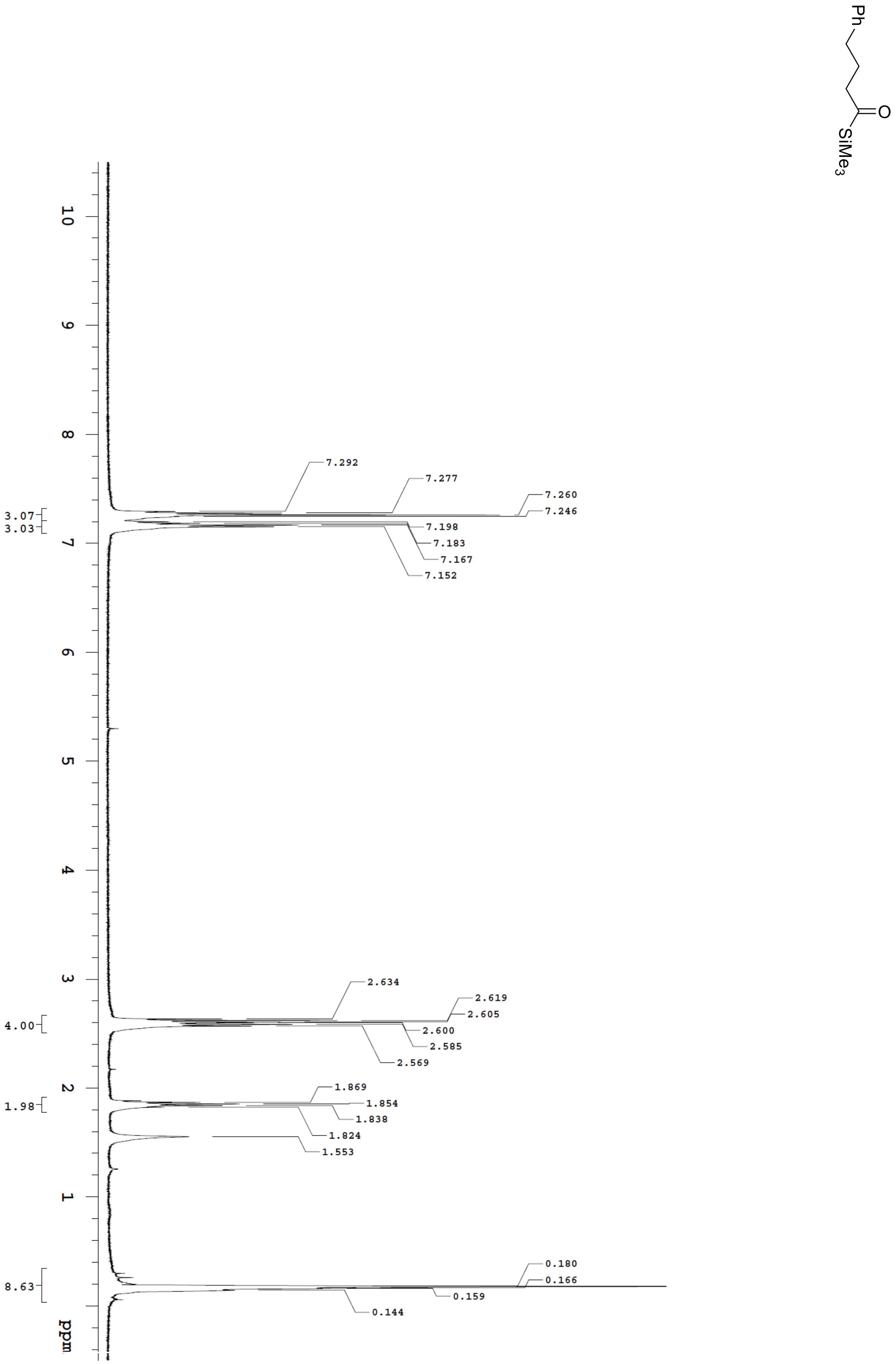

Supplementary Figure 3. ^{1}H NMR Spectrum of 4-Phenyl-1-(trimethylsilyl)butan-1-one (**1b**)

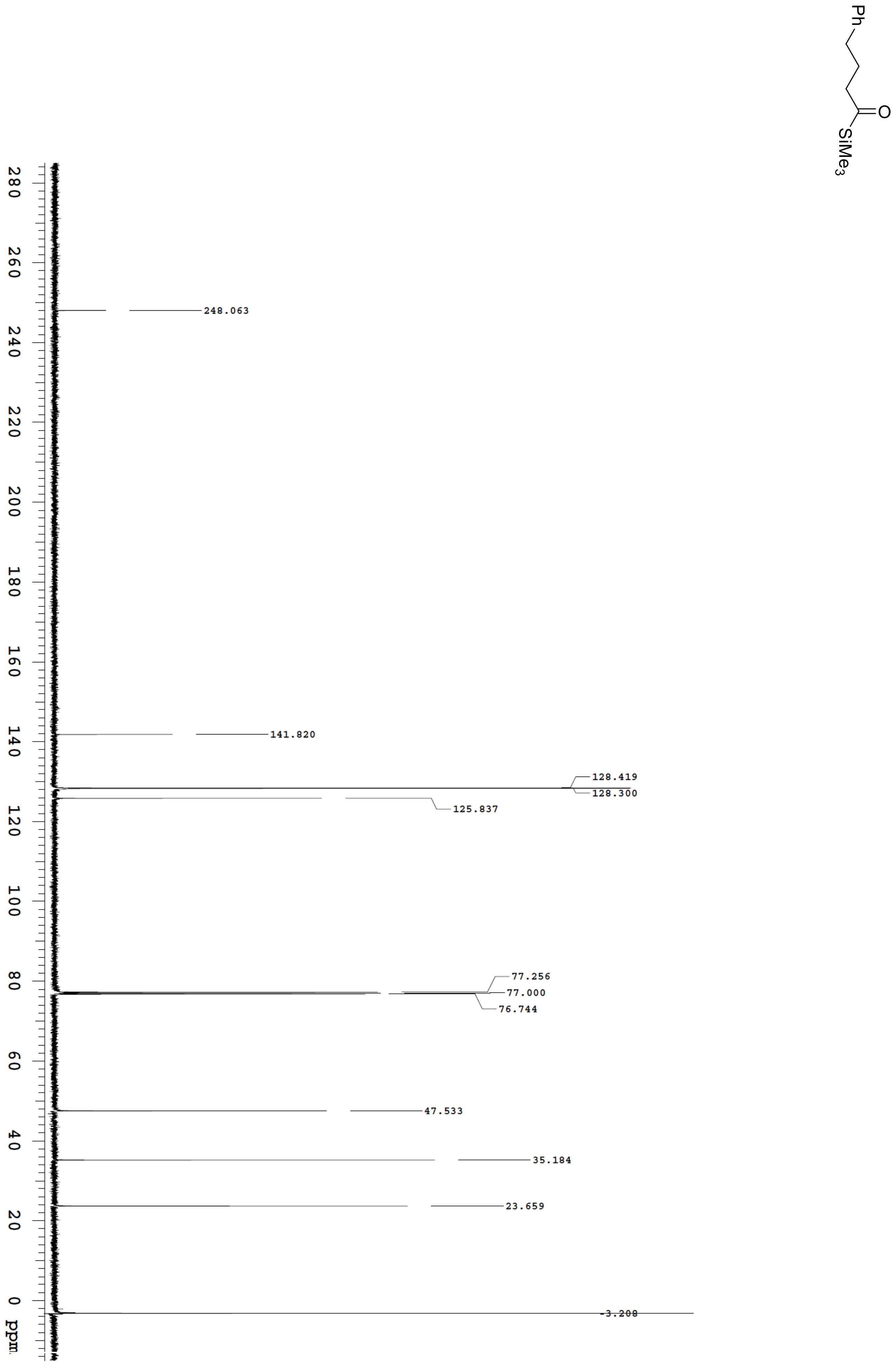

Supplementary Figure 4. ^{13}C NMR Spectrum of 4-Phenyl-1-(trimethylsilyl)butan-1-one (**1b**)

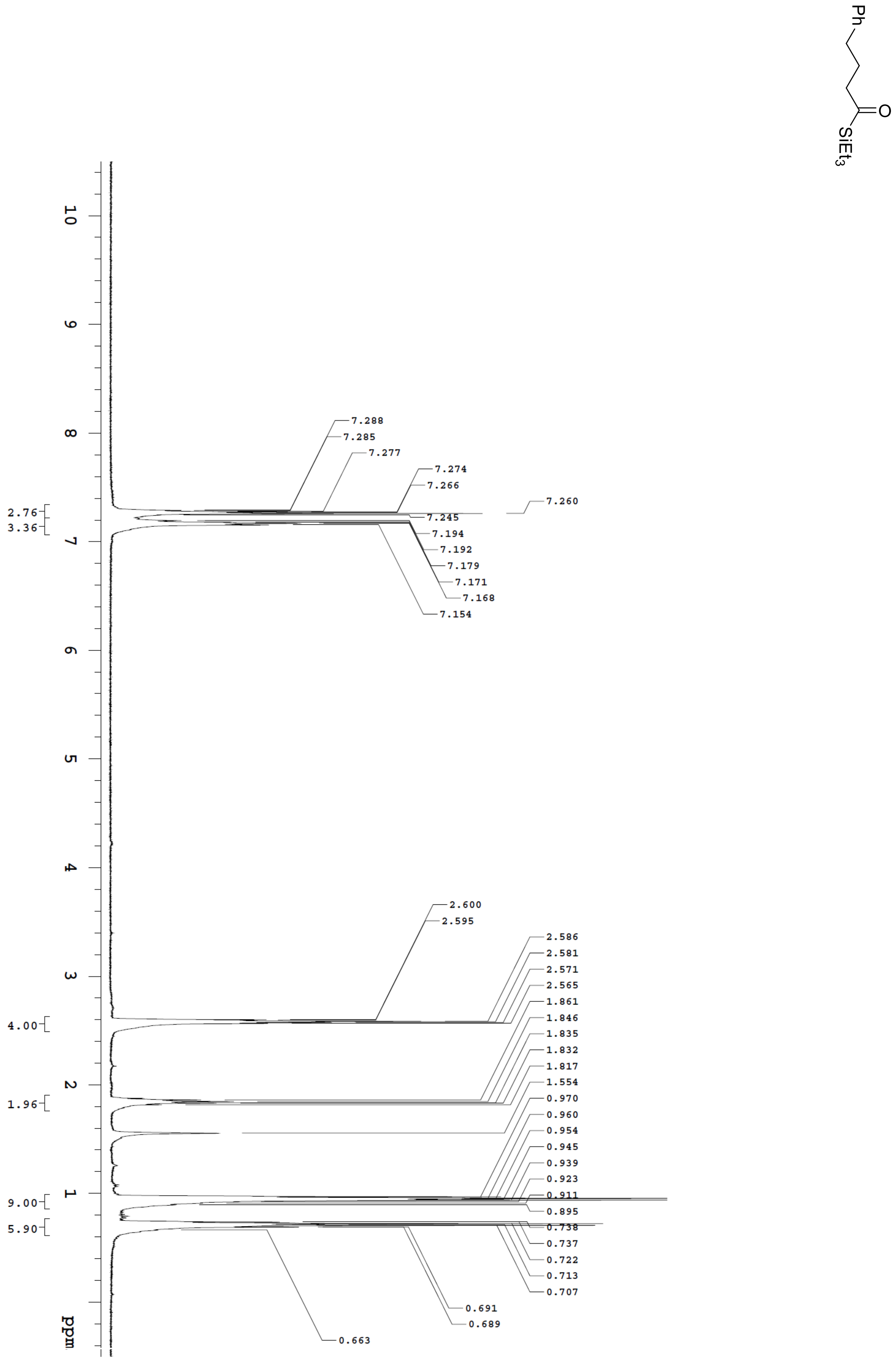

Supplementary Figure 5. ^{1}H NMR Spectrum of 4-Phenyl-1-(triethylsilyl)butan-1-one (**1c**)

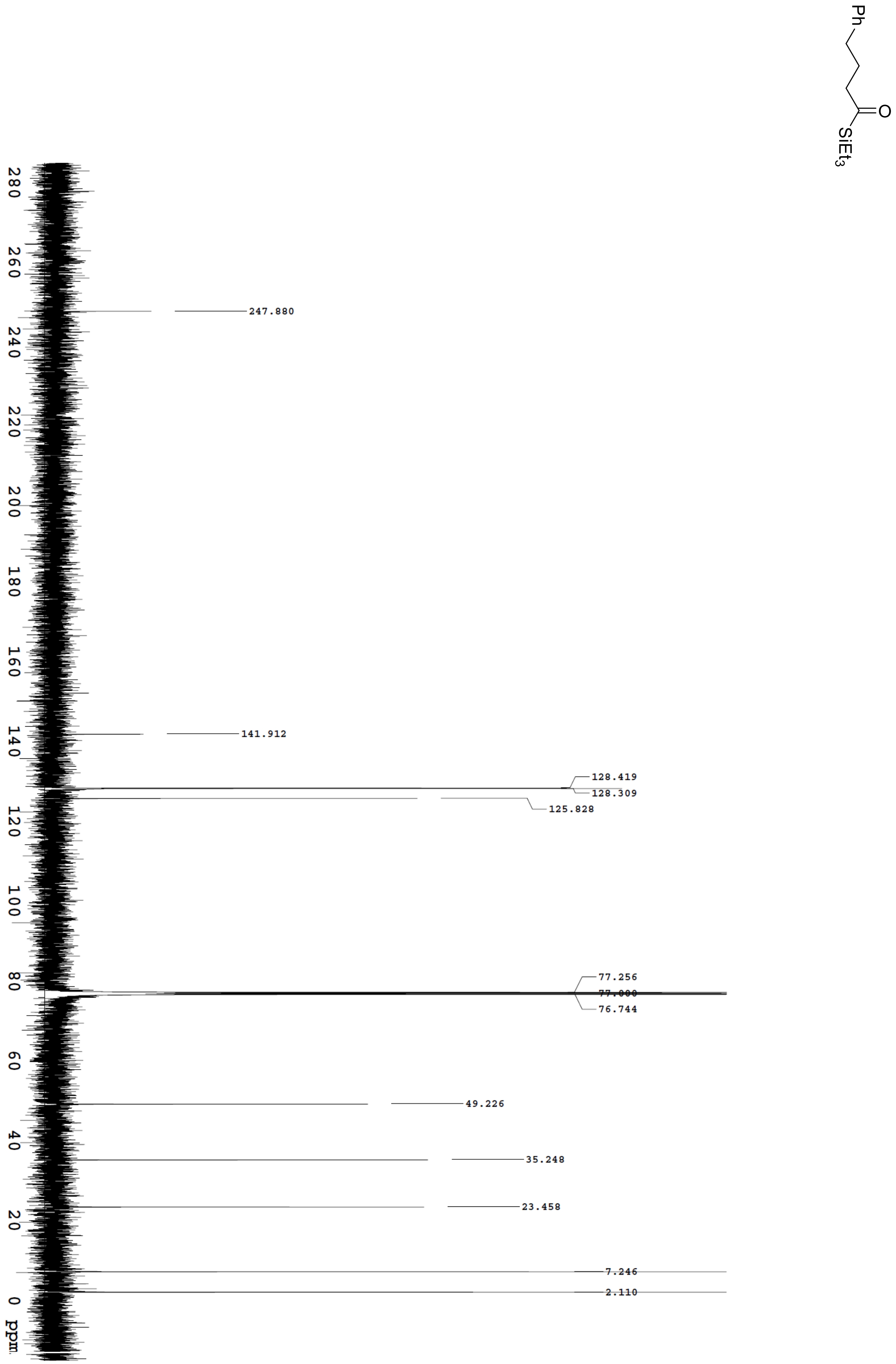

Supplementary Figure 6. ^{13}C NMR Spectrum of 4-Phenyl-1-(triethylsilyl)butan-1-one (**1c**)

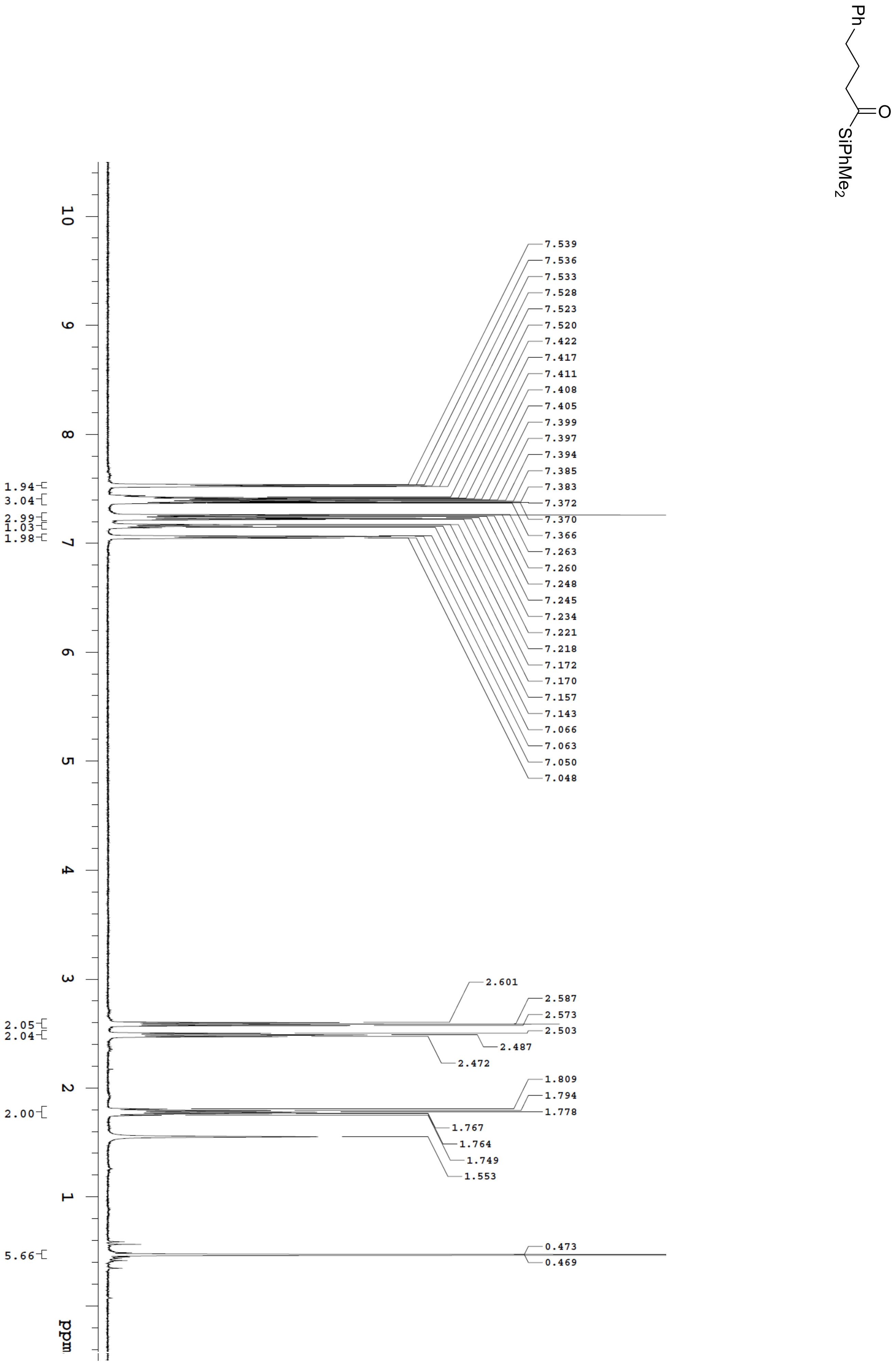

Supplementary Figure 7. ^{1}H NMR Spectrum of 1-(Dimethyl(phenyl)silyl)-4-phenylbutan-1-one (**1d**)

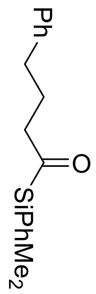

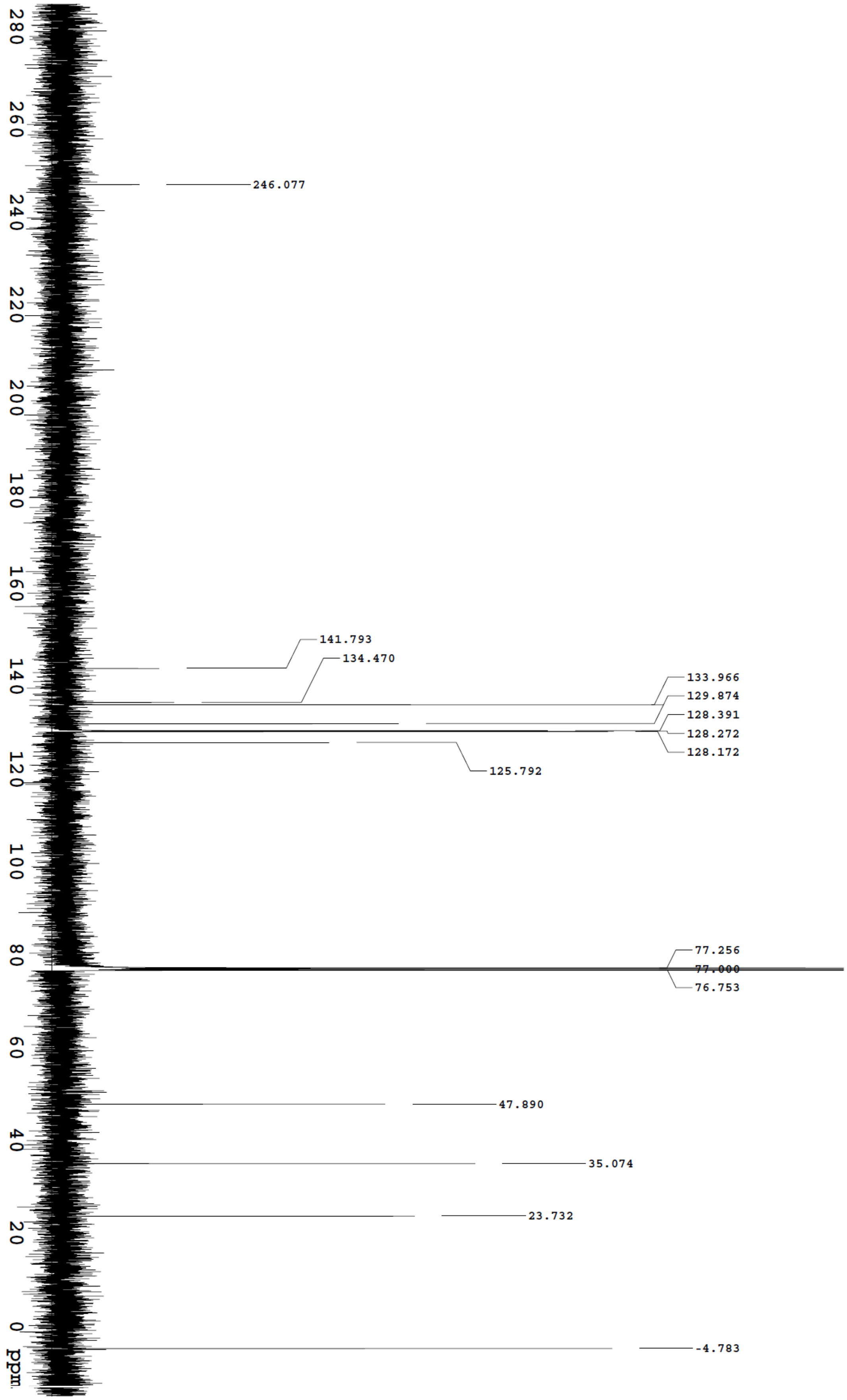

Supplementary Figure 8. ^{13}C NMR Spectrum of

1-(Dimethyl(phenyl)silyl)-4-phenylbutan-1-one (**1d**)

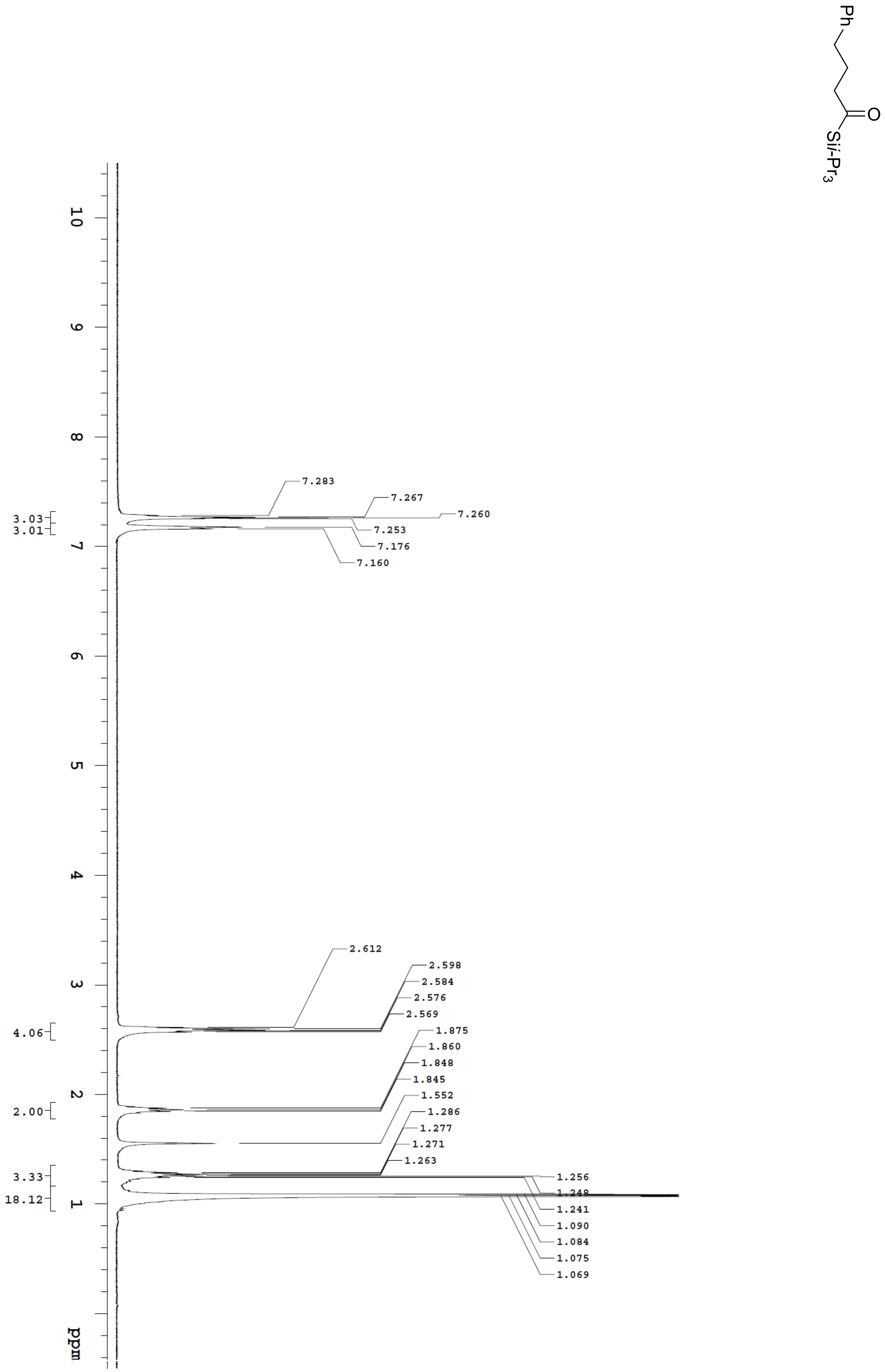

Supplementary Figure 9. ^{1}H NMR Spectrum of 4-Phenyl-1-(triisopropylsilyl)butan-1-one (**1e**)

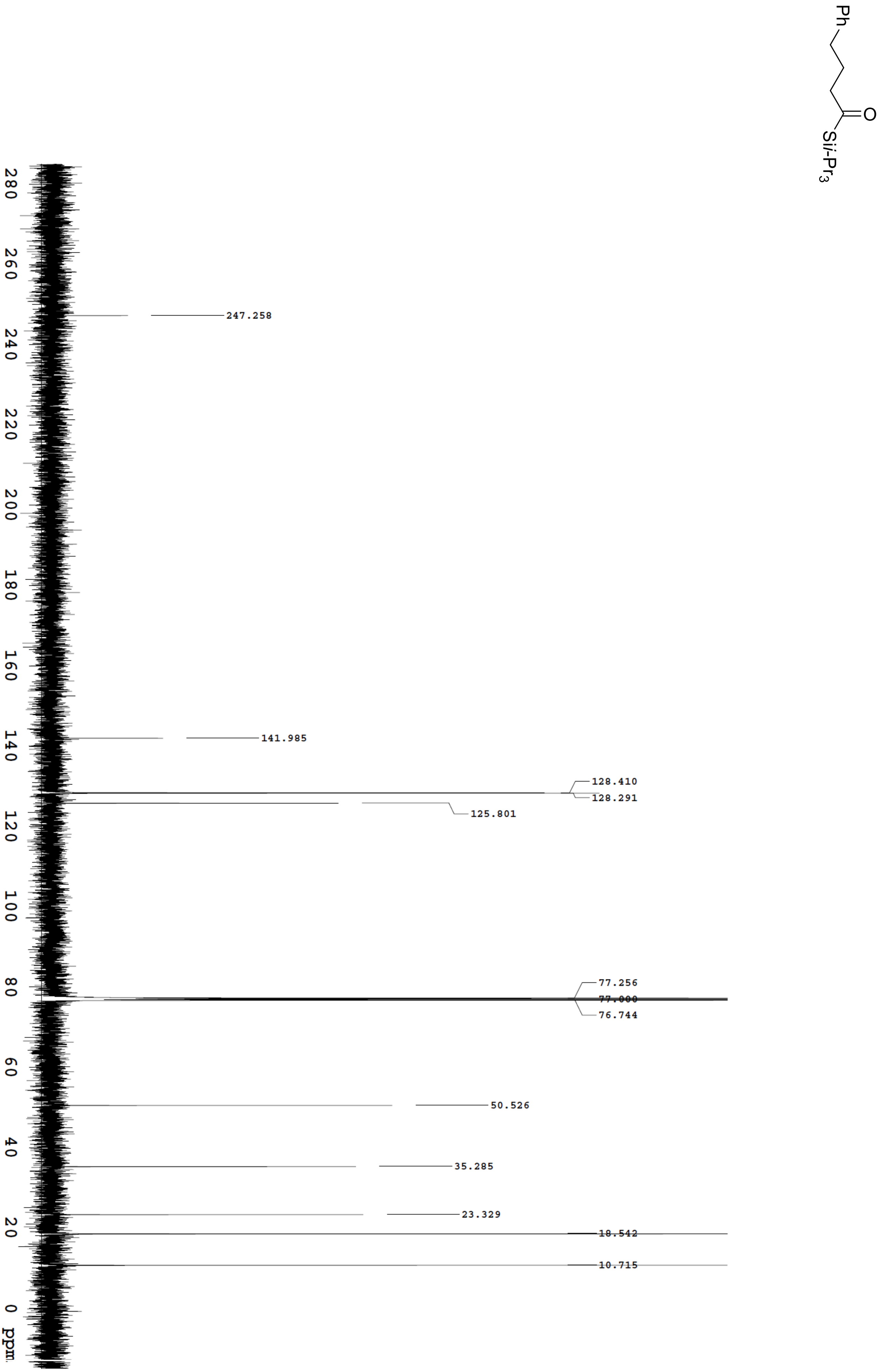

Supplementary Figure 10. ^{13}C NMR Spectrum of 4-Phenyl-1-(triisopropylsilyl)butan-1-one (**1e**)

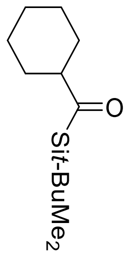

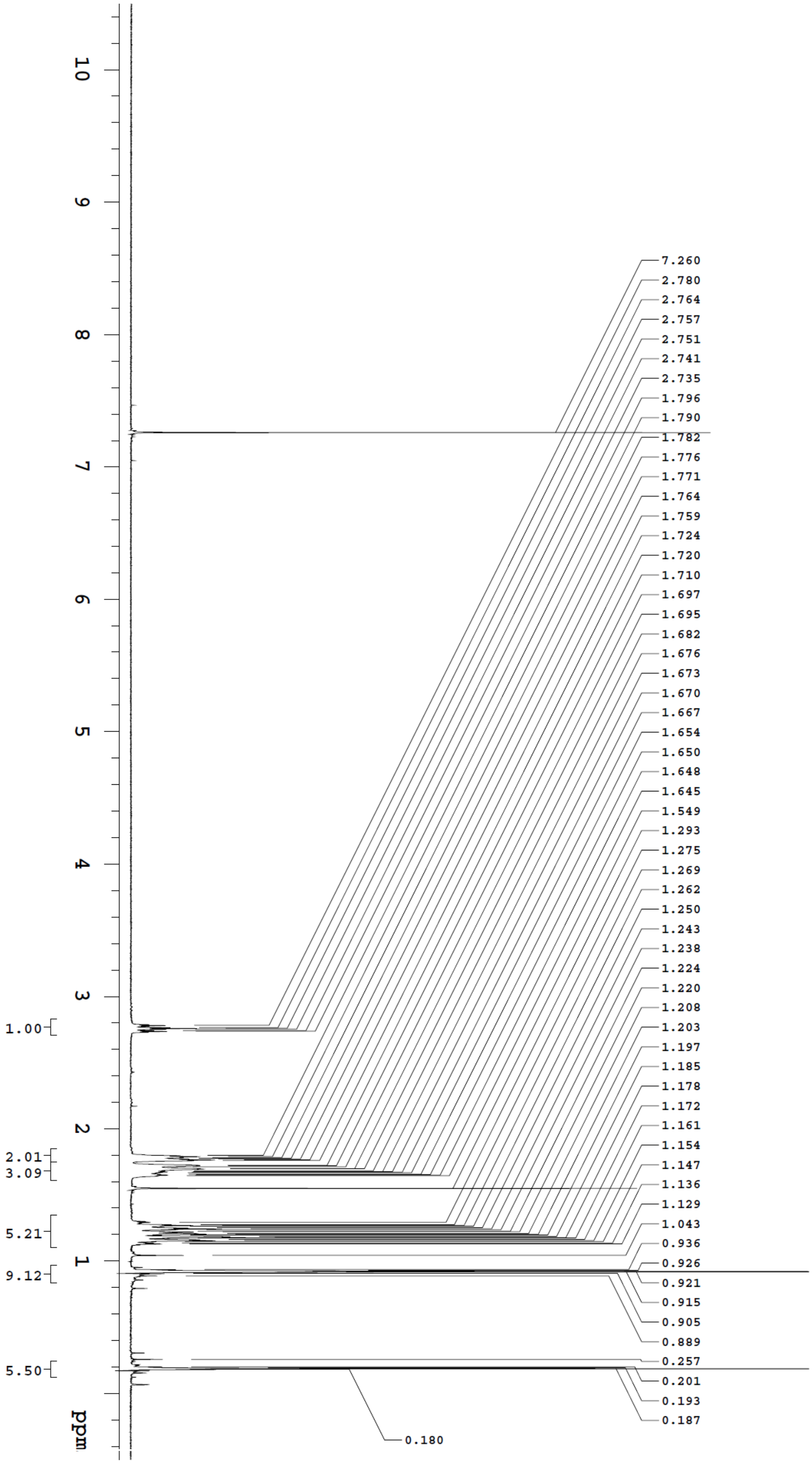

Supplementary Figure 11. ^{1}H NMR Spectrum of (*tert*-Butyldimethylsilyl)(cyclohexyl)methanone (**1f**)

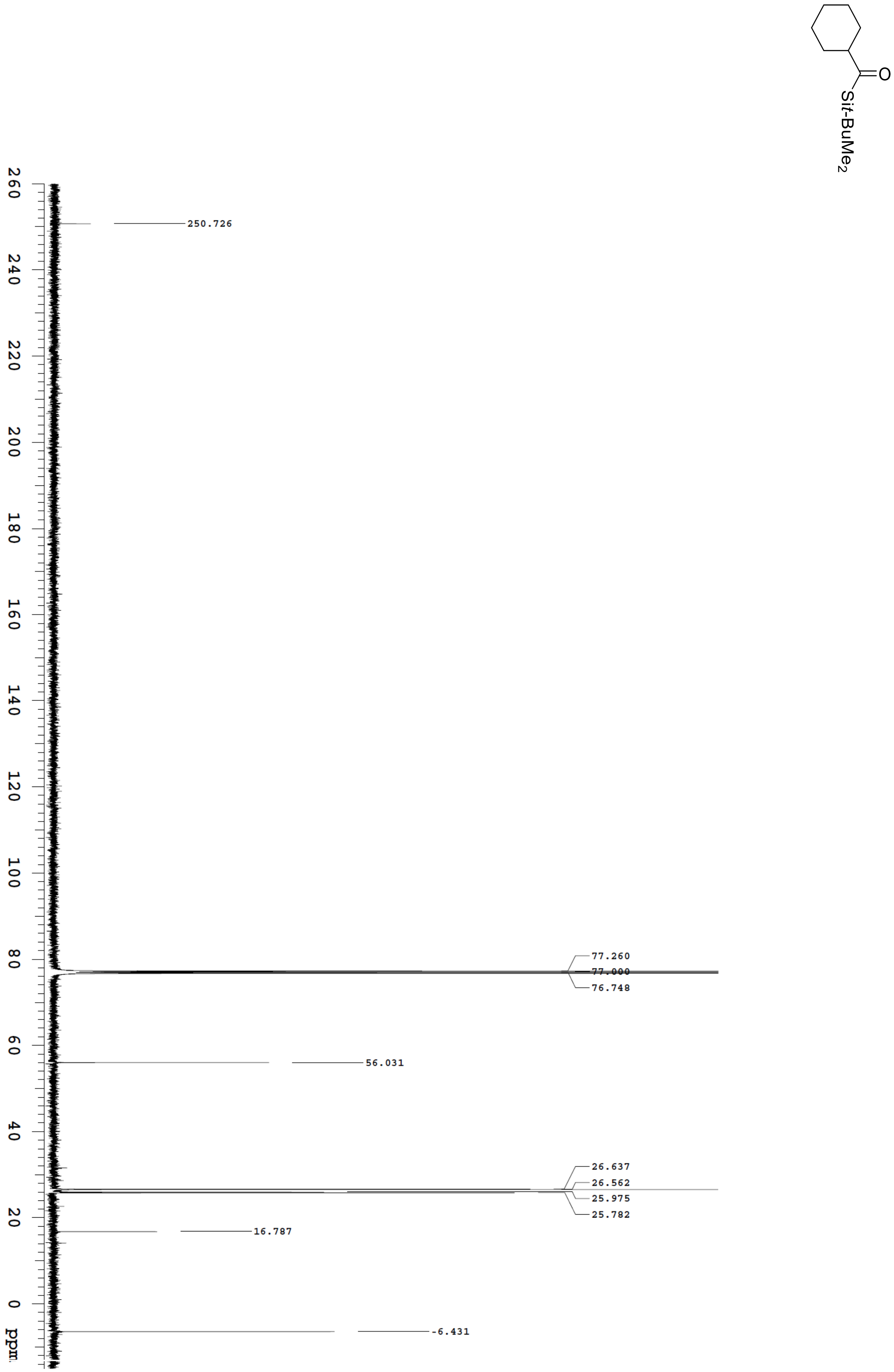

Supplementary Figure 12. ^{13}C NMR Spectrum of (*tert*-Butyldimethylsilyl)(cyclohexyl)methanone (**1f**)

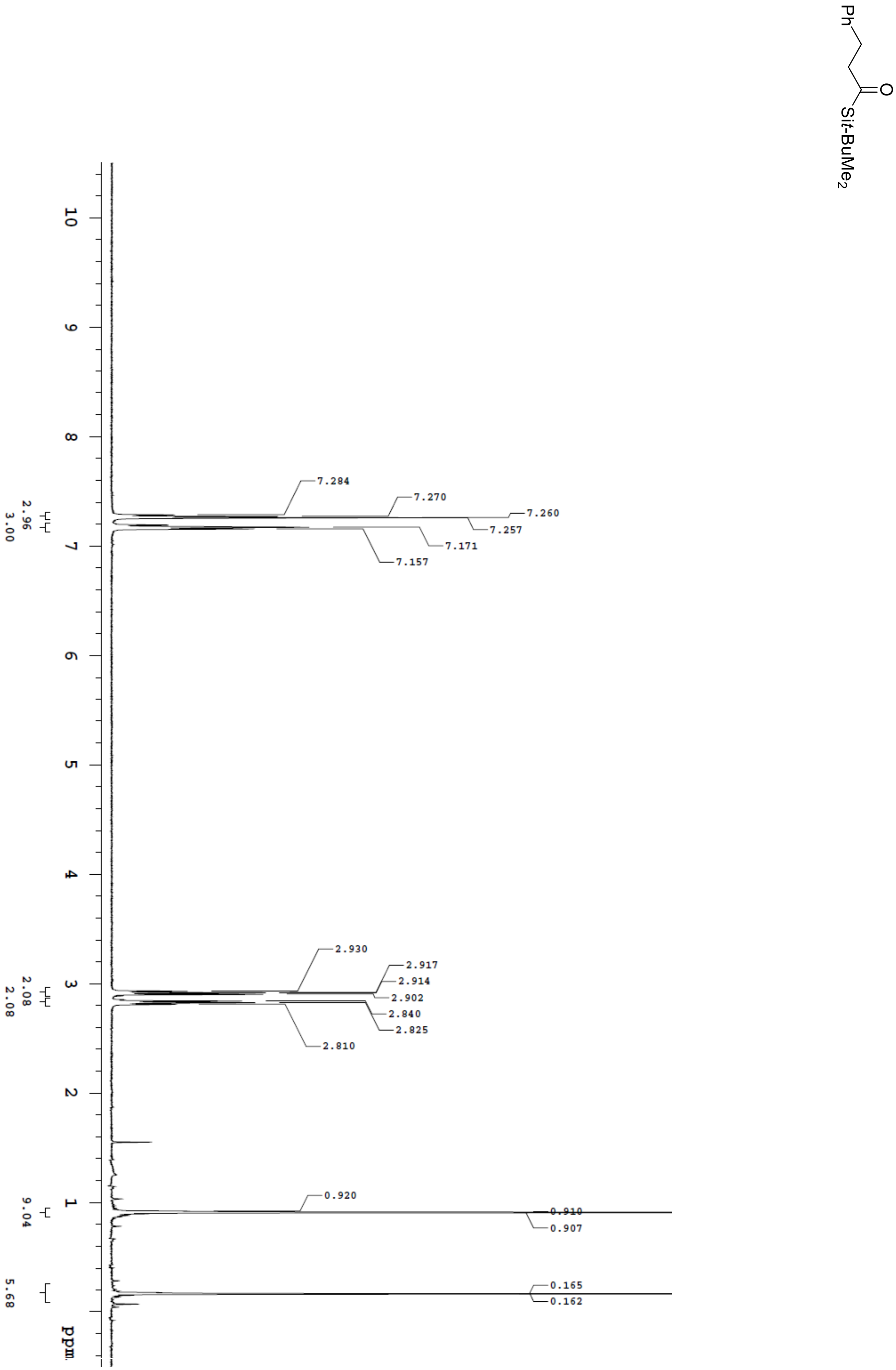

Supplementary Figure 13. ^{1}H NMR Spectrum of 1-(*tert*-Butyldimethylsilyl)-3-phenylpropan-1-one (**1g**)

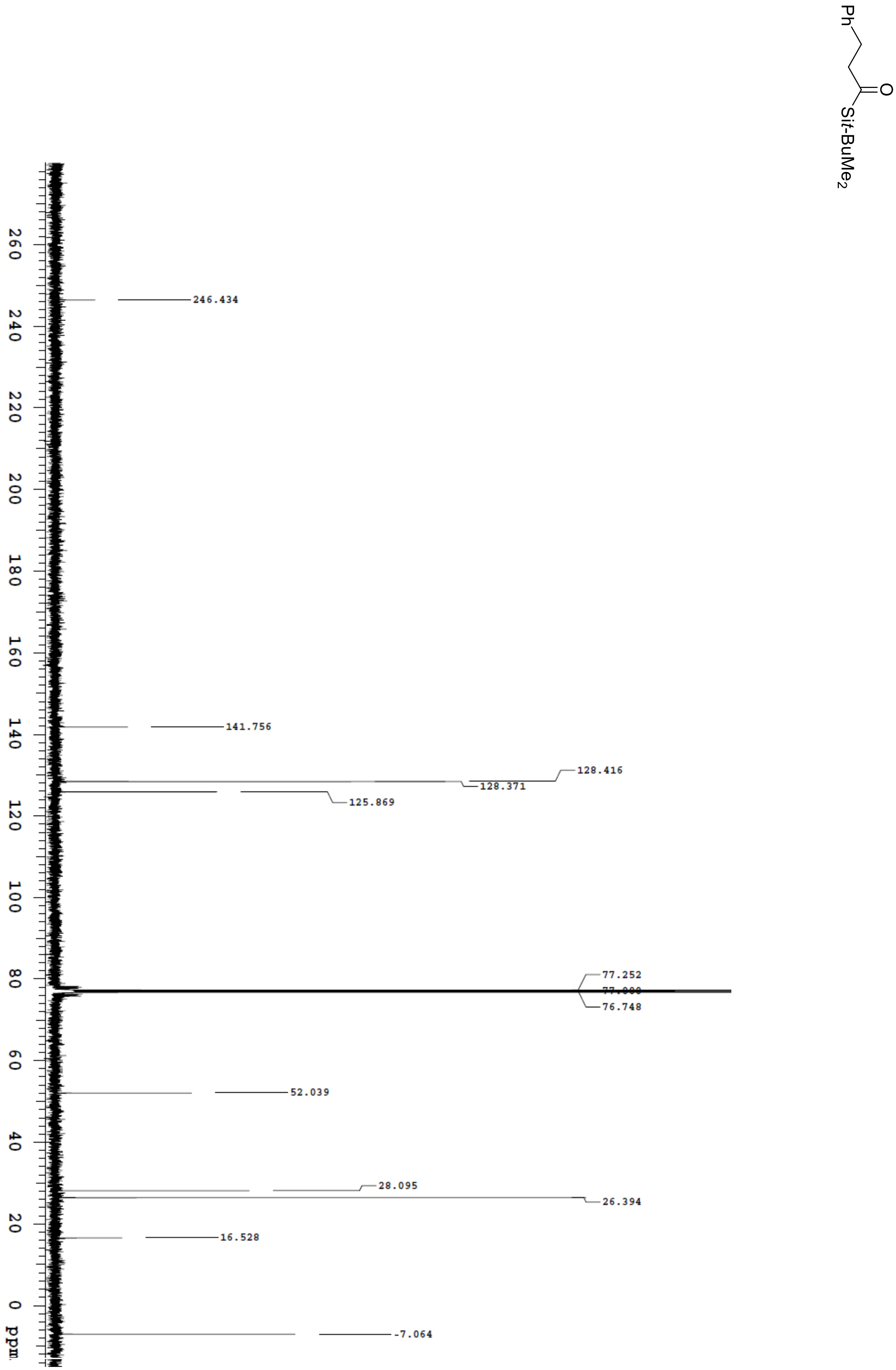

Supplementary Figure 14. ^{13}C NMR Spectrum of 1-(*tert*-Butyldimethylsilyl)-3-phenylpropan-1-one (**1g**)

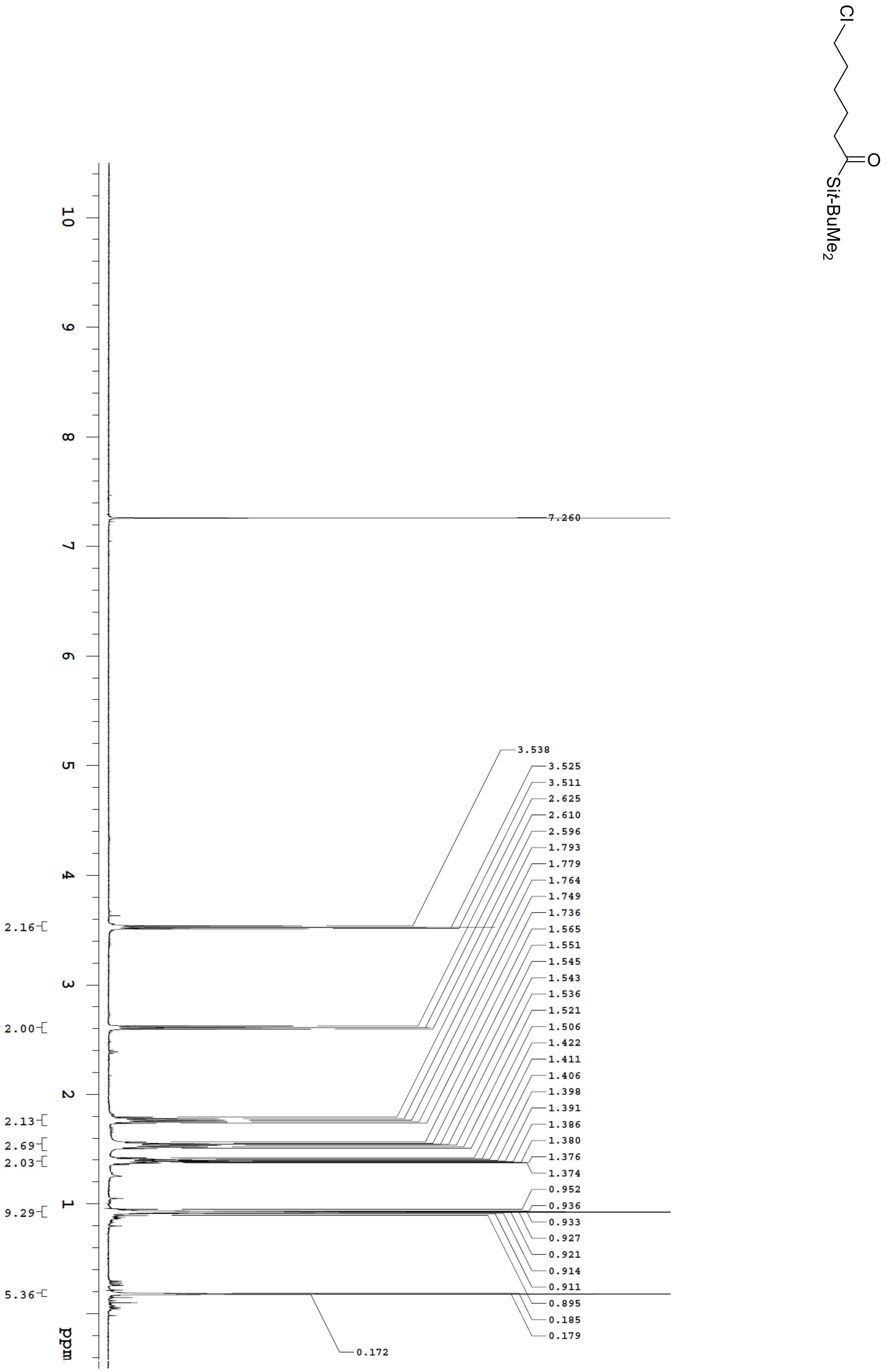

Supplementary Figure 15. ^{1}H NMR Spectrum of 1-(*tert*-Butyldimethylsilyl)-6-chlorohexan-1-one (**1h**)

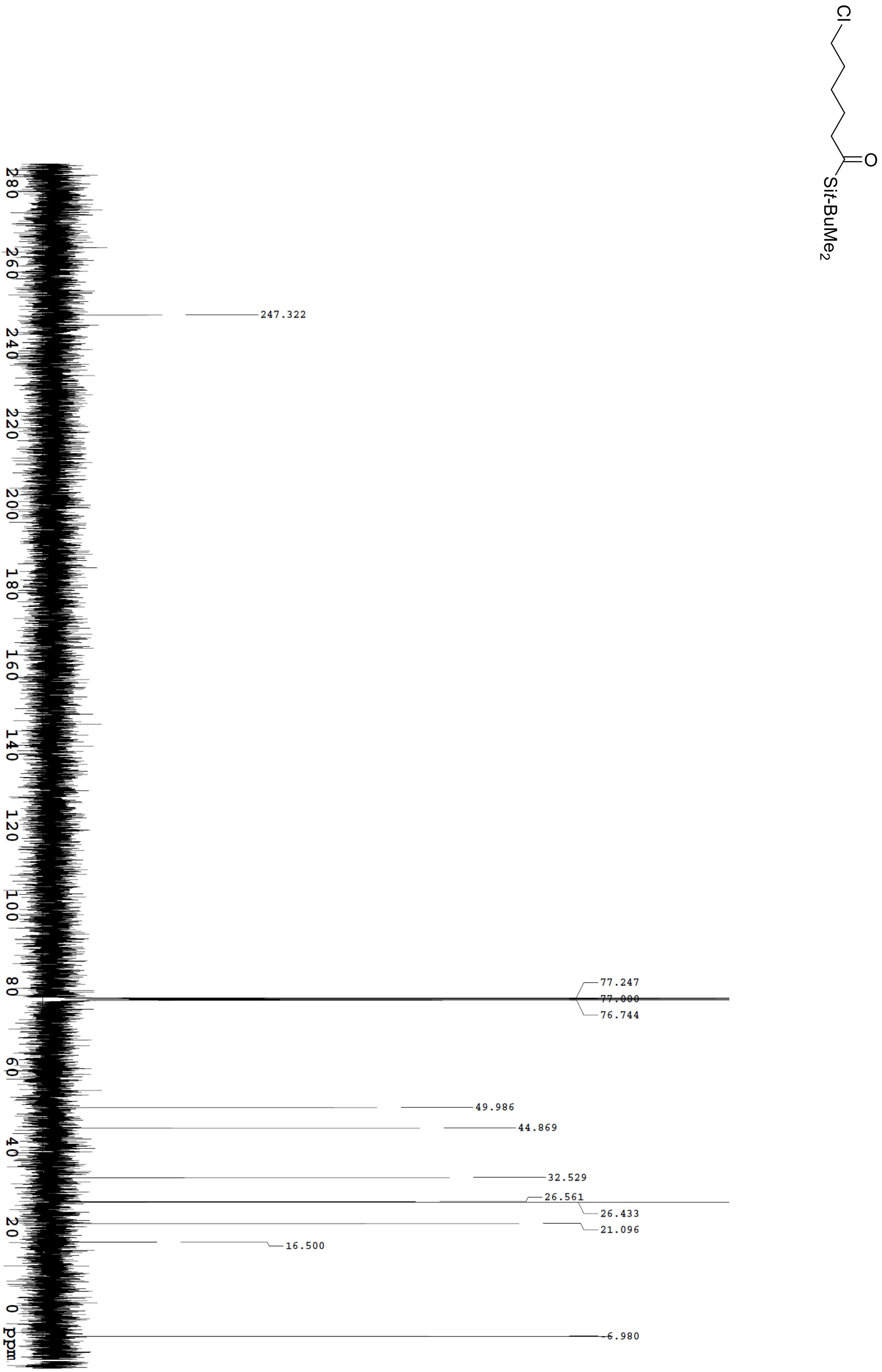

Supplementary Figure 16. ^{13}C NMR Spectrum of 1-(*tert*-Butyldimethylsilyl)-6-chlorohexan-1-one (**1h**)

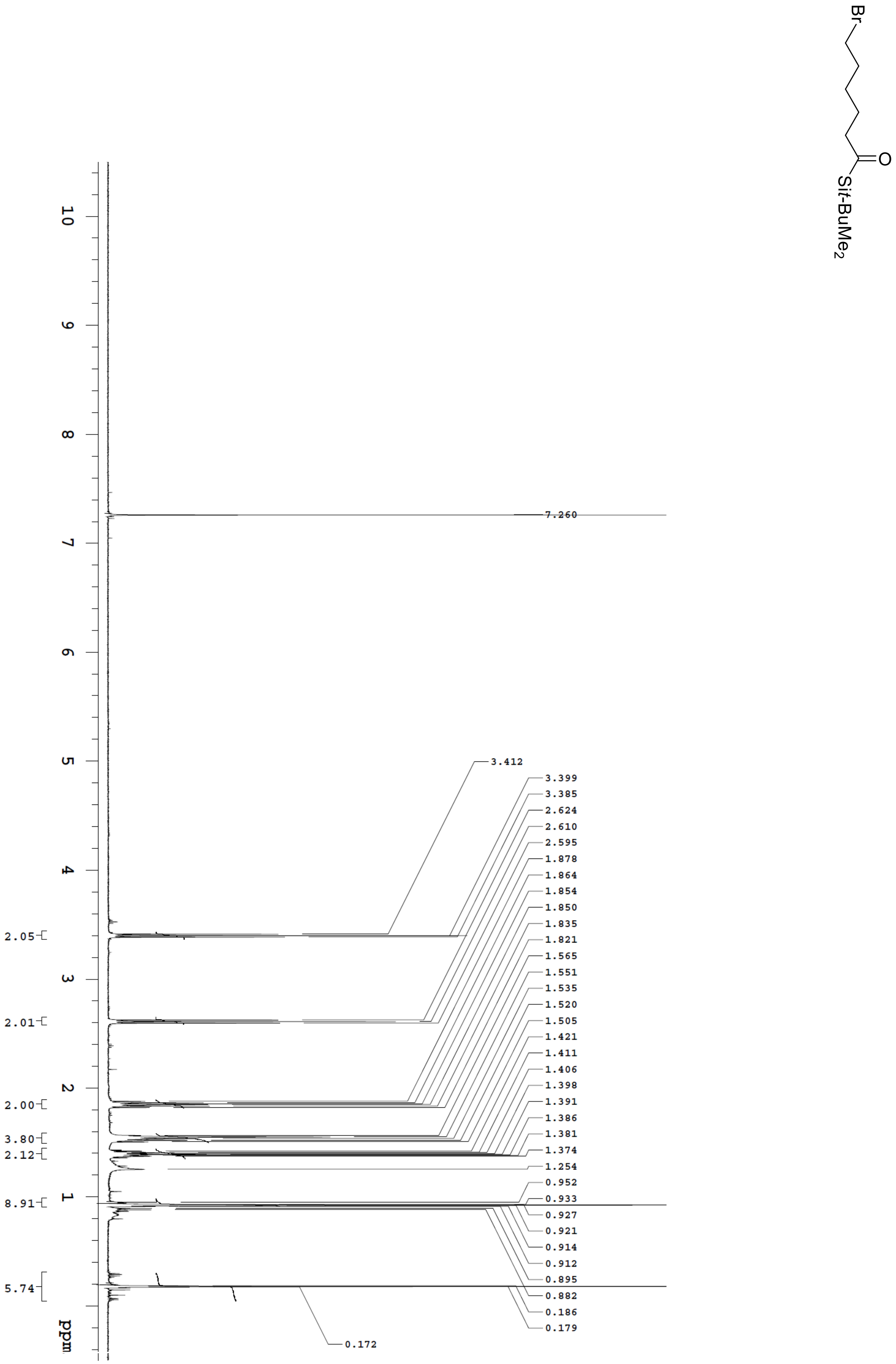

Supplementary Figure 17. ^{1}H NMR Spectrum of 6-Bromo-1-(*tert*-butyldimethylsilyl)hexan-1-one (**1i**)

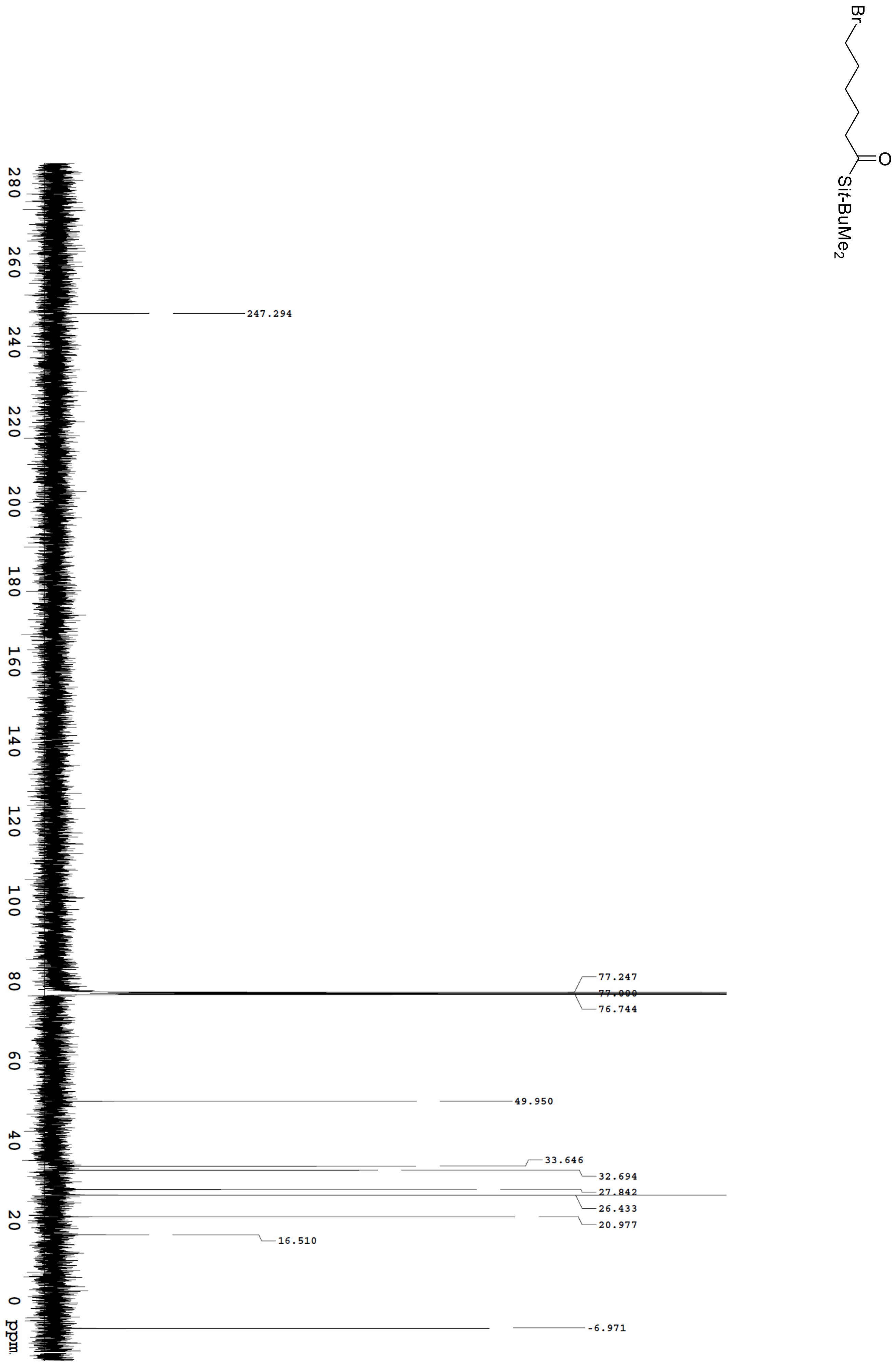

Supplementary Figure 18. ^{13}C NMR Spectrum of 6-Bromo-1-(*tert*-butyldimethylsilyl)hexan-1-one (**1i**)

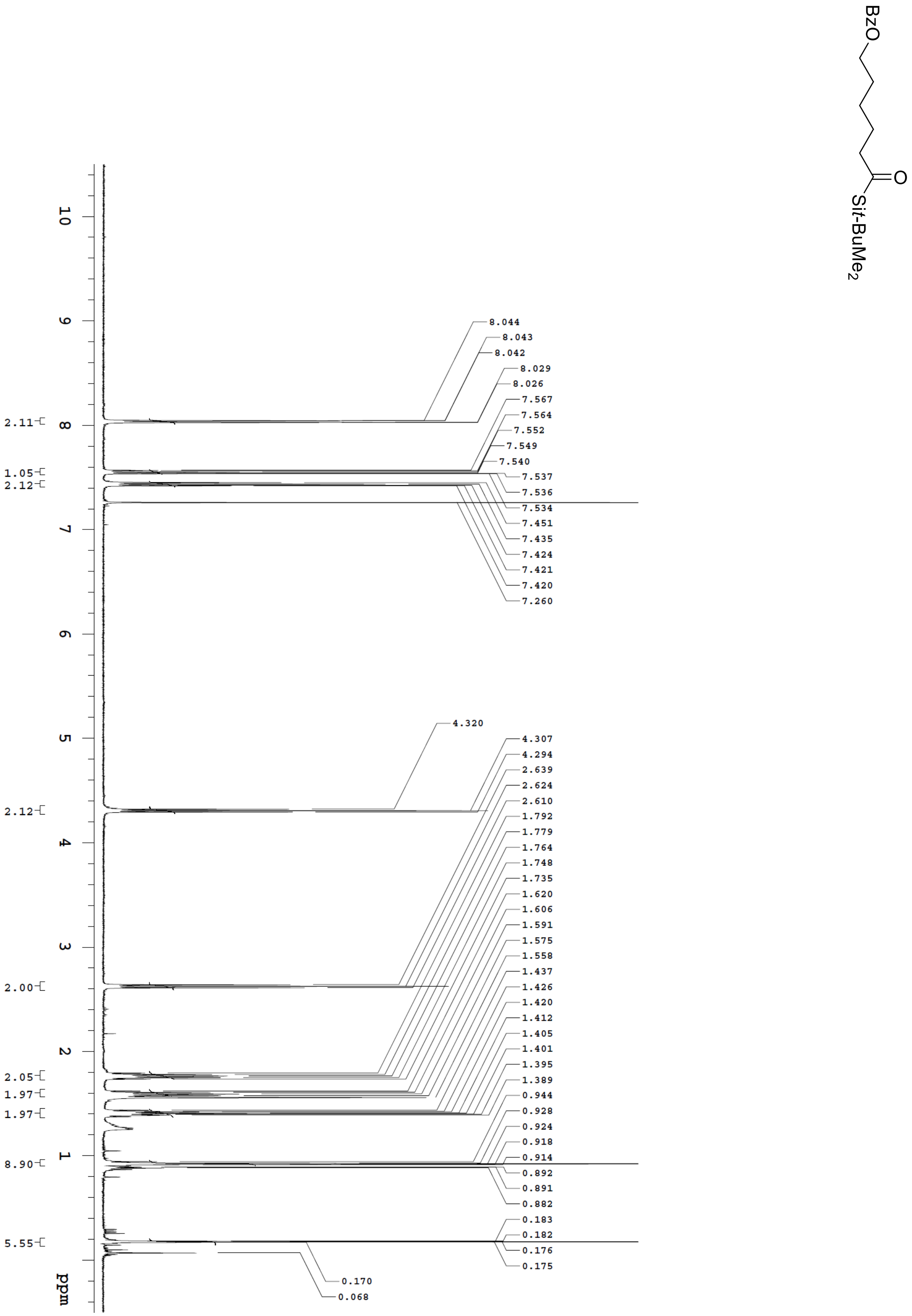

Supplementary Figure 19. ^{1}H NMR Spectrum of 6-(*tert*-Butyldimethylsilyl)-6-oxohexyl benzoate (**1j**)

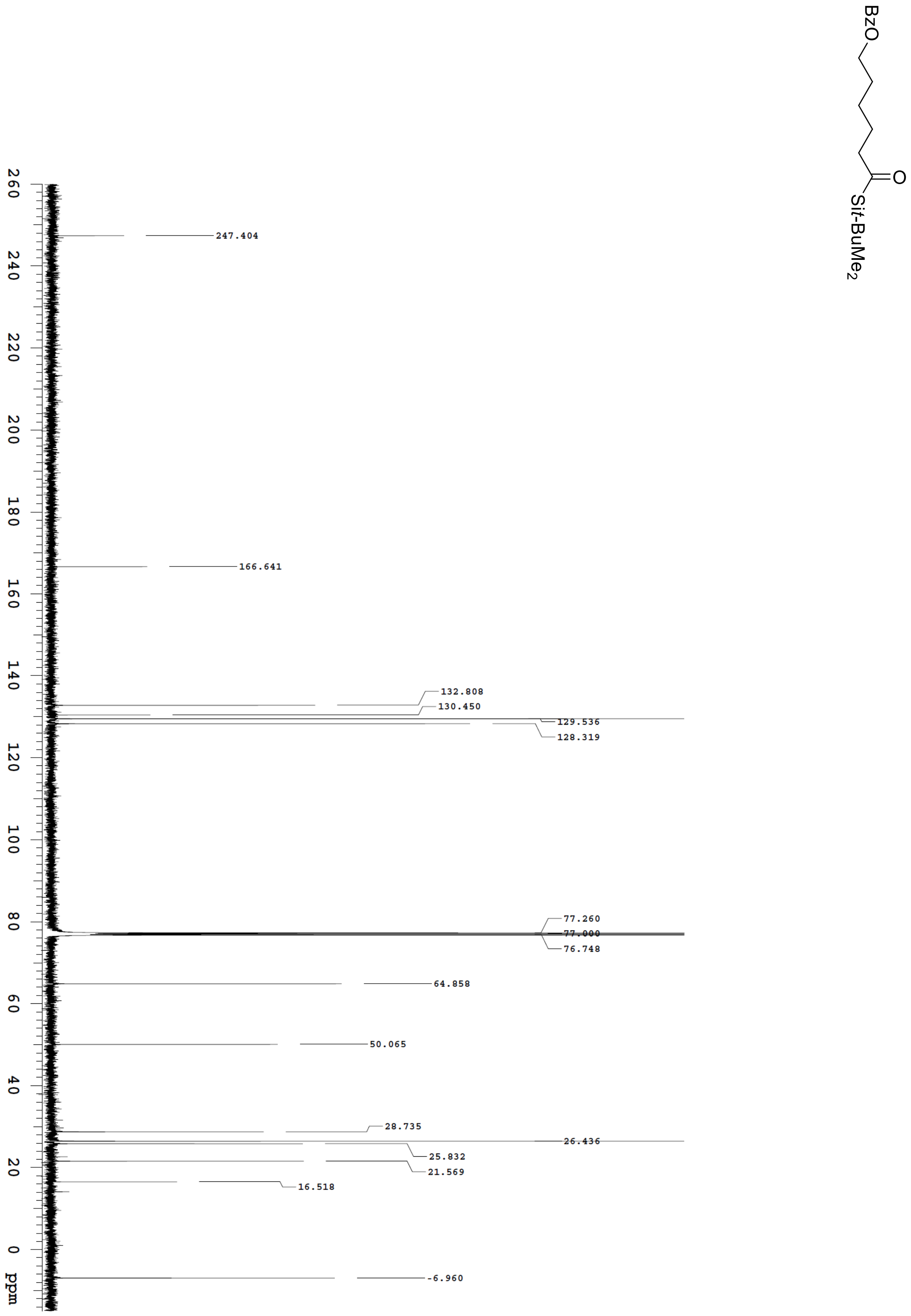

Supplementary Figure 20. ^{13}C NMR Spectrum of 6-(*tert*-Butyldimethylsilyl)-6-oxohexyl benzoate (**1j**)

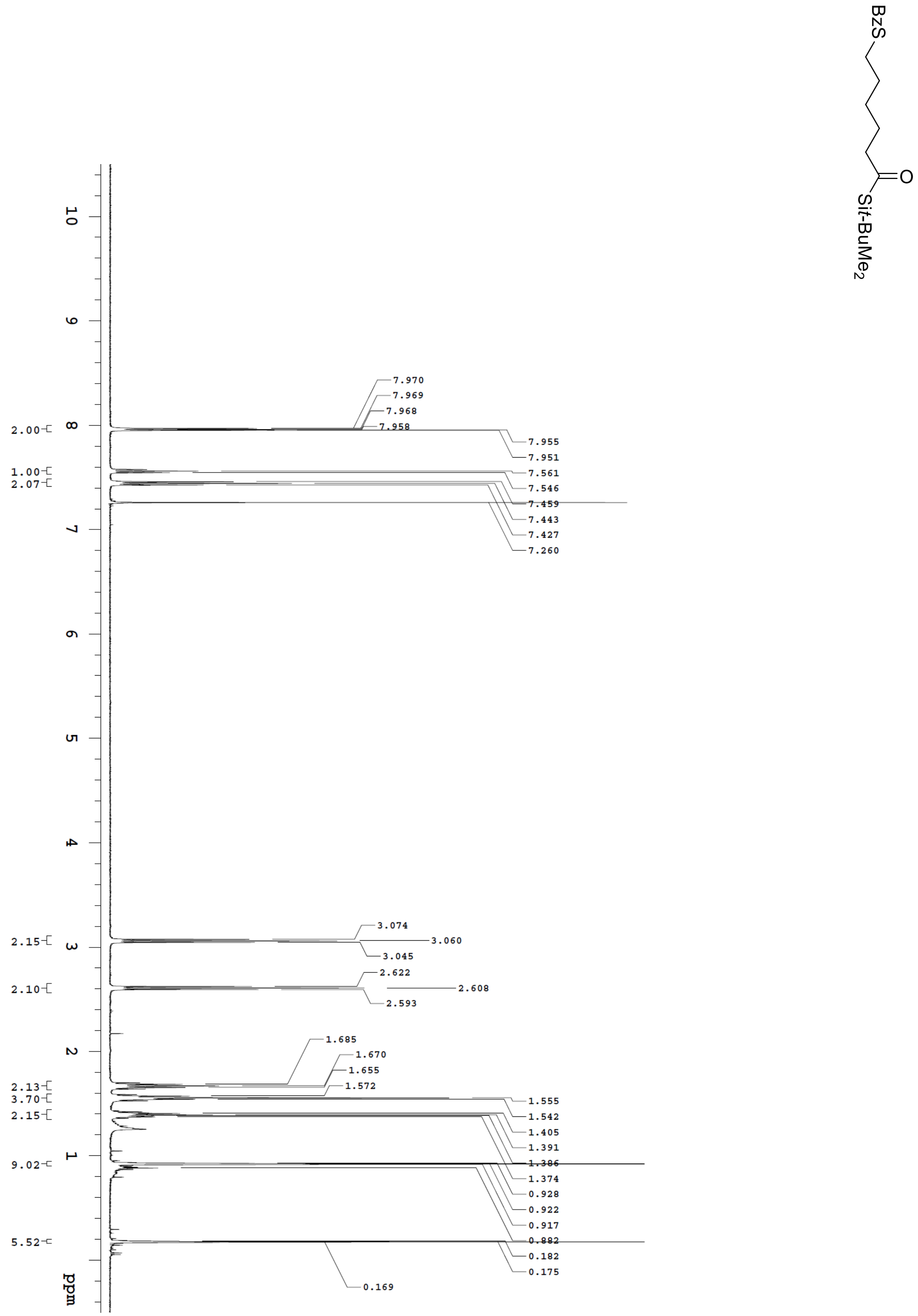

Supplementary Figure 21. ^{1}H NMR Spectrum of *S*-(6-(*tert*-Butyldimethylsilyl)-6-oxohexyl) benzothioate (**1k**)

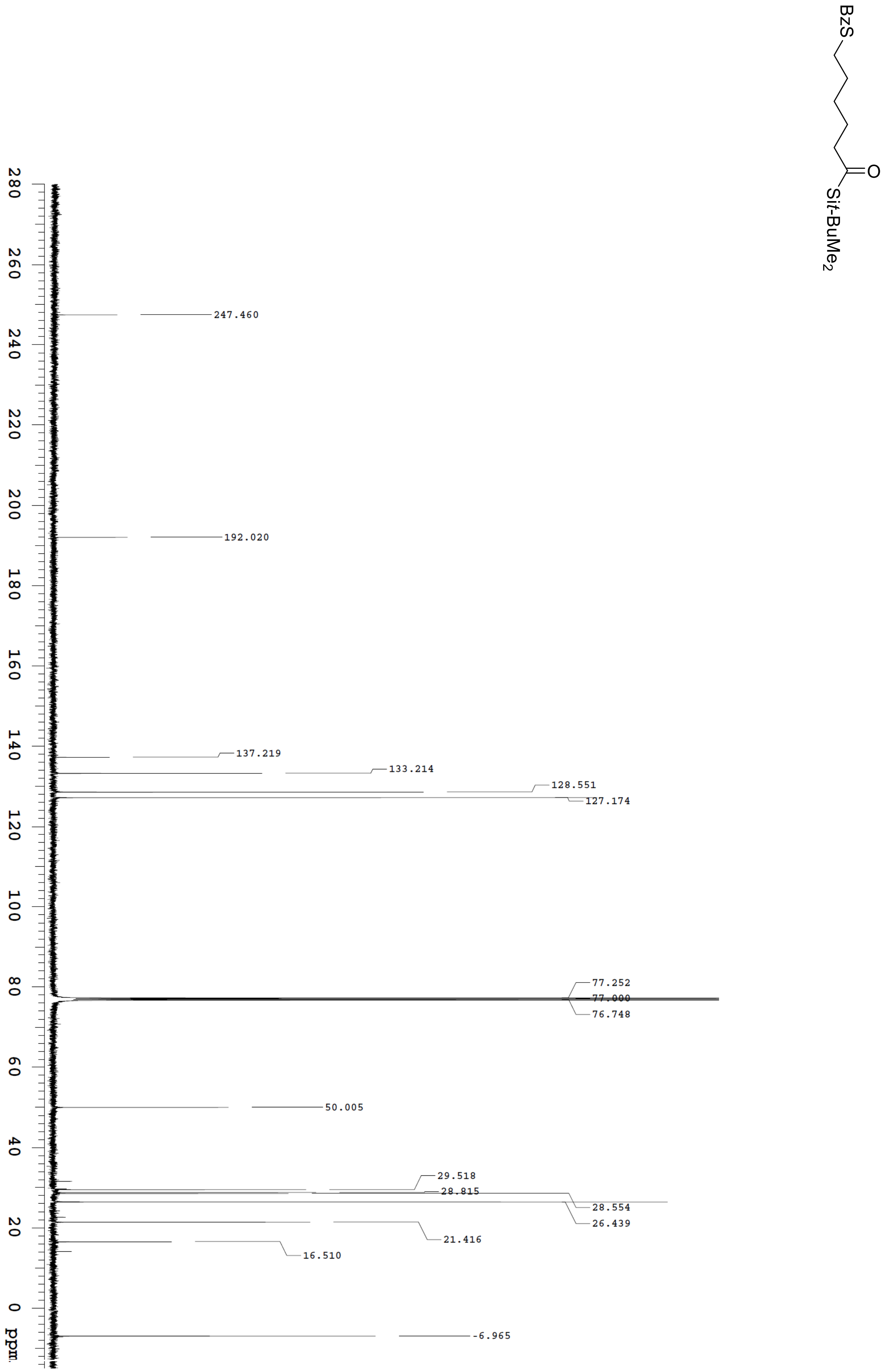

Supplementary Figure 22. ^{13}C NMR Spectrum of

S-(6-(*tert*-Butyldimethylsilyl)-6-oxohexyl) benzothioate (**1k**)

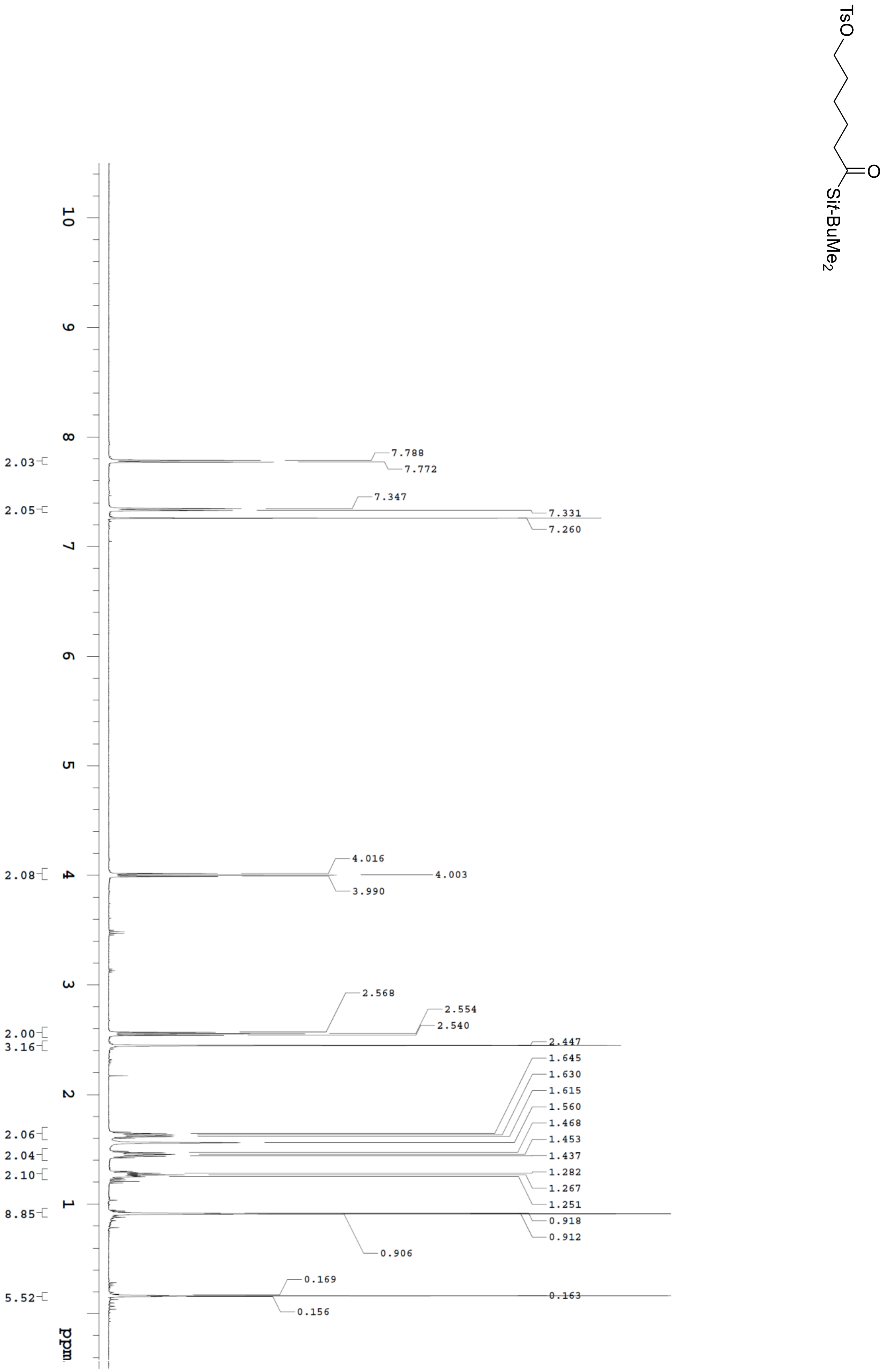

Supplementary Figure 23. ^{1}H NMR Spectrum of 6-(*tert*-Butyldimethylsilyl)-6-oxohexyl 4-methylbenzenesulfonate (**1l**)

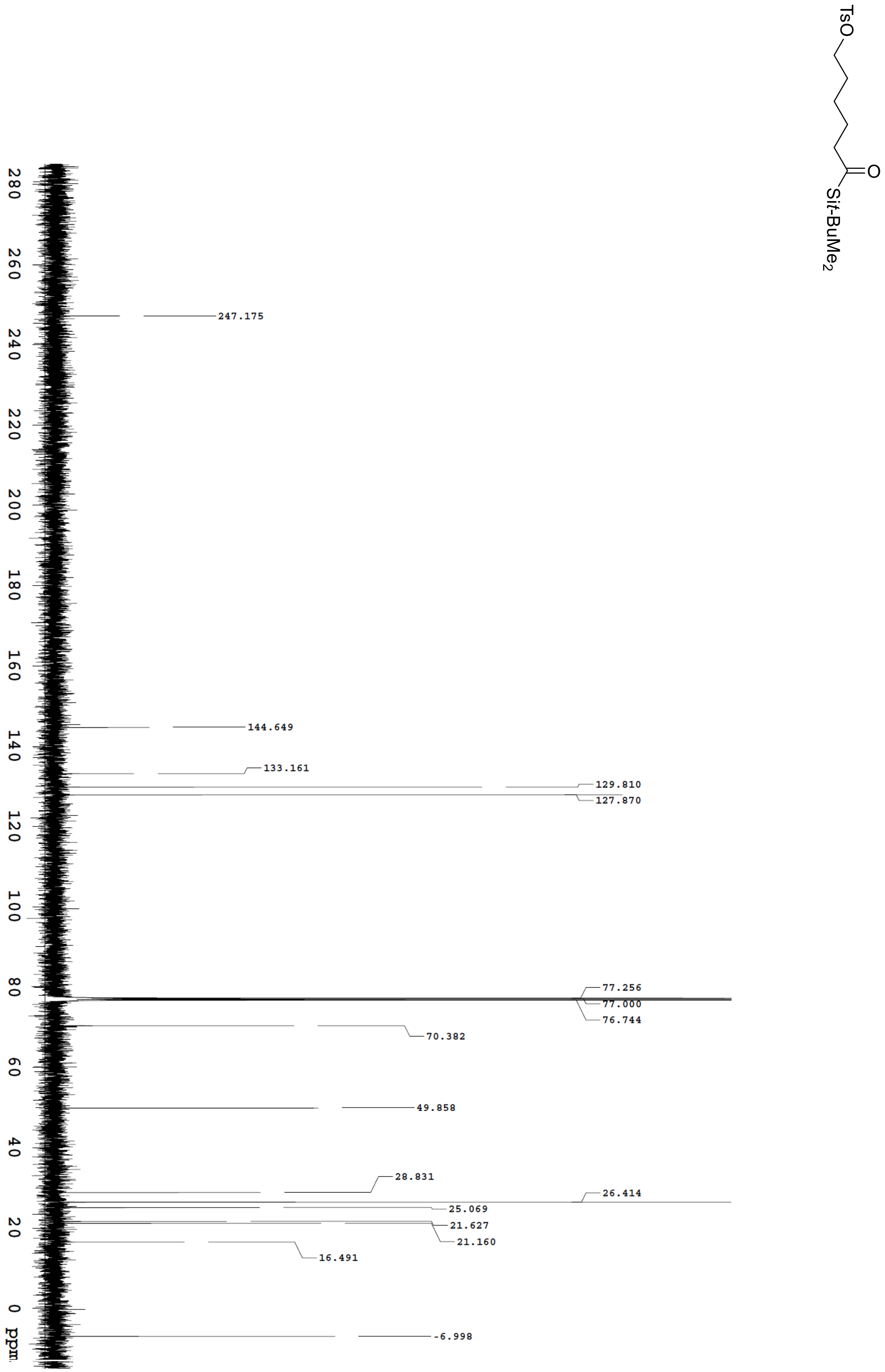

Supplementary Figure 24. ^{13}C NMR Spectrum of 6-(*tert*-Butyldimethylsilyl)-6-oxohexyl 4-methylbenzenesulfonate (**1l**)

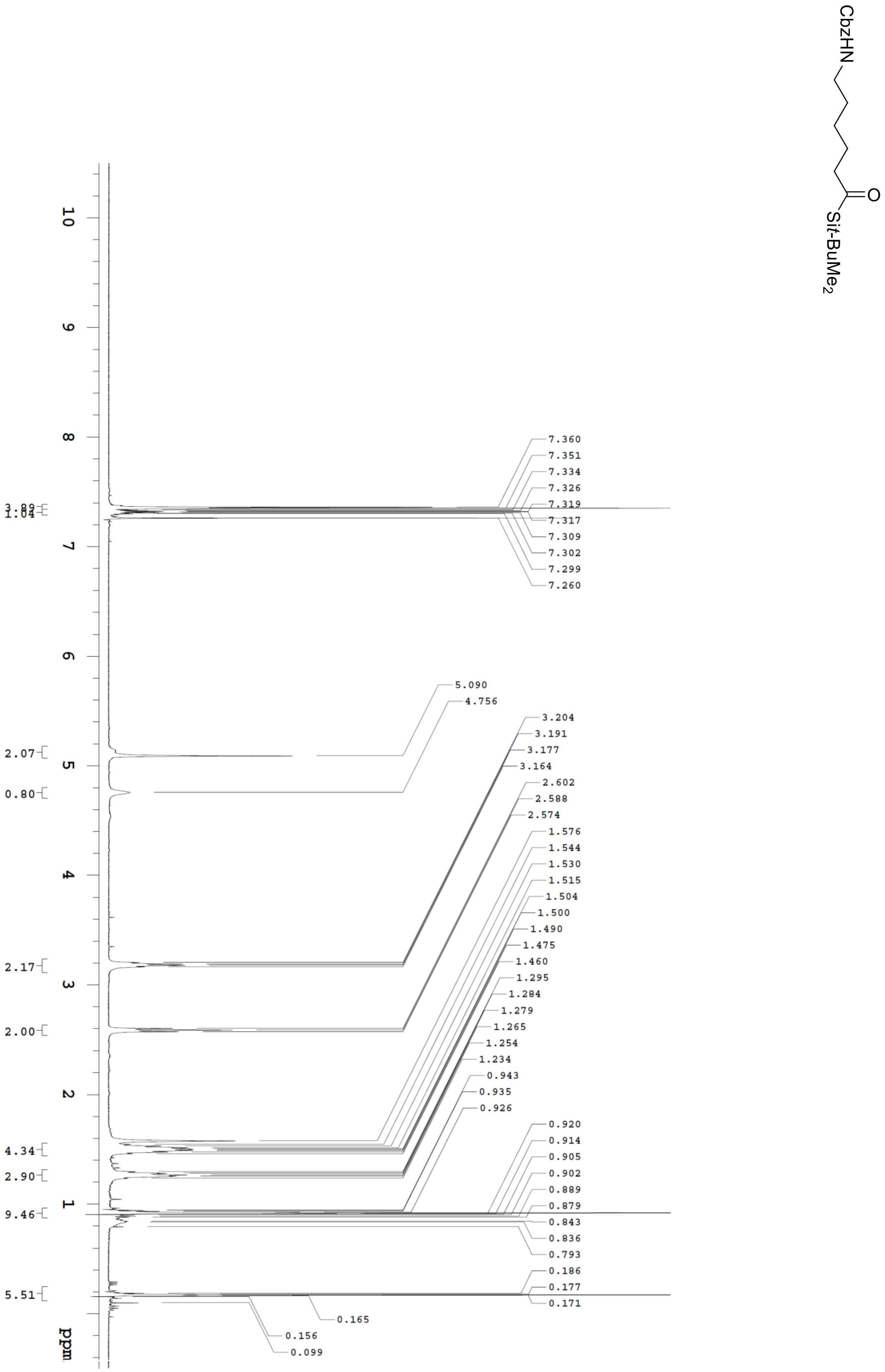

Supplementary Figure 25. ^{1}H NMR Spectrum of

Benzyl (6-(*tert*-butyldimethylsilyl)-6-oxohexyl)carbamate (**1m**)

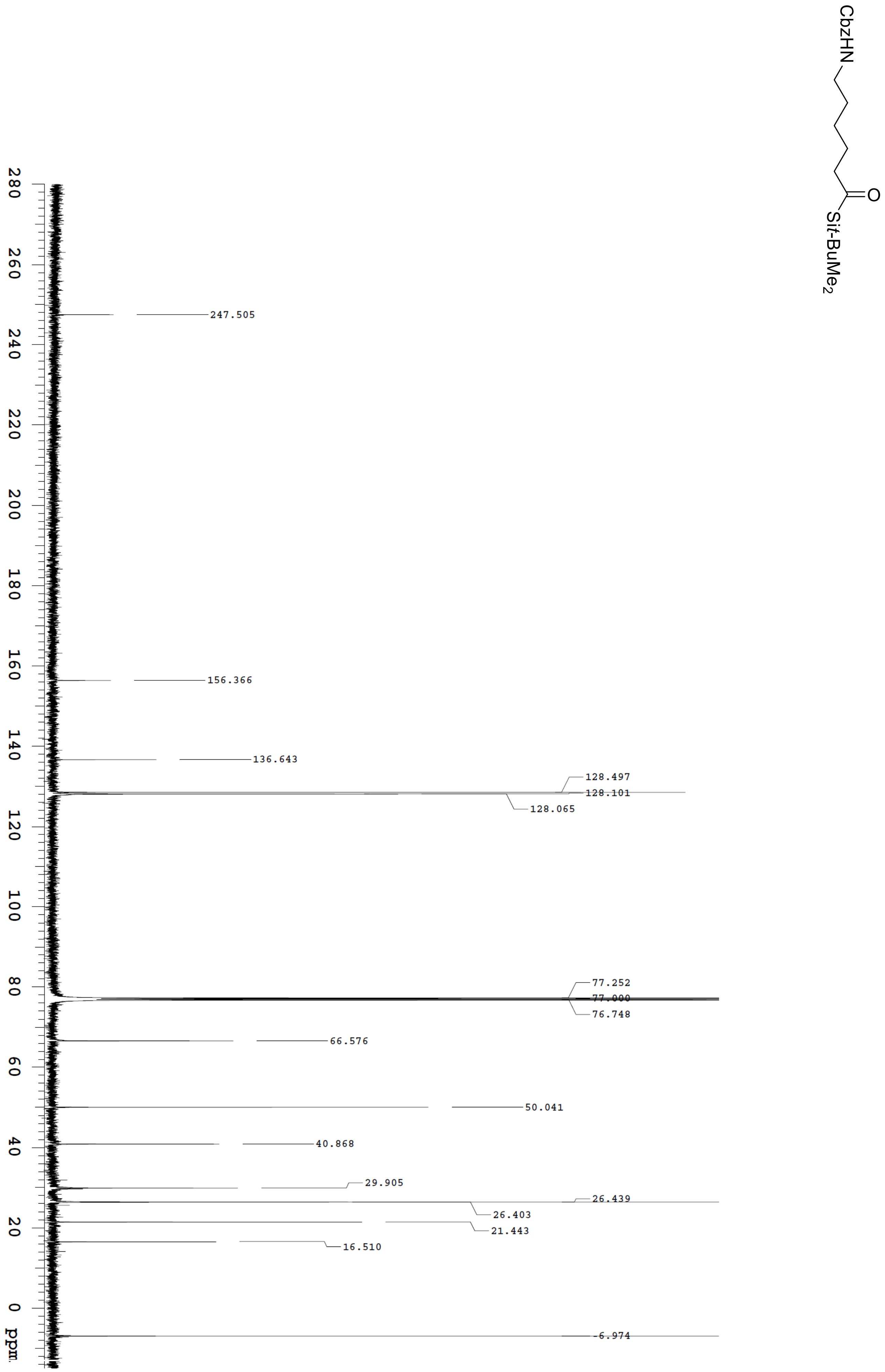

Supplementary Figure 26. ^{13}C NMR Spectrum of

Benzyl (6-(*tert*-butyldimethylsilyl)-6-oxohexyl)carbamate (**1m**)

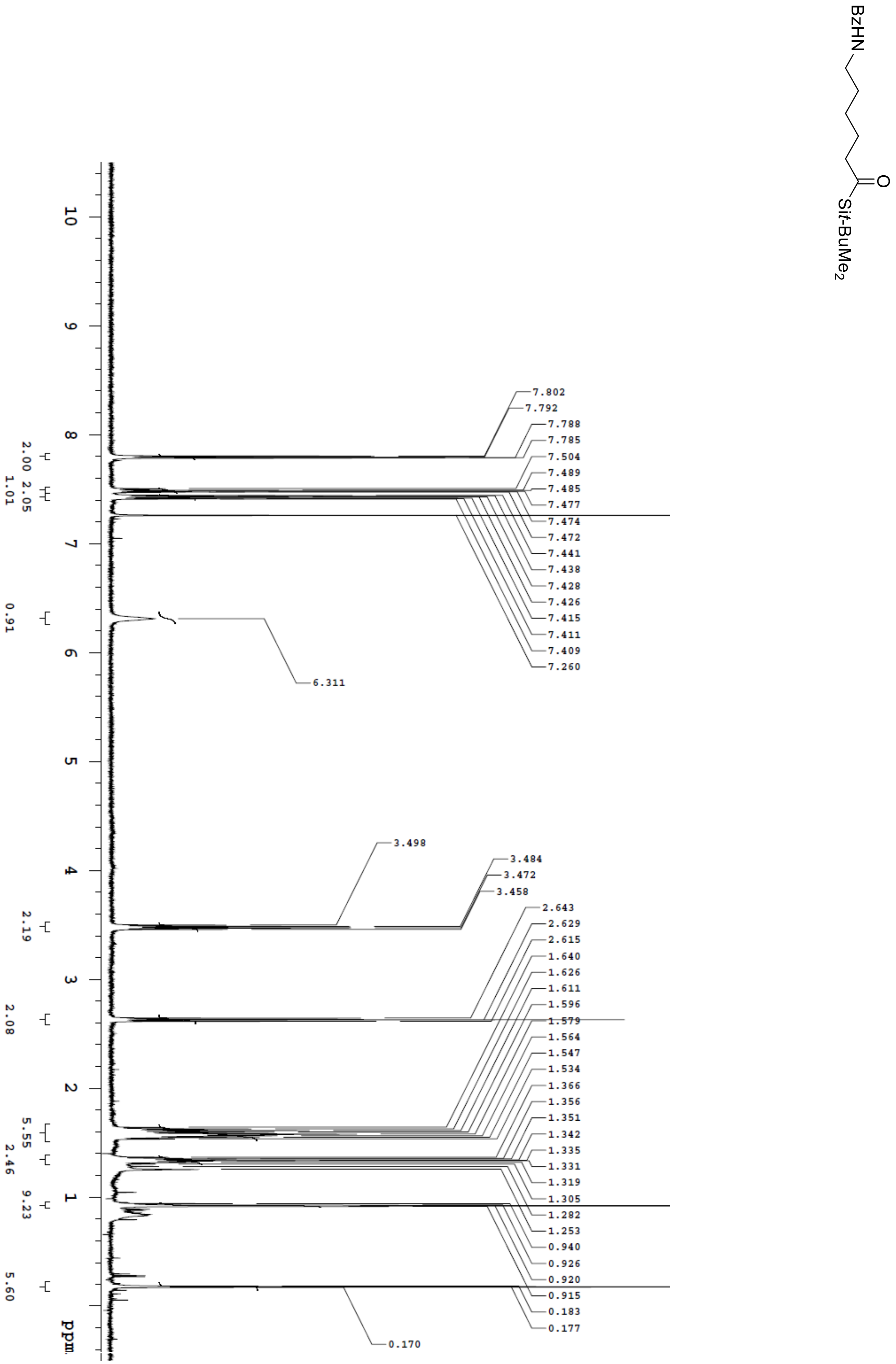

Supplementary Figure 27. ^{1}H NMR Spectrum of *N*-(6-(*tert*-butyldimethylsilyl)-6-oxohexyl)benzamide (**1n**)

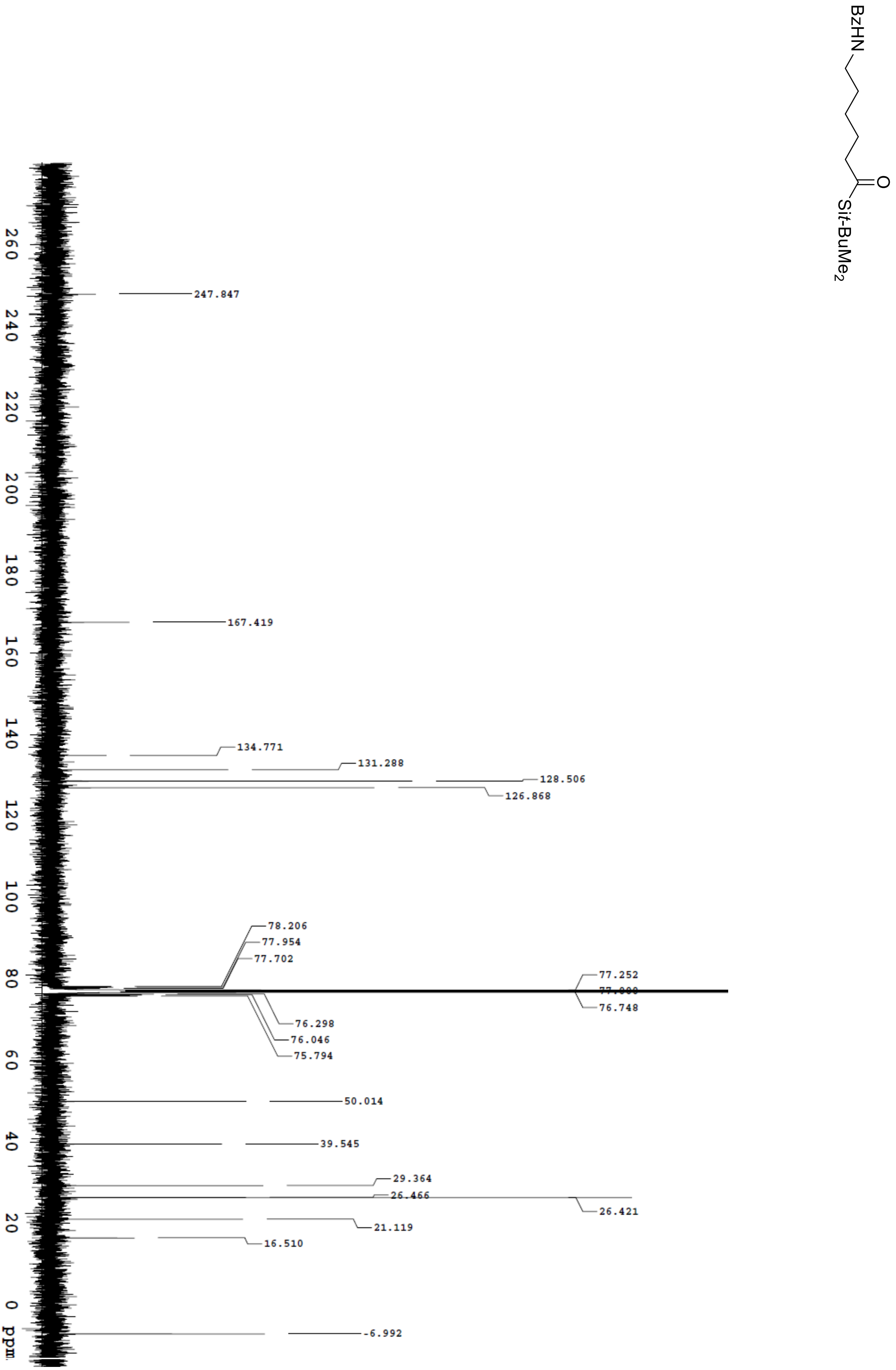

Supplementary Figure 28. ^{13}C NMR Spectrum of *N*-(6-(*tert*-butyldimethylsilyl)-6-oxohexyl)benzamide (**1n**)

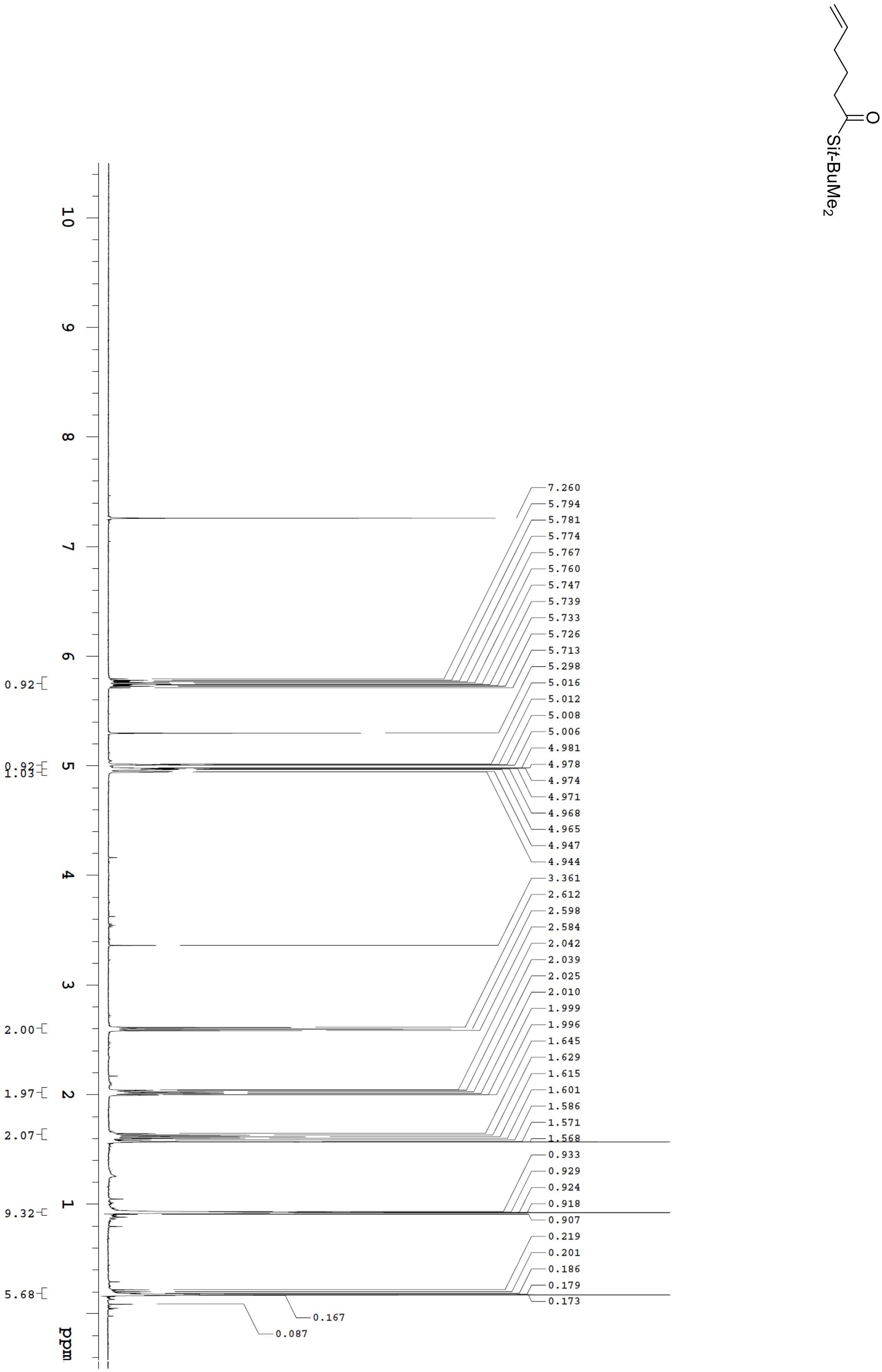

Supplementary Figure 29. ^{1}H NMR Spectrum of 1-(*tert*-Butyldimethylsilyl)hex-5-en-1-one (**1o**)

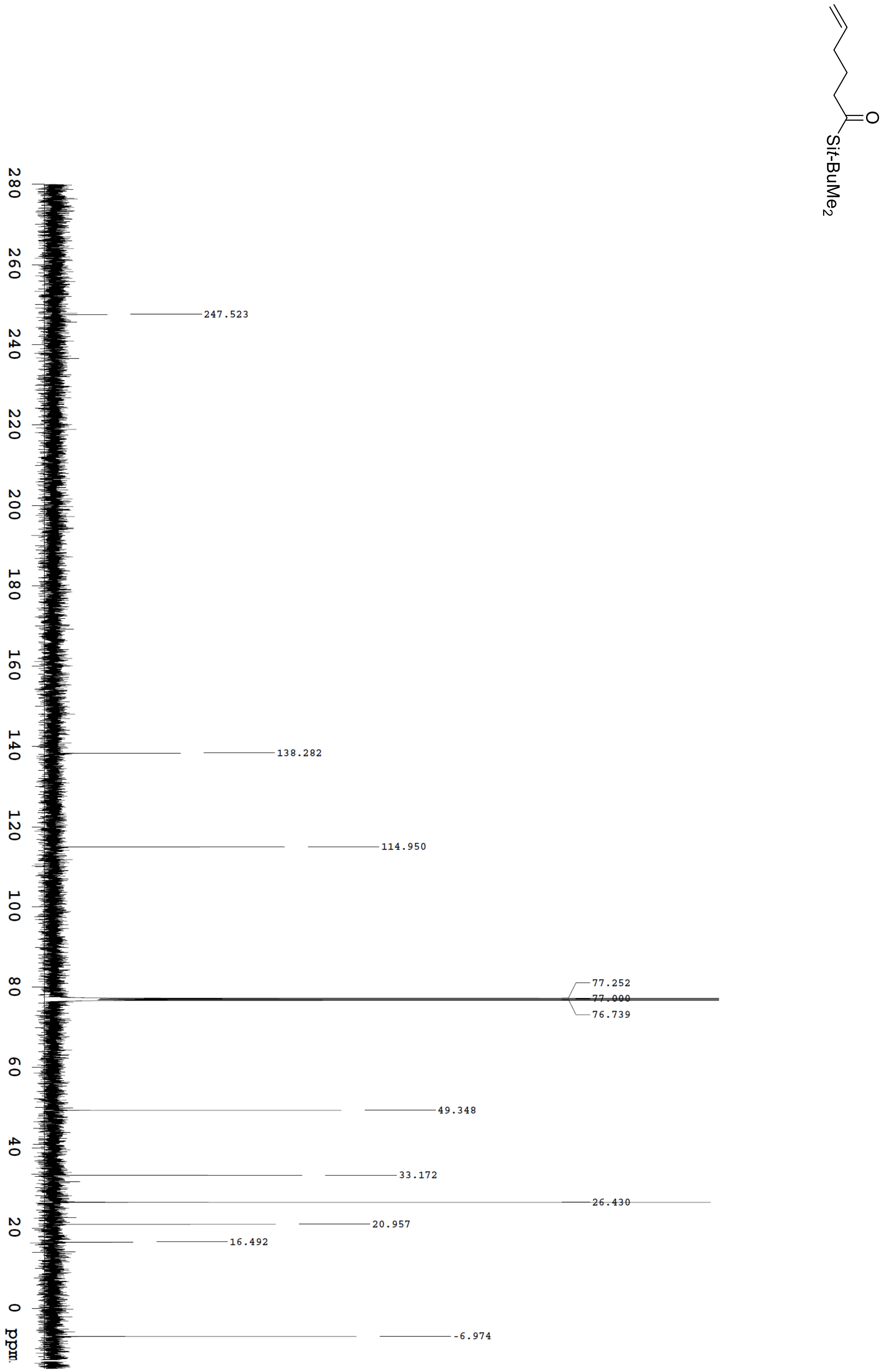

Supplementary Figure 30. ^{13}C NMR Spectrum of 1-(*tert*-Butyldimethylsilyl)hex-5-en-1-one (**1o**)

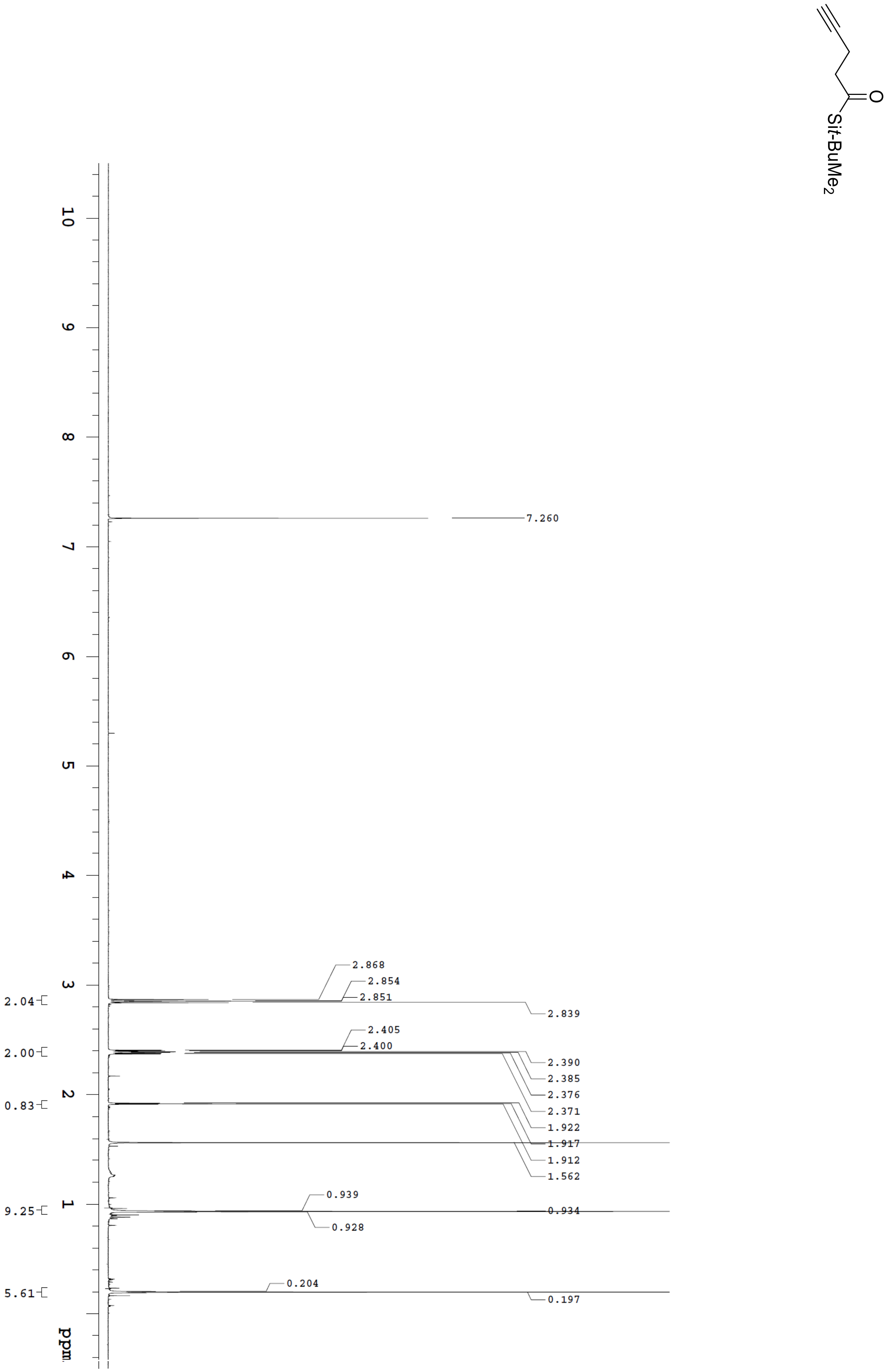

Supplementary Figure 31. ^{1}H NMR Spectrum of 1-(*tert*-Butyldimethylsilyl)pent-4-yn-1-one (**1p**)

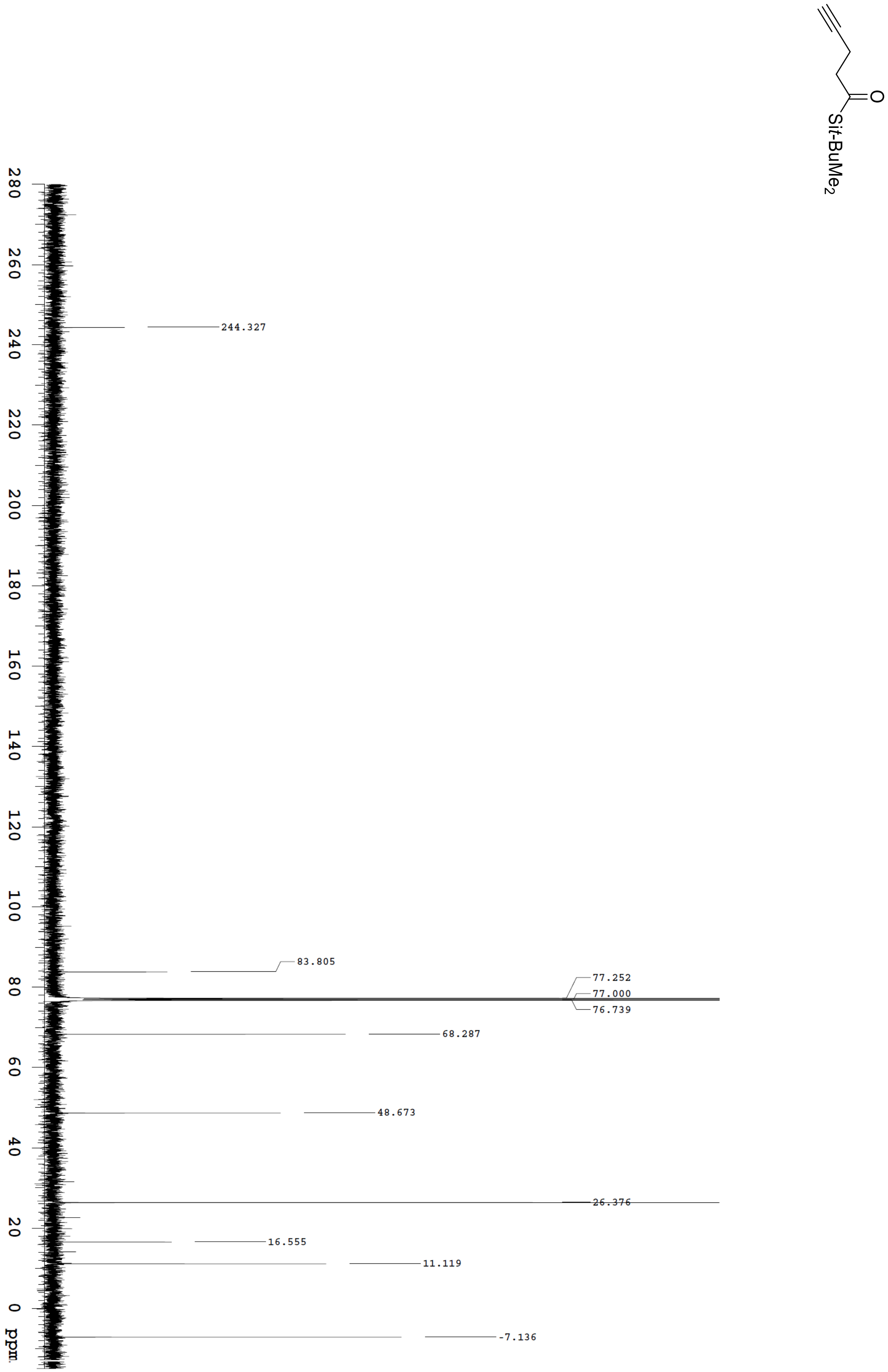

Supplementary Figure 32. ^{13}C NMR Spectrum of 1-(*tert*-Butyldimethylsilyl)pent-4-yn-1-one (**1p**)

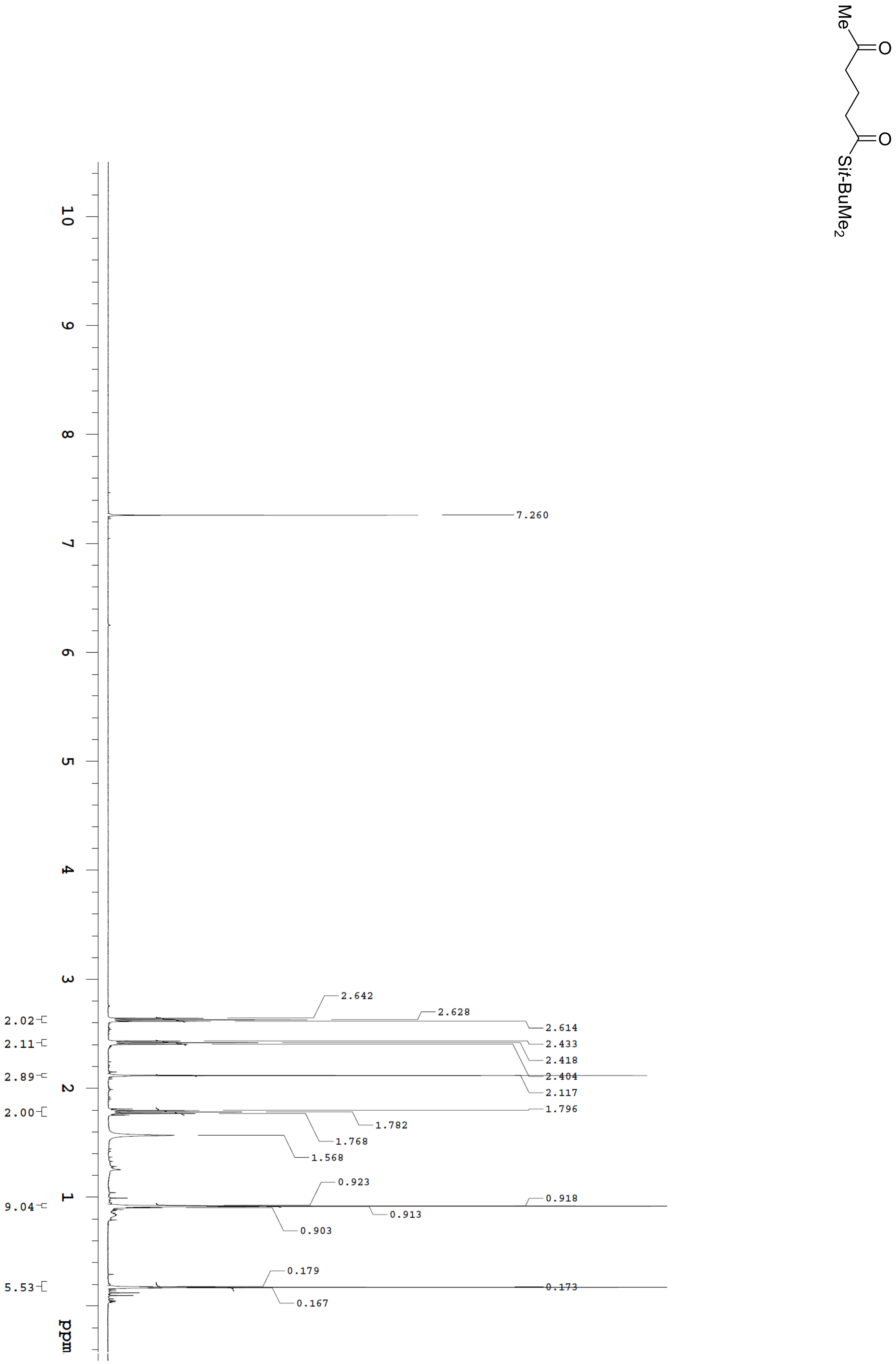

Supplementary Figure 33. ^{1}H NMR Spectrum of 1-(*tert*-Butyldimethylsilyl)hexane-1,5-dione (**1q**)

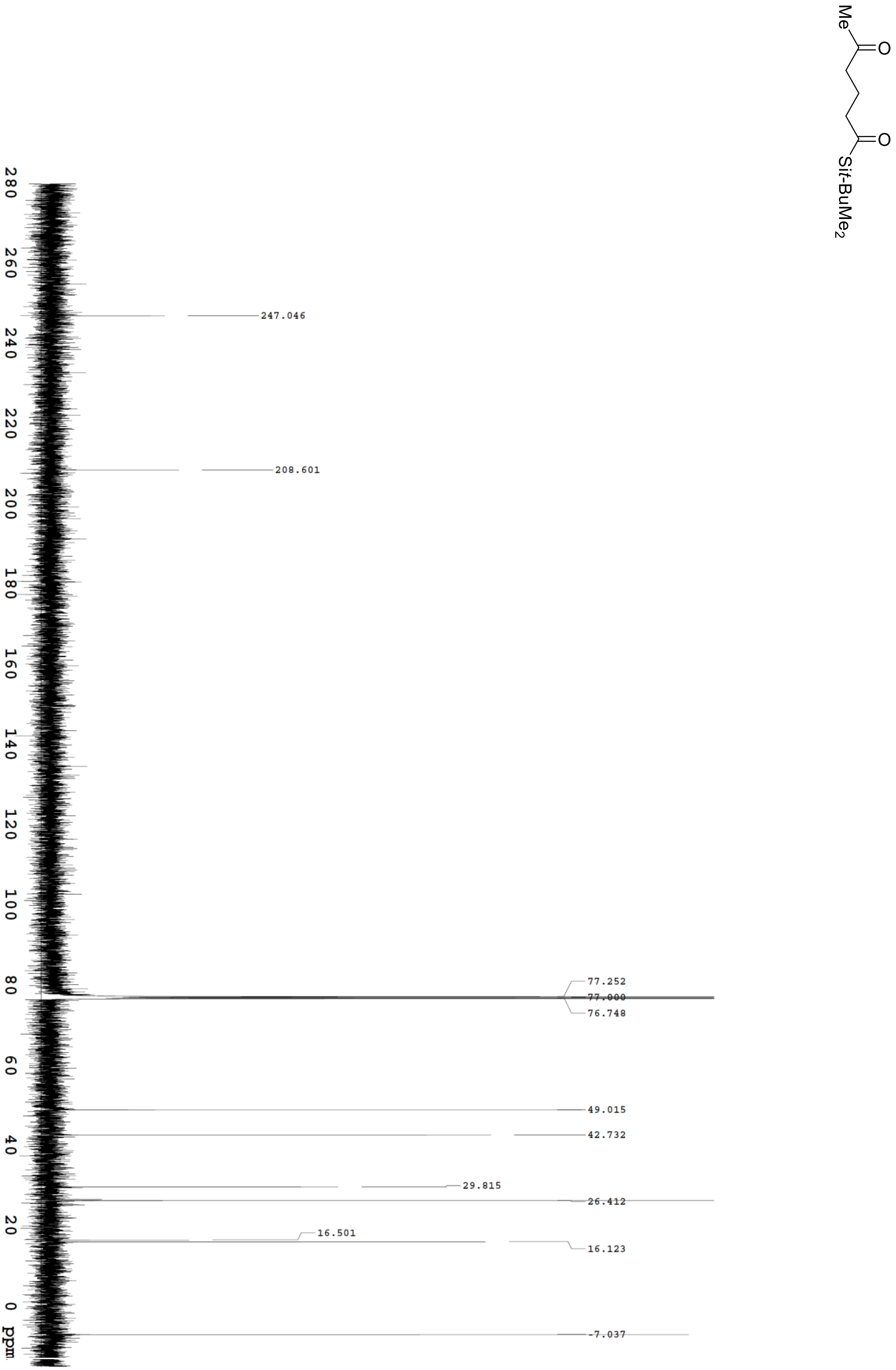

Supplementary Figure 34. ^{13}C NMR Spectrum of 1-(*tert*-Butyldimethylsilyl)hexane-1,5-dione (**1q**)

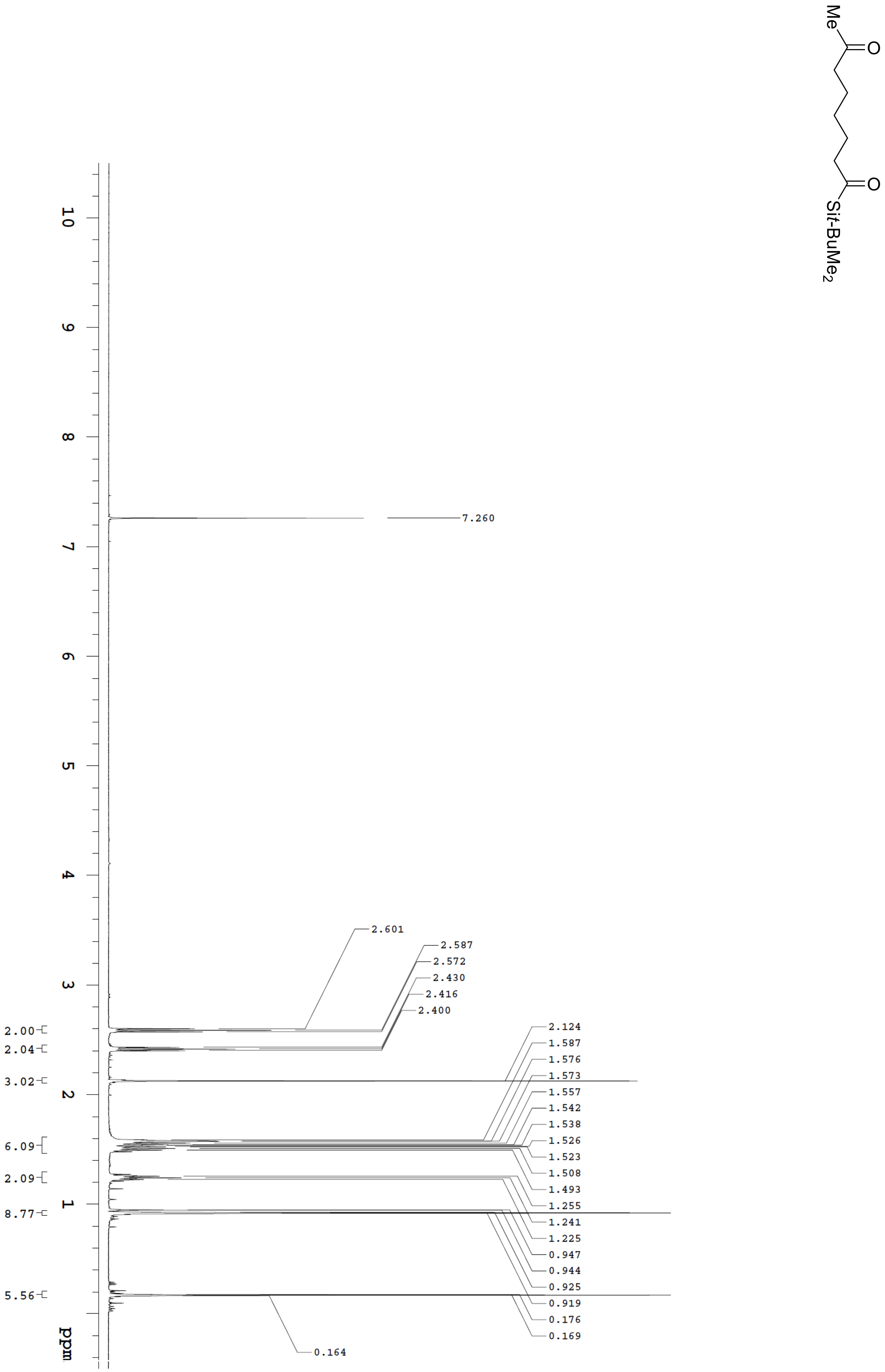

Supplementary Figure 35. ^{1}H NMR Spectrum of 1-(*tert*-Butyldimethylsilyl)octane-1,7-dione (**1r**)

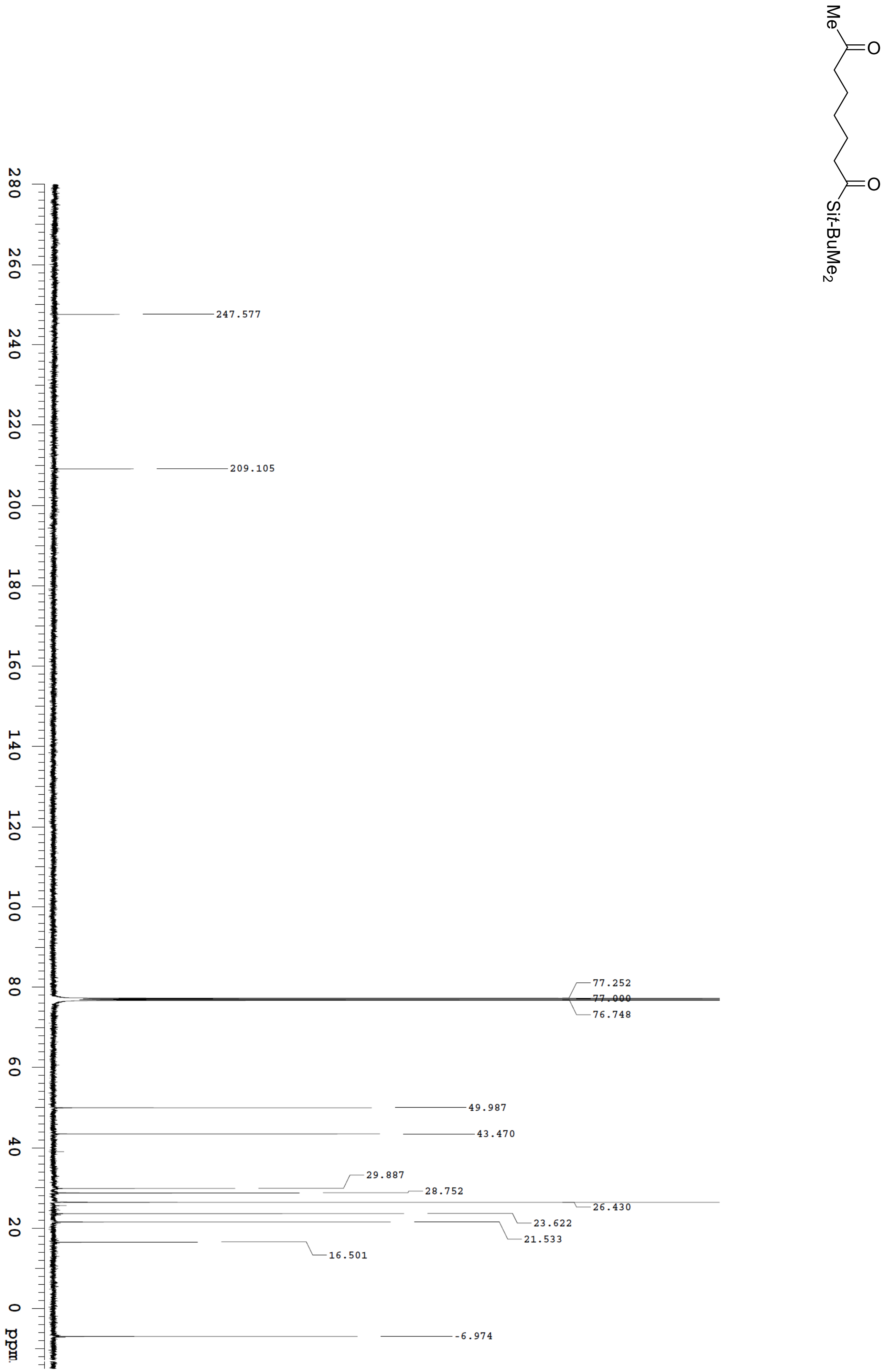

Supplementary Figure 36. ^{13}C NMR Spectrum of 1-(*tert*-Butyldimethylsilyl)octane-1,7-dione (**1r**)

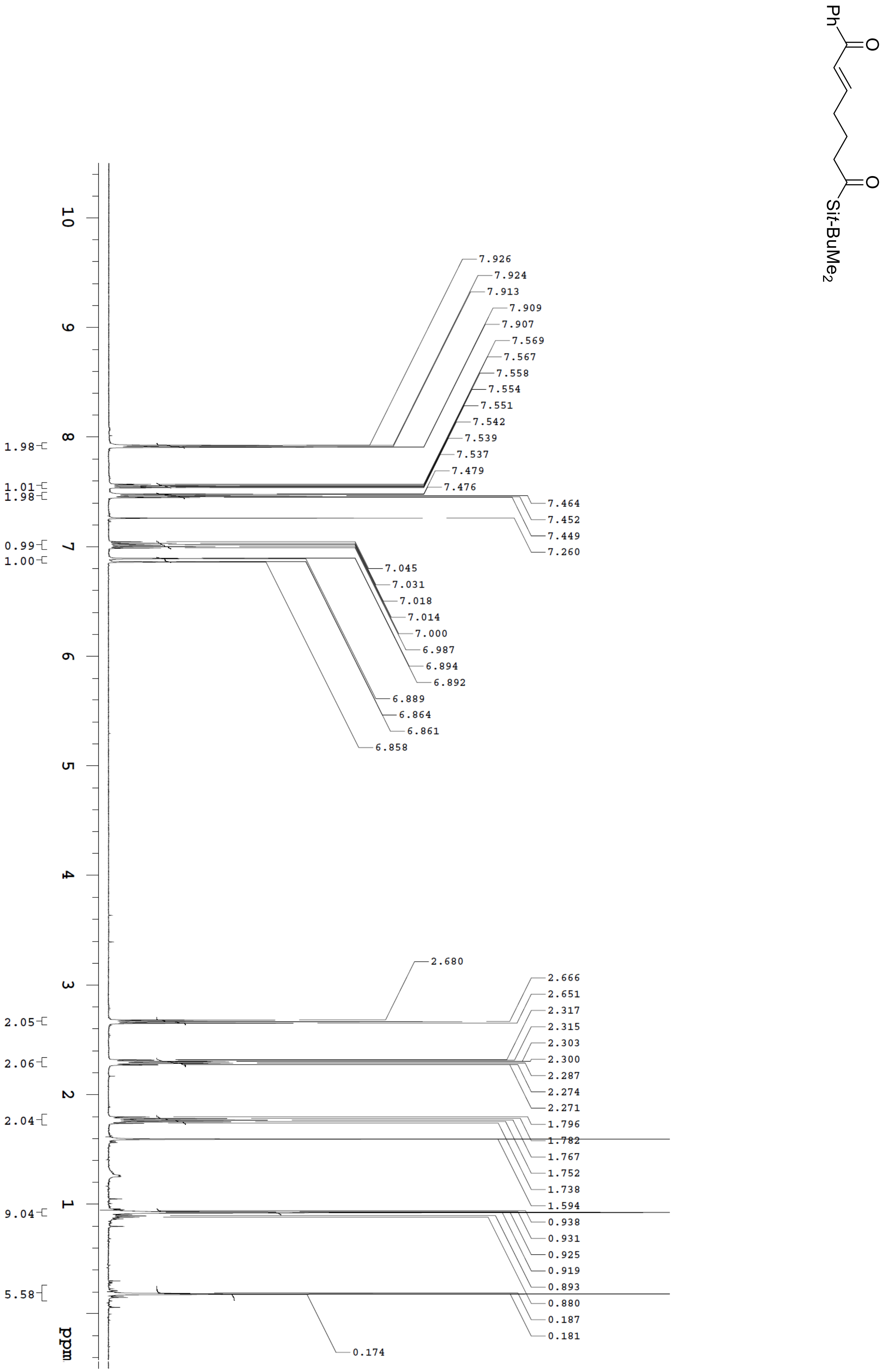

Supplementary Figure 37. ^{1}H NMR Spectrum of (*E*)-7-(*tert*-Butyldimethylsilyl)-1-phenylhept-2-ene-1,7-dione (**1s**)

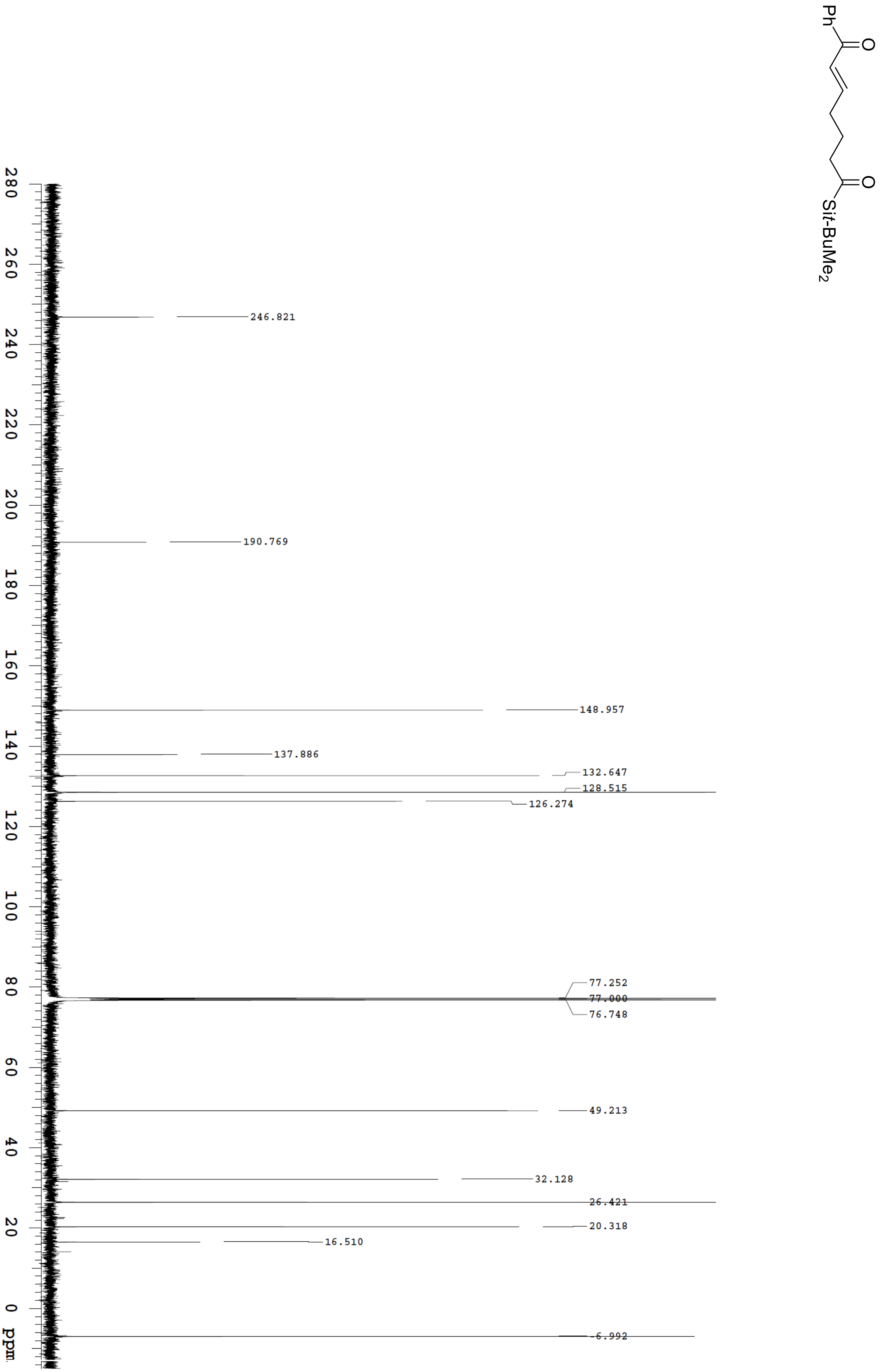

Supplementary Figure 38. ^{13}C NMR Spectrum of (*E*)-7-(*tert*-Butyldimethylsilyl)-1-phenylhept-2-ene-1,7-dione (**1s**)

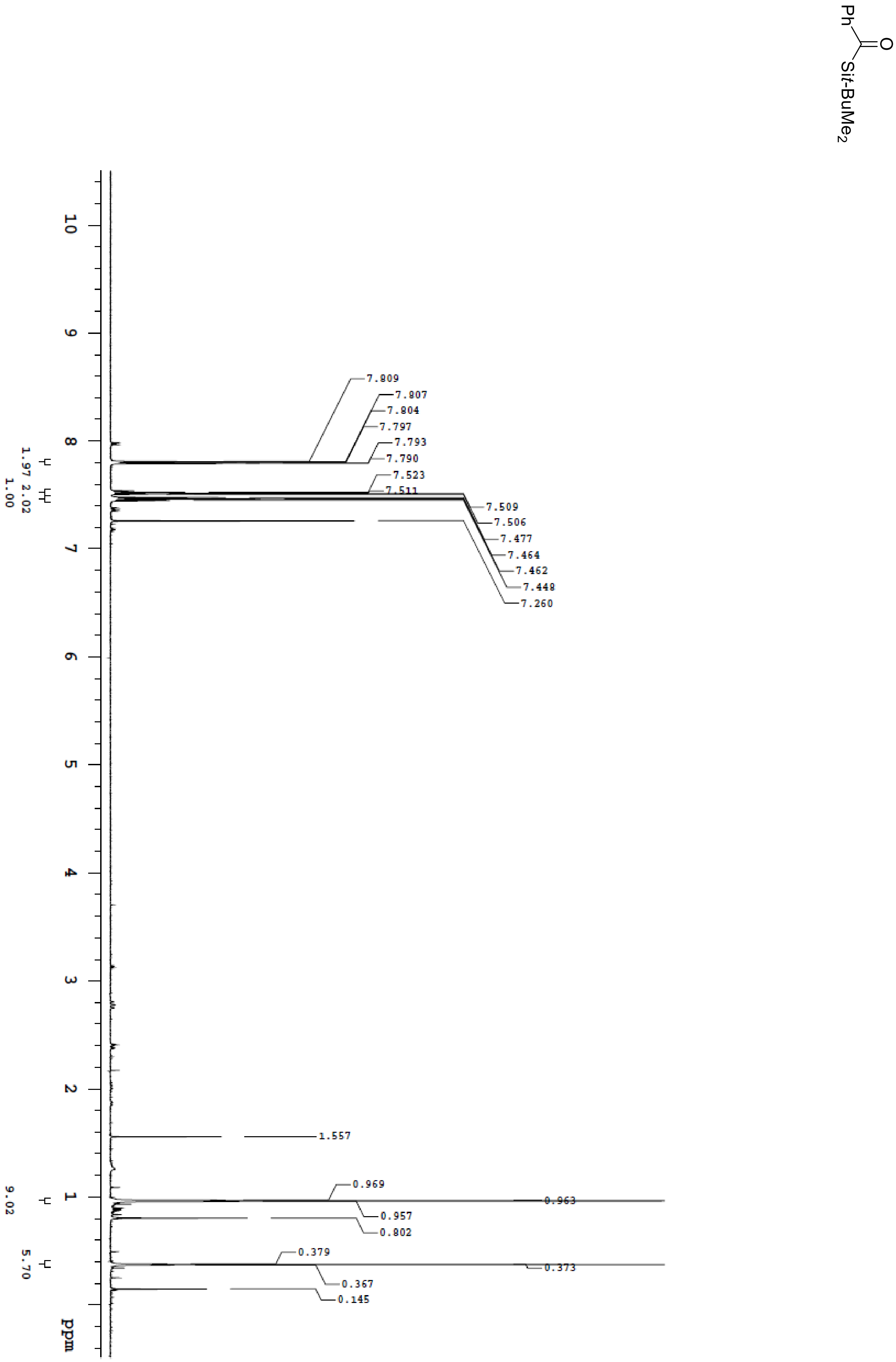

Supplementary Figure 39. ^{1}H NMR Spectrum of (*tert*-Butyldimethylsilyl)(phenyl)methanone (**1t**)

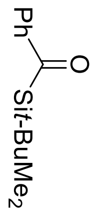

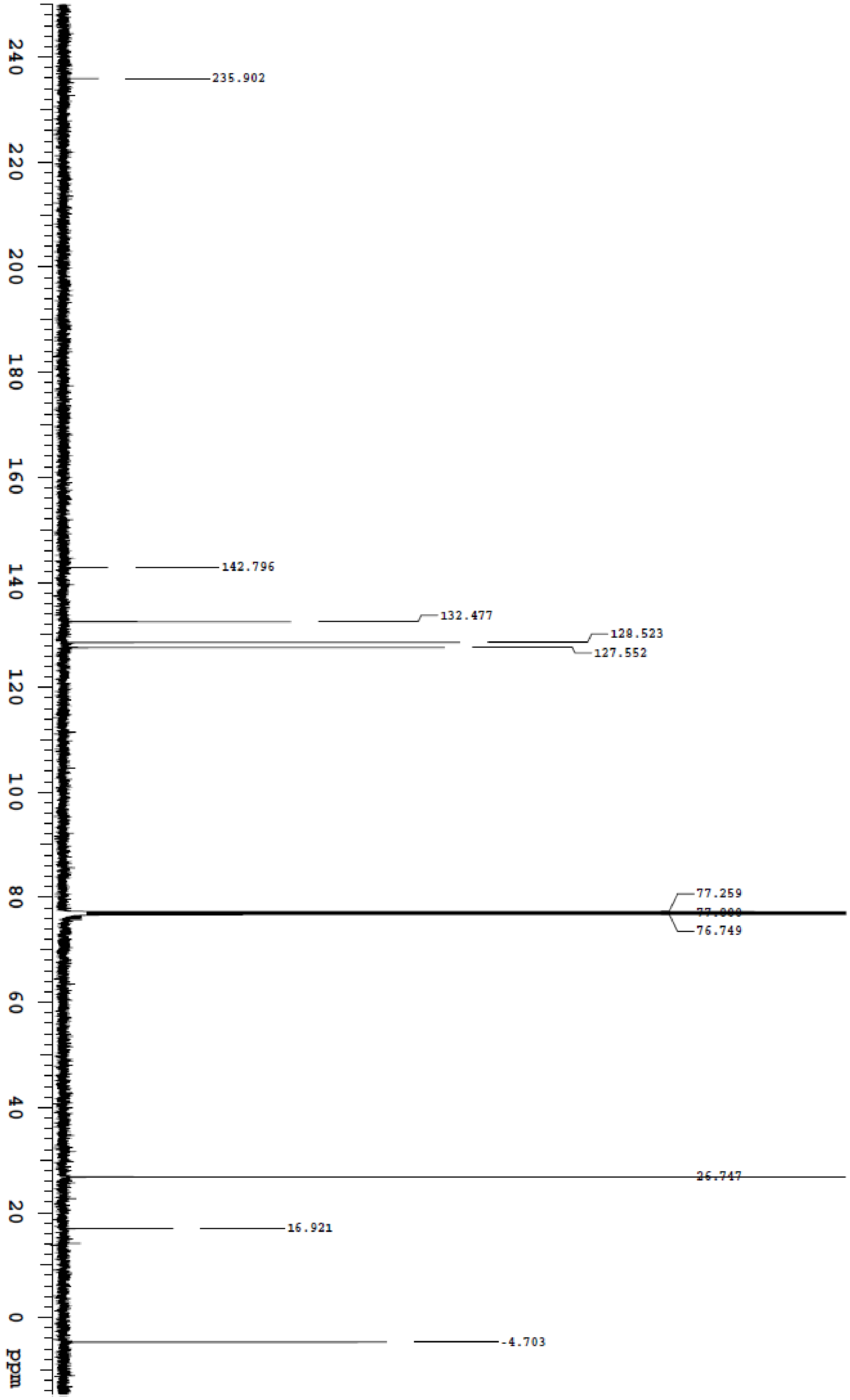

Supplementary Figure 40. ^{13}C NMR Spectrum of (*tert*-Butyldimethylsilyl)(phenyl)methanone (**1t**)

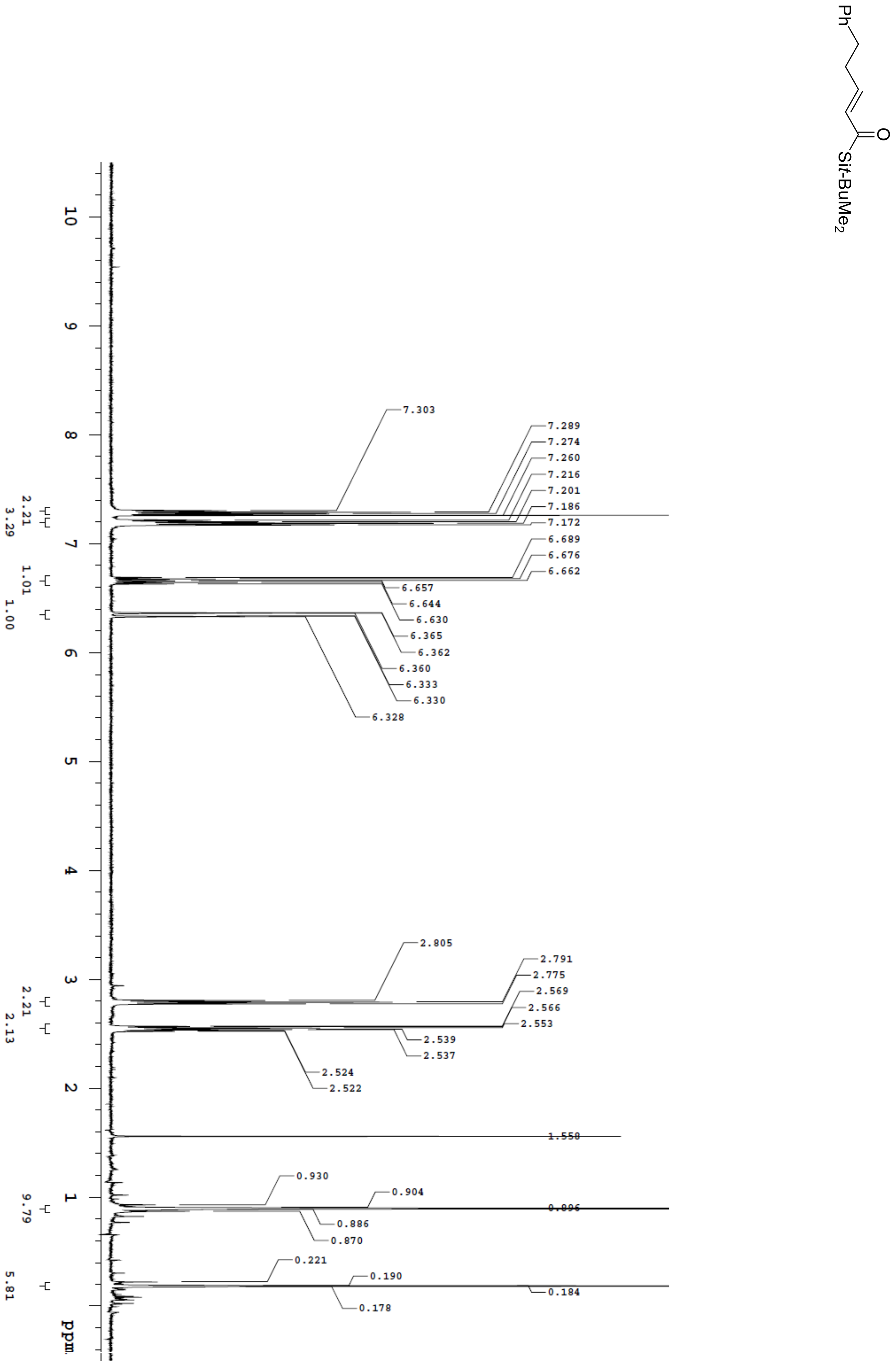

Supplementary Figure 41. ^{1}H NMR Spectrum of

(*E*)-1-(*tert*-Butyldimethylsilyl)-5-phenylpent-2-en-1-one (**1u**)

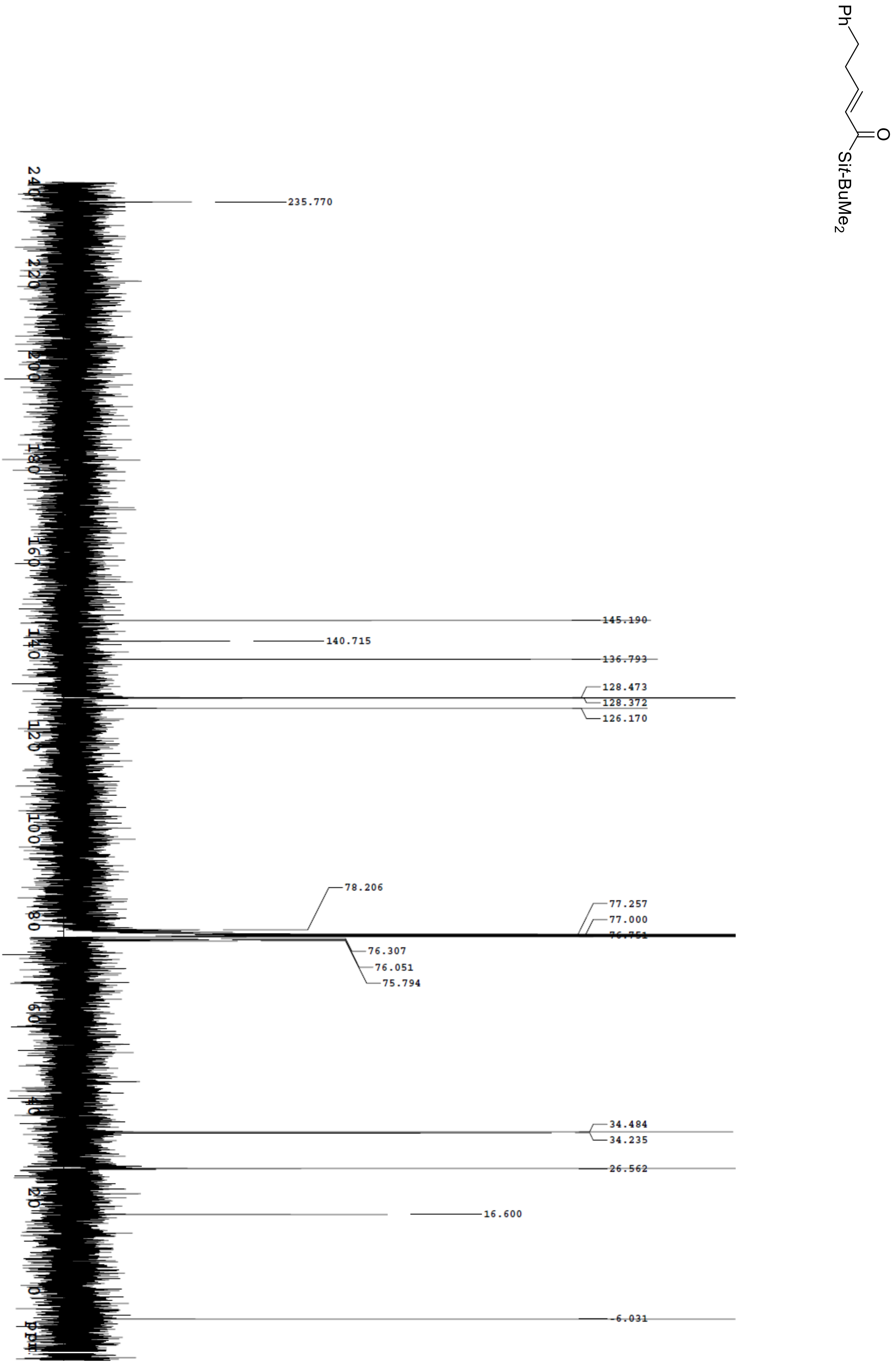

Supplementary Figure 42. ^{13}C NMR Spectrum of

(*E*)-1-(*tert*-Butyldimethylsilyl)-5-phenylpent-2-en-1-one (**1u**)

NMR Spectra (^{1}H, ^{13}C) of Products

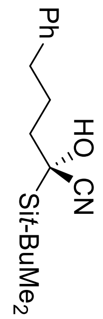

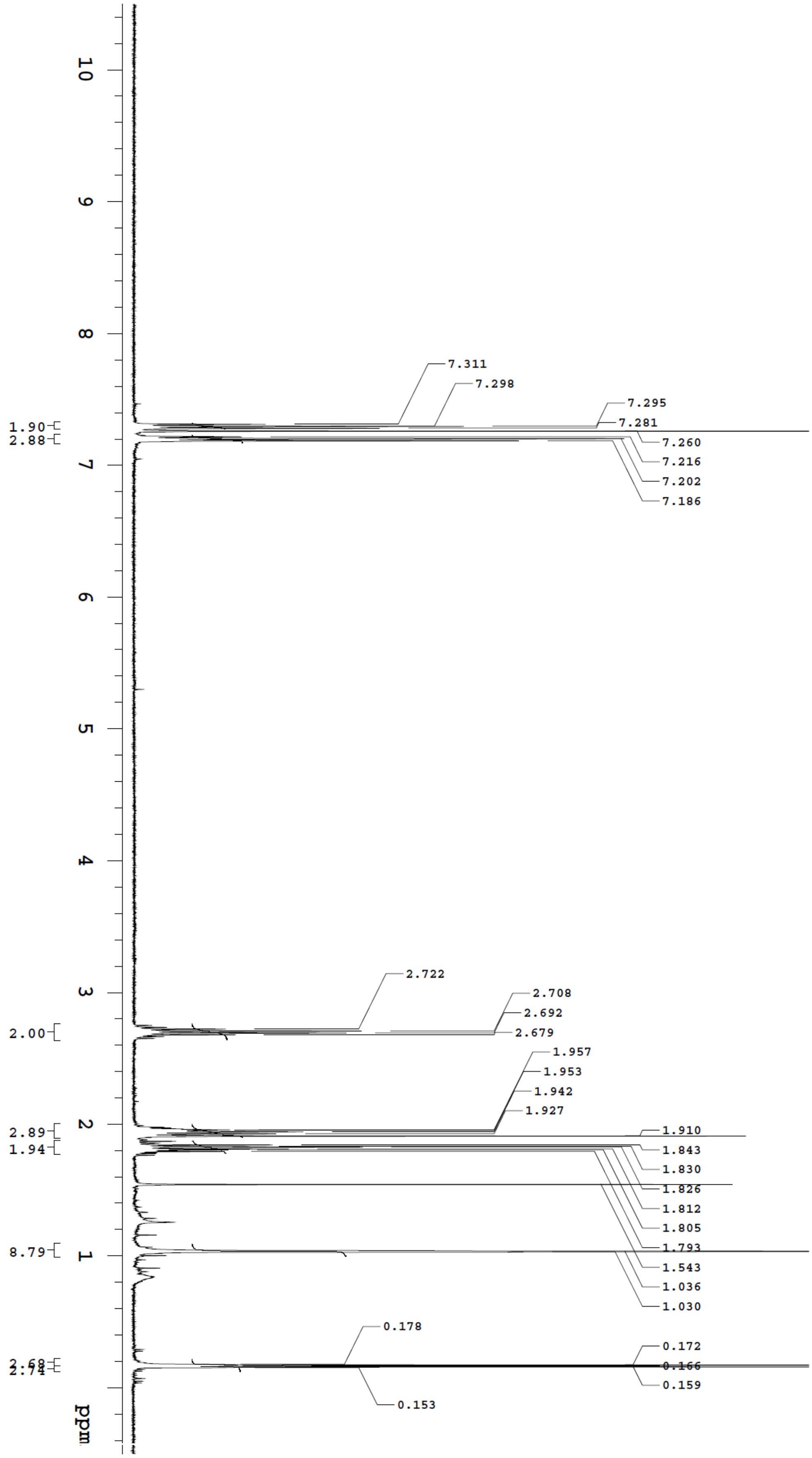

Supplementary Figure 43. ^{1}H NMR Spectrum of (*S*)-2-(*tert*-Butyldimethylsilyl)-2-hydroxy-5-phenylpentanenitrile (**2a**)

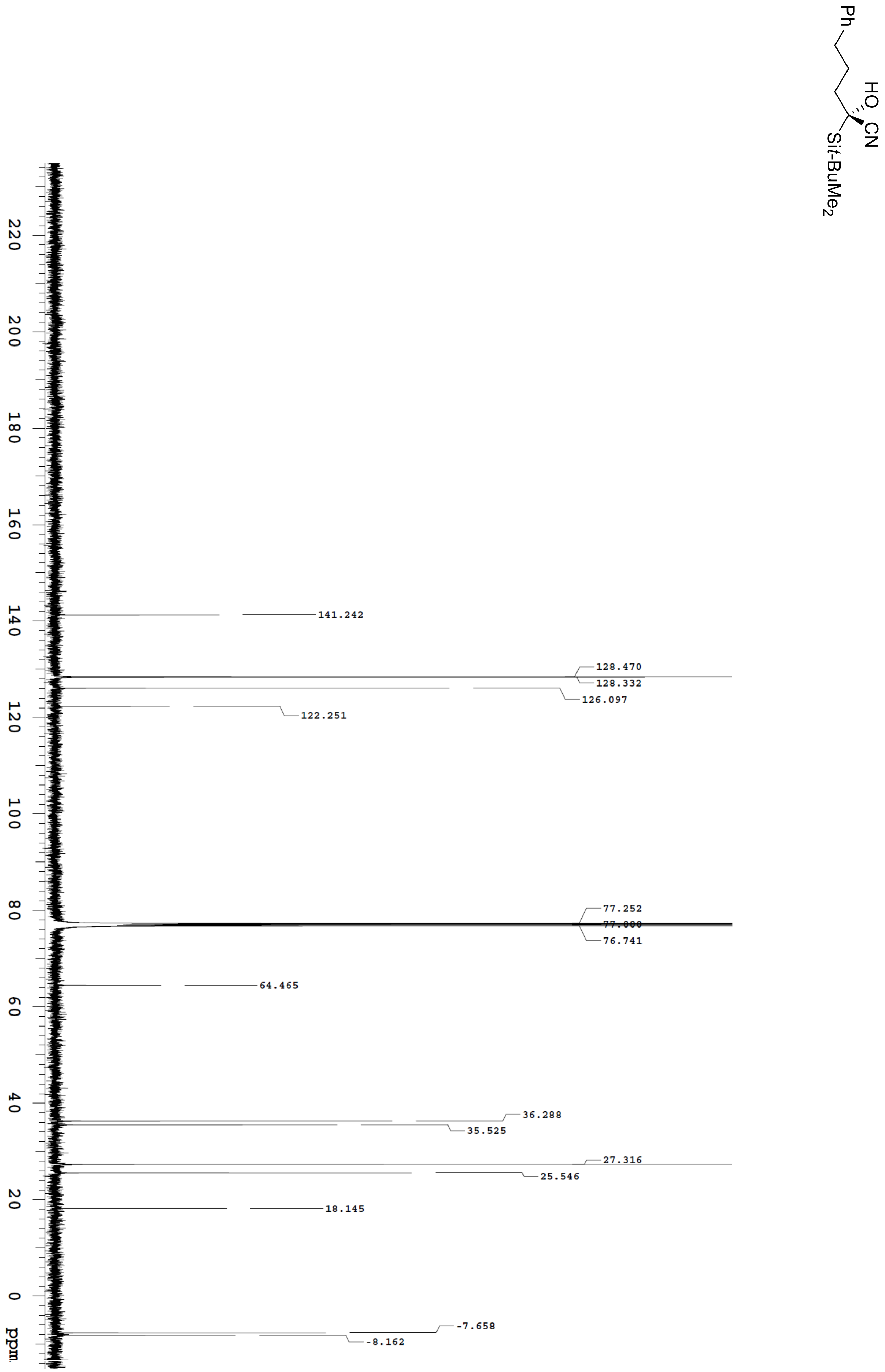

Supplementary Figure 44. ^{13}C NMR Spectrum of (*S*)-2-(*tert*-Butyldimethylsilyl)-2-hydroxy-5-phenylpentanenitrile (**2a**)

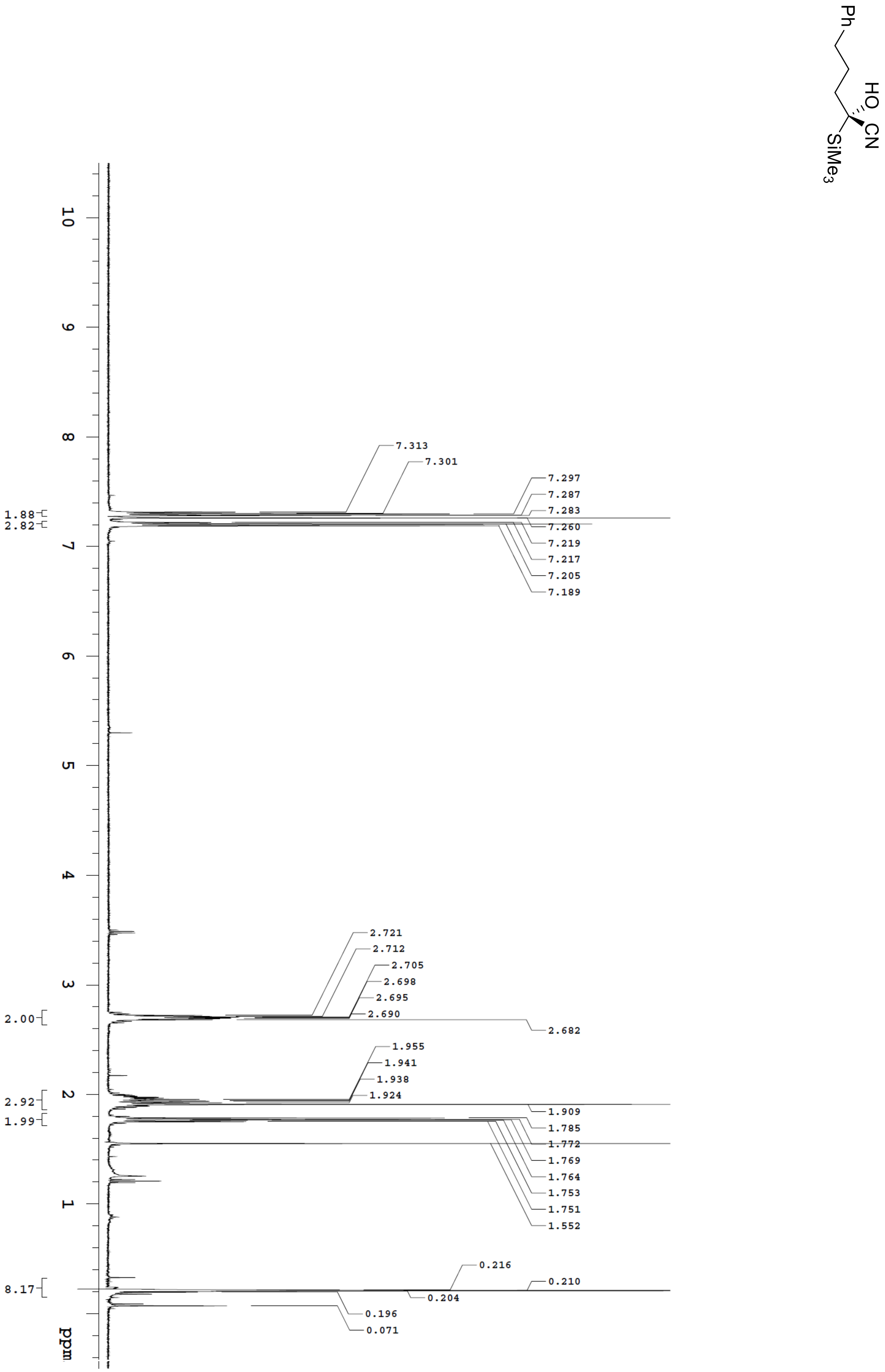

Supplementary Figure 45. ^{1}H NMR Spectrum of 2-Hydroxy-5-phenyl-2-(trimethylsilyl)pentanenitrile (**2b**)

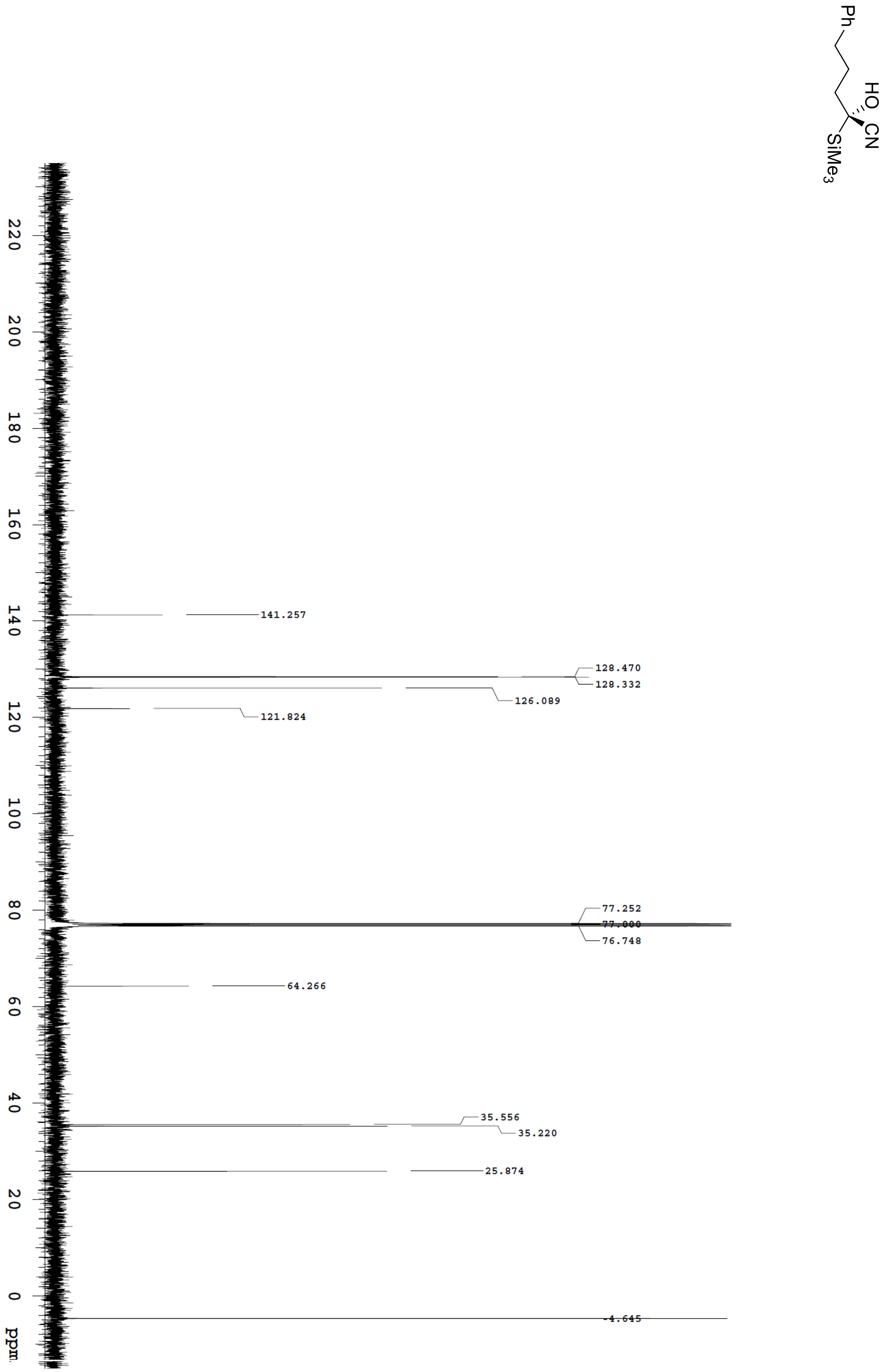

Supplementary Figure 46. ^{13}C NMR Spectrum of 2-Hydroxy-5-phenyl-2-(trimethylsilyl)pentanenitrile (**2b**)

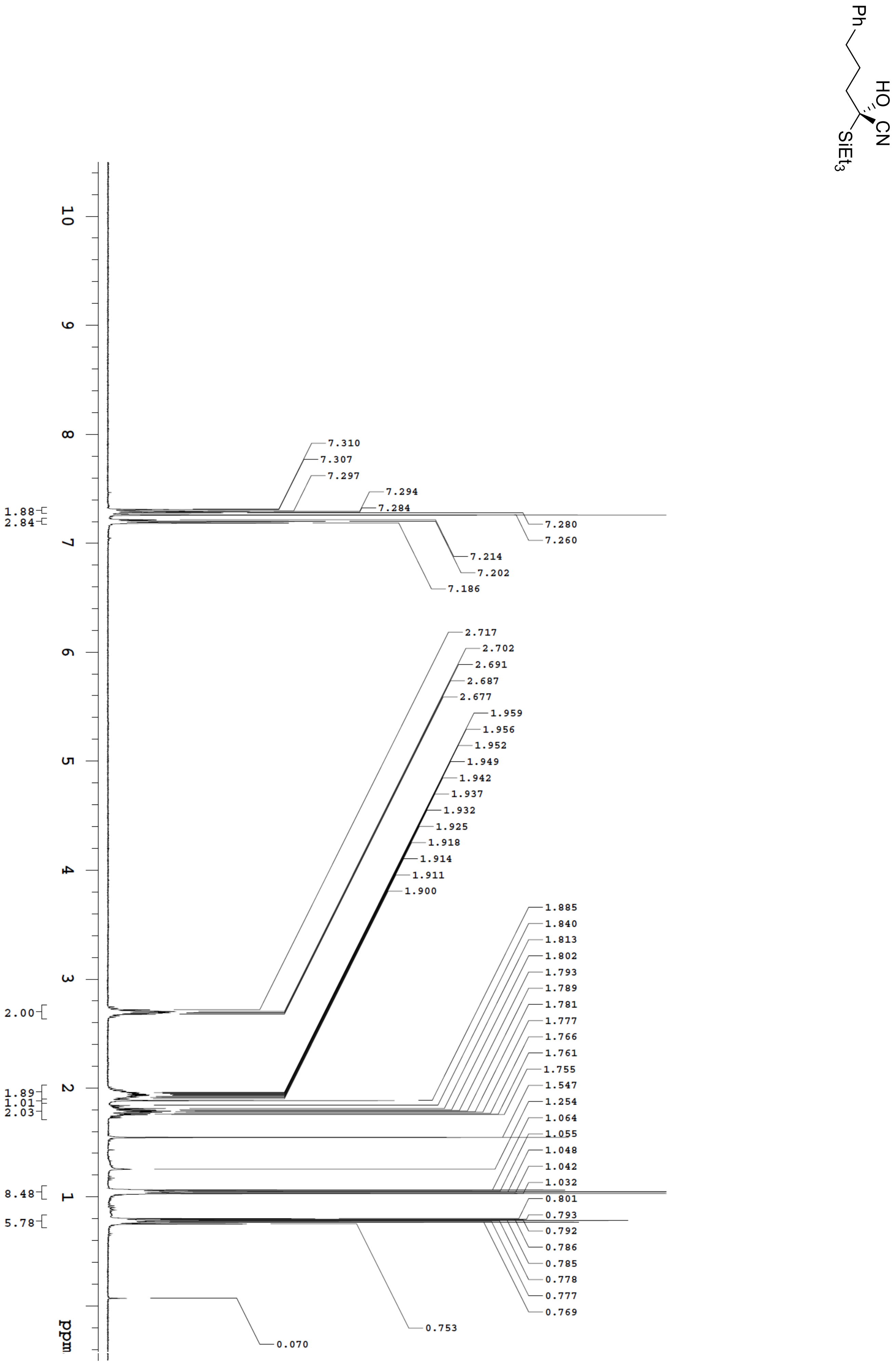

Supplementary Figure 47. ^{1}H NMR Spectrum of 2-Hydroxy-5-phenyl-2-(triethylsilyl)pentanenitrile (**2c**)

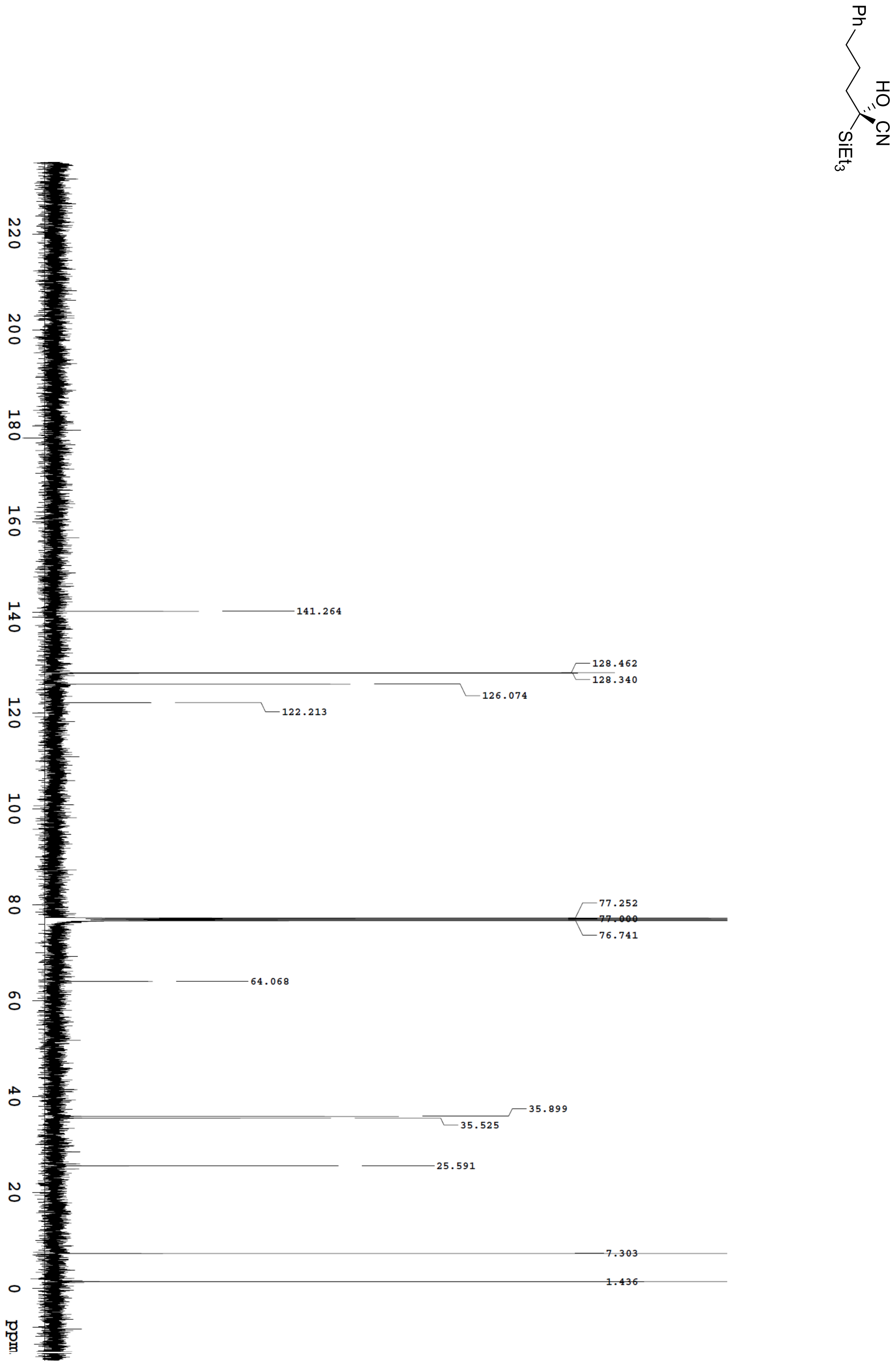

Supplementary Figure 48. ^{13}C NMR Spectrum of 2-Hydroxy-5-phenyl-2-(triethylsilyl)pentanenitrile (**2c**)

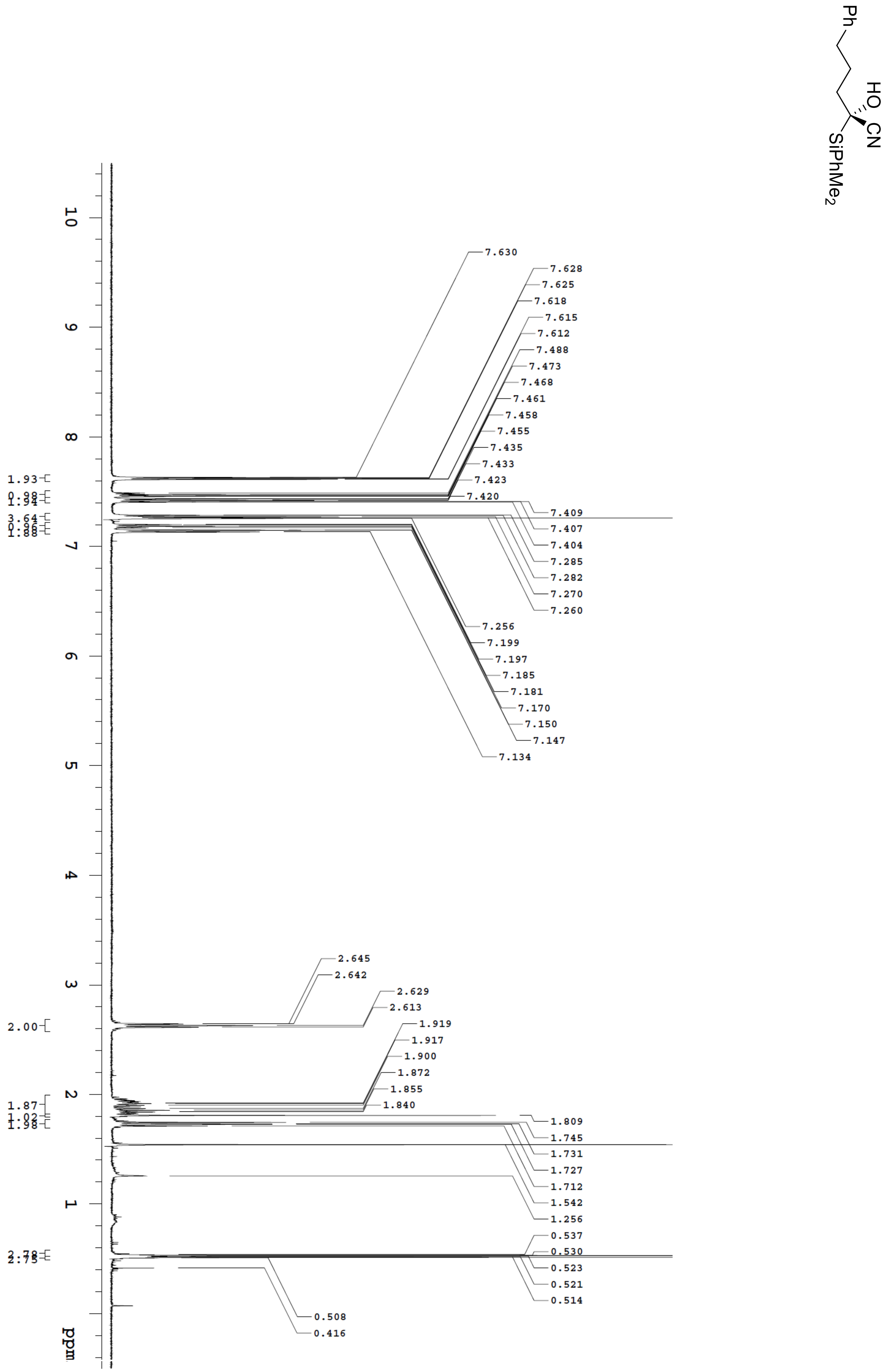

Supplementary Figure 49. ^{1}H NMR Spectrum of 2-(Dimethyl(phenyl)silyl)-2-hydroxy-5-phenylpentanenitrile (**2d**)

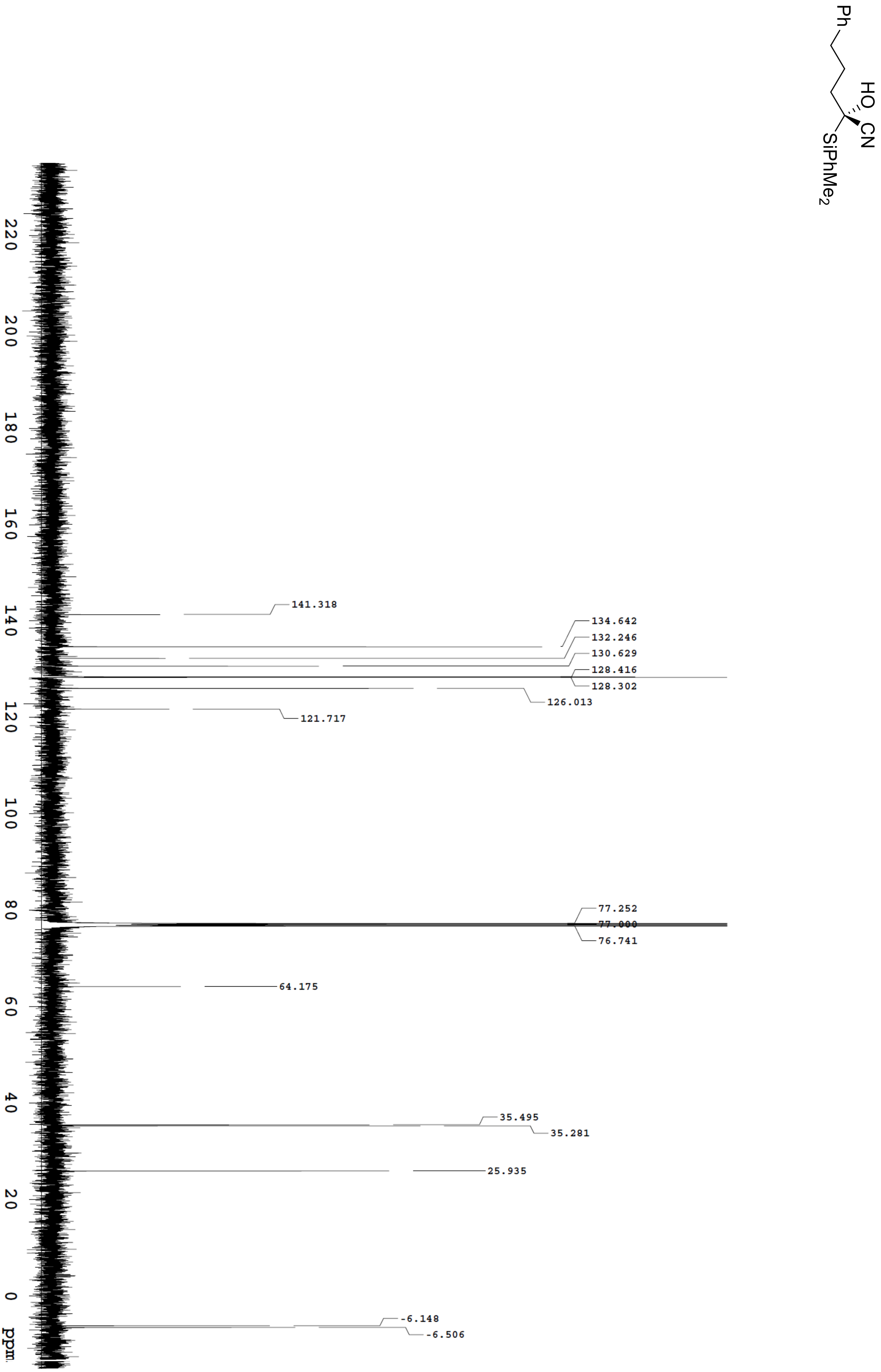

Supplementary Figure 50. ^{13}C NMR Spectrum of 2-(Dimethyl(phenyl)silyl)-2-hydroxy-5-phenylpentanenitrile (**2d**)

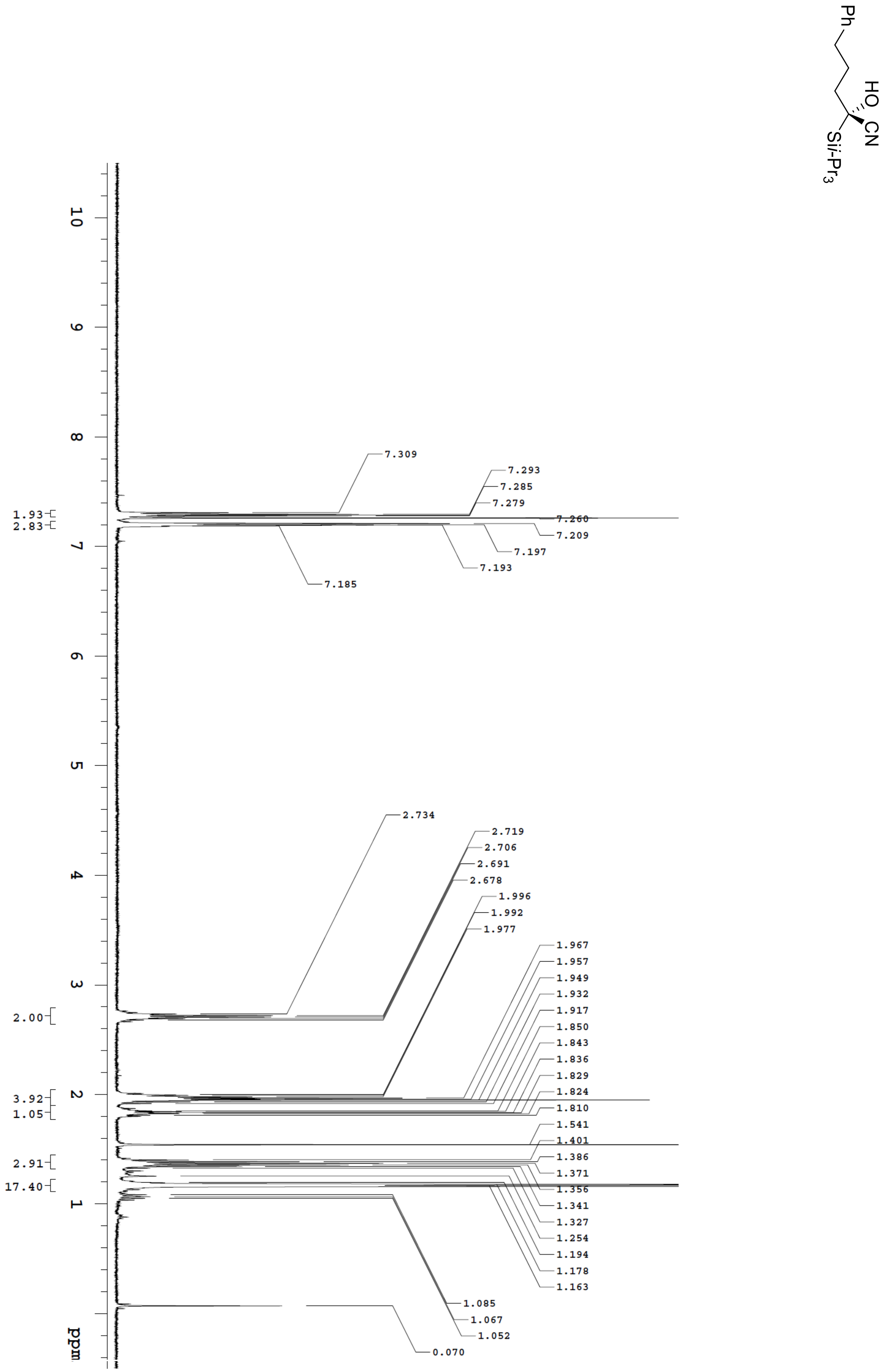

Supplementary Figure 51. ^{1}H NMR Spectrum of

2-Hydroxy-5-phenyl-2-(triisopropylsilyl)pentanenitrile (**2e**)

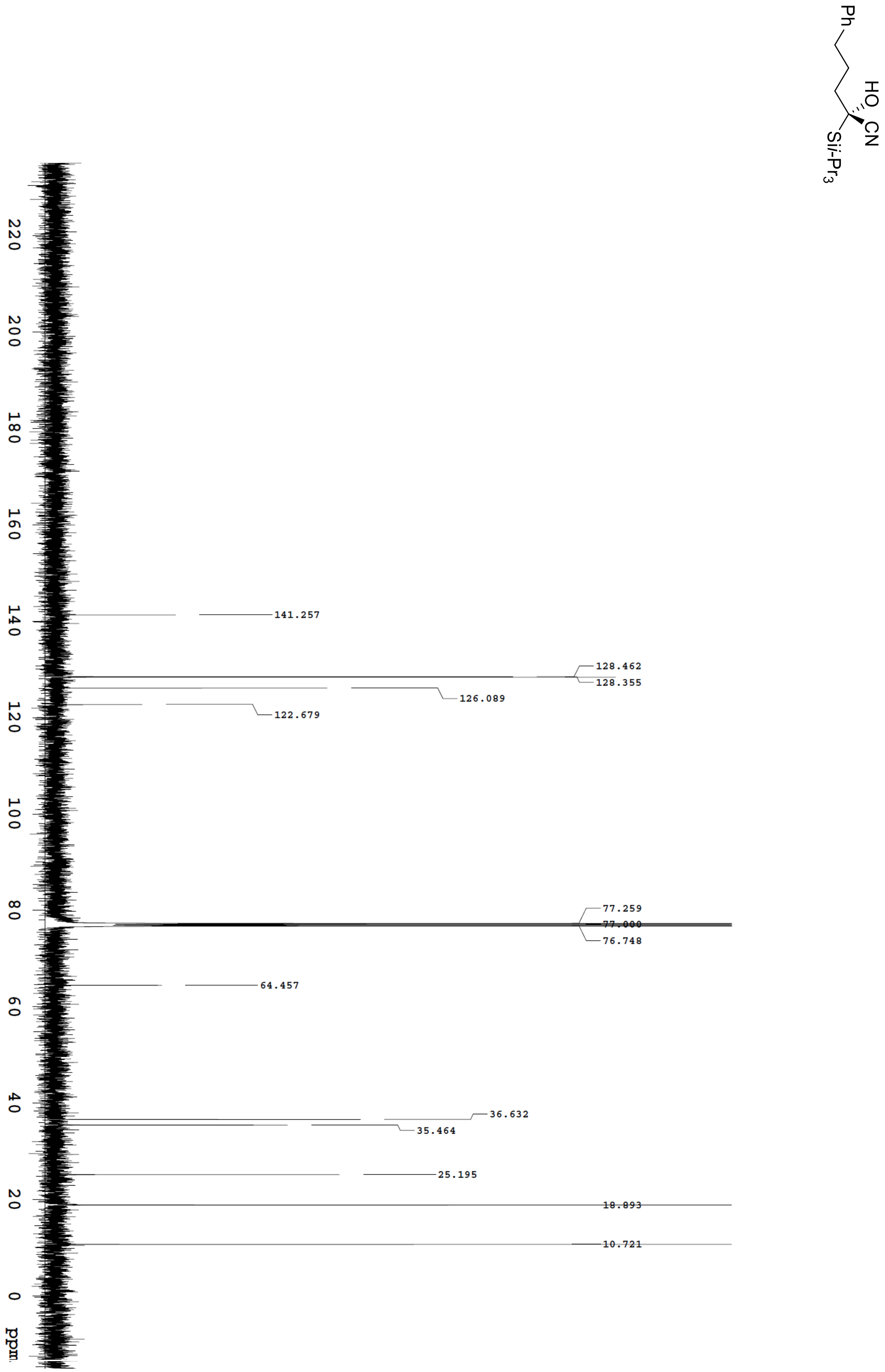

Supplementary Figure 52. ^{13}C NMR Spectrum of 2-Hydroxy-5-phenyl-2-(triisopropylsilyl)pentanenitrile (**2e**)

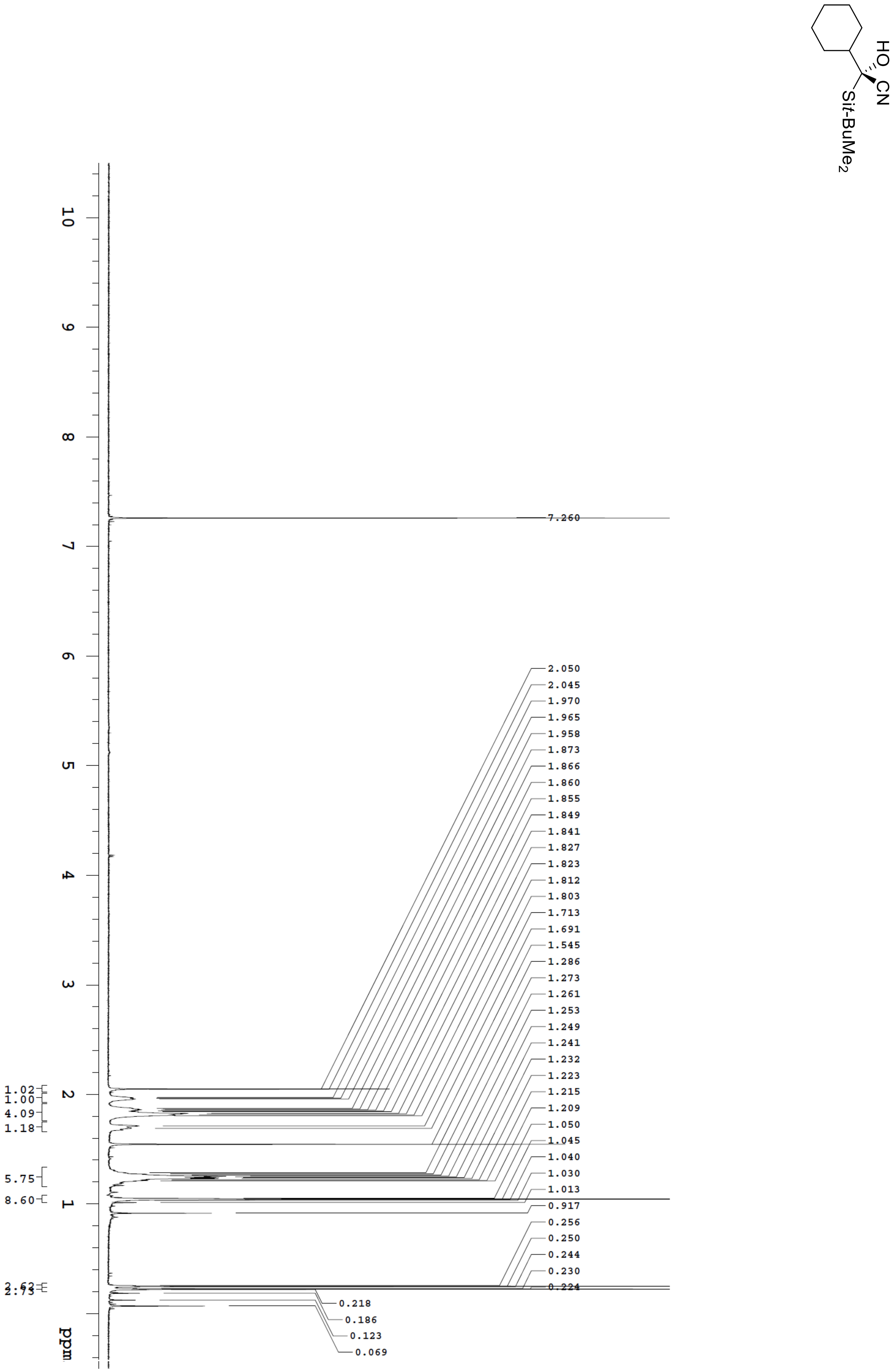

Supplementary Figure 53. ^{1}H NMR Spectrum of 2-(*tert*-Butyldimethylsilyl)-2-cyclohexyl-2-hydroxyacetonitrile (**2f**)

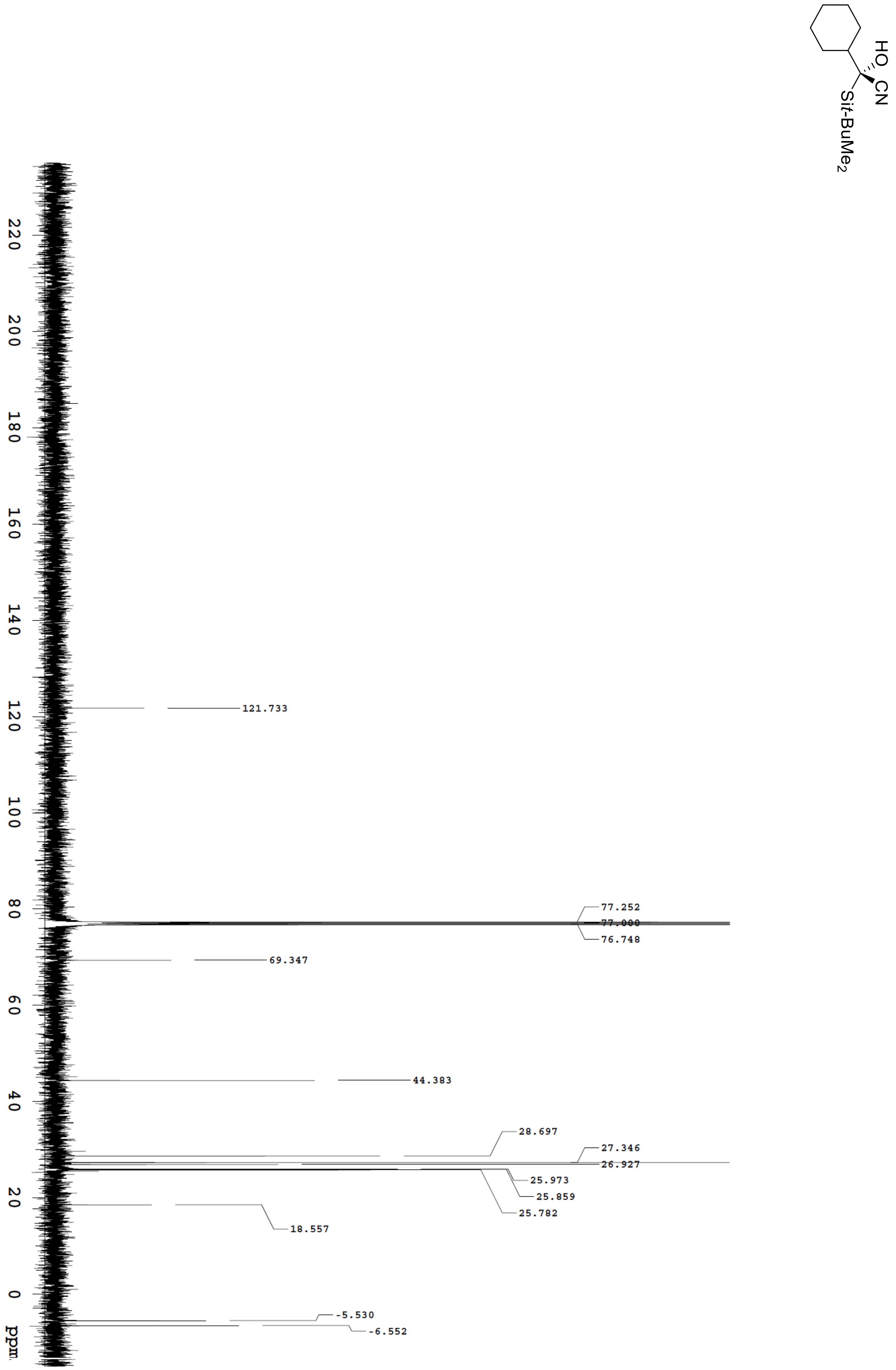

Supplementary Figure 54. ^{13}C NMR Spectrum of 2-(*tert*-Butyldimethylsilyl)-2-cyclohexyl-2-hydroxyacetonitrile (**2f**)

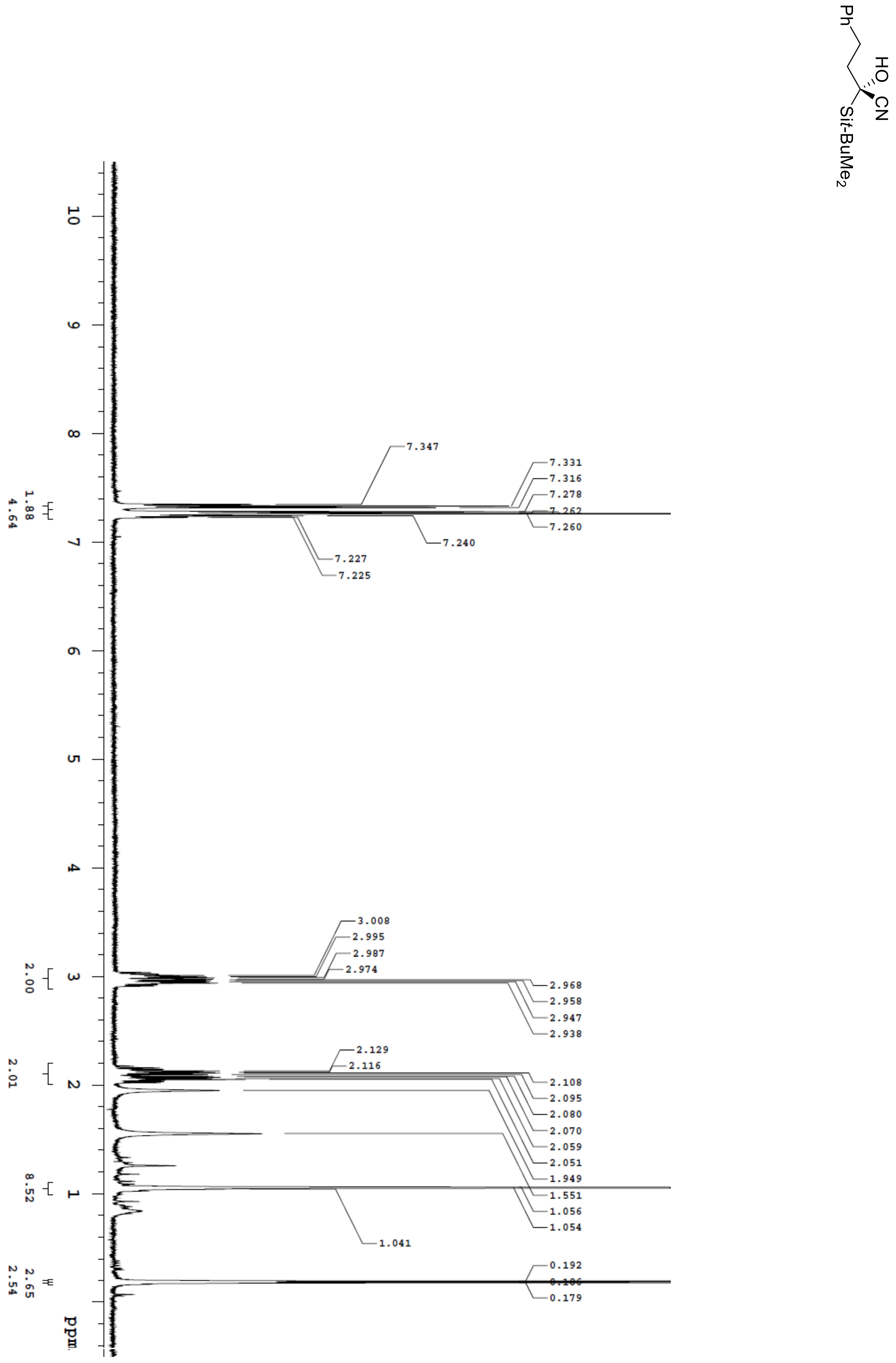

Supplementary Figure 55. ^{1}H NMR Spectrum of 2-(*tert*-Butyldimethylsilyl)-2-hydroxy-4-phenylbutanenitrile (**2g**)

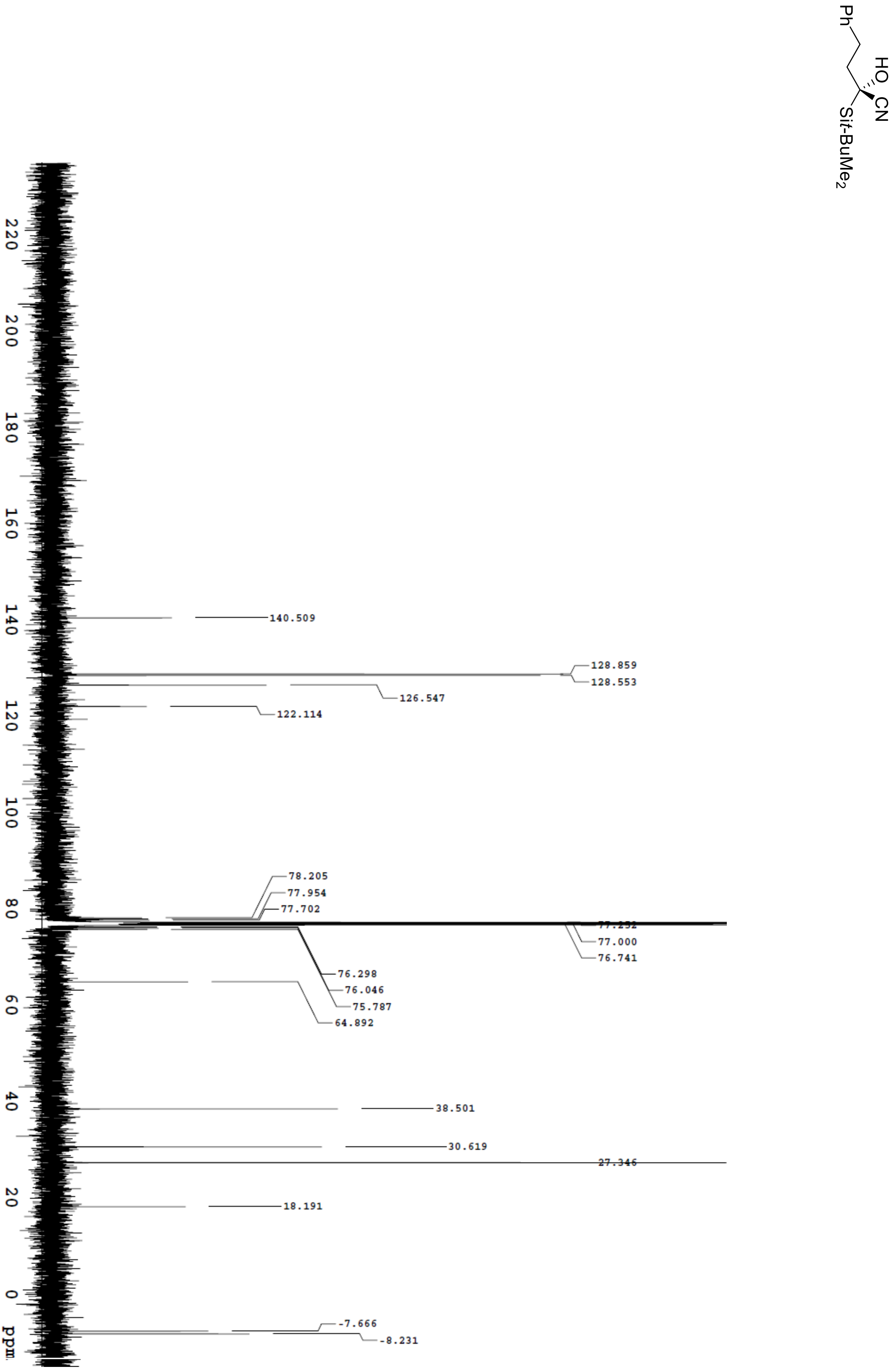

Supplementary Figure 56. ^{13}C NMR Spectrum of

2-(*tert*-Butyldimethylsilyl)-2-hydroxy-4-phenylbutanenitrile (**2g**)

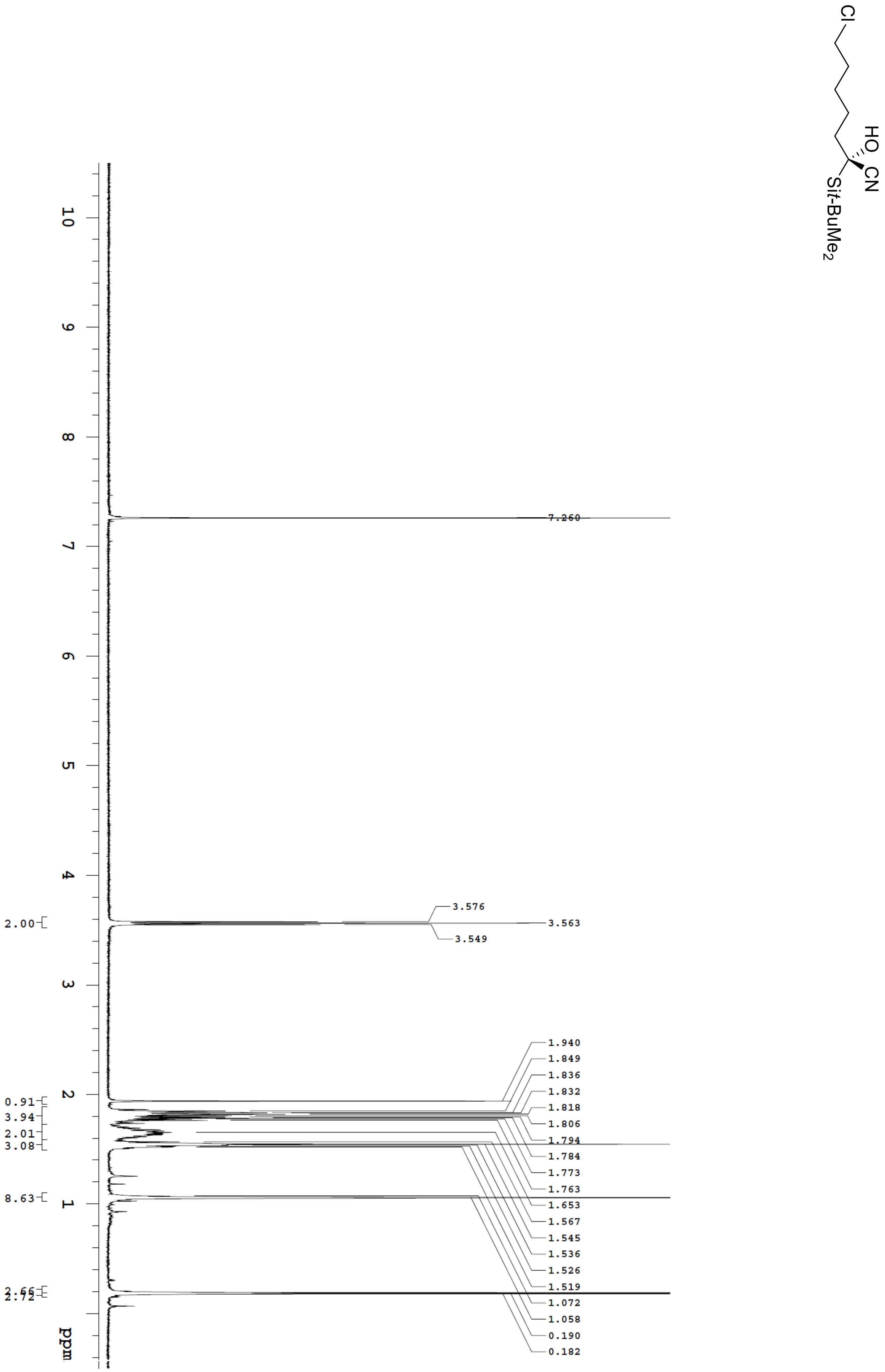

Supplementary Figure 57. ^{1}H NMR Spectrum of 2-(*tert*-Butyldimethylsilyl)-7-chloro-2-hydroxyheptanenitrile (**2h**)

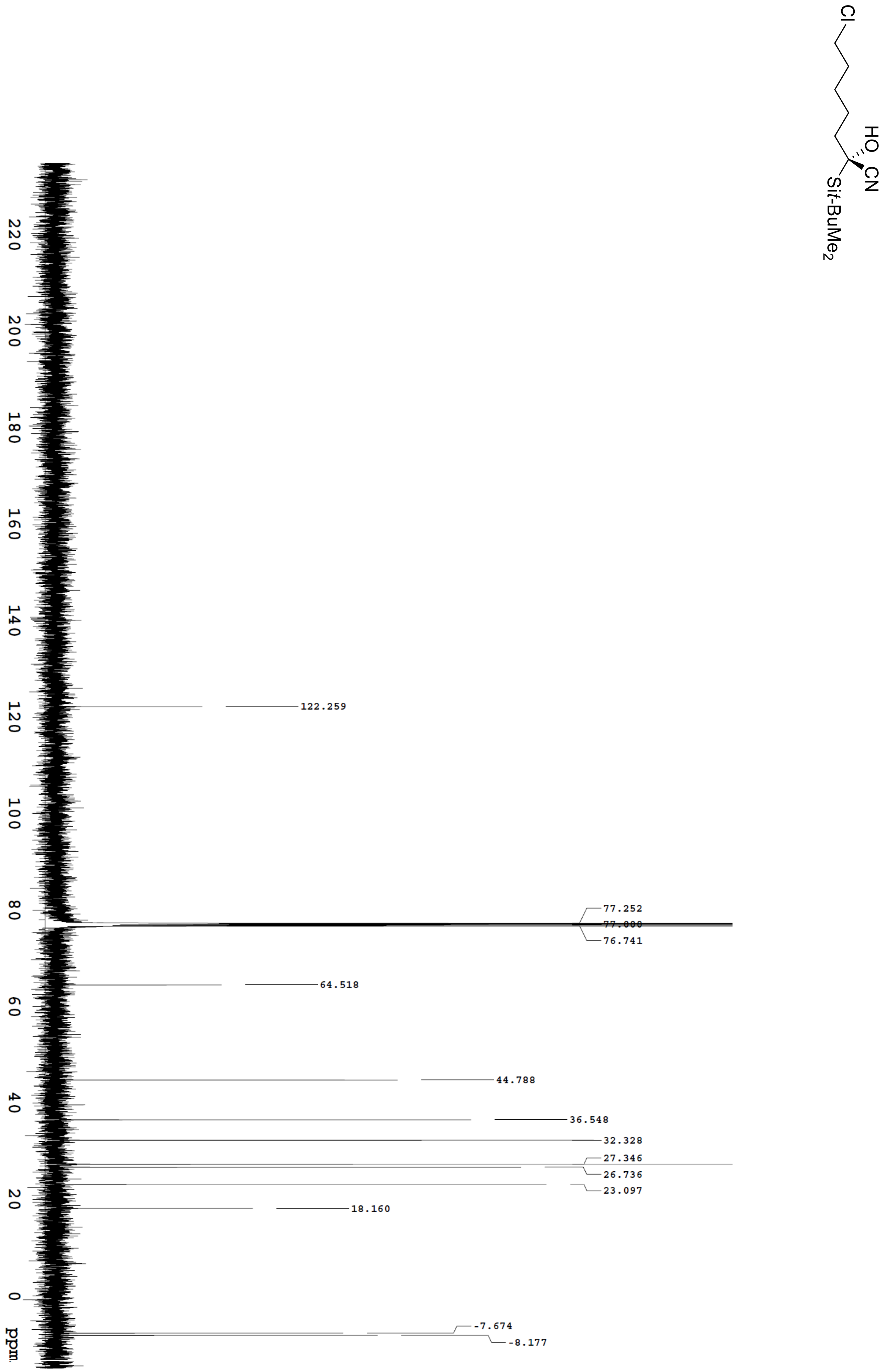

Supplementary Figure 58. ^{13}C NMR Spectrum of 2-(*tert*-Butyldimethylsilyl)-7-chloro-2-hydroxyheptanenitrile (**2h**)

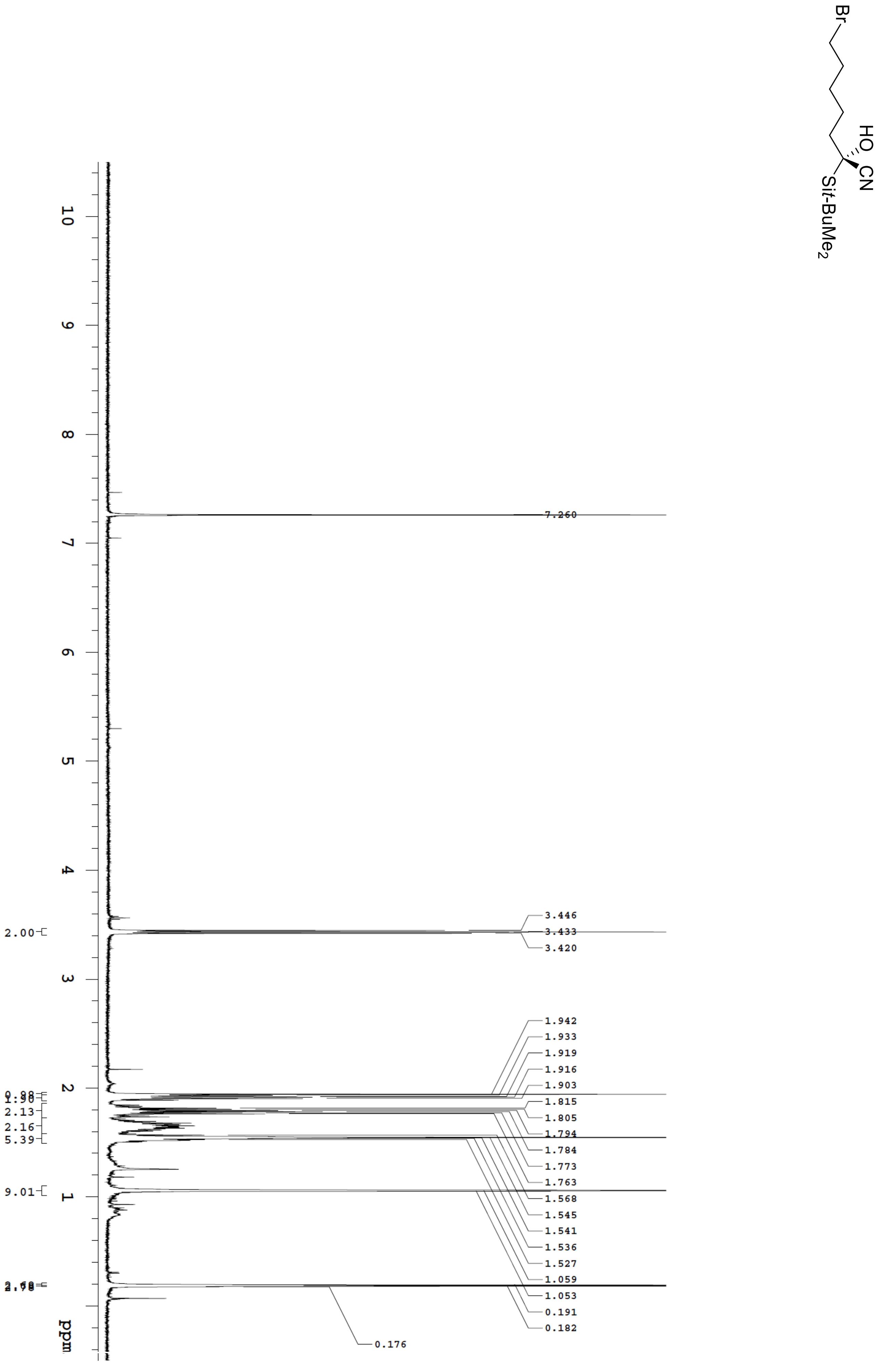

Supplementary Figure 59. ^{1}H NMR Spectrum of 7-Bromo-2-(*tert*-butyldimethylsilyl)-2-hydroxyheptanenitrile (**2i**)

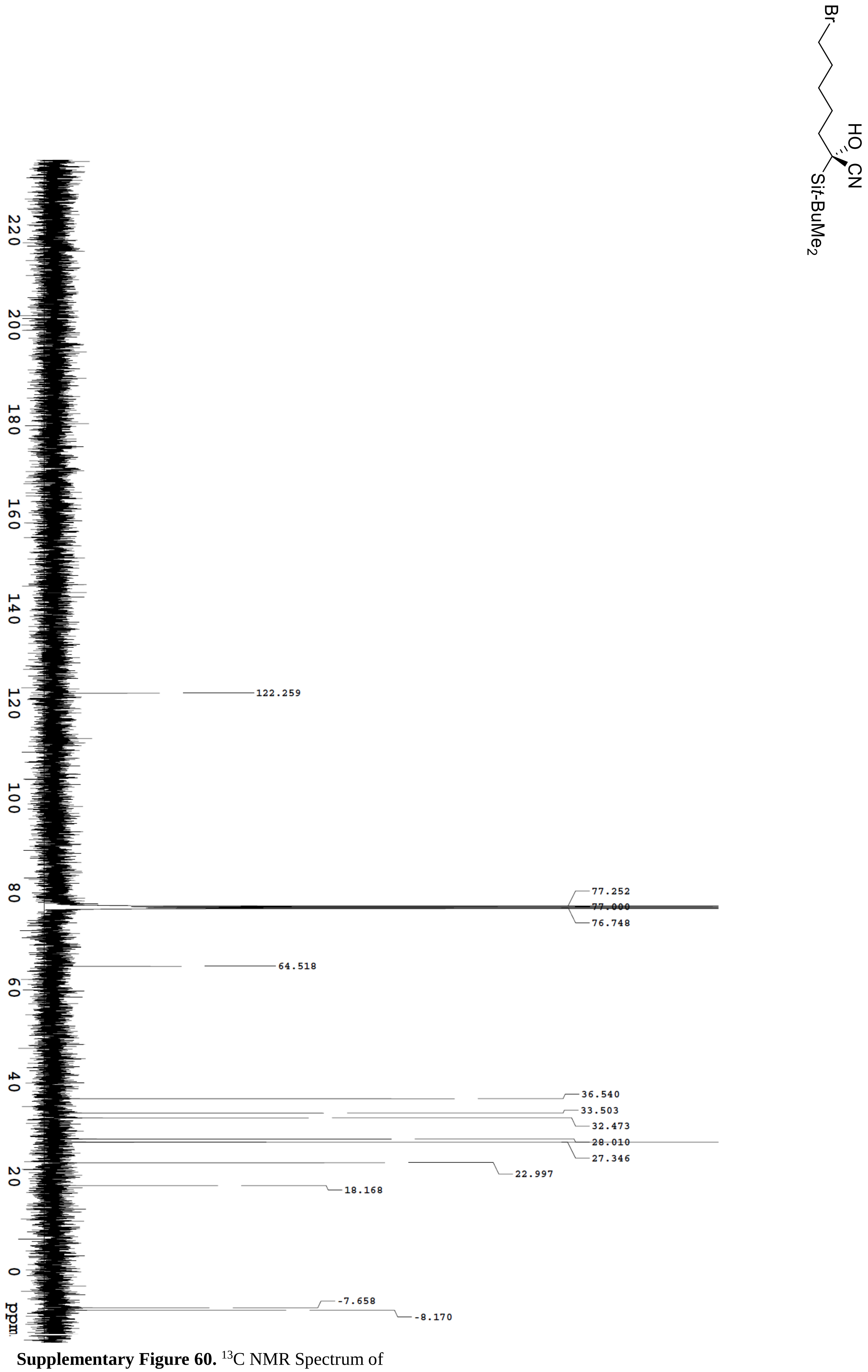

Supplementary Figure 60. ^{13}C NMR Spectrum of

7-Bromo-2-(*tert*-butyldimethylsilyl)-2-hydroxyheptanenitrile (**2i**)

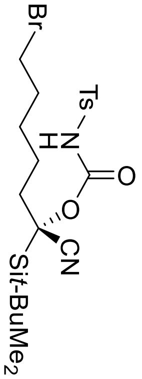

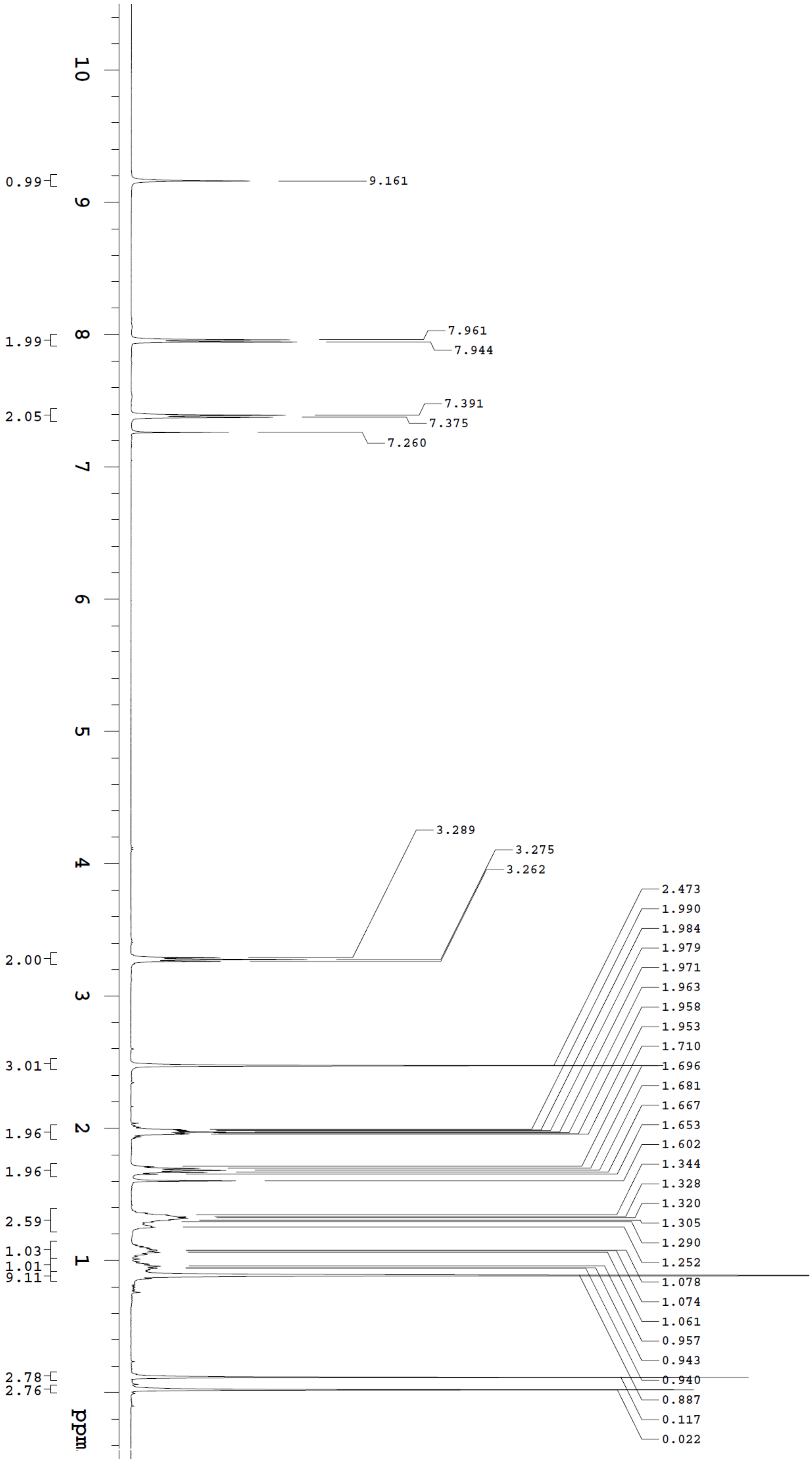

Supplementary Figure 61. ^{1}H NMR Spectrum of 6-Bromo-1-(*tert*-butyldimethylsilyl)-1-cyanohexyl tosylcarbamate (**2i'**)

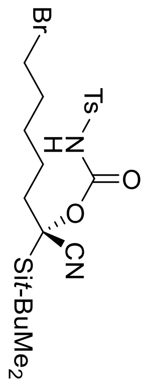

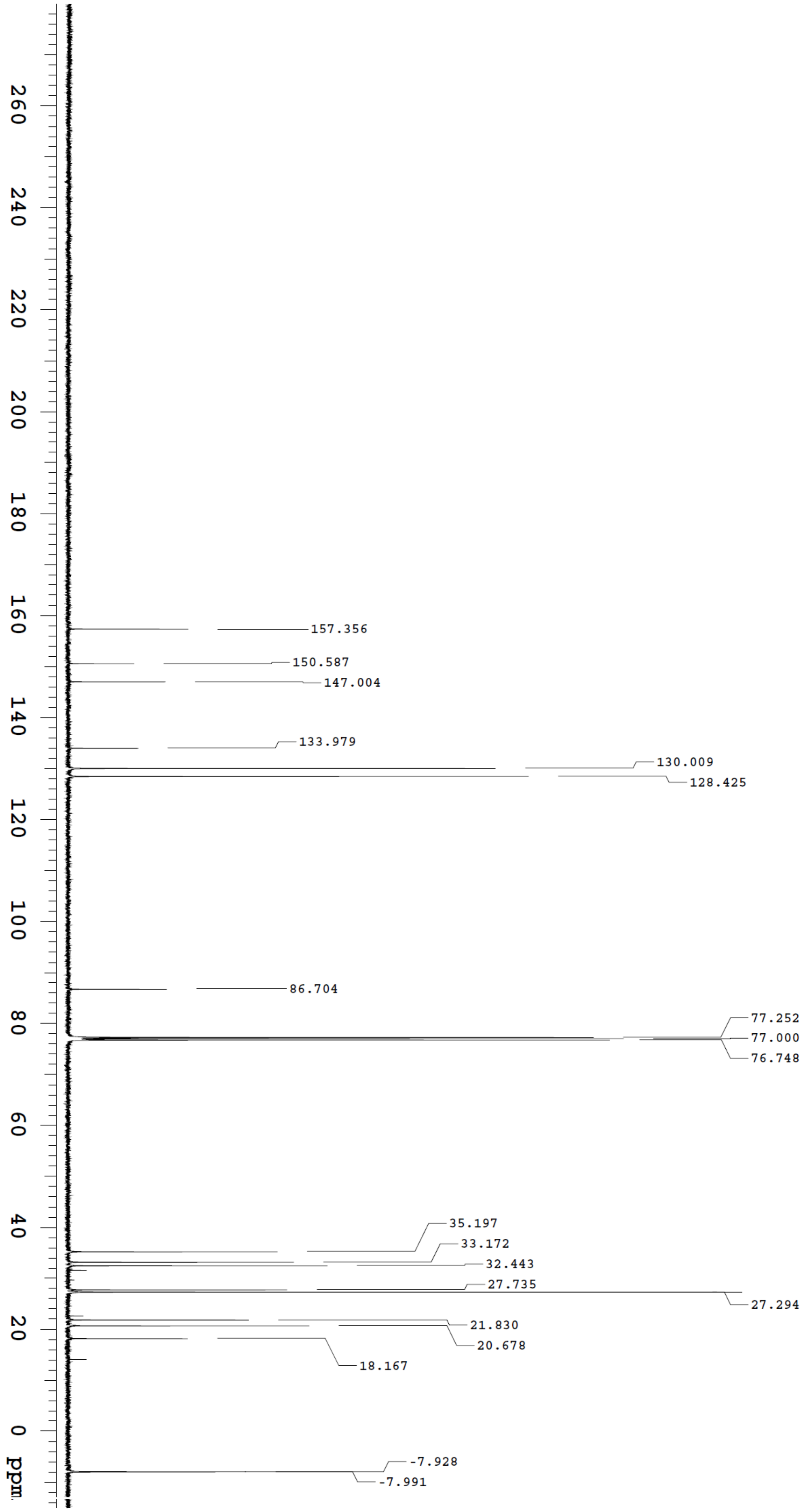

Supplementary Figure 62. ^{13}C NMR Spectrum of

6-Bromo-1-(*tert*-butyldimethylsilyl)-1-cyanohexyl tosylcarbamate (**2i'**)

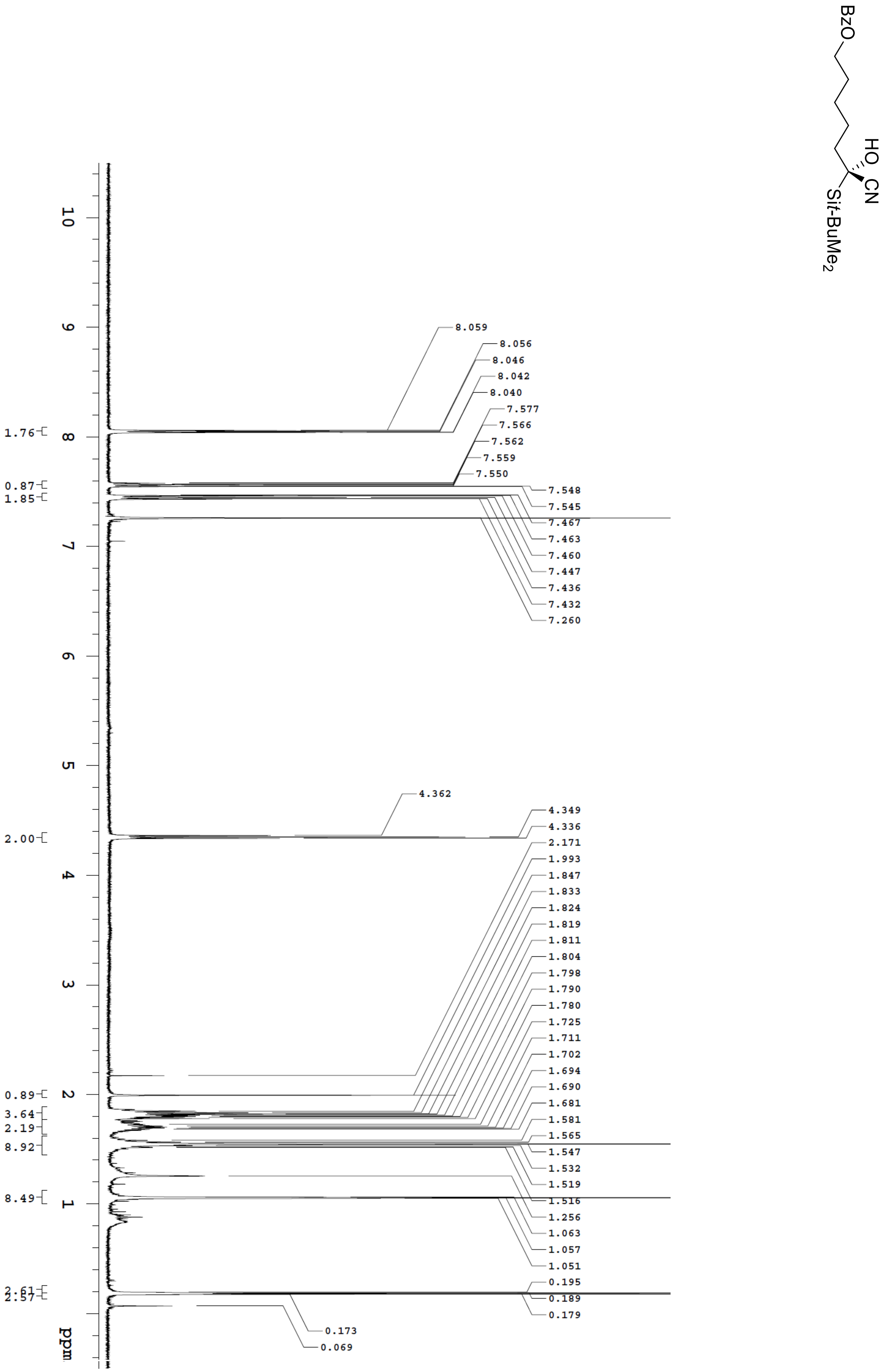

Supplementary Figure 63. ^{1}H NMR Spectrum of 6-(*tert*-Butyldimethylsilyl)-6-cyano-6-hydroxyhexyl benzoate (**2j**)

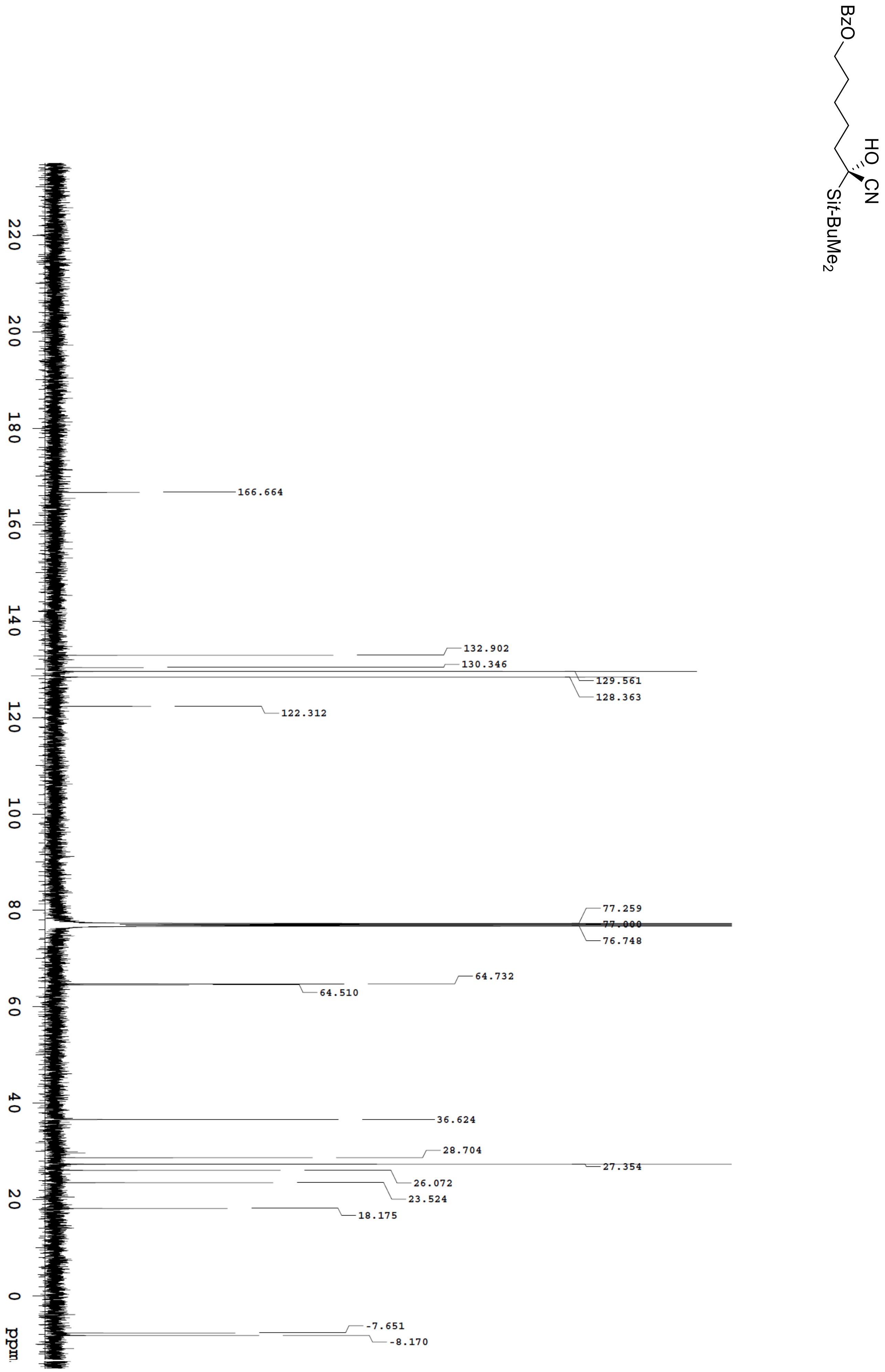

Supplementary Figure 64. ^{13}C NMR Spectrum of 6-(*tert*-Butyldimethylsilyl)-6-cyano-6-hydroxyhexyl benzoate (**2j**)

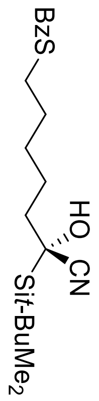

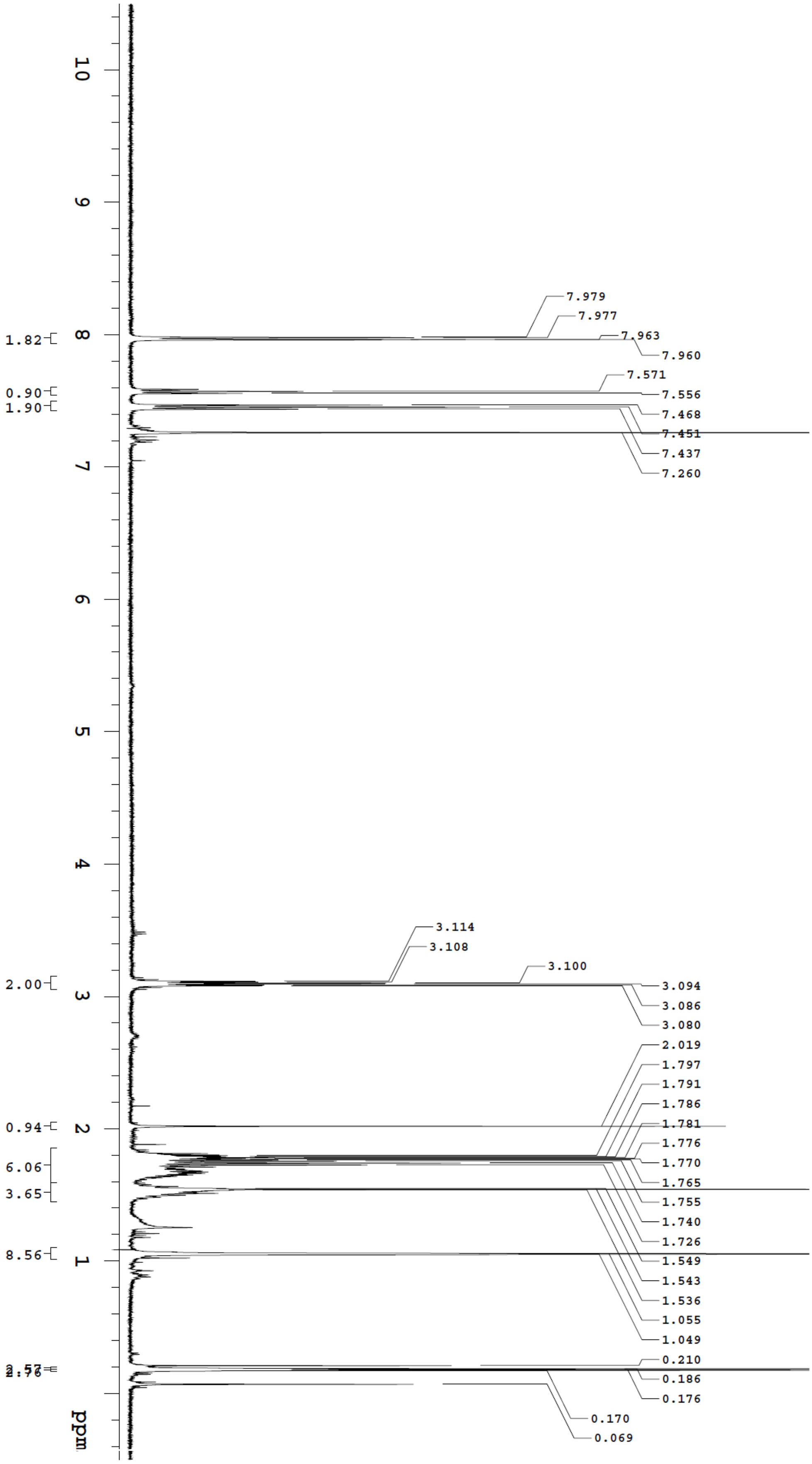

Supplementary Figure 65. ¹H NMR Spectrum of

S-(6-(*tert*-Butyldimethylsilyl)-6-cyano-6-hydroxyhexyl) benzothioate (**2k**)

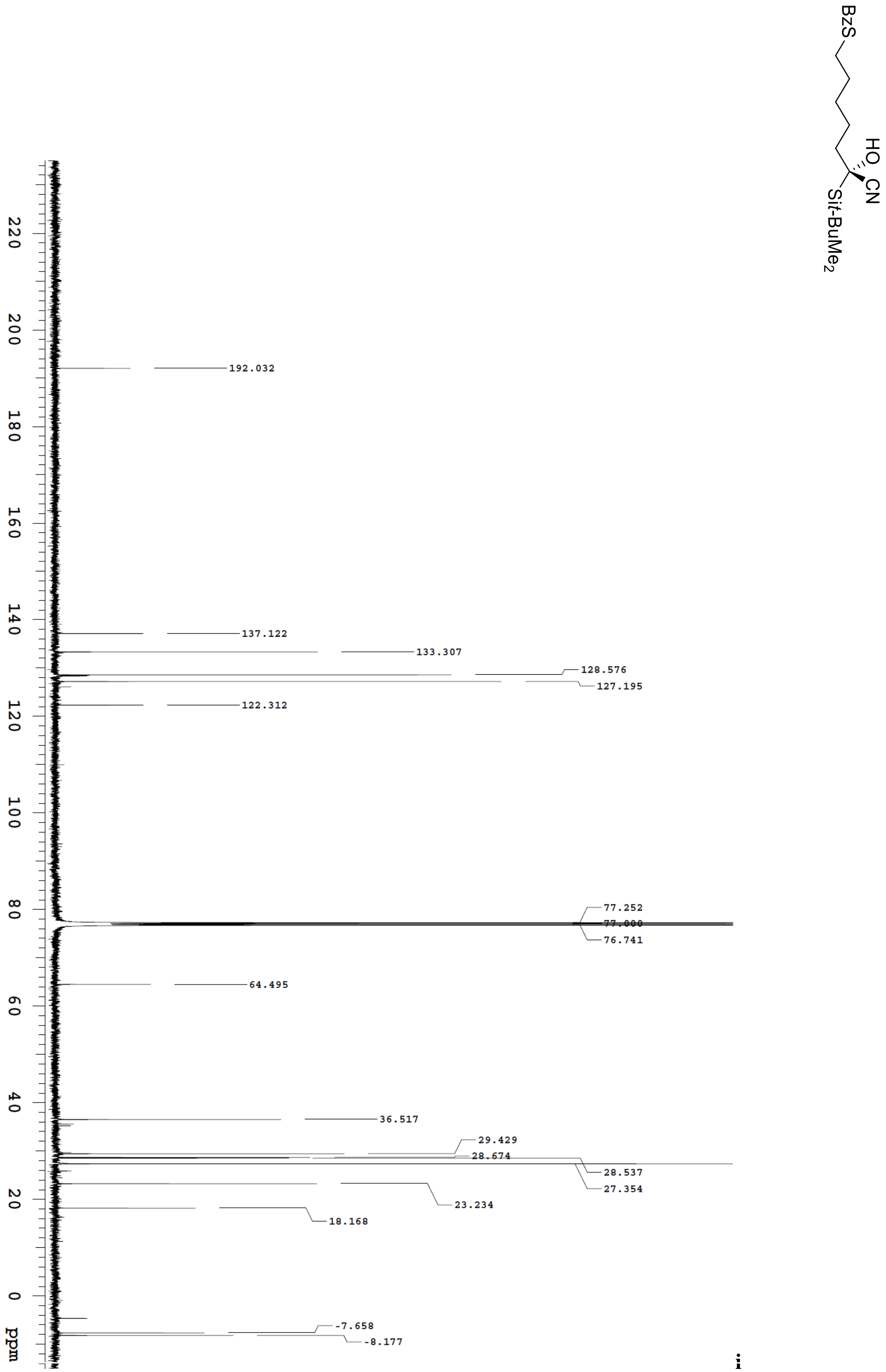

Supplementary Figure 66. ^{13}C NMR Spectrum of

S-(6-(*tert*-Butyldimethylsilyl)-6-cyano-6-hydroxyhexyl) benzothioate (**2k**)

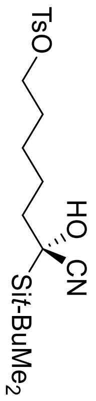

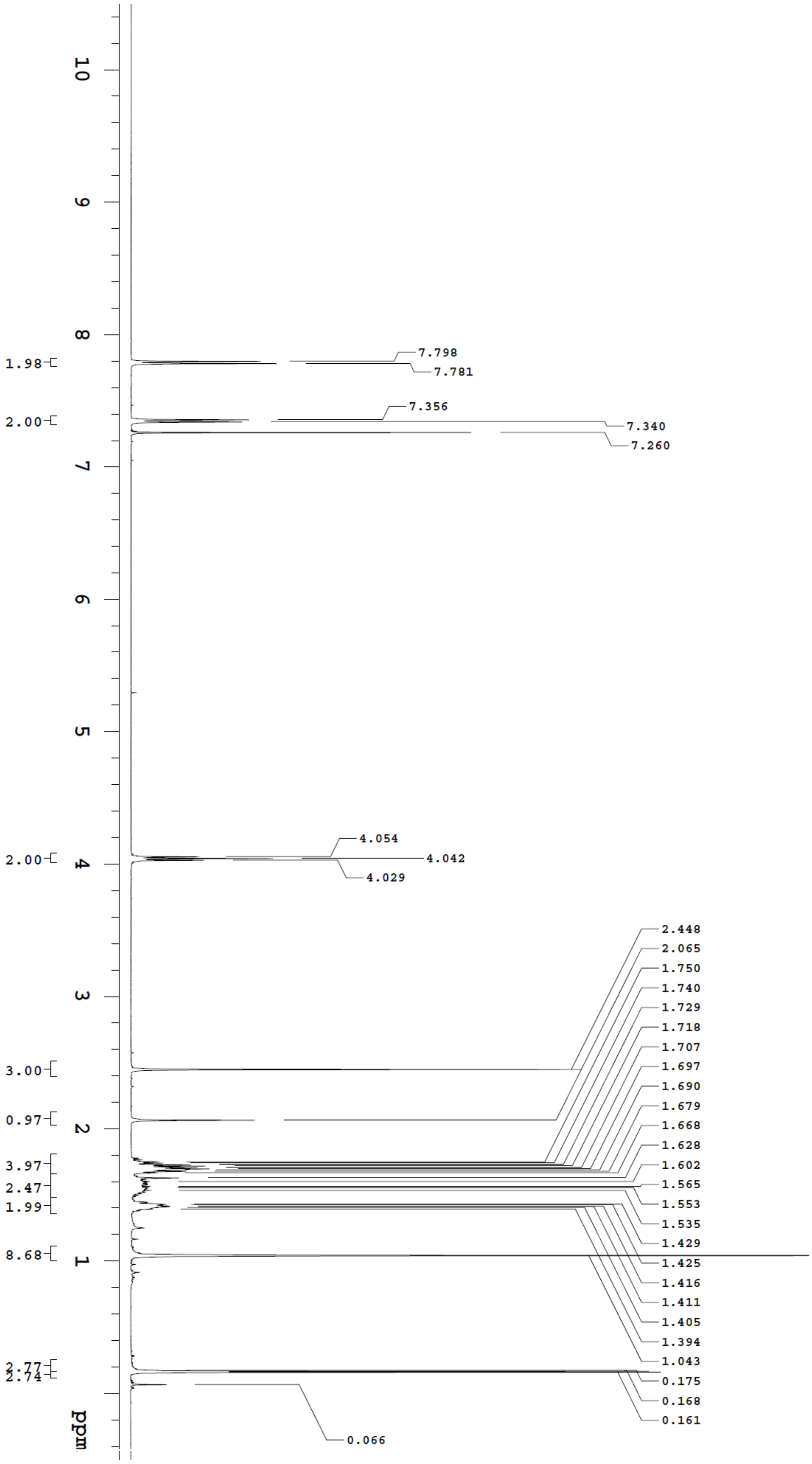

Supplementary Figure 67. ^{1}H NMR Spectrum of 6-(*tert*-Butyldimethylsilyl)-6-cyano-6-hydroxyhexyl 4-methylbenzenesulfonate (**2l**)

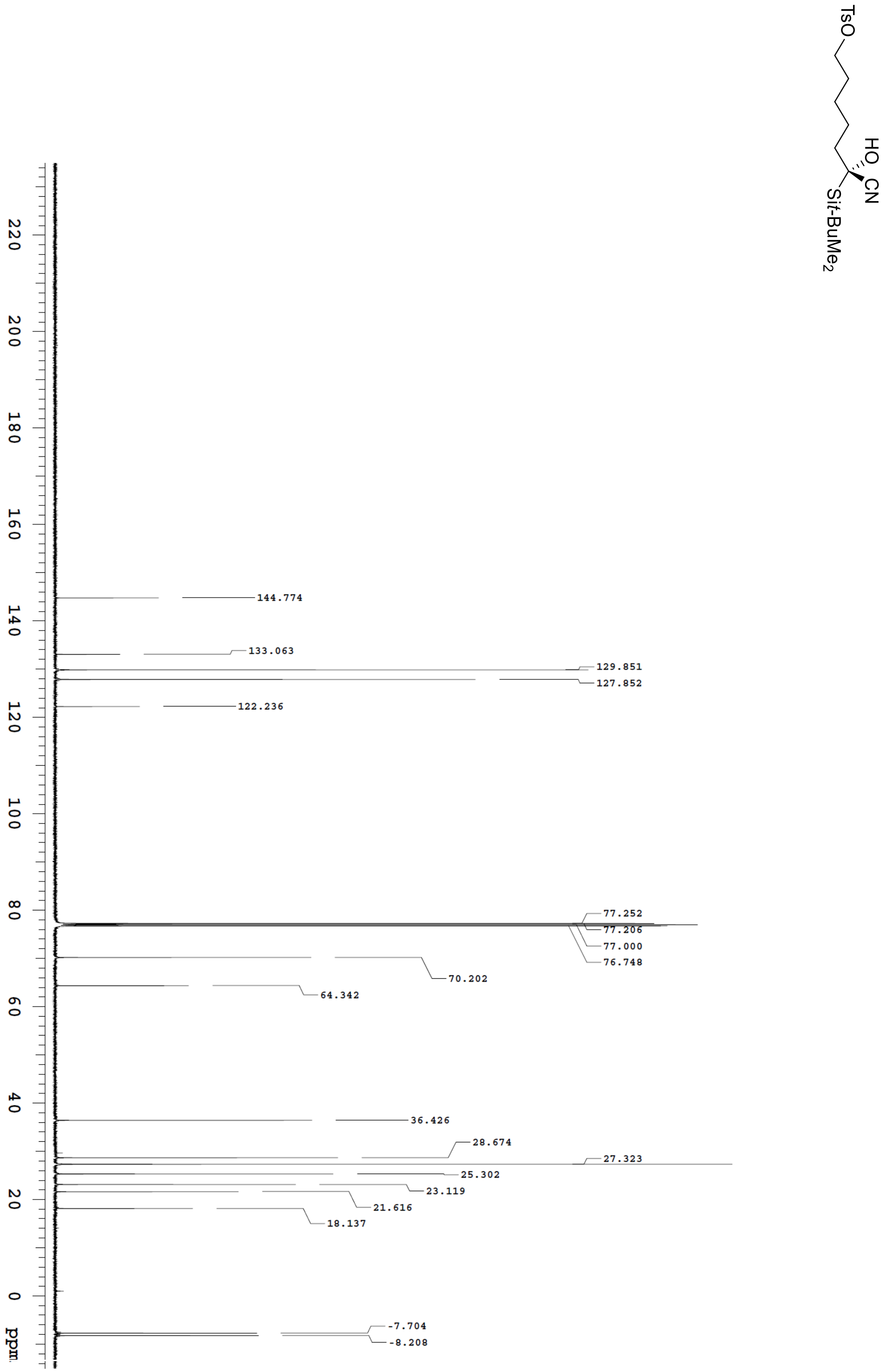

Supplementary Figure 68. ^{13}C NMR Spectrum of 6-(*tert*-Butyldimethylsilyl)-6-cyano-6-hydroxyhexyl 4-methylbenzenesulfonate (**2l**)

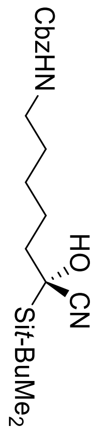

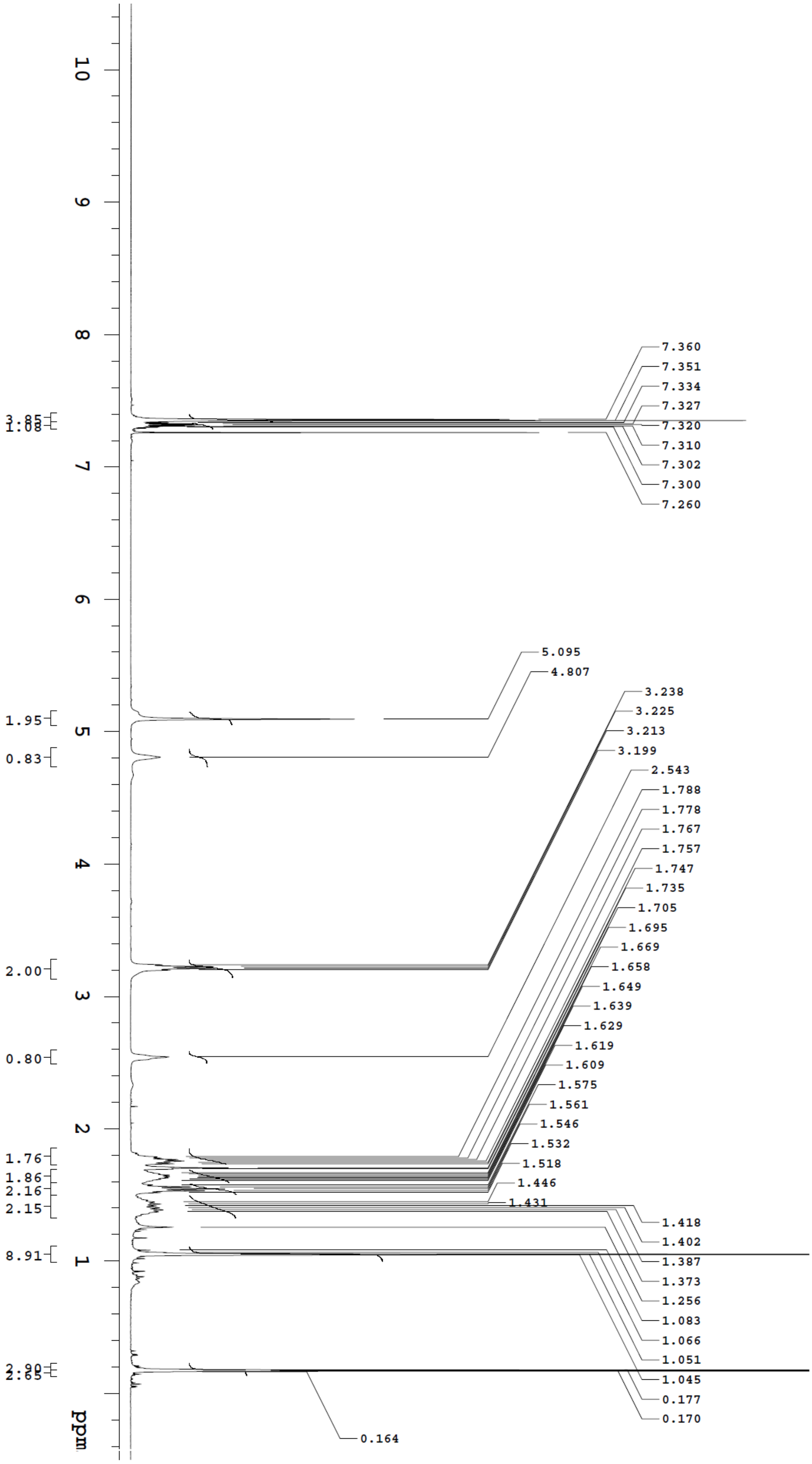

Supplementary Figure 69. ^{1}H NMR Spectrum of

Benzyl (6-(*tert*-butyldimethylsilyl)-6-cyano-6-hydroxyhexyl)carbamate (**2m**)

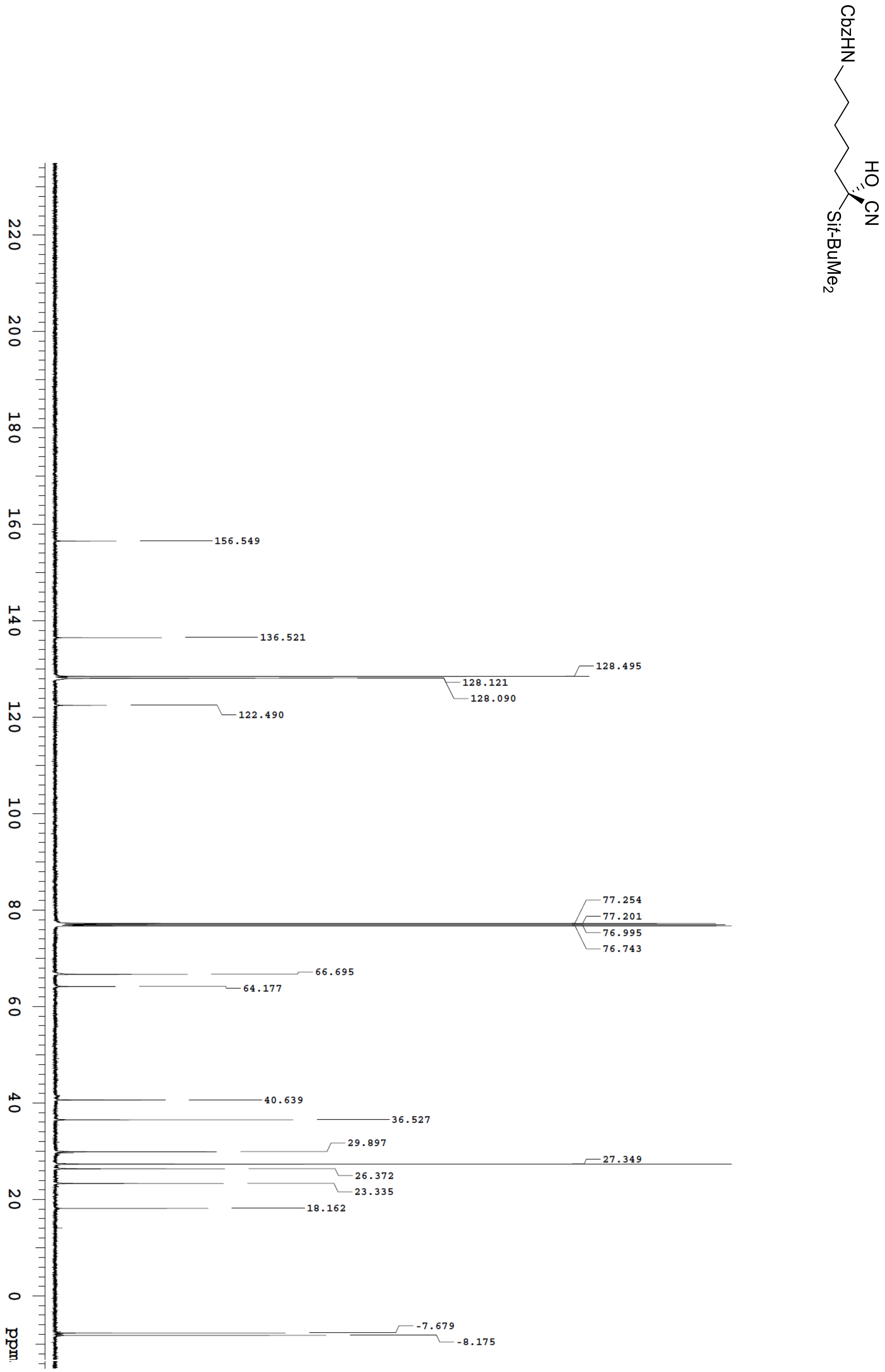

Supplementary Figure 70. ^{13}C NMR Spectrum of

Benzyl (6-(*tert*-butyldimethylsilyl)-6-cyano-6-hydroxyhexyl)carbamate (**2m**)

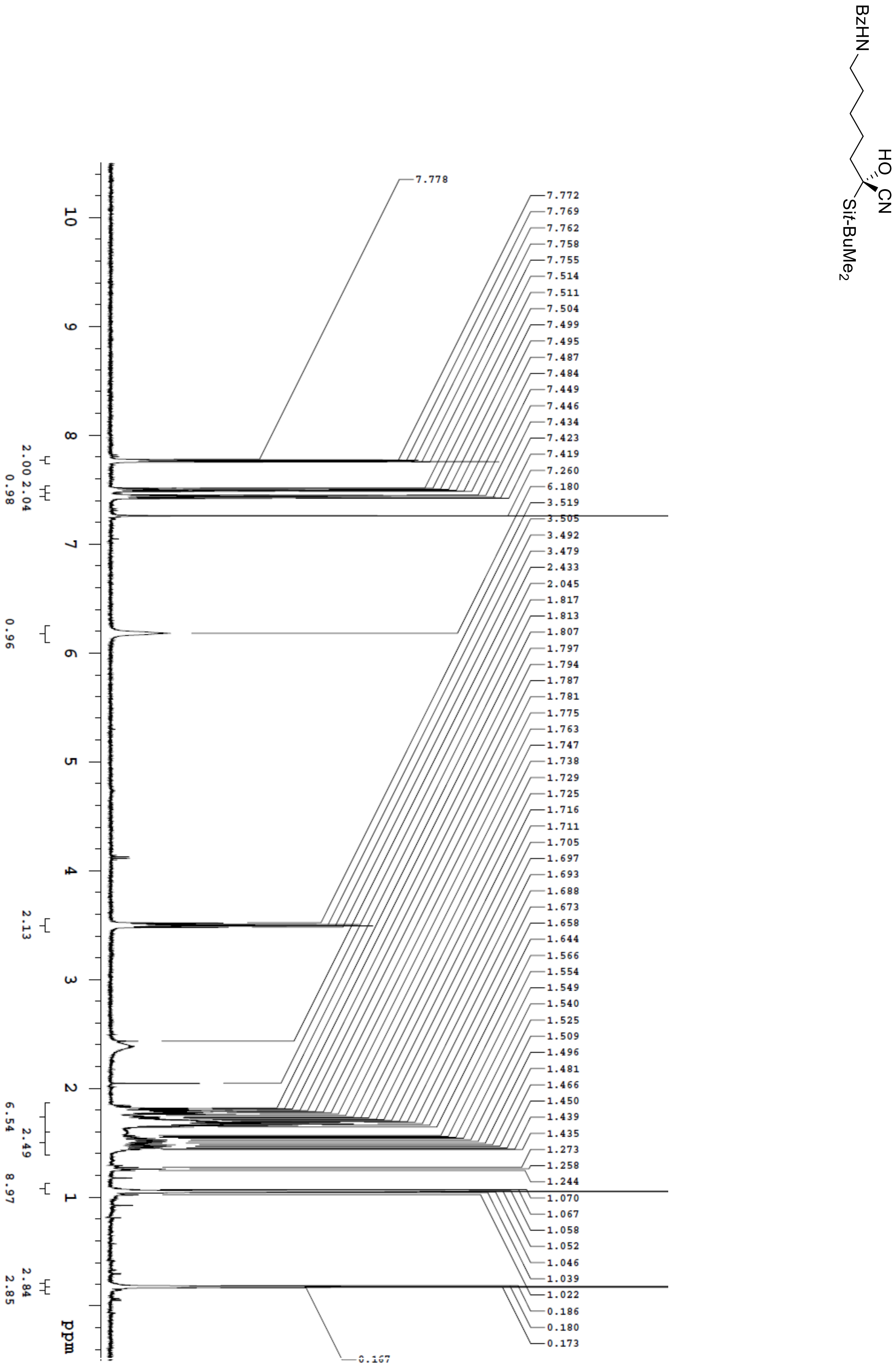

Supplementary Figure 71. ^{1}H NMR Spectrum of *N*-(6-(*tert*-Butyldimethylsilyl)-6-cyano-6-hydroxyhexyl)benzamide (**2n**)

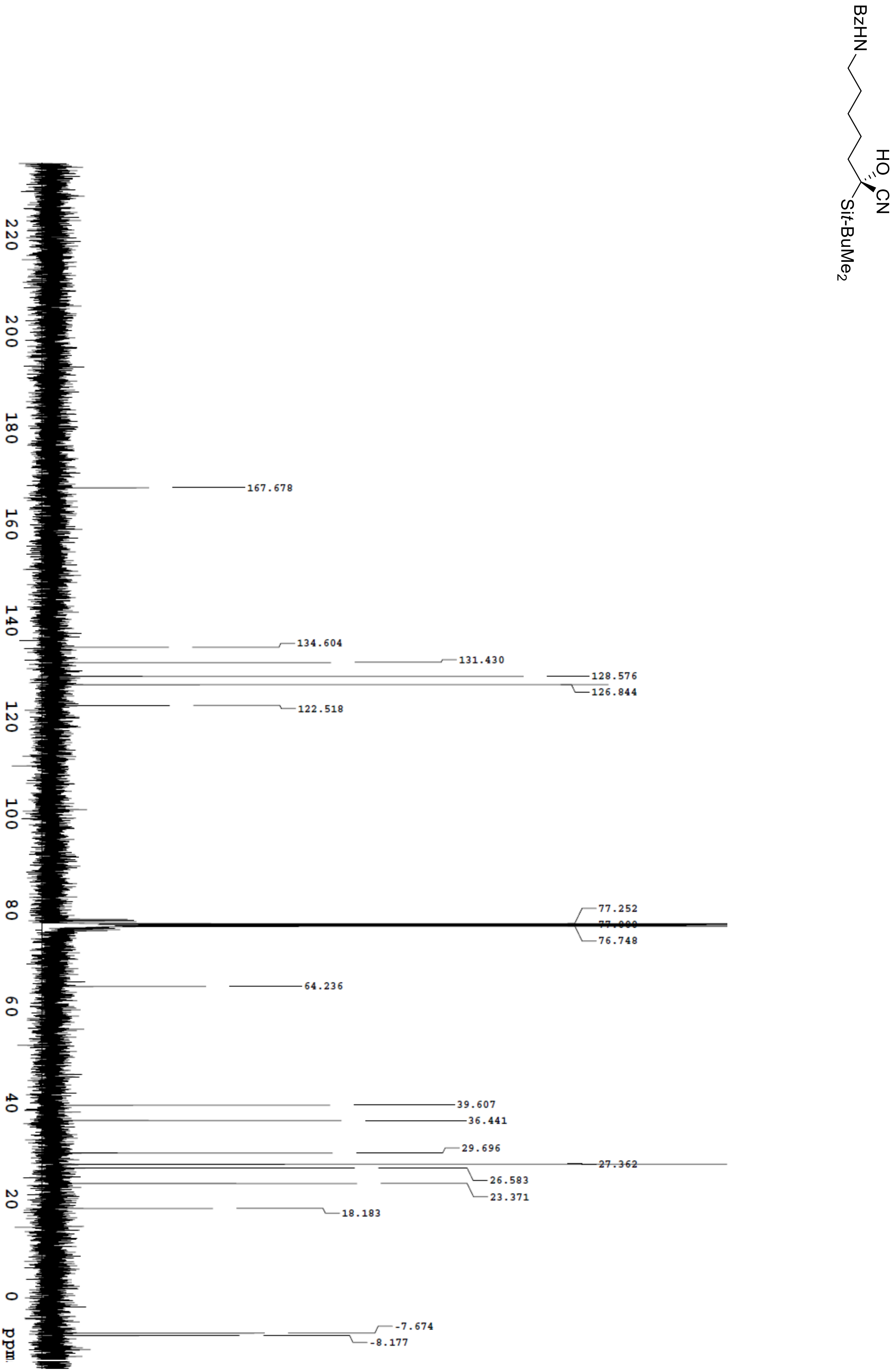

Supplementary Figure 72. ^{13}C NMR Spectrum of *N*-(6-(*tert*-Butyldimethylsilyl)-6-cyano-6-hydroxyhexyl)benzamide (**2n**)

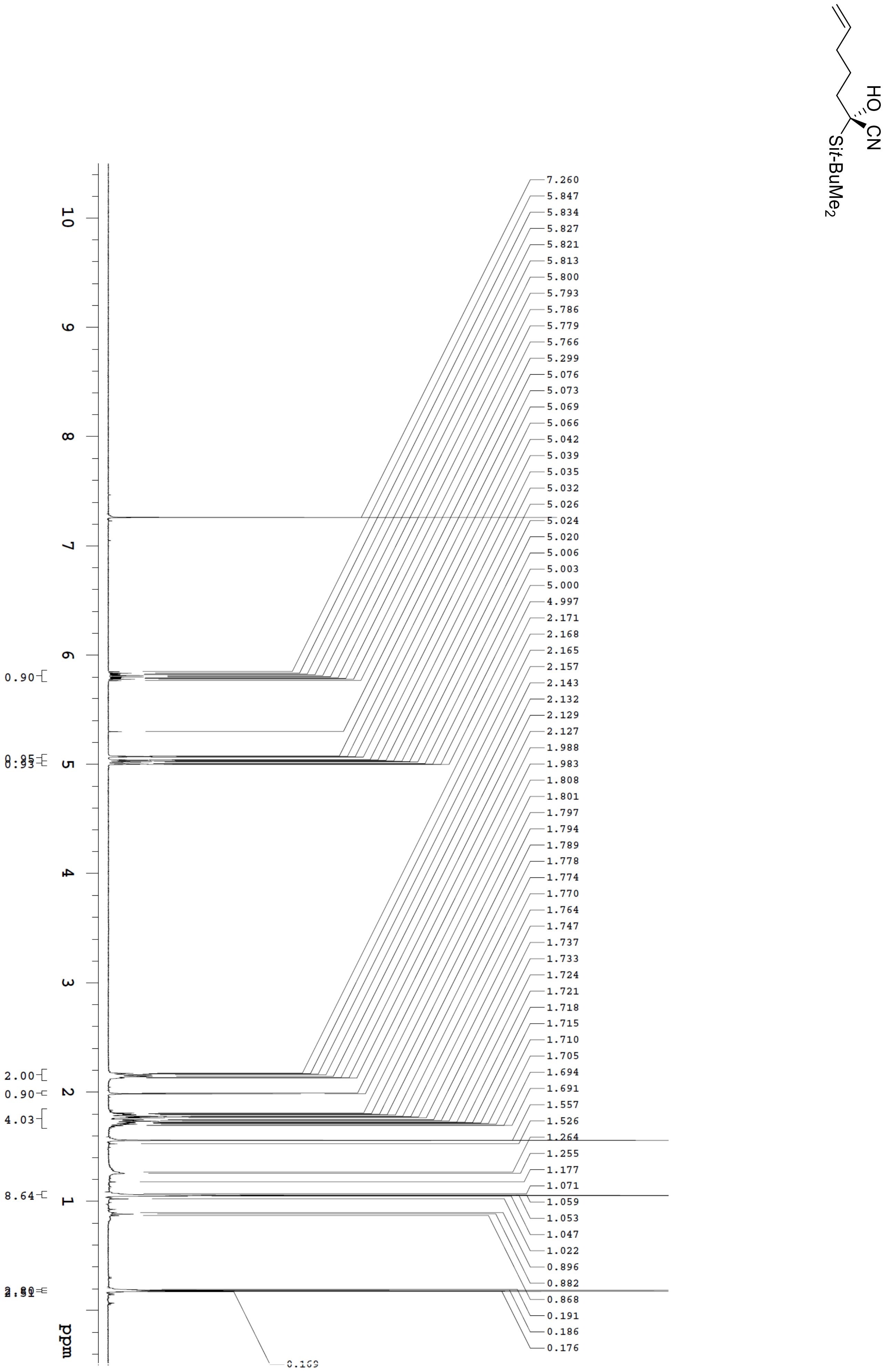

Supplementary Figure 73. ^{1}H NMR Spectrum of 2-(*tert*-Butyldimethylsilyl)-2-hydroxyhept-6-enenitrile (**2o**)

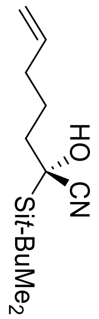

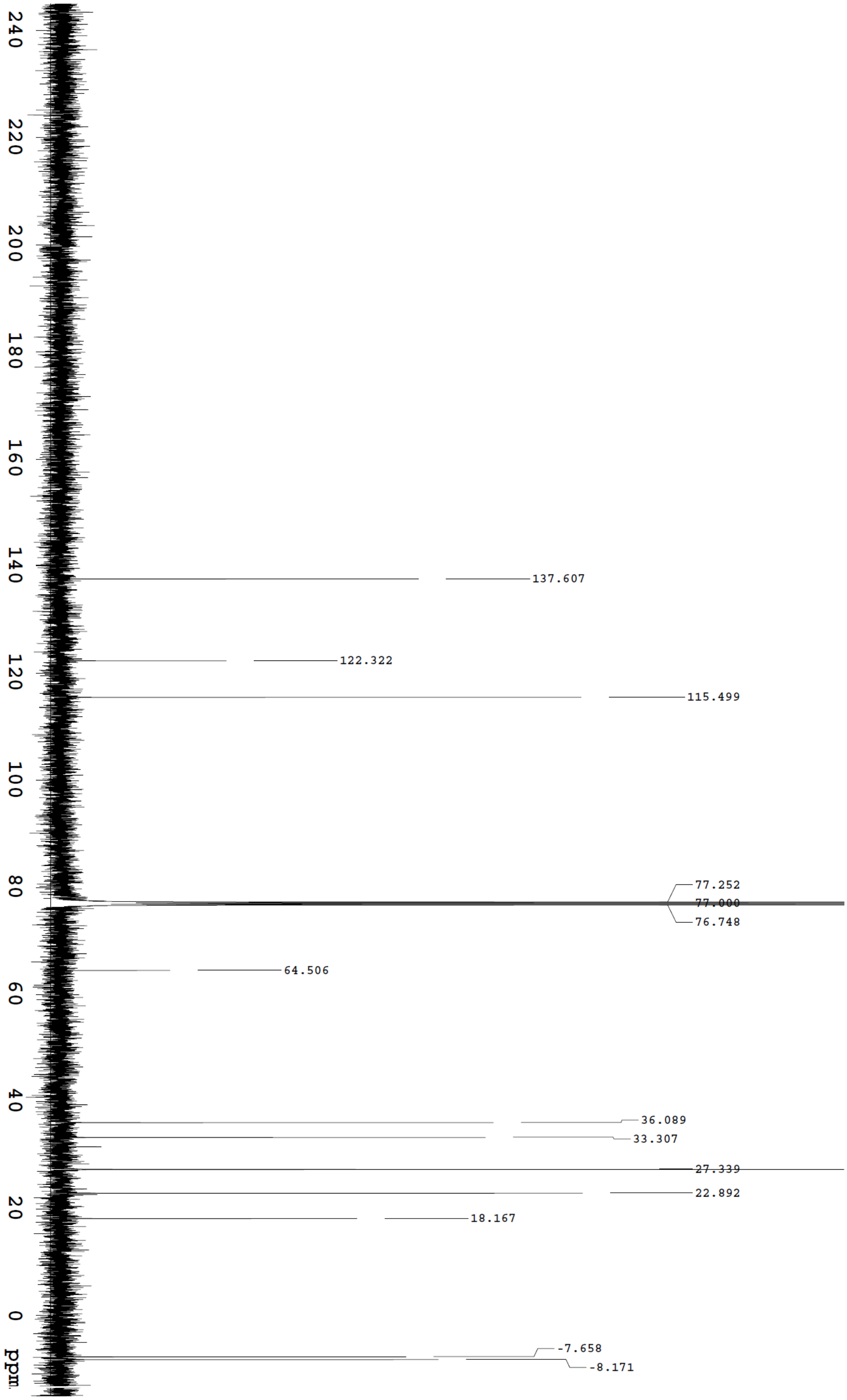

Supplementary Figure 74. ^{13}C NMR Spectrum of 2-(*tert*-Butyldimethylsilyl)-2-hydroxyhept-6-enenitrile (**2o**)

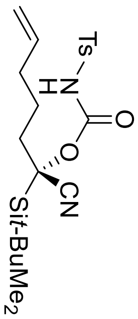

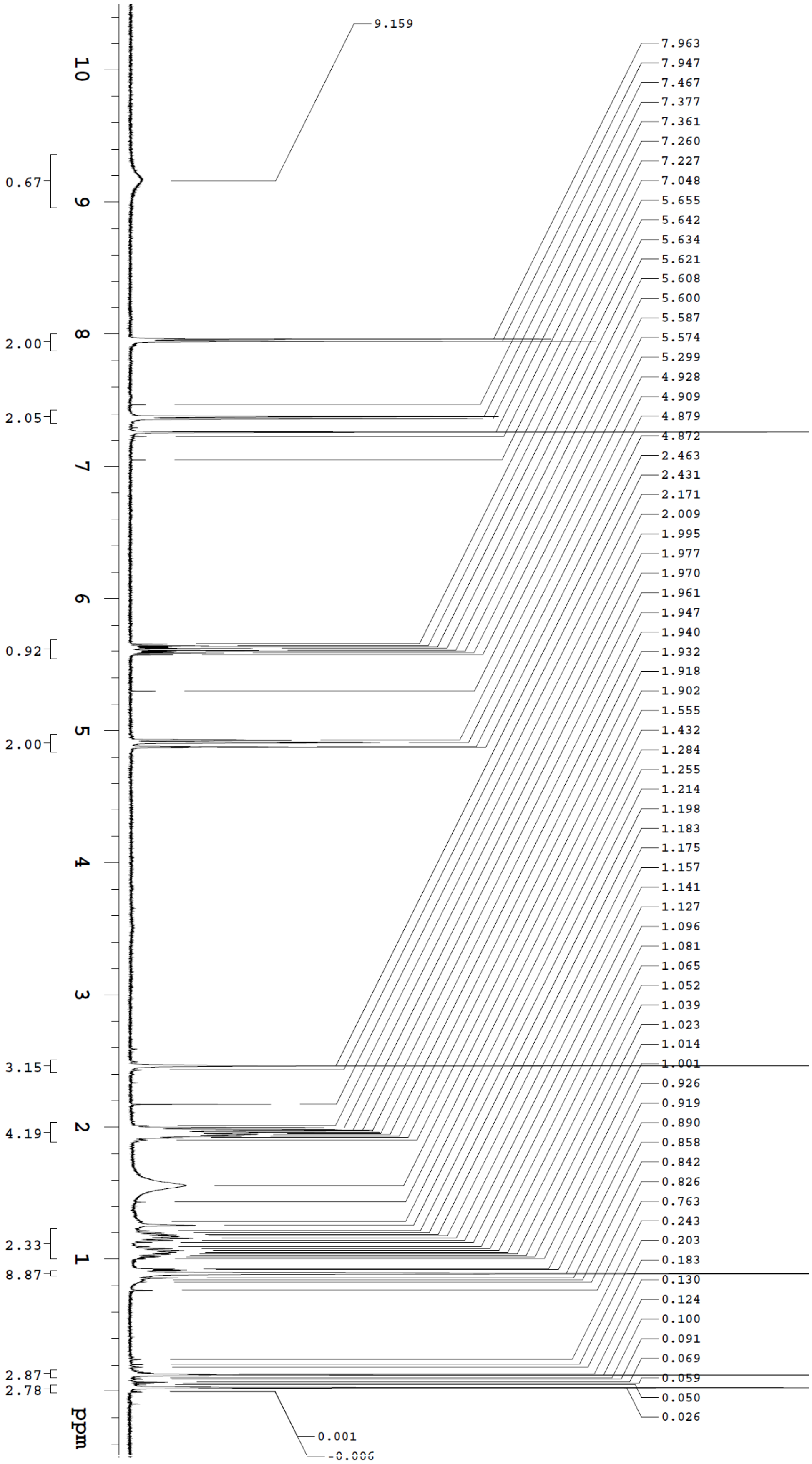

Supplementary Figure 75. ^{1}H NMR Spectrum of 1-(*tert*-Butyldimethylsilyl)-1-cyanohex-5-en-1-yl tosylcarbamate (**2o'**)

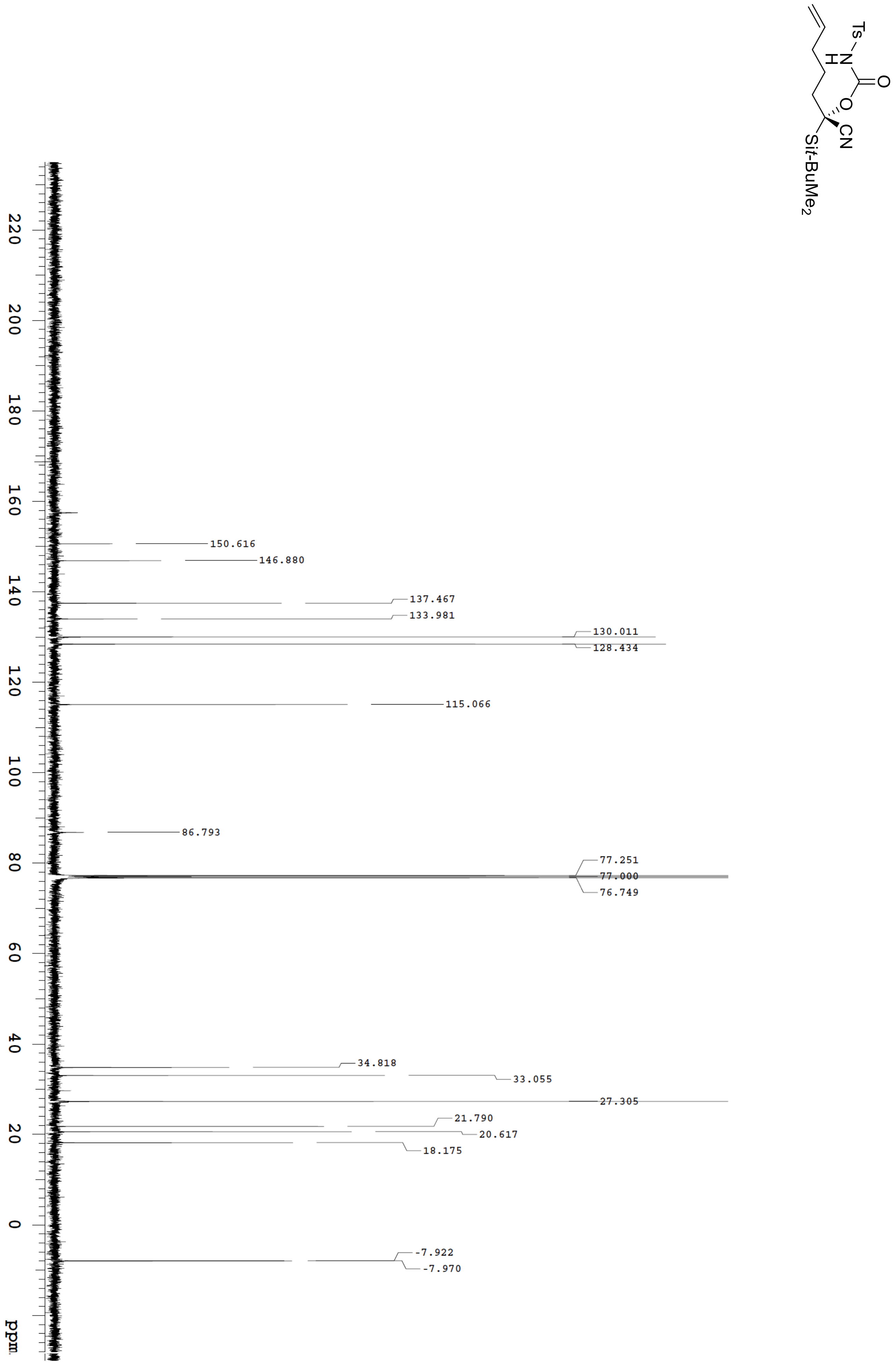

Supplementary Figure 76. ^{13}C NMR Spectrum of 1-(*tert*-Butyldimethylsilyl)-1-cyanohex-5-en-1-yl tosylcarbamate (**2o'**)

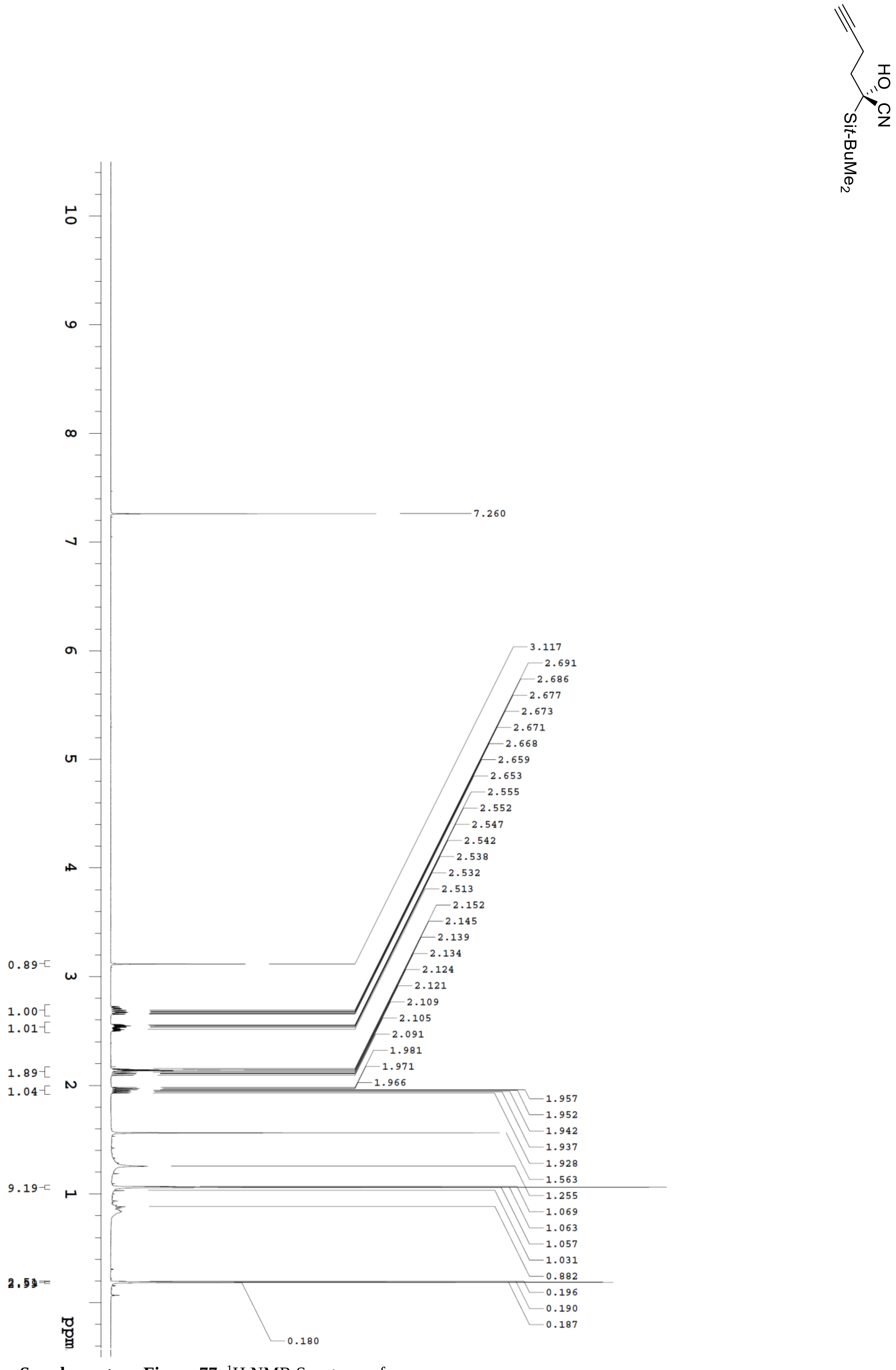

Supplementary Figure 77. ^{1}H NMR Spectrum of 2-(*tert*-Butyldimethylsilyl)-2-hydroxyhex-5-ynenitrile (**2p**)

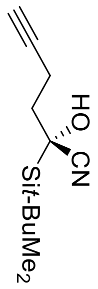

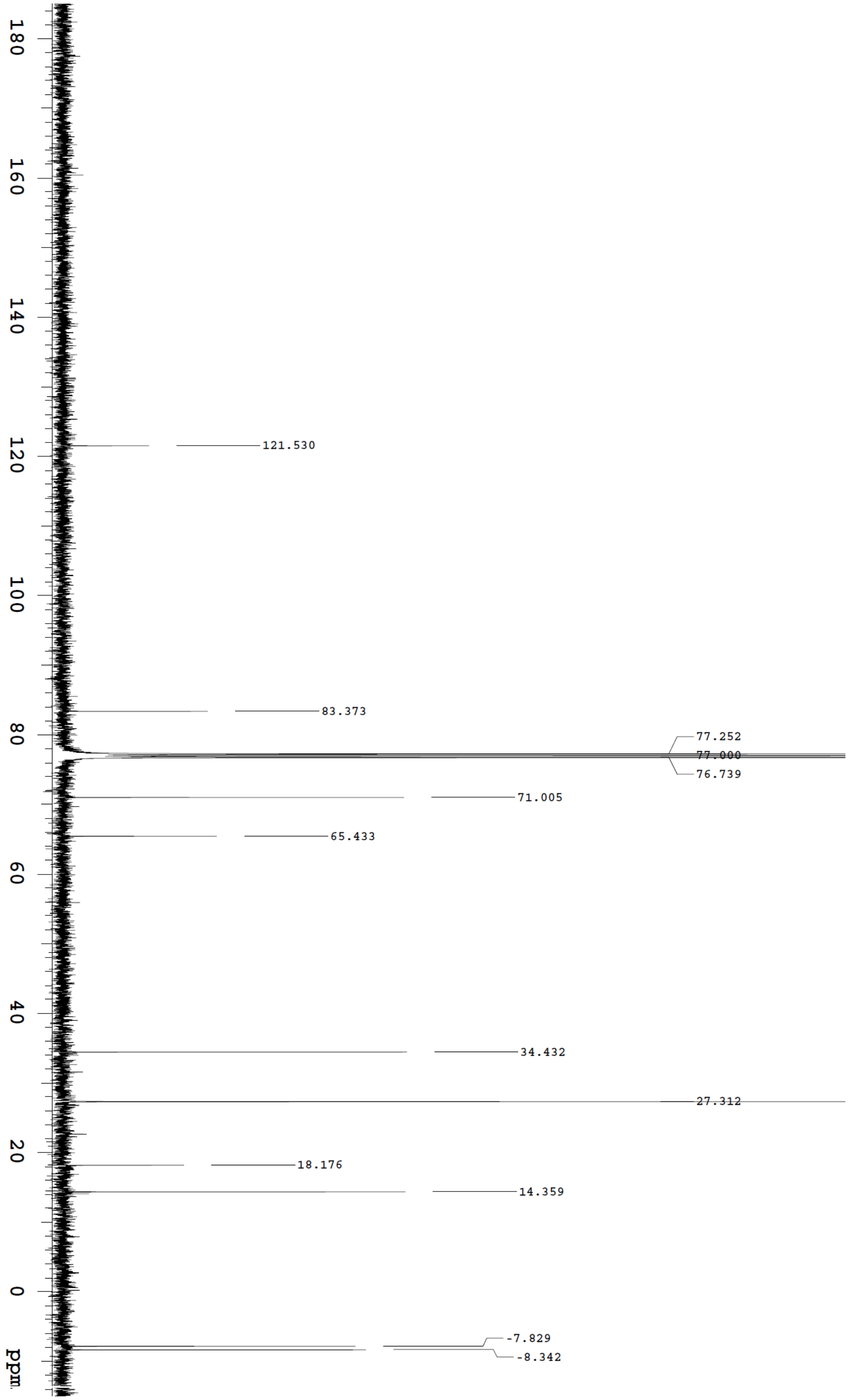

Supplementary Figure 78. ^{13}C NMR Spectrum of 2-(*tert*-Butyldimethylsilyl)-2-hydroxyhex-5-ynenitrile (**2p**)

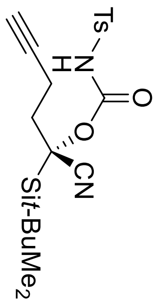

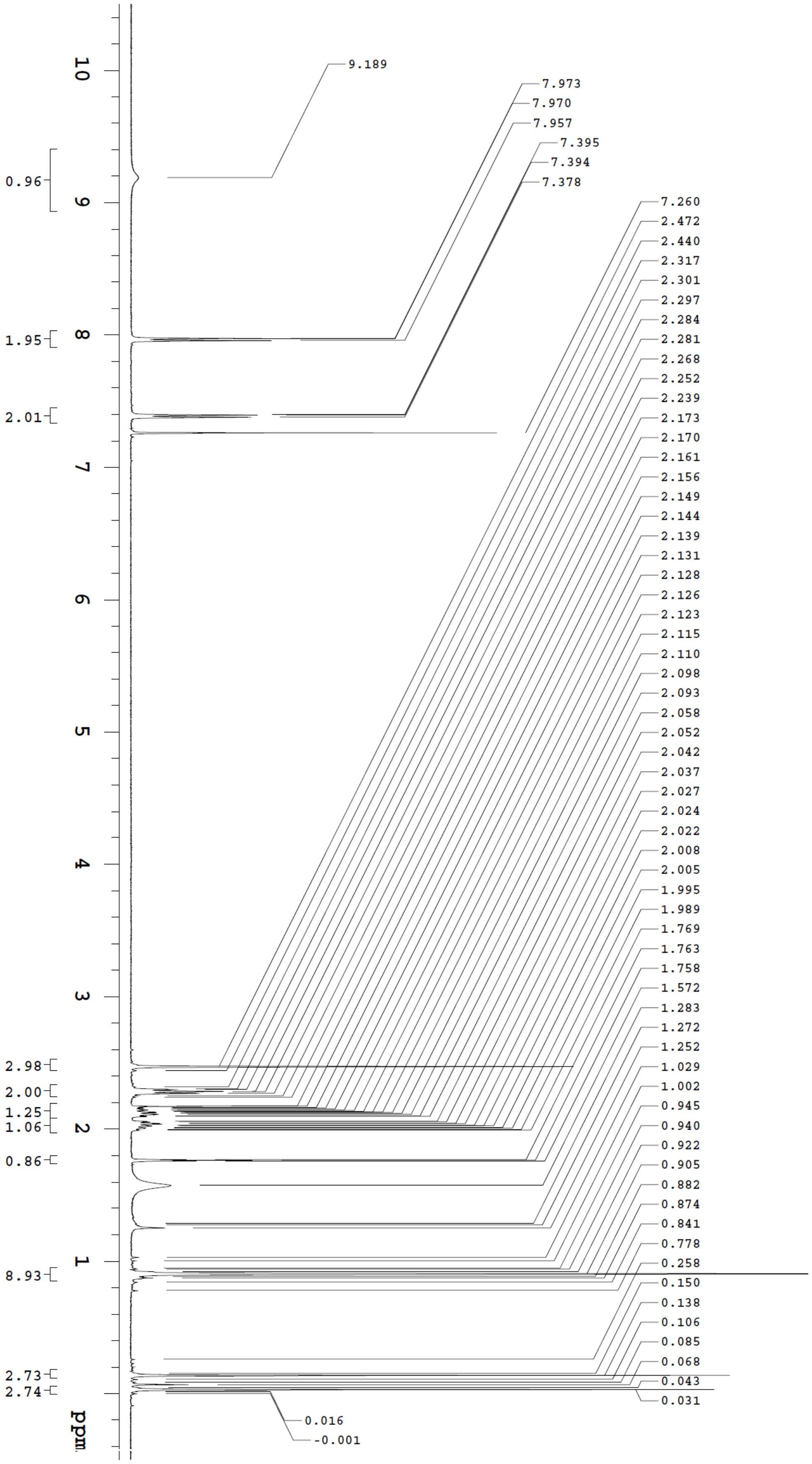

Supplementary Figure 79. ^{1}H NMR Spectrum of 1-(*tert*-Butyldimethylsilyl)-1-cyanopent-4-yn-1-yl tosylcarbamate (**2p'**)

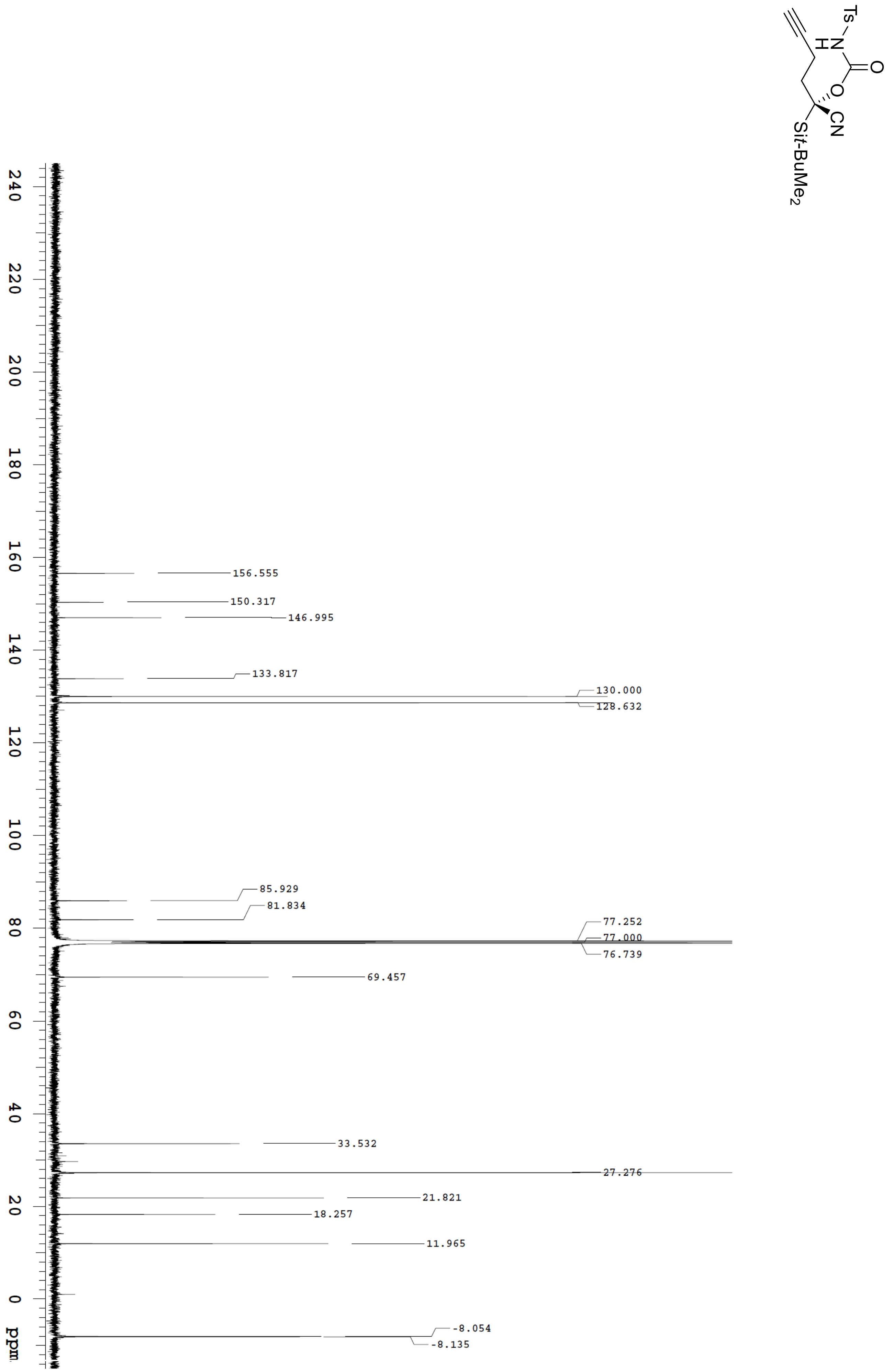

Supplementary Figure 80. ^{13}C NMR Spectrum of 1-(*tert*-Butyldimethylsilyl)-1-cyanopent-4-yn-1-yl tosylcarbamate (**2p'**)

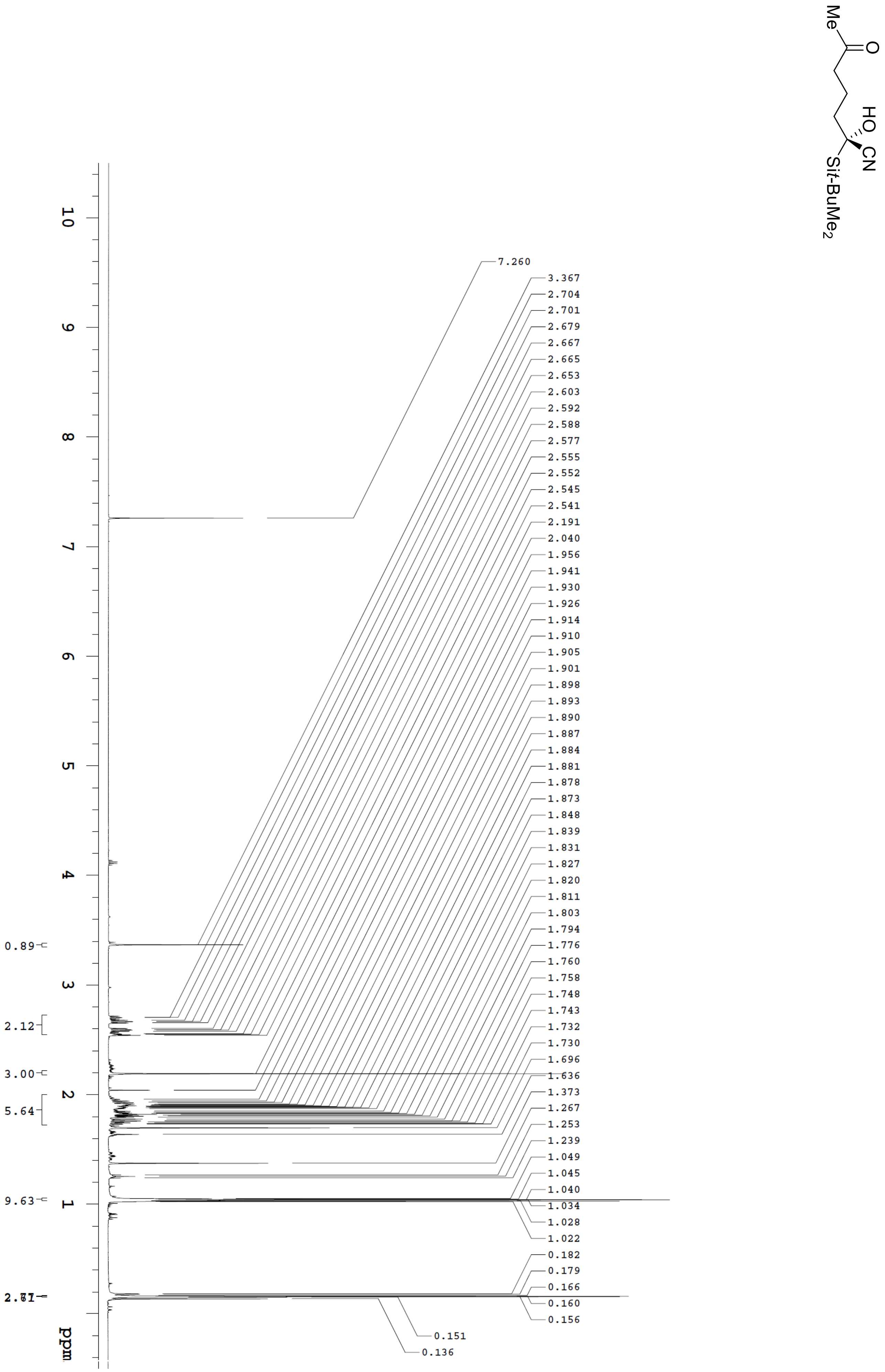

Supplementary Figure 81. ^{1}H NMR Spectrum of

2-(*tert*-Butyldimethylsilyl)-2-hydroxy-6-oxoheptanenitrile (**2q**)

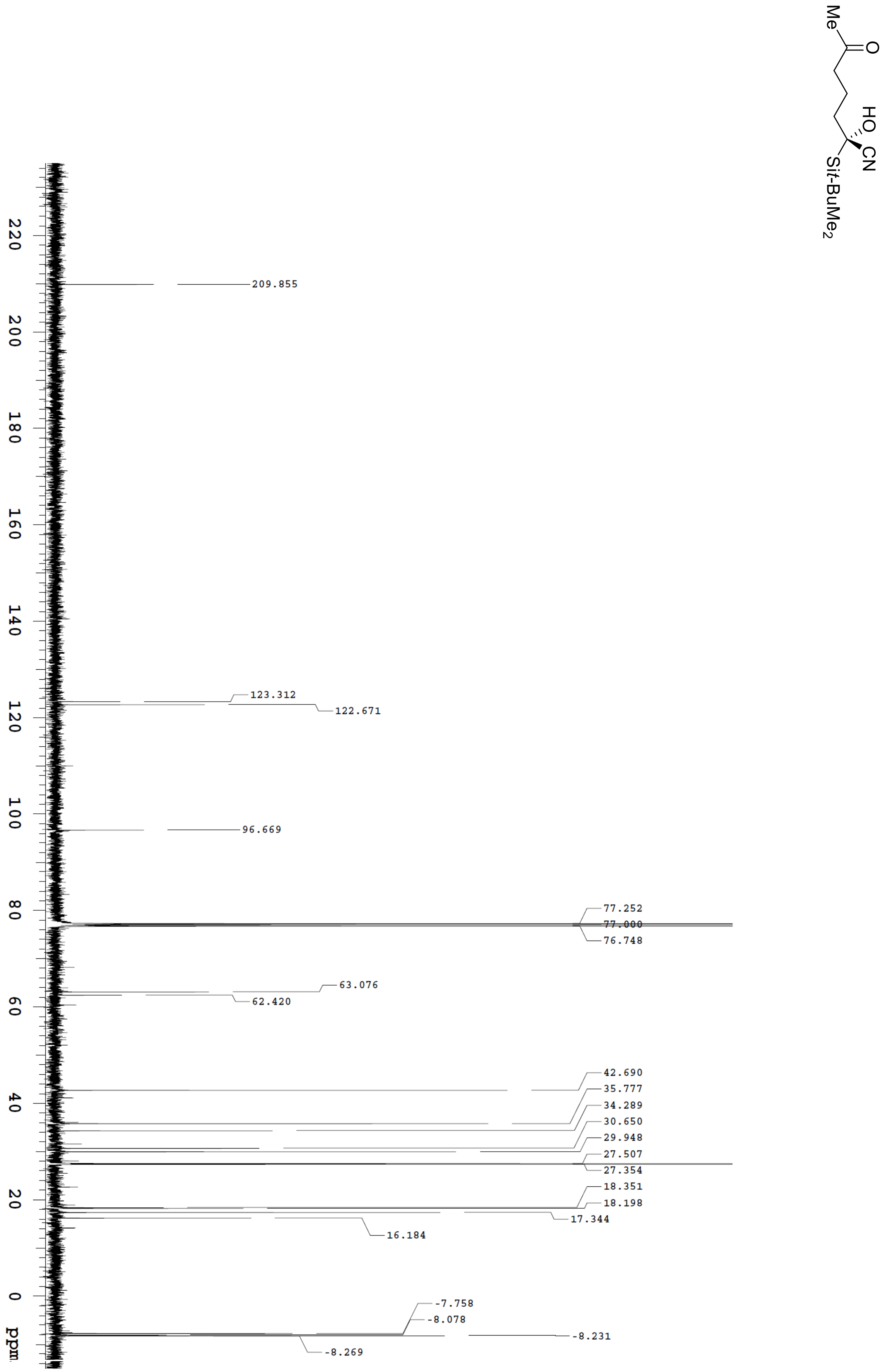

Supplementary Figure 82. ^{13}C NMR Spectrum of 2-(*tert*-Butyldimethylsilyl)-2-hydroxy-6-oxoheptanenitrile (**2q**)

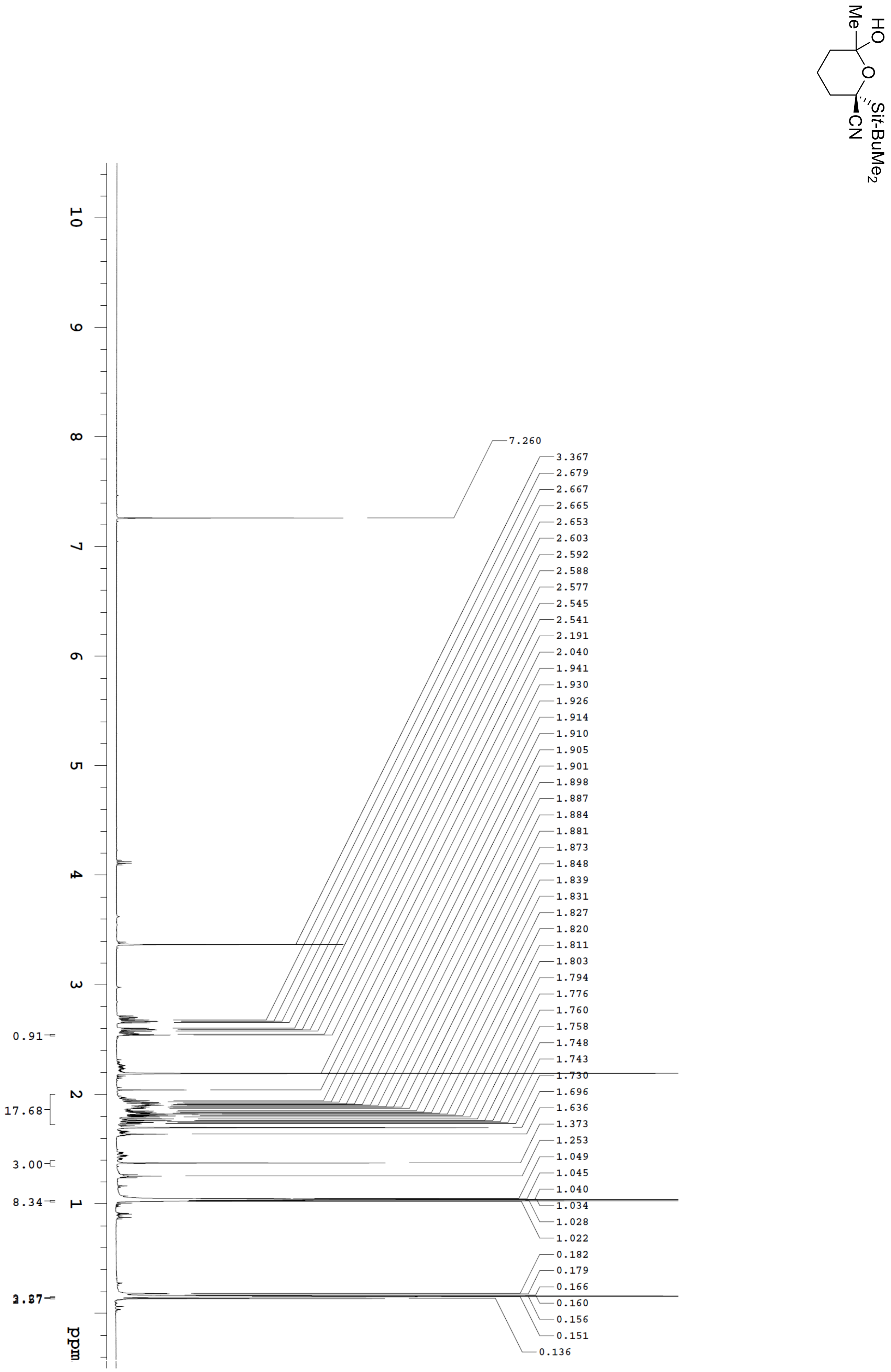

Supplementary Figure 83. ^{1}H NMR Spectrum of 2-(*tert*-Butyldimethylsilyl)-6-hydroxy-6-methyltetrahydro-2*H*-pyran-2-carbonitrile (**34**)

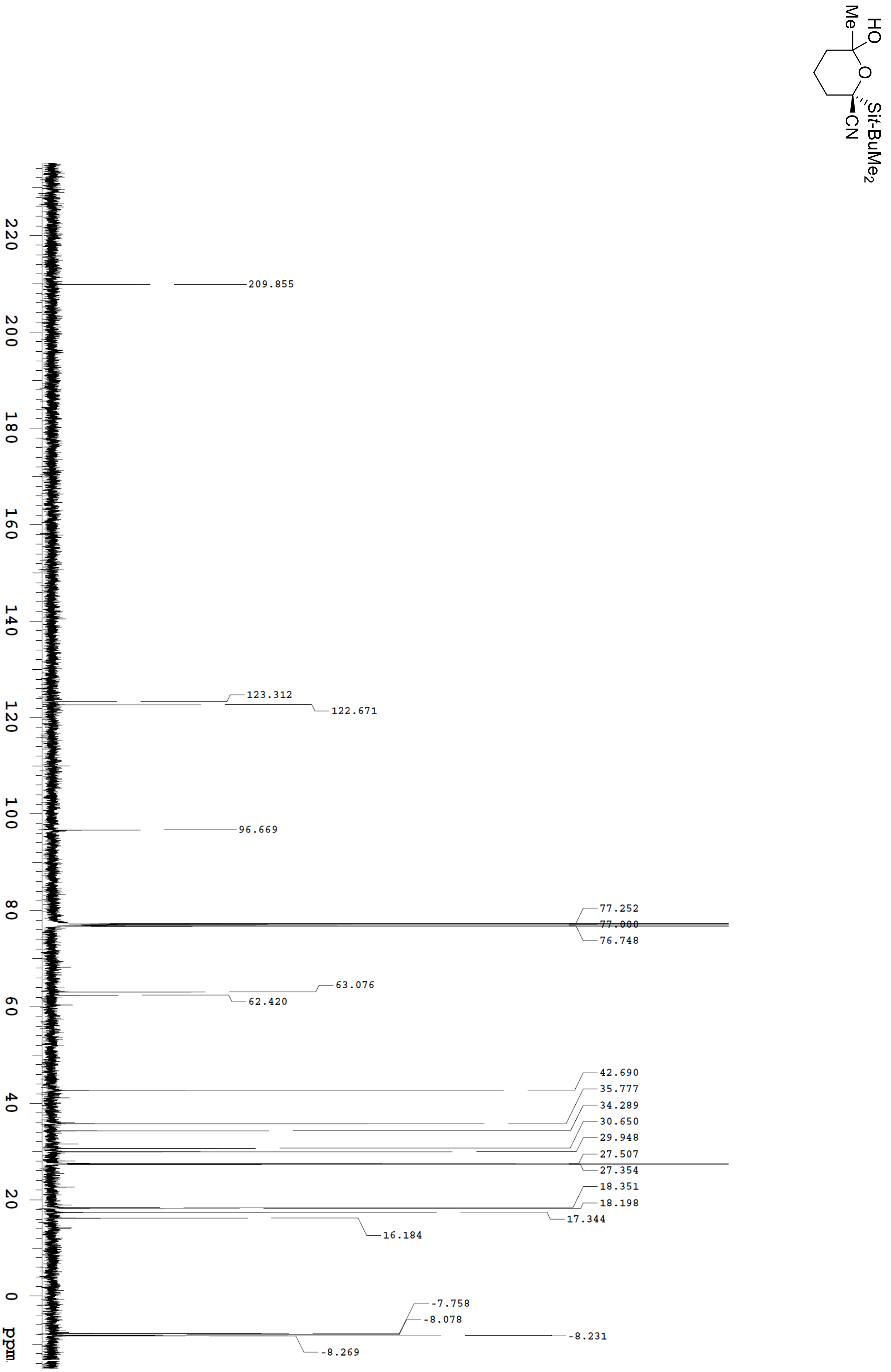

Supplementary Figure 84. ^{13}C NMR Spectrum of 2-(*tert*-Butyldimethylsilyl)-6-hydroxy-6-methyltetrahydro-2*H*-pyran-2-carbonitrile (**34**)

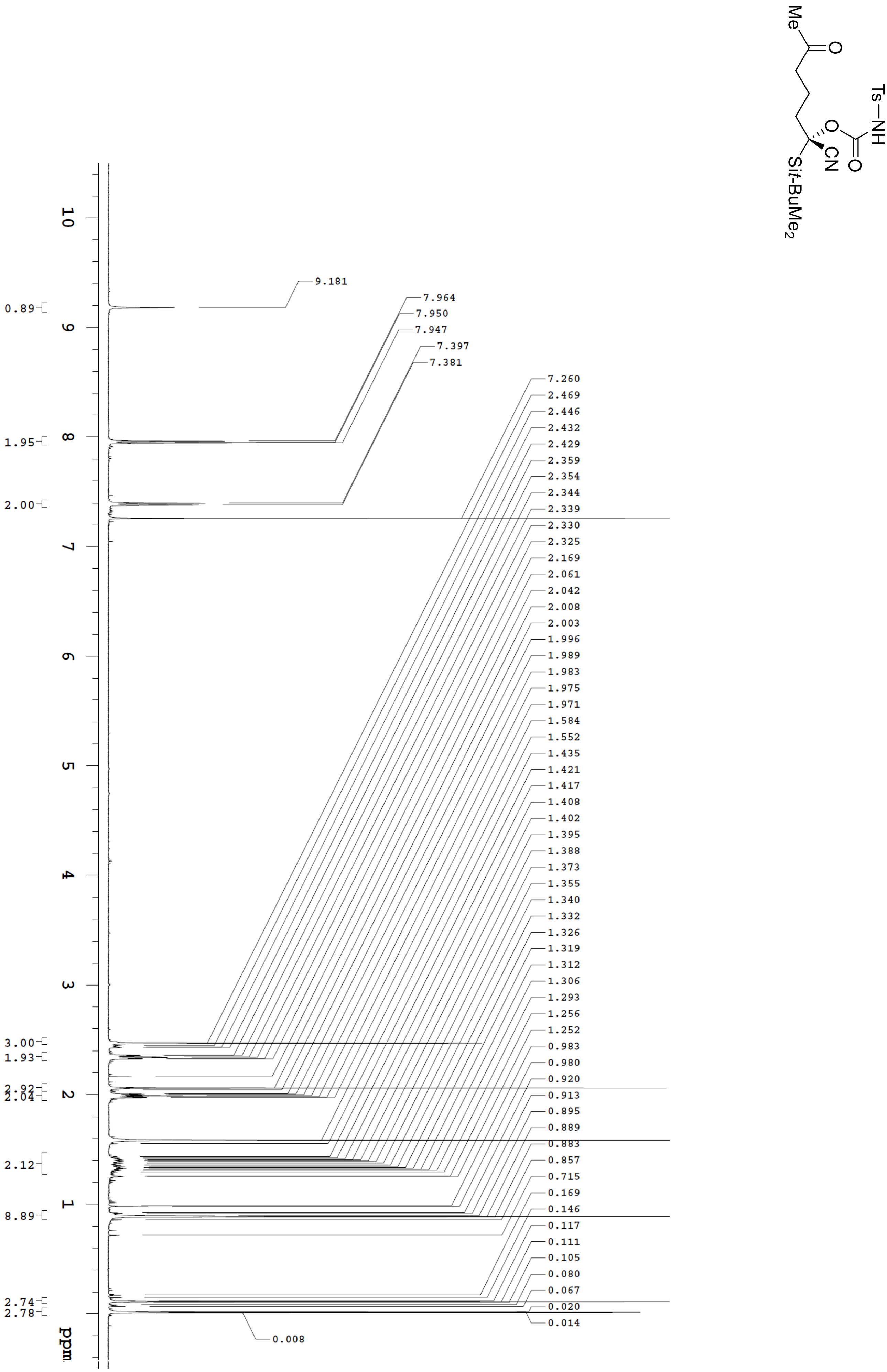

Supplementary Figure 85. ^{1}H NMR Spectrum of

1-(*tert*-Butyldimethylsilyl)-1-cyano-5-oxohexyl tosylcarbamate (**2q'**)

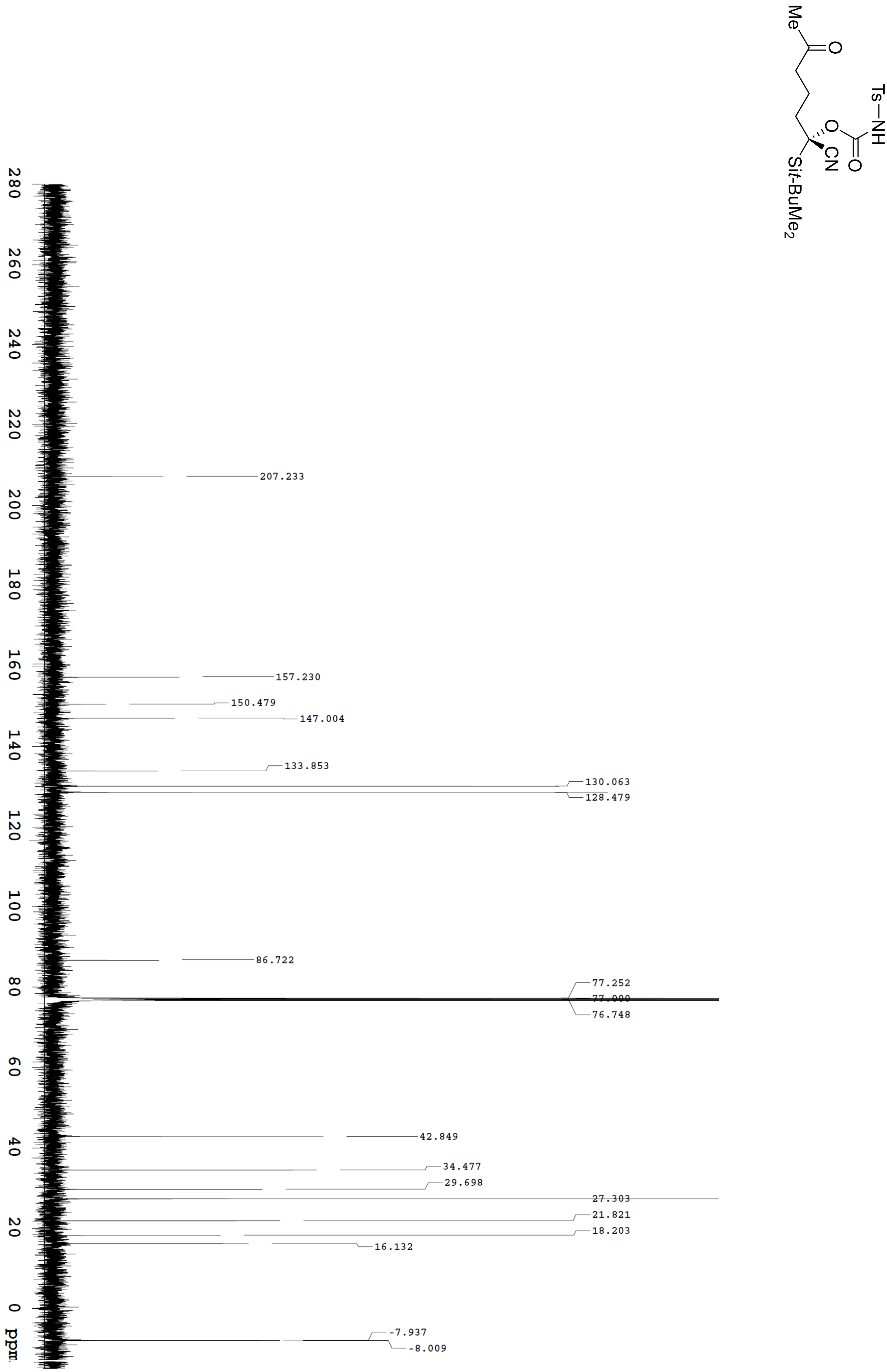

Supplementary Figure 86. ^{13}C NMR Spectrum of 1-(*tert*-Butyldimethylsilyl)-1-cyano-5-oxohexyl tosylcarbamate (**2q'**)

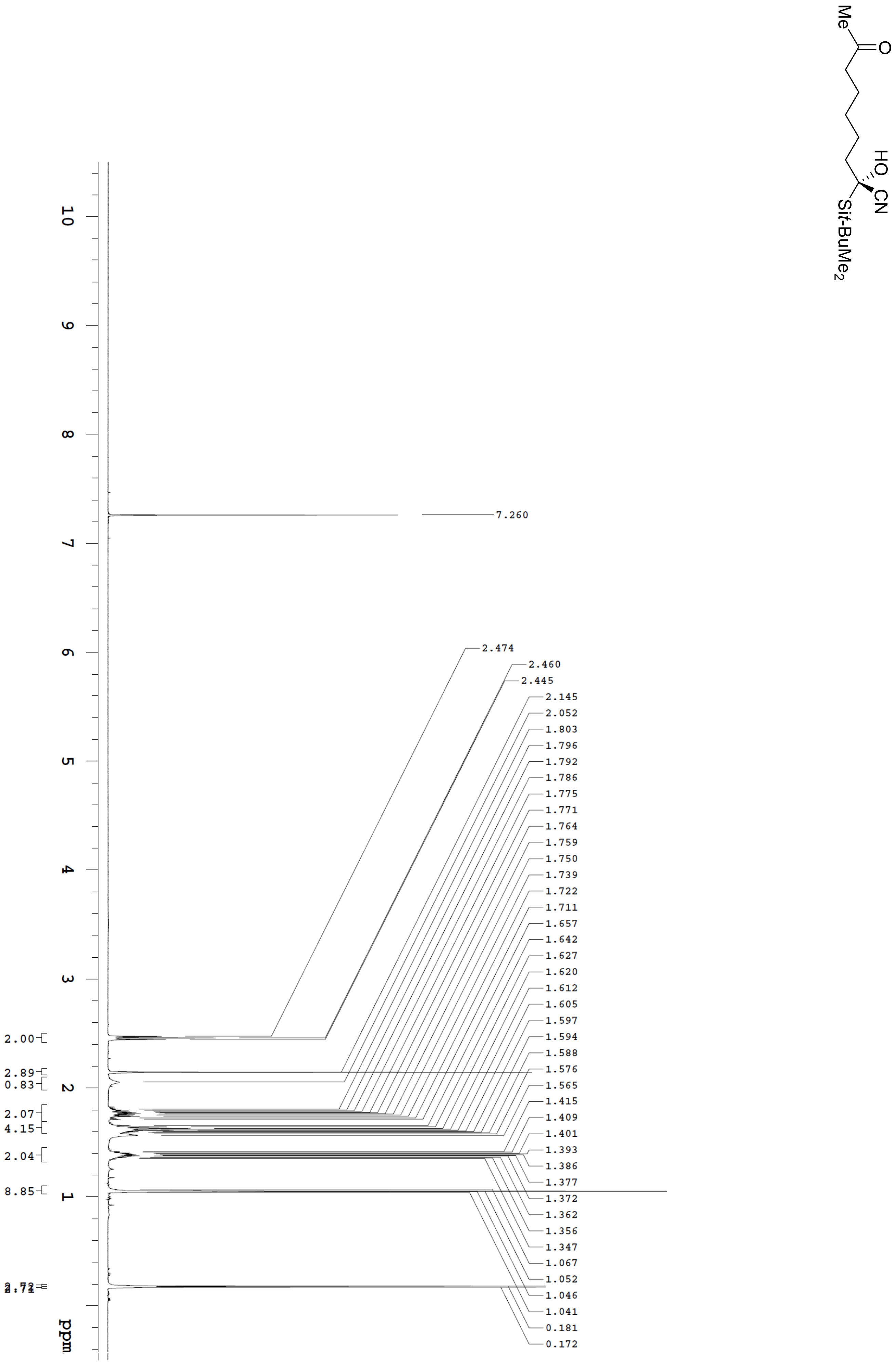

Supplementary Figure 87. ^{1}H NMR Spectrum of 2-(*tert*-Butyldimethylsilyl)-2-hydroxy-8-oxononanenitrile (**2r**)

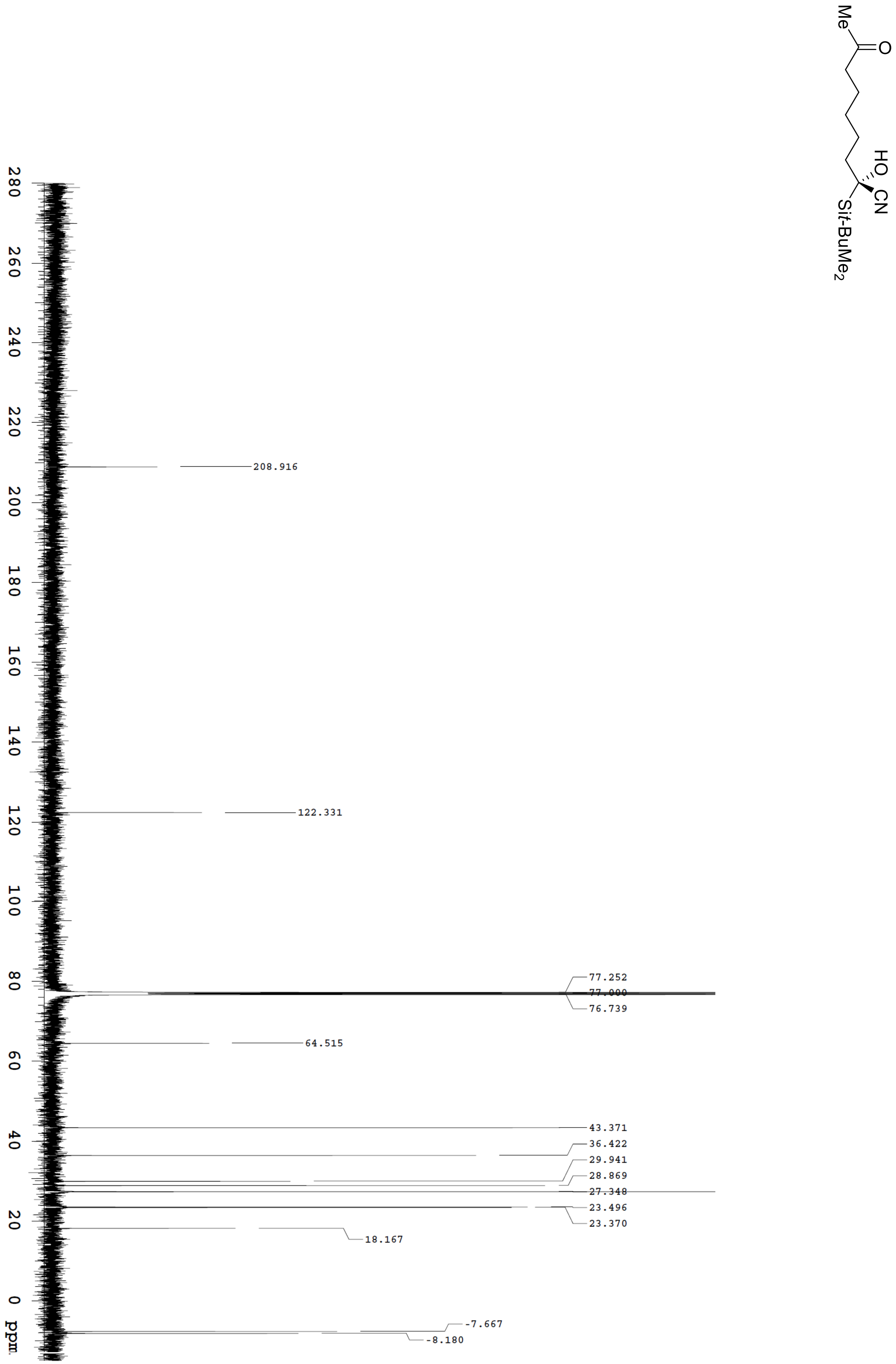

Supplementary Figure 88. ^{13}C NMR Spectrum of 2-(*tert*-Butyldimethylsilyl)-2-hydroxy-8-oxononanenitrile (**2r**)

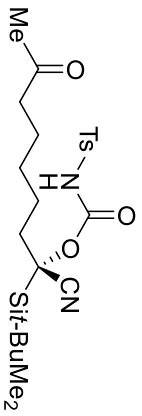

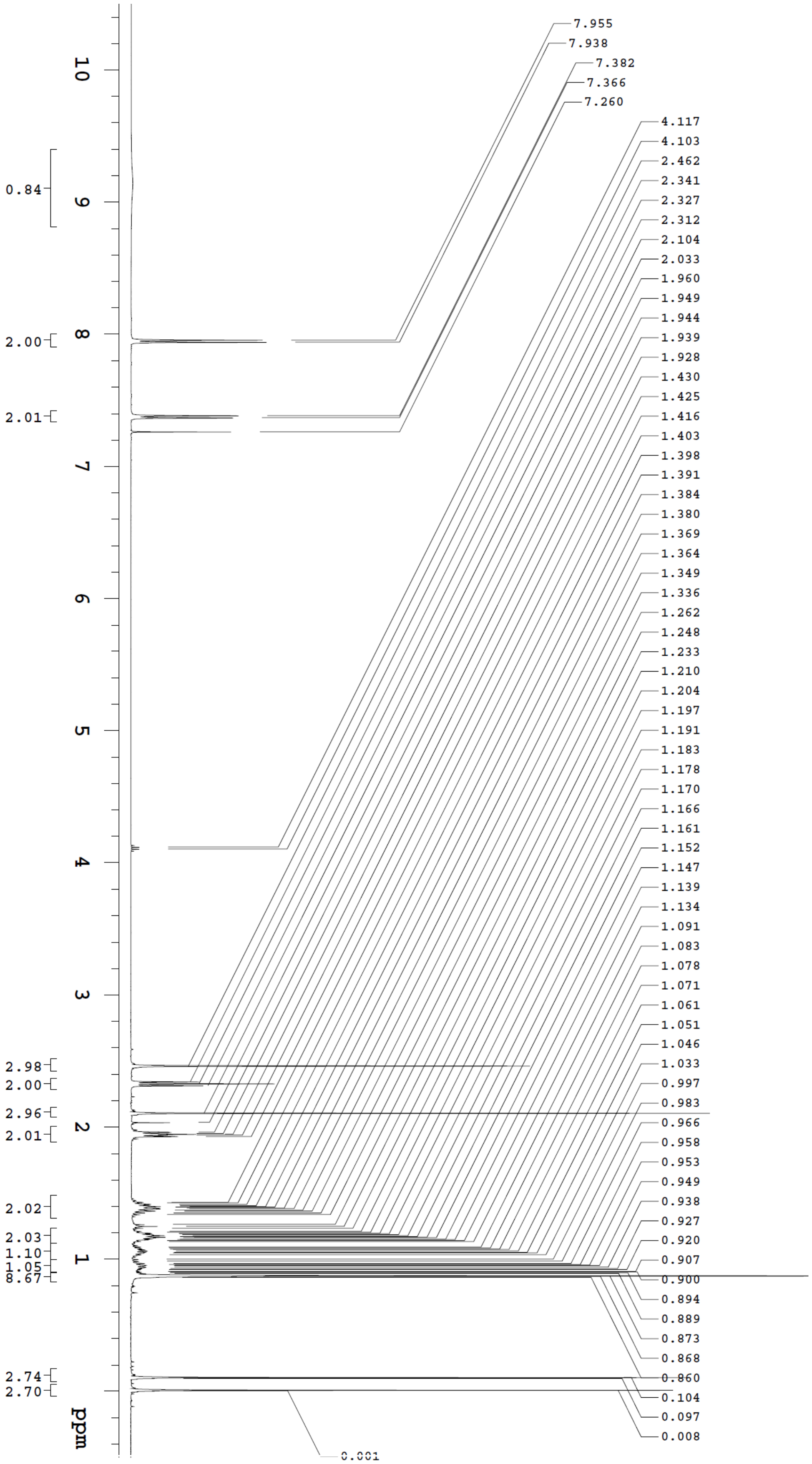

Supplementary Figure 89. ^{1}H NMR Spectrum of 1-(*tert*-Butyldimethylsilyl)-1-cyano-7-oxooctyl tosylcarbamate (**2r'**)

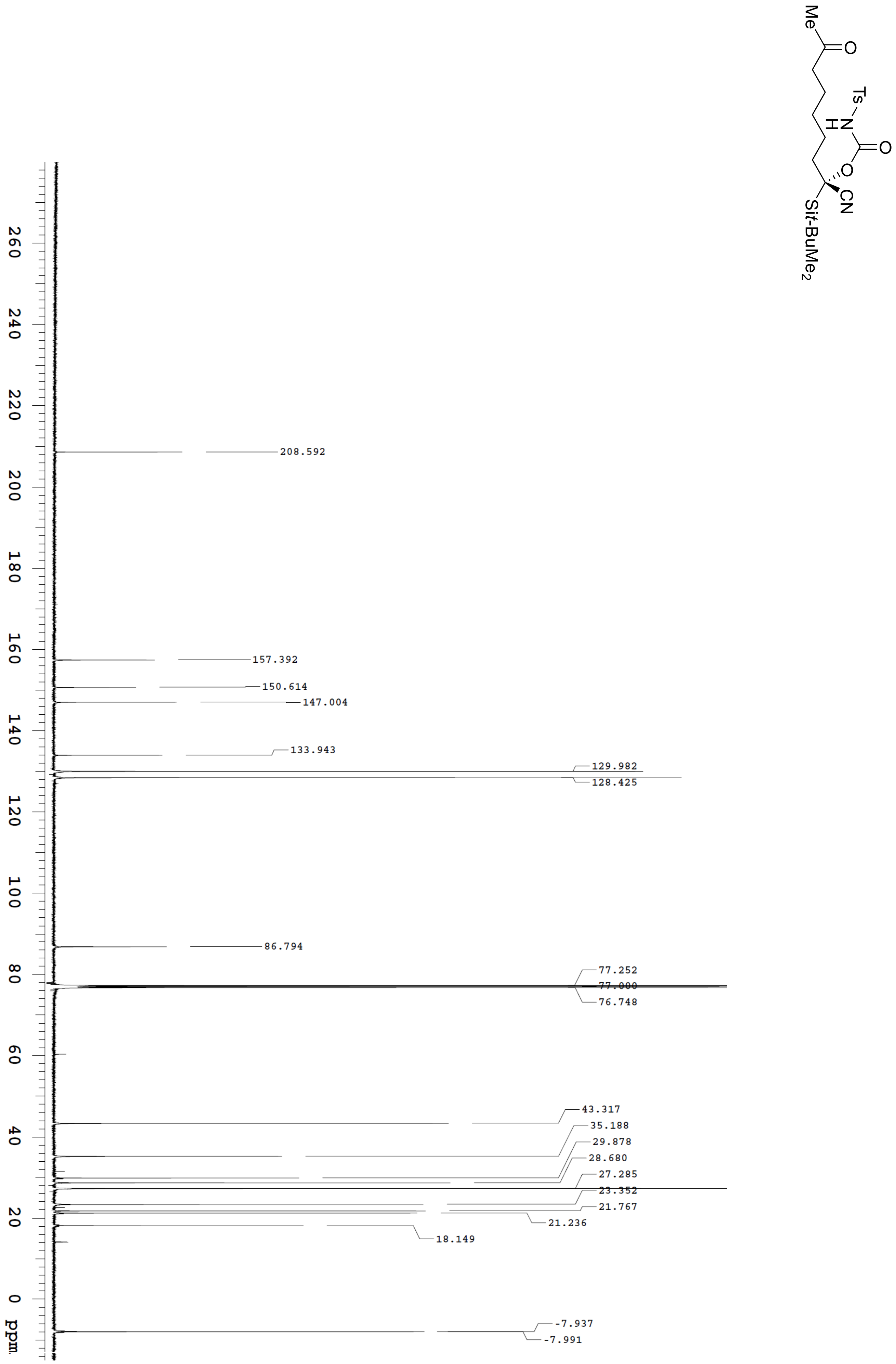

Supplementary Figure 90. ^{13}C NMR Spectrum of 1-(*tert*-Butyldimethylsilyl)-1-cyano-7-oxooctyl tosylcarbamate (**2r'**)

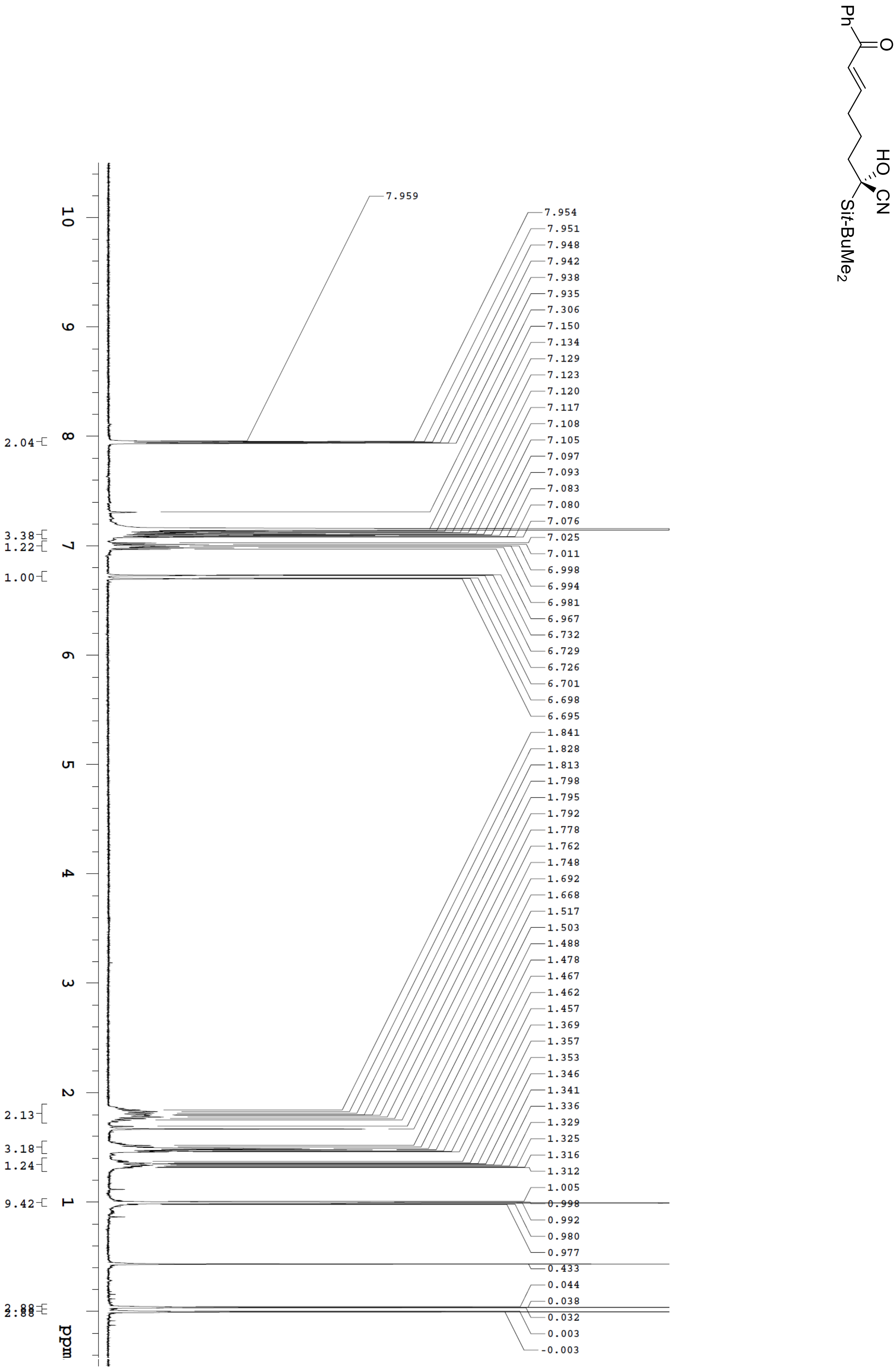

Supplementary Figure 91. ^{1}H NMR Spectrum of (*E*)-2-(*tert*-Butyldimethylsilyl)-2-hydroxy-8-oxo-8-phenyloct-6-enenitrile (**2s**)

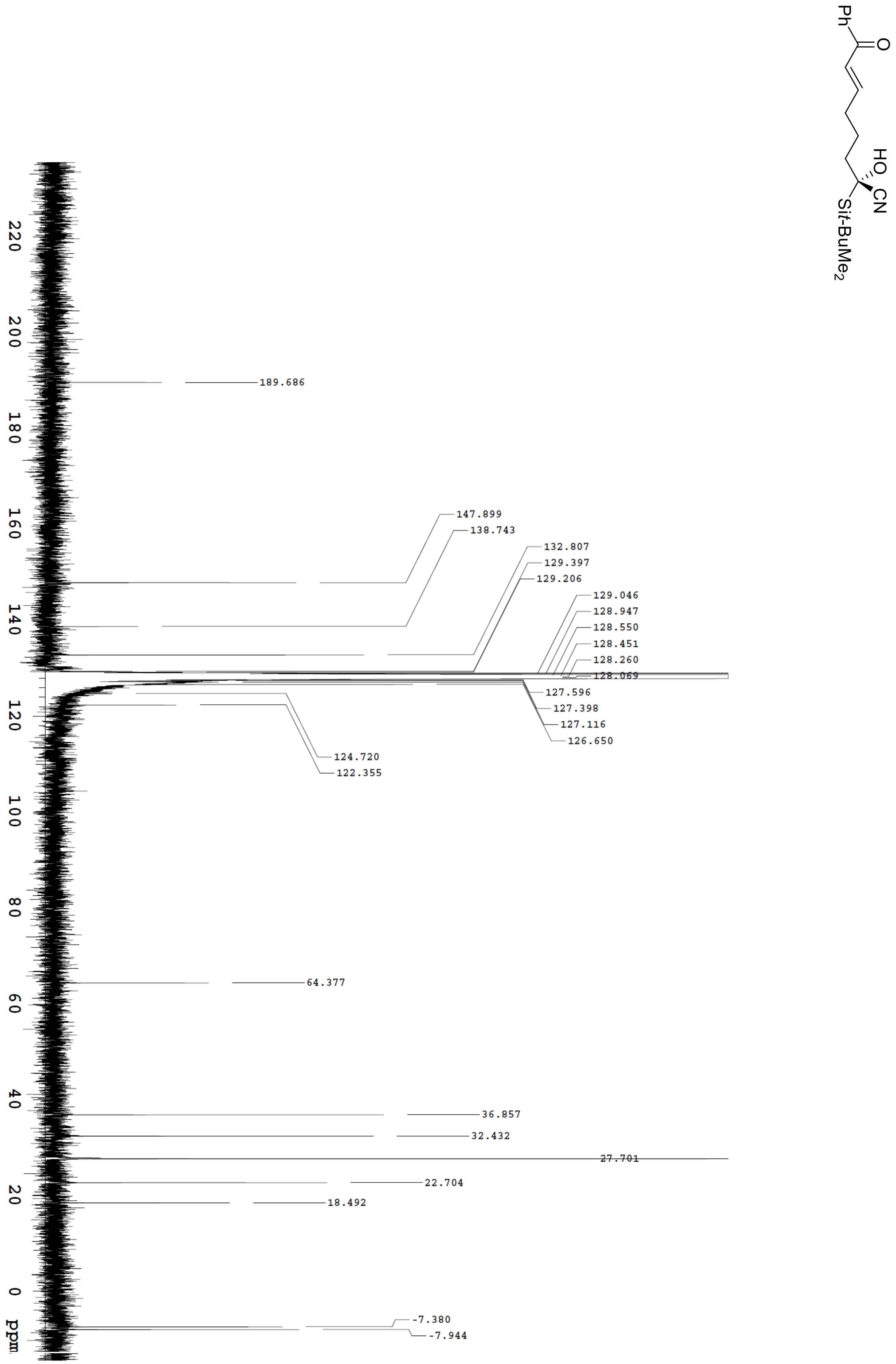

Supplementary Figure 92. ^{13}C NMR Spectrum of (*E*)-2-(*tert*-Butyldimethylsilyl)-2-hydroxy-8-oxo-8-phenyloct-6-enenitrile (**2s**)

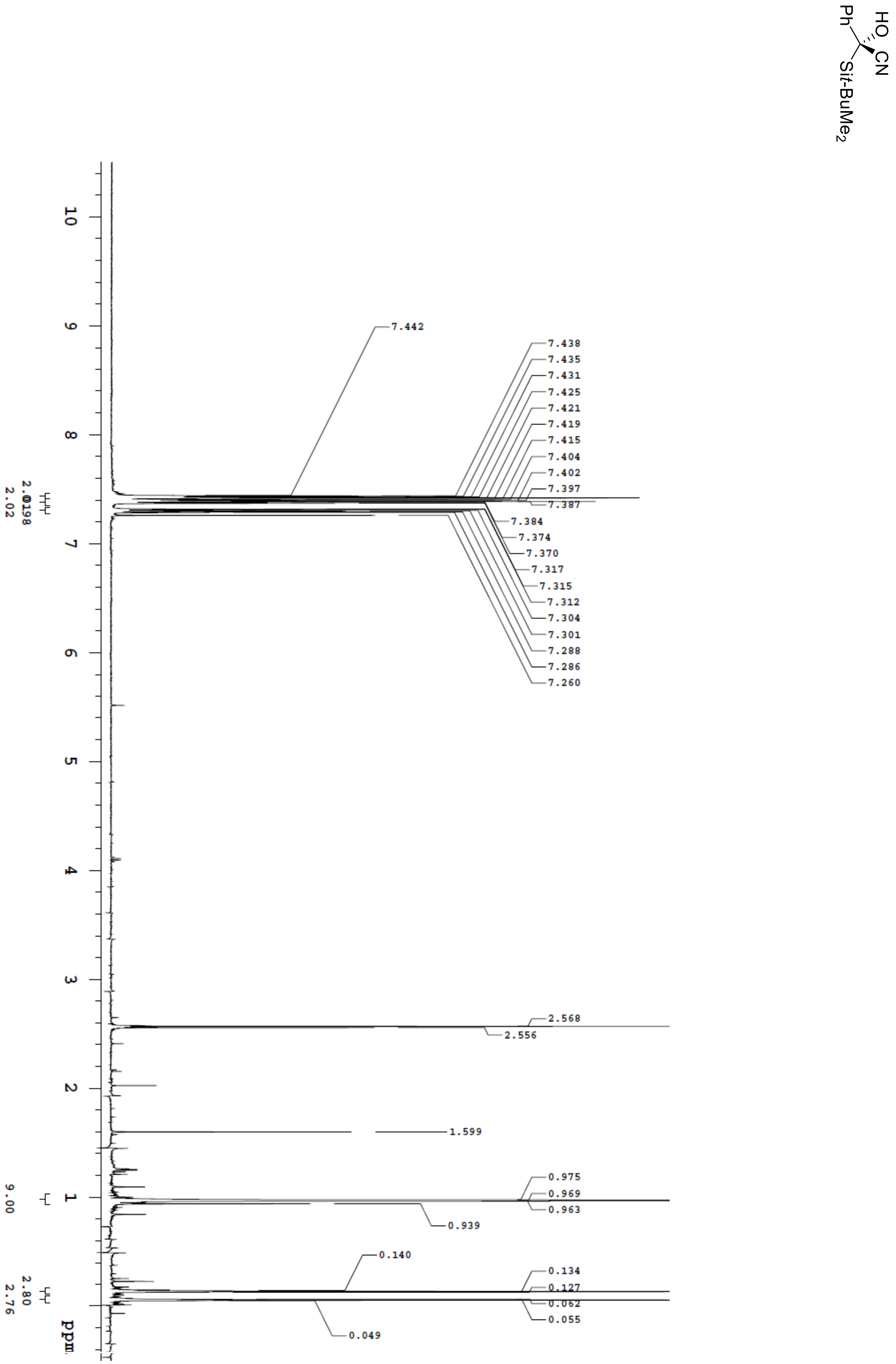

Supplementary Figure 93. ^{1}H NMR Spectrum of 2-(*tert*-Butyldimethylsilyl)-2-hydroxy-2-phenylacetonitrile (**2t**)

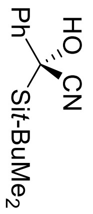

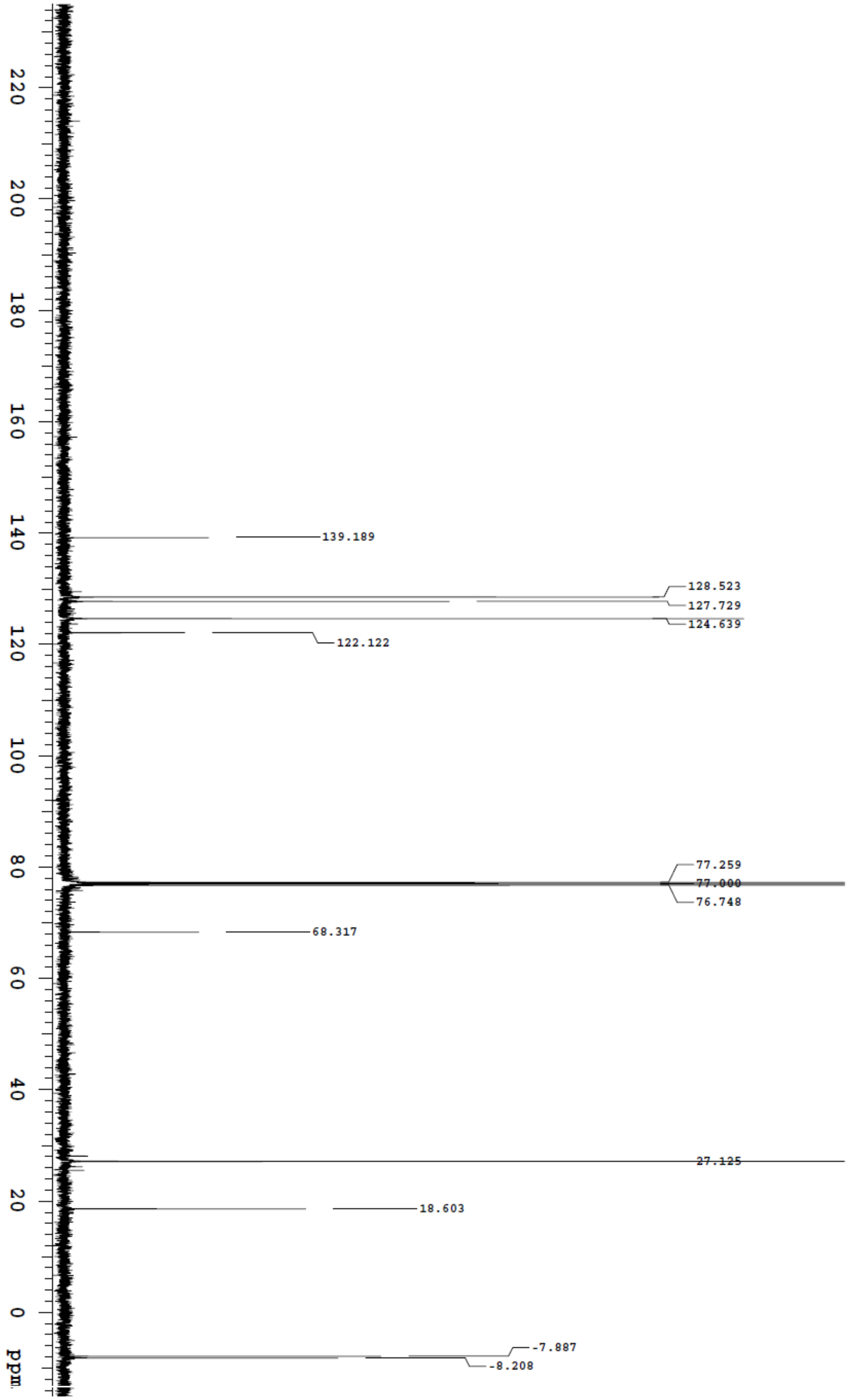

Supplementary Figure 94. ^{13}C NMR Spectrum of 2-(*tert*-Butyldimethylsilyl)-2-hydroxy-2-phenylacetonitrile (**2t**)

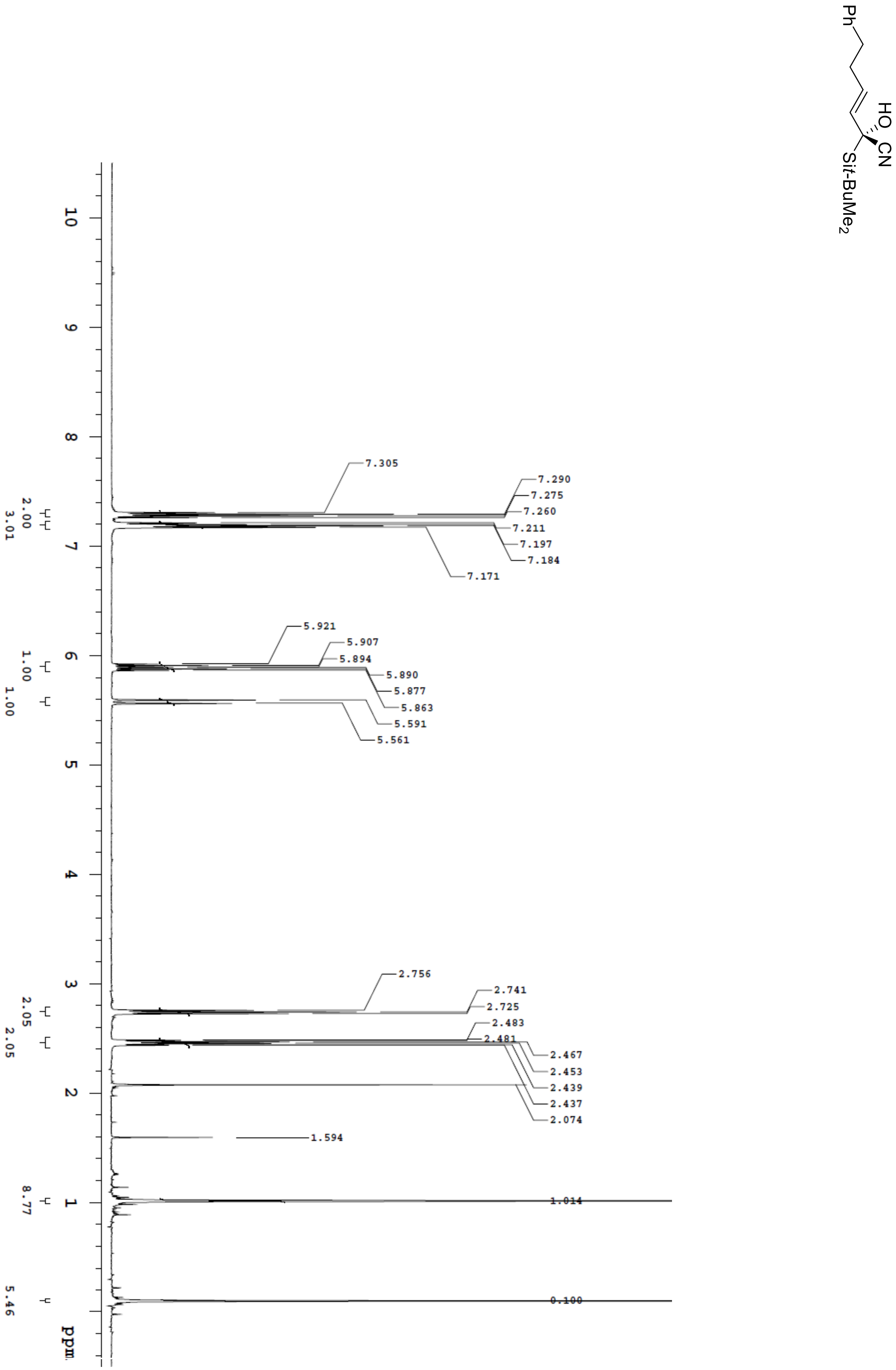

Supplementary Figure 95. ^{1}H NMR Spectrum of (*E*)-2-(*tert*-Butyldimethylsilyl)-2-hydroxy-6-phenylhex-3-enenitrile (**2u**)

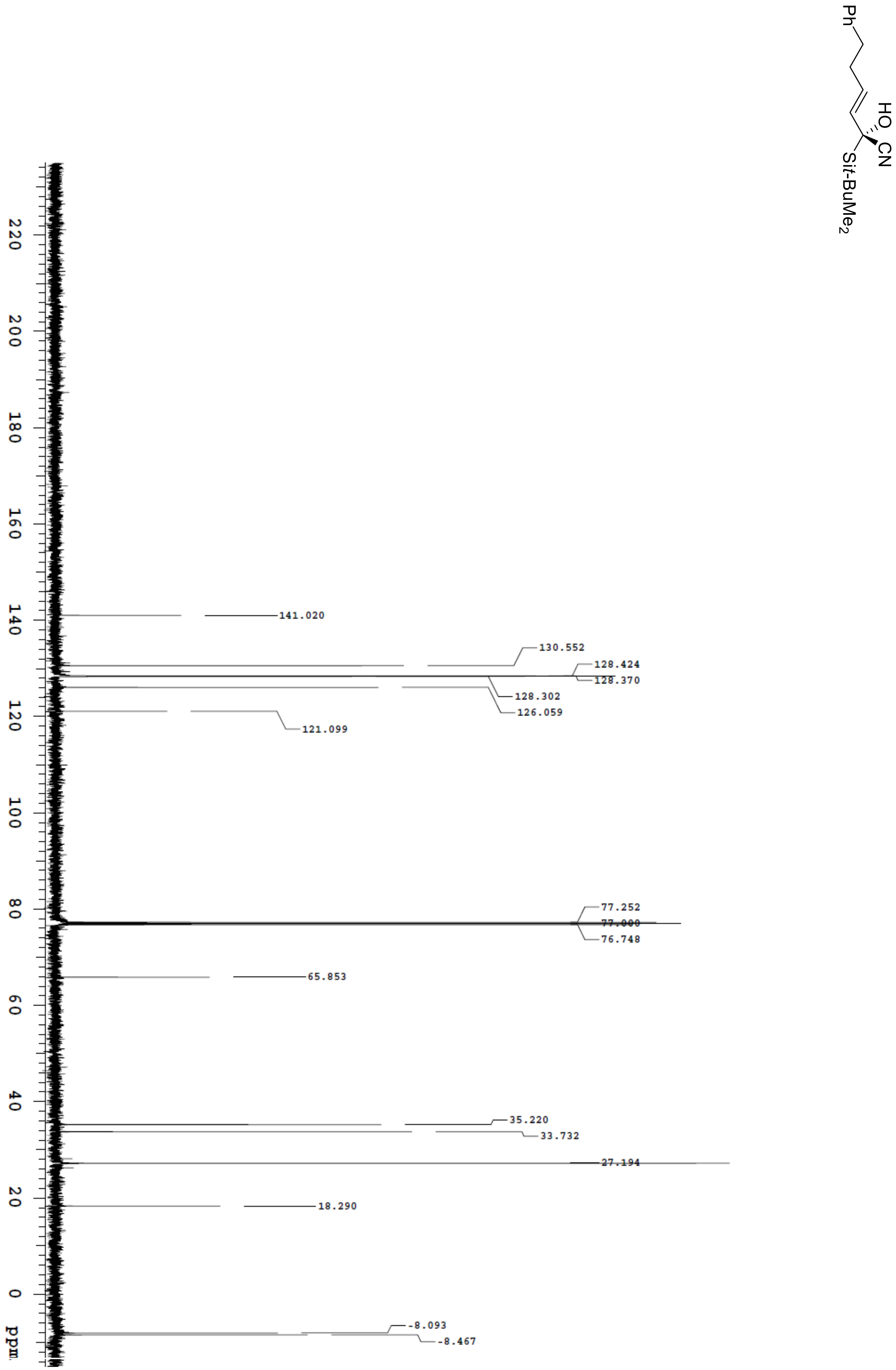

Supplementary Figure 96. ^{13}C NMR Spectrum of (*E*)-2-(*tert*-Butyldimethylsilyl)-2-hydroxy-6-phenylhex-3-enenitrile (**2u**)

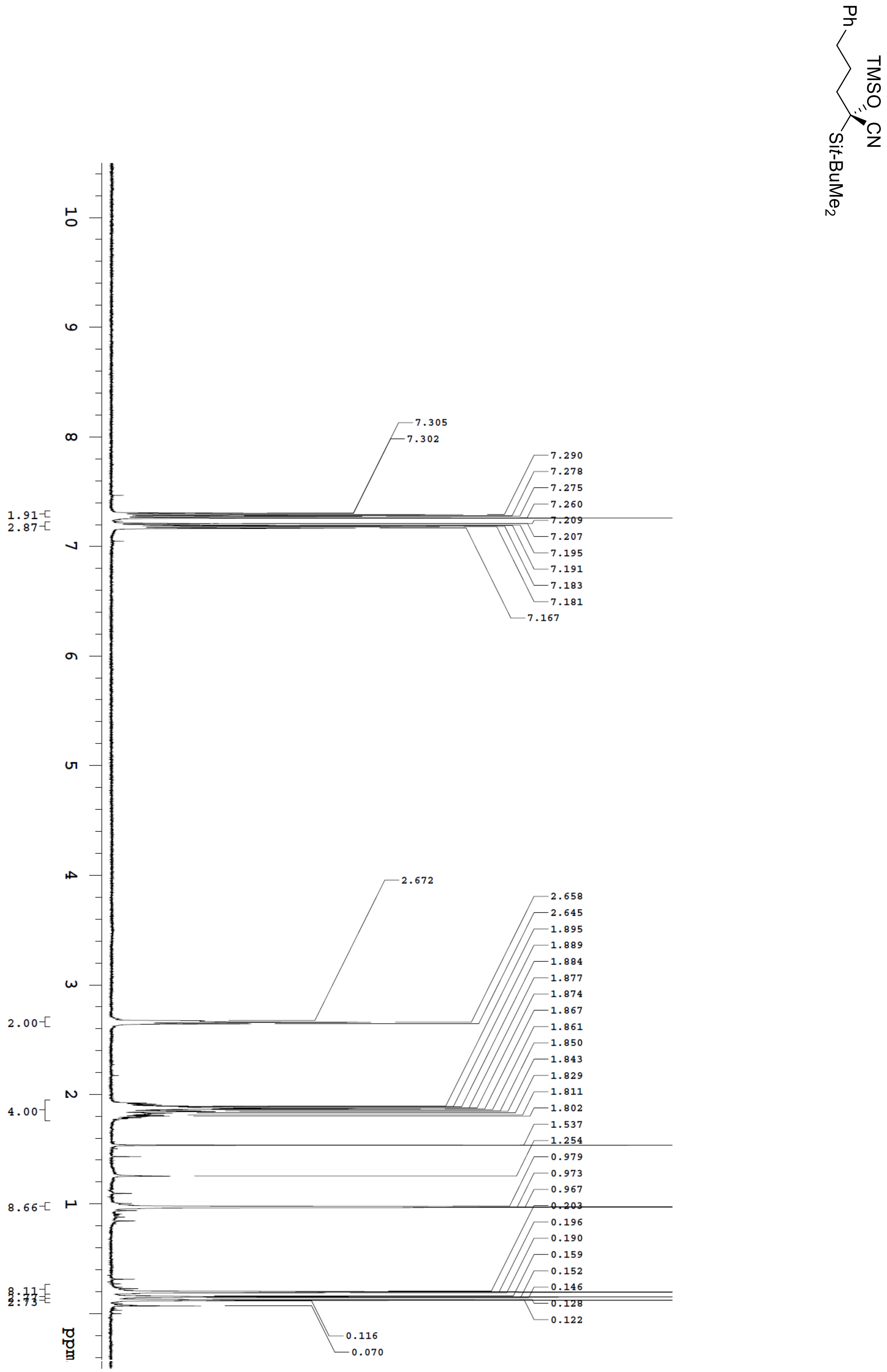

Supplementary Figure 97. ^{1}H NMR Spectrum of 2-(*tert*-Butyldimethylsilyl)-5-phenyl-2-((trimethylsilyl)oxy)pentanenitrile (**5a**)

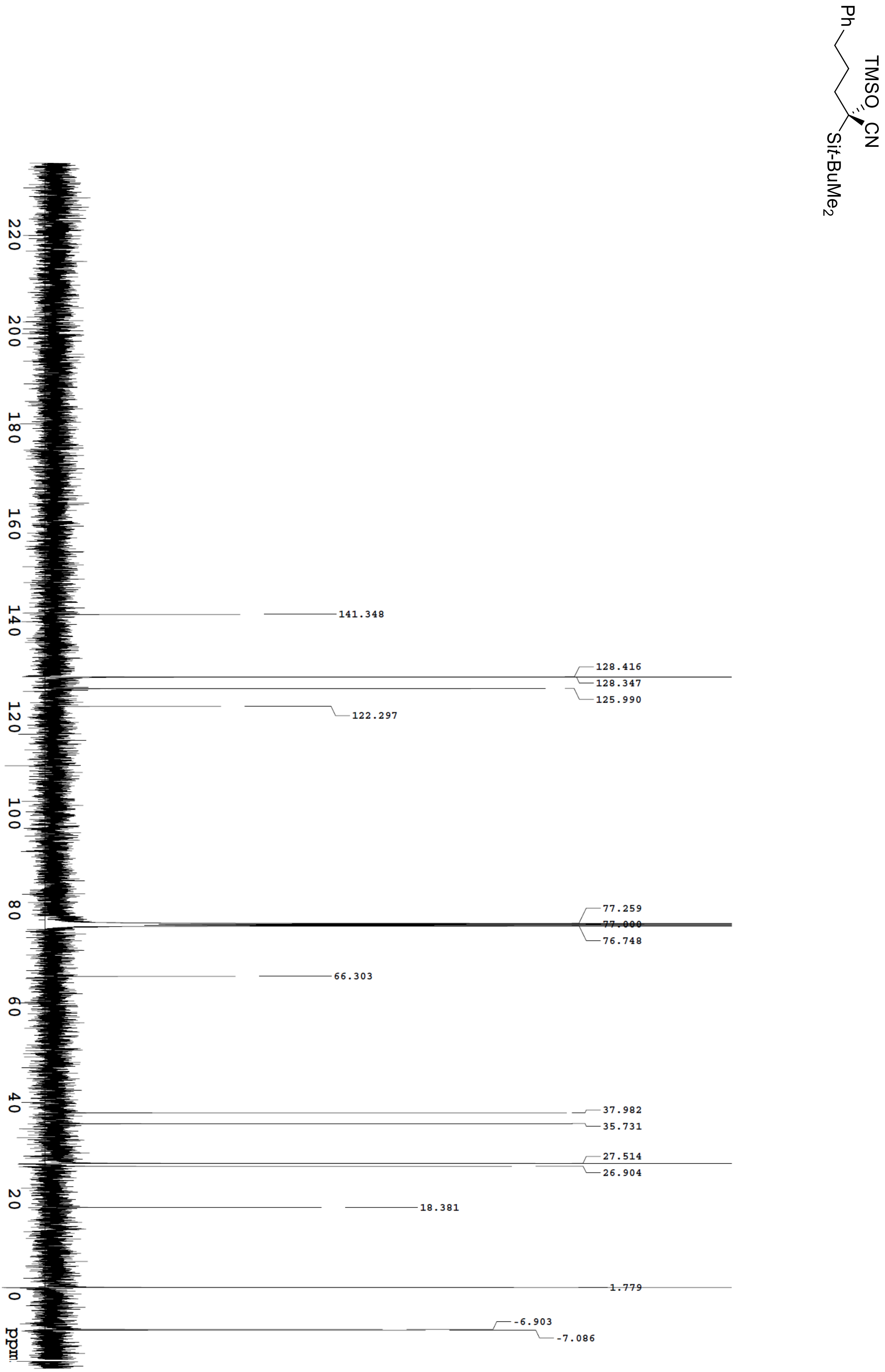

Supplementary Figure 98. ^{13}C NMR Spectrum of

2-(*tert*-Butyldimethylsilyl)-5-phenyl-2-((trimethylsilyl)oxy)pentanenitrile (**5a**)

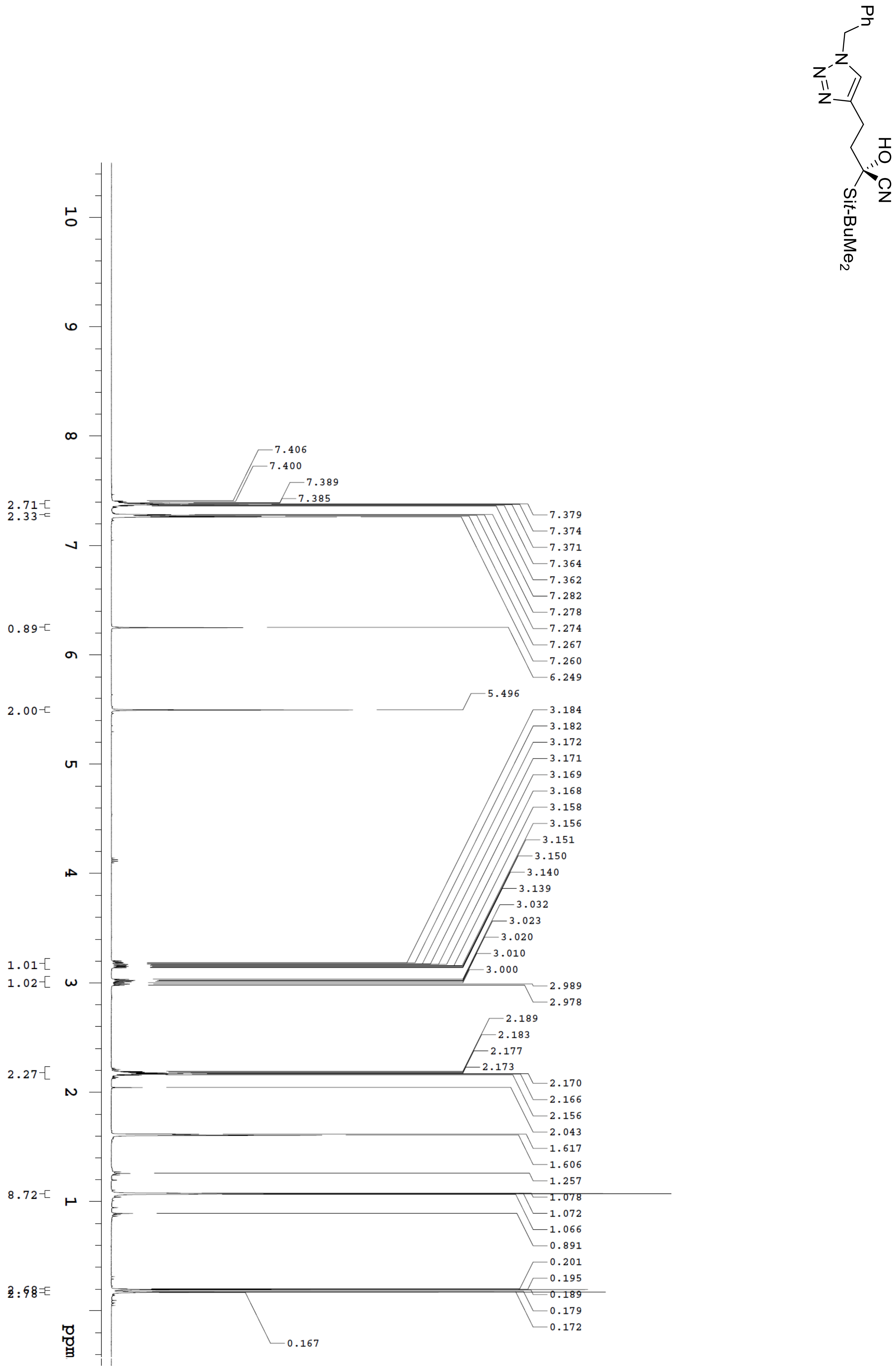

Supplementary Figure 99. ^{1}H NMR Spectrum of 4-(1-Benzyl-1*H*-1,2,3-triazol-4-yl)-2-(*tert*-butyldimethylsilyl)-2-hydroxybutanenitrile (**4**)

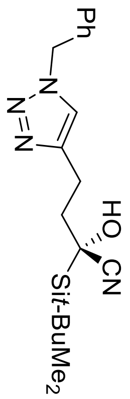

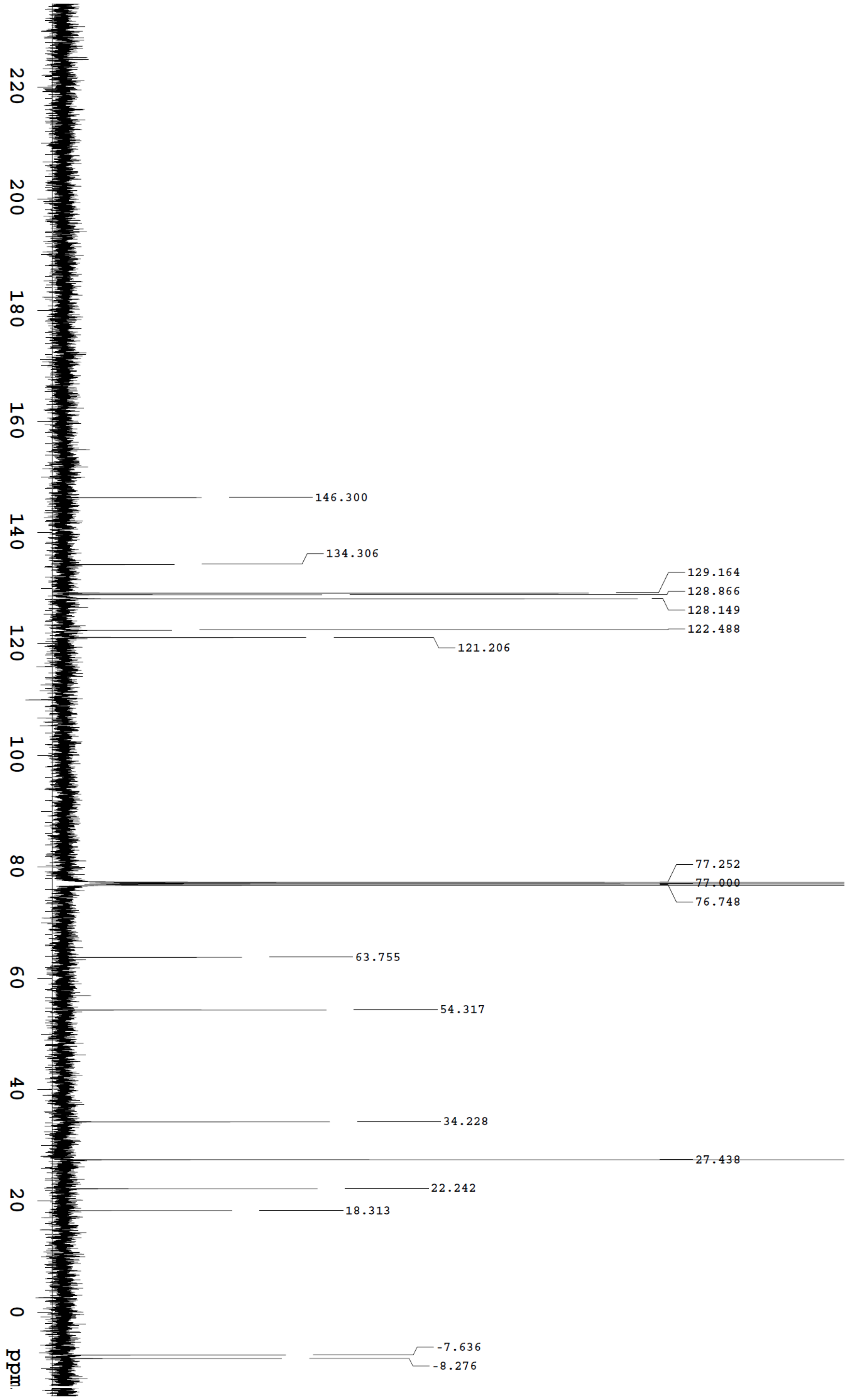

Supplementary Figure 100. ^{13}C NMR Spectrum of 4-(1-Benzyl-1*H*-1,2,3-triazol-4-yl)-2-(*tert*-butyldimethylsilyl)-2-hydroxybutanenitrile (**4**)

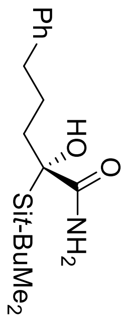

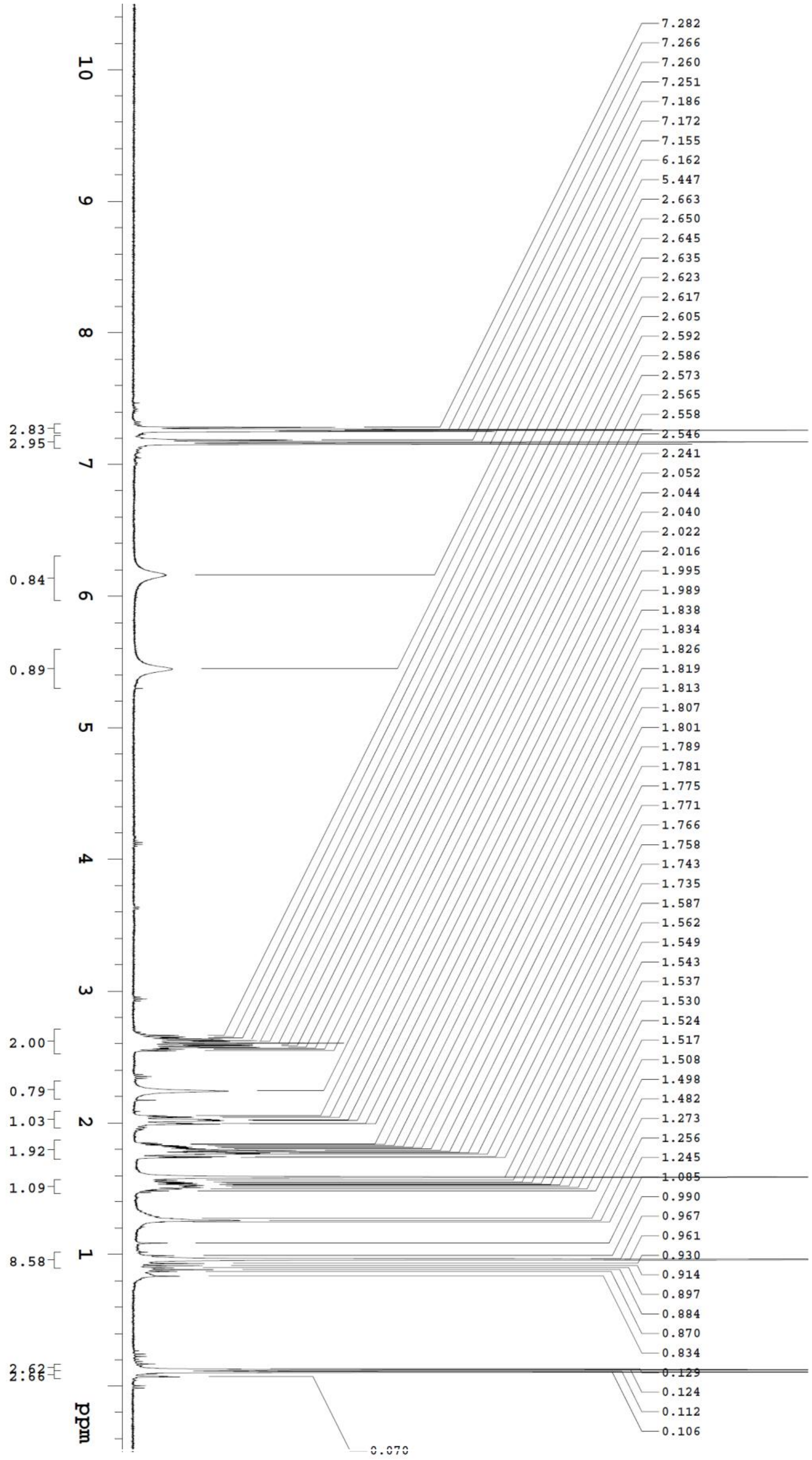

Supplementary Figure 101. ^{1}H NMR Spectrum of 2-(*tert*-Butyldimethylsilyl)-2-hydroxy-5-phenylpentanamide (**8**)

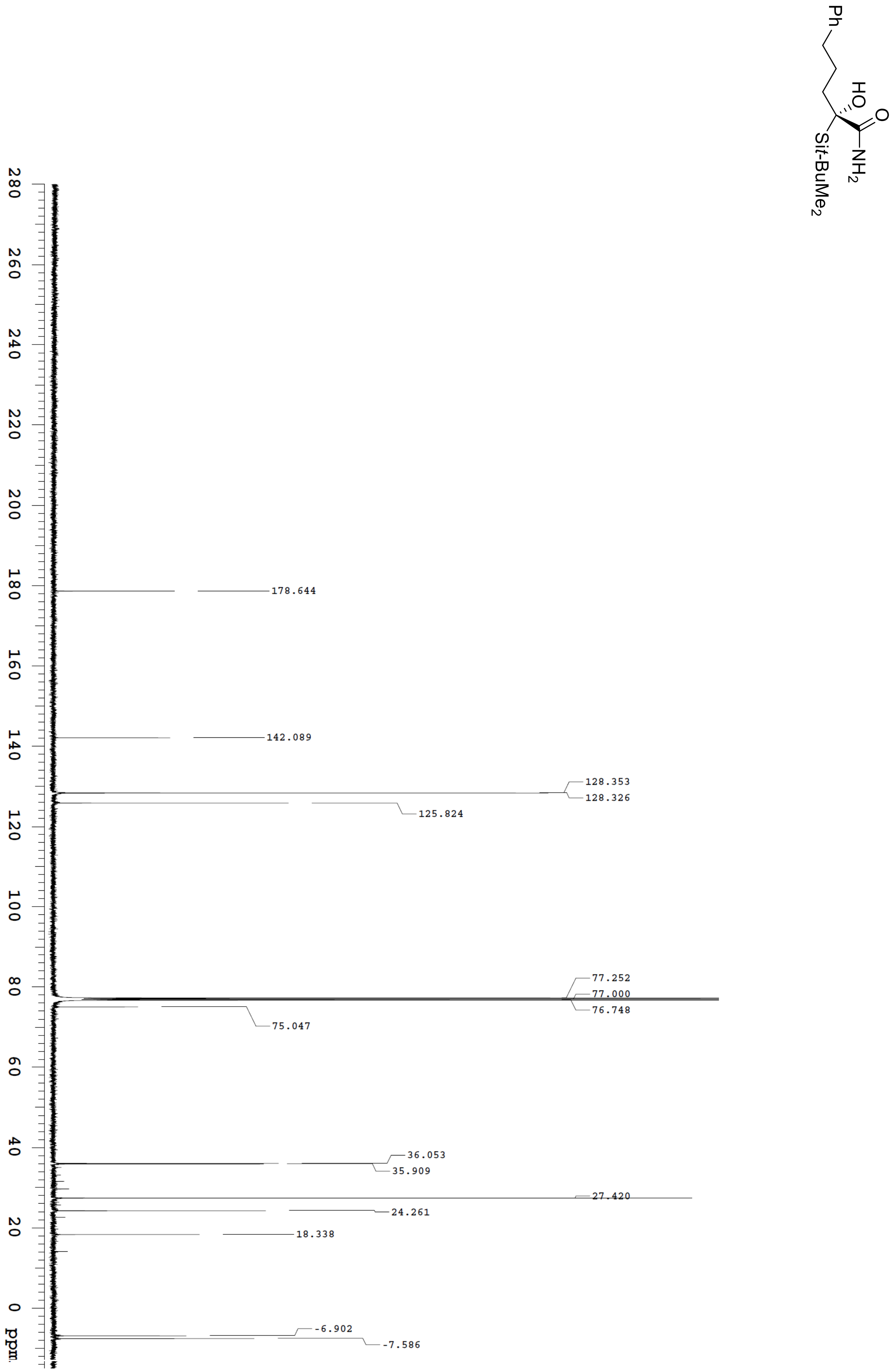

Supplementary Figure 102. ^{13}C NMR Spectrum of 2-(*tert*-Butyldimethylsilyl)-2-hydroxy-5-phenylpentanamide (**8**)

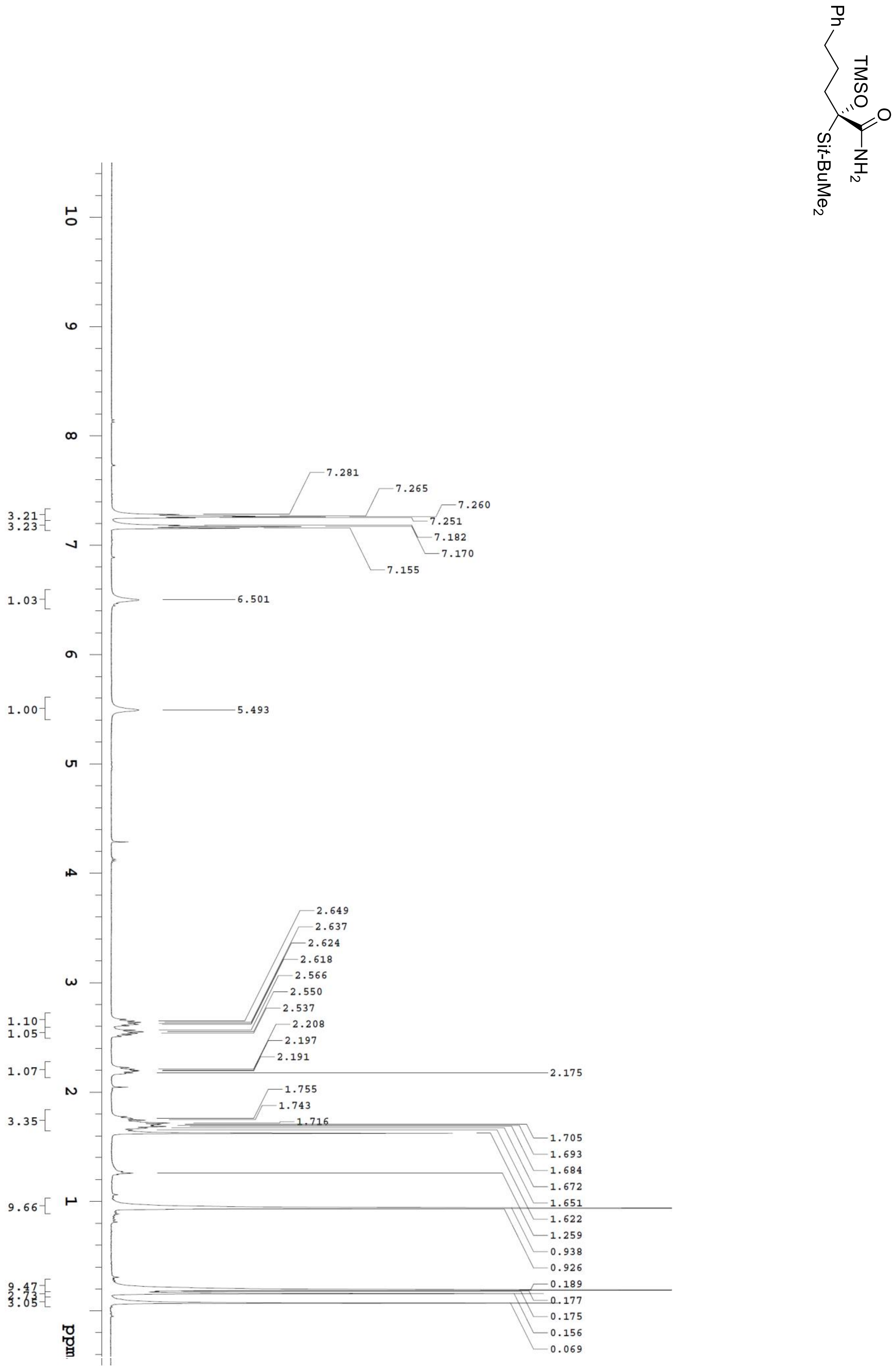

Supplementary Figure 103. ^{1}H NMR Spectrum of 2-(*tert*-Butyldimethylsilyl)-5-phenyl-2-((trimethylsilyl)oxy)pentanamide (**10**)

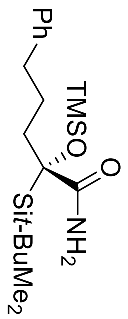

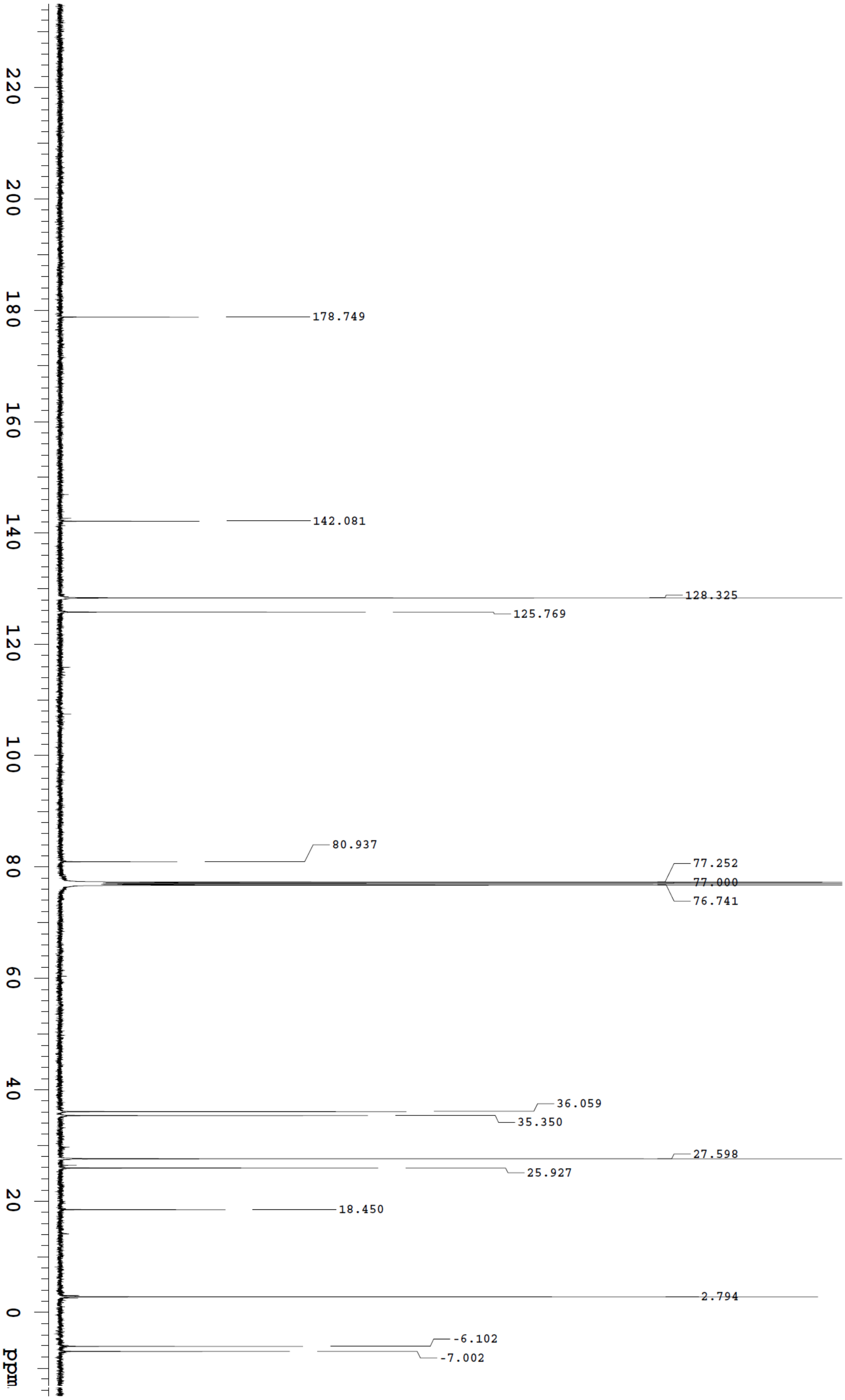

Supplementary Figure 104. ^{13}C NMR Spectrum of 2-(*tert*-Butyldimethylsilyl)-5-phenyl-2-((trimethylsilyl)oxy)pentanamide (**10**)

HPLC Chromatogram Profiles

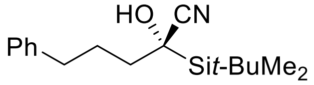

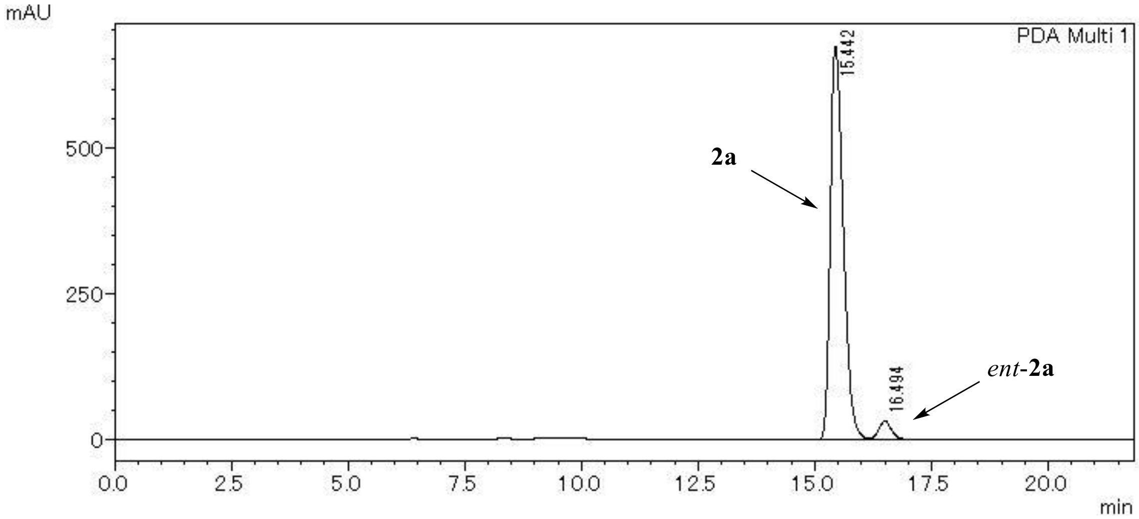

peak	retention time (min)	area %
1	15.442	95.521
2	16.494	4.479

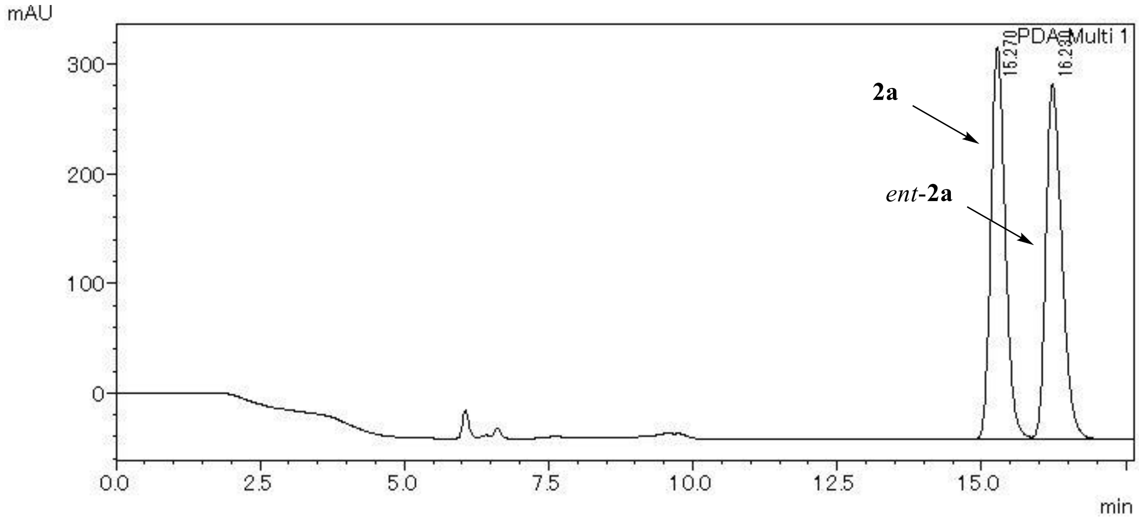

peak	retention time (min)	area %
1	15.270	50.059
2	16.230	49.941

Supplementary Figure 105. HPLC Chromatogram Profiles of (*S*)-2-(*tert*-Butyldimethylsilyl)-2-hydroxy-5-phenylpentanenitrile (**2a**)

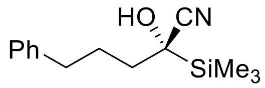

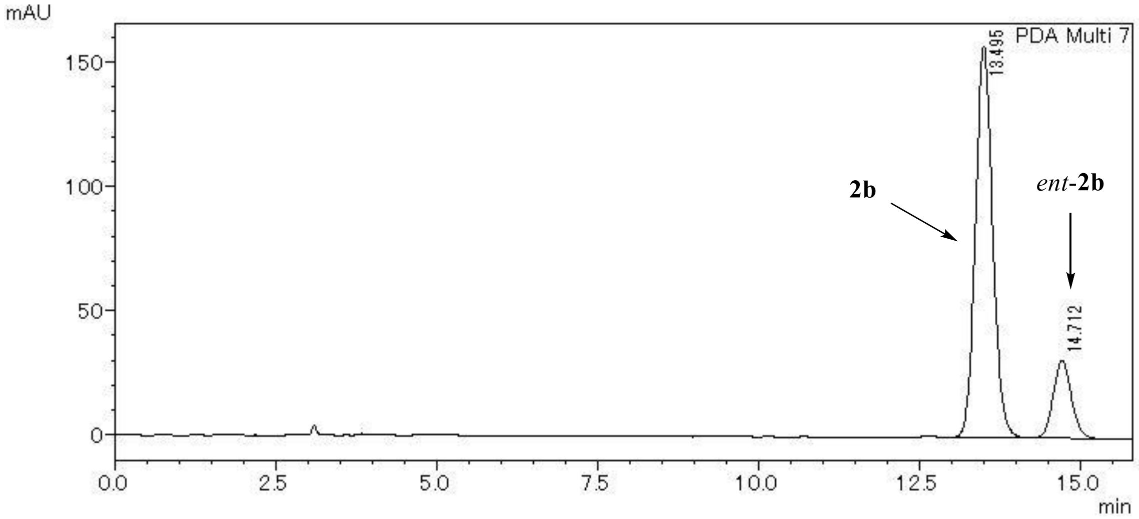

peak	retention time (min)	area %
1	13.495	82.540
2	14.712	17.460

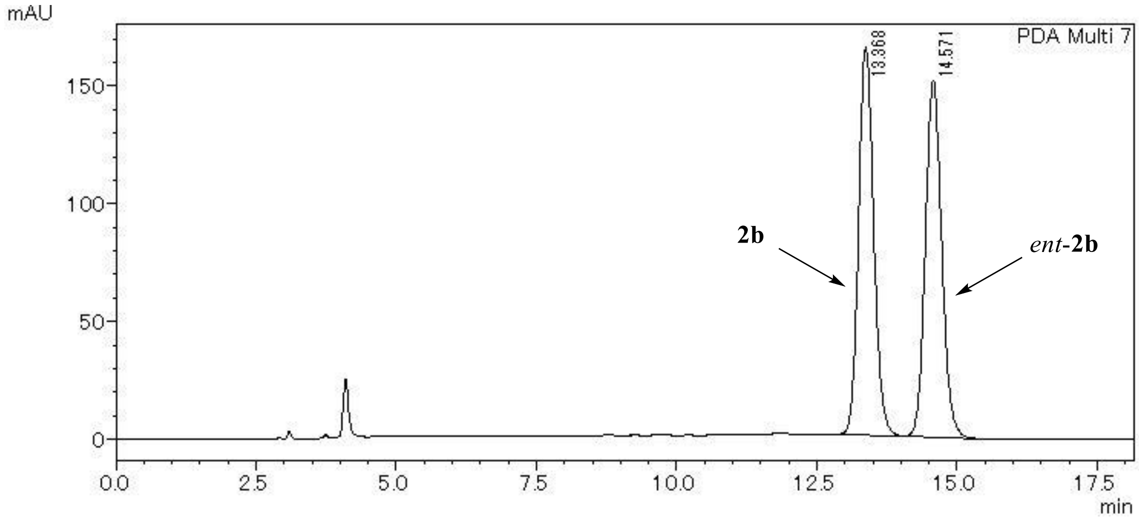

peak	retention time (min)	area %
1	13.368	50.113
2	14.571	49.887

Supplementary Figure 106. HPLC Chromatogram Profiles of 2-Hydroxy-5-phenyl-2-(trimethylsilyl)pentanenitrile (**2b**)

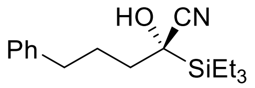

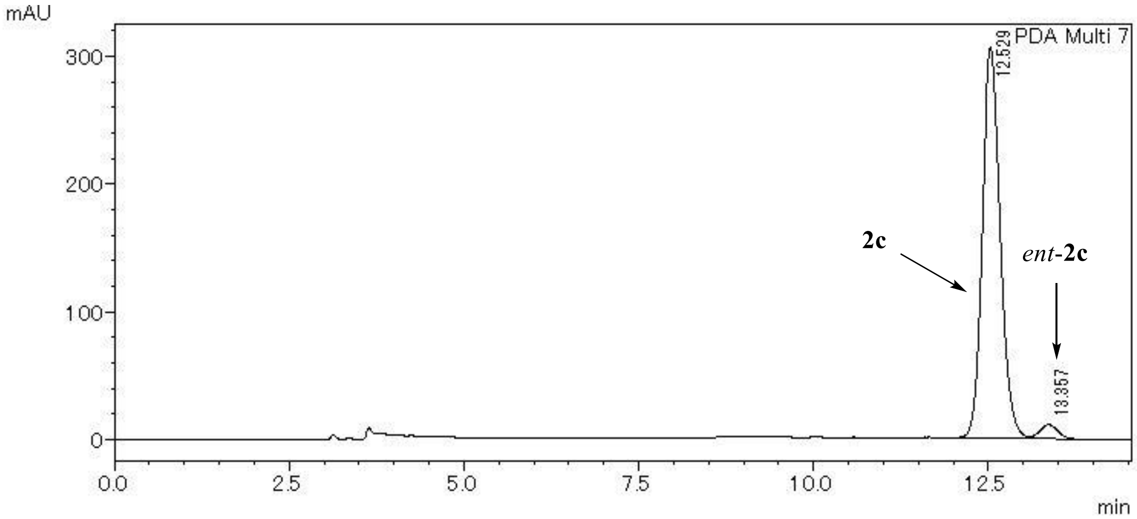

peak	retention time (min)	area %
1	12.529	96.327
2	13.357	3.673

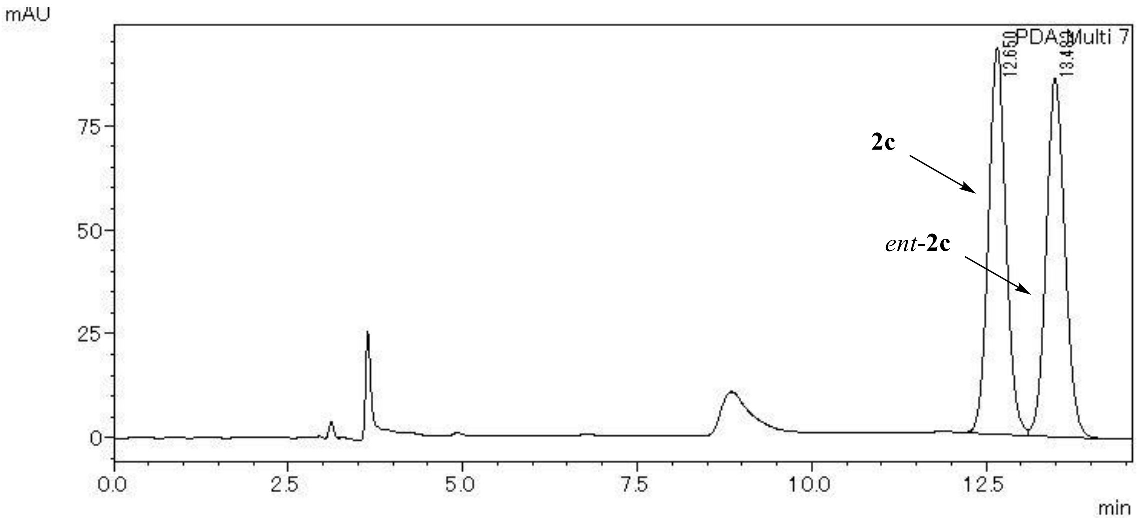

peak	retention time (min)	area %
1	12.650	50.242
2	13.482	49.758

Supplementary Figure 107. HPLC Chromatogram Profiles of 2-Hydroxy-5-phenyl-2-(triethylsilyl)pentanenitrile (**2c**)

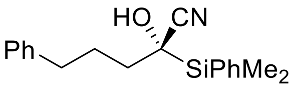

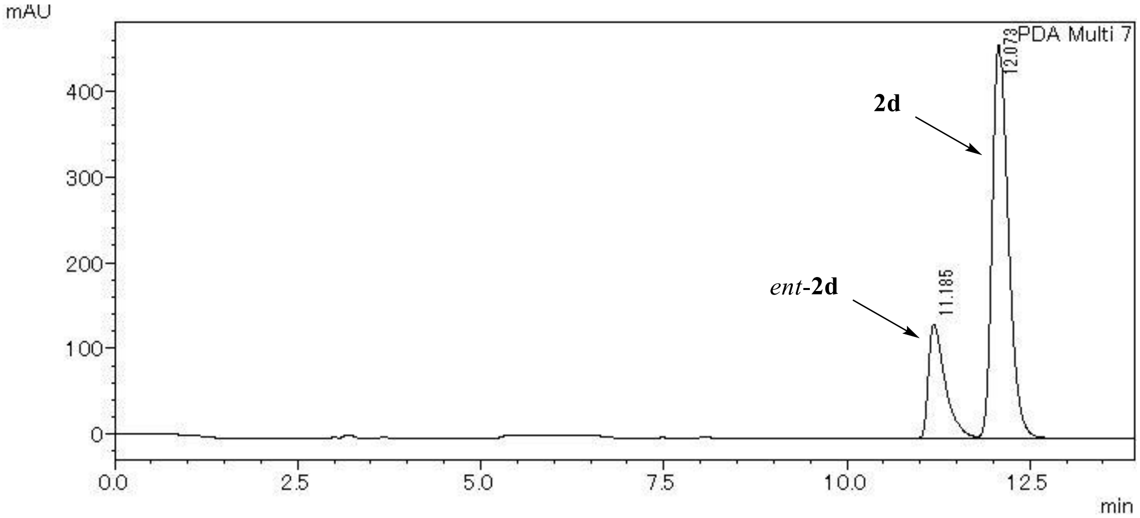

peak	retention time (min)	area %
1	11.185	23.628
2	12.073	76.372

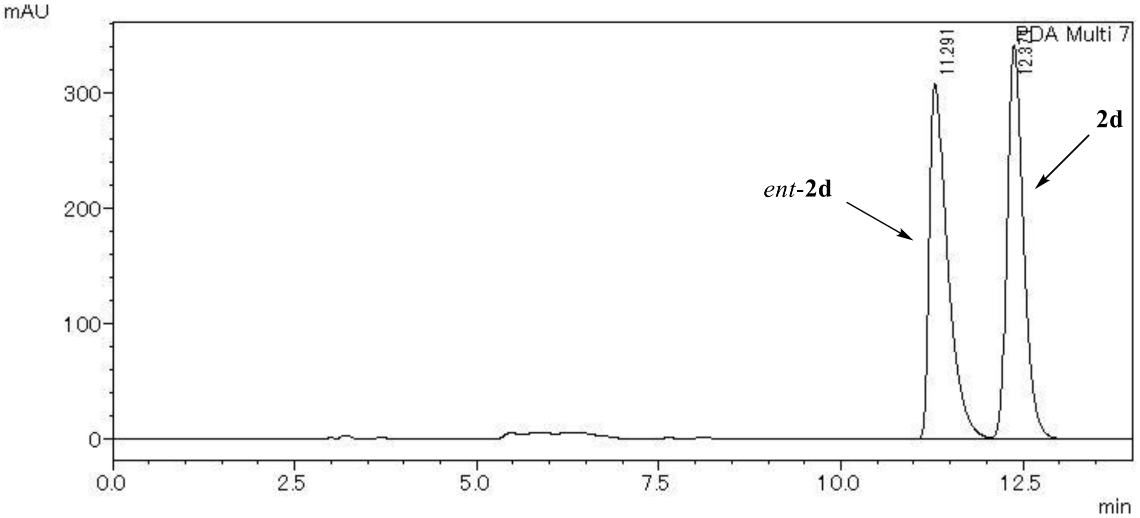

peak	retention time (min)	area %
1	11.291	49.929
2	12.371	50.071

Supplementary Figure 108. HPLC Chromatogram Profiles of 2-(Dimethyl(phenyl)silyl)-2-hydroxy-5-phenylpentanenitrile (**2d**)

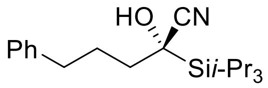

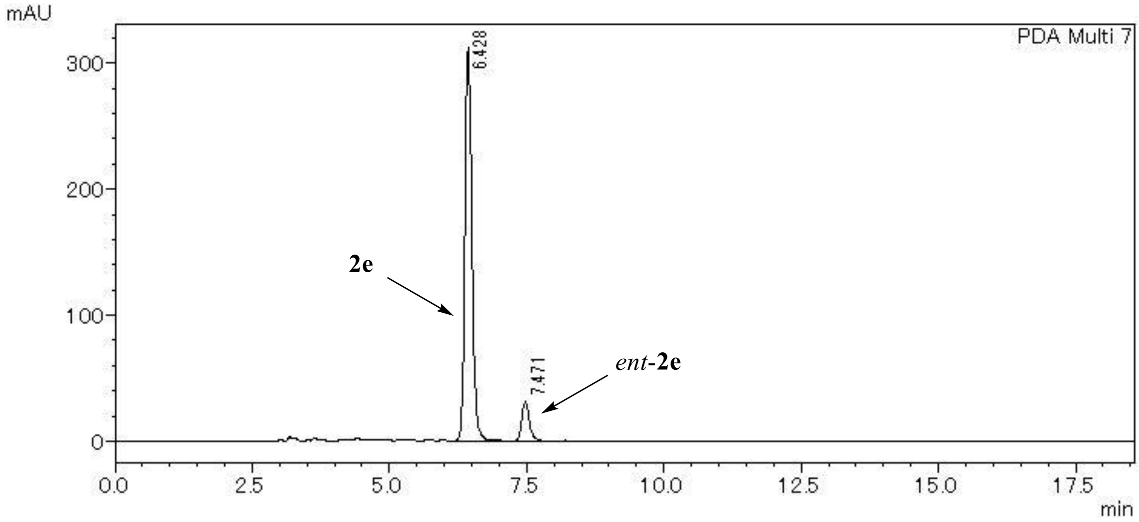

peak	retention time (min)	area %
1	6.428	90.042
2	7.471	9.958

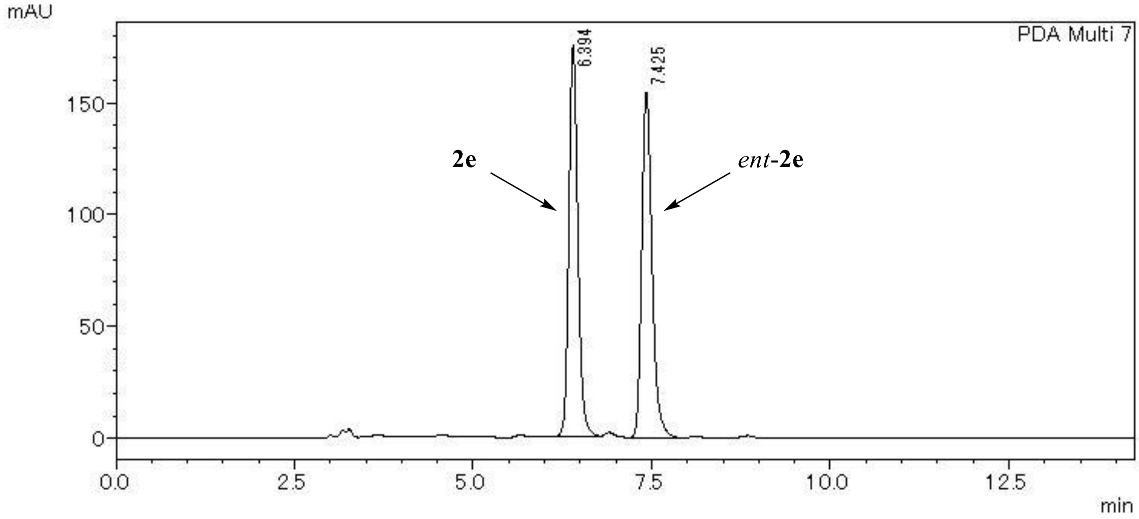

peak	retention time (min)	area %
1	6.394	49.785
2	7.425	50.215

Supplementary Figure 109. HPLC Chromatogram Profiles of 2-Hydroxy-5-phenyl-2-(triisopropylsilyl)pentanenitrile (**2e**)

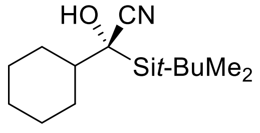

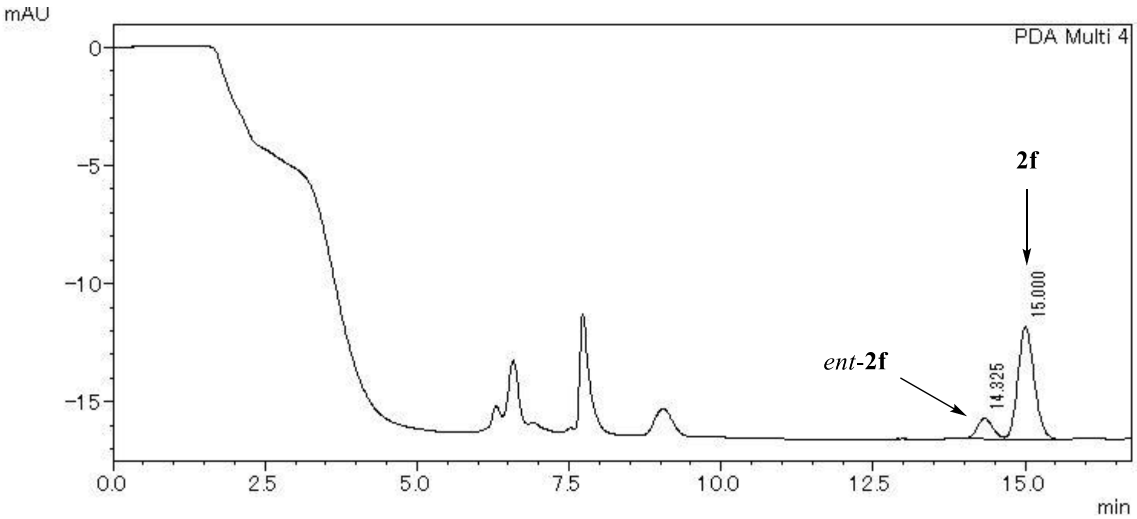

peak	retention time (min)	area %
1	14.325	14.760
2	15.000	85.240

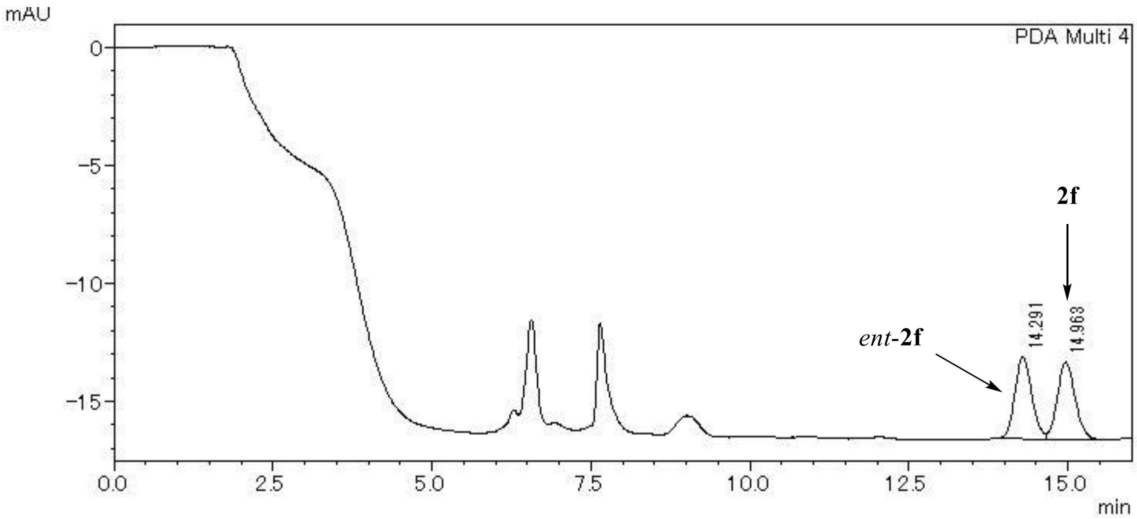

peak	retention time (min)	area %
1	14.291	50.182
2	14.963	49.818

Supplementary Figure 110. HPLC Chromatogram Profiles of 2-(*tert*-Butyldimethylsilyl)-2-cyclohexyl-2-hydroxyacetonitrile (**2f**)

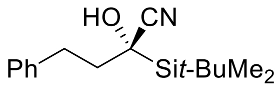

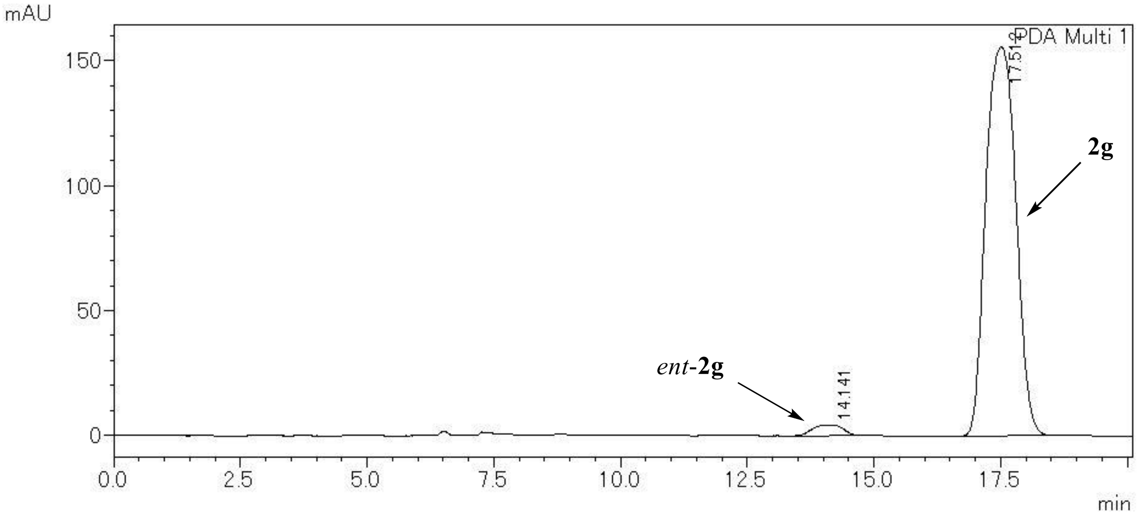

peak	retention time (min)	area %
1	14.141	2.745
2	17.512	97.255

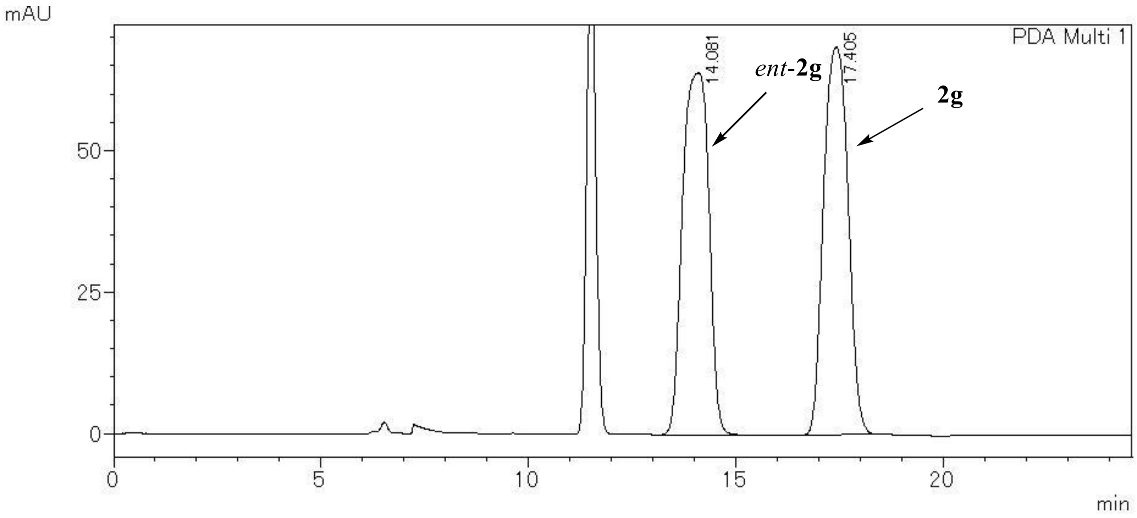

peak	retention time (min)	area %
1	14.081	50.088
2	17.405	49.912

Supplementary Figure 111. HPLC Chromatogram Profiles of 2-(*tert*-Butyldimethylsilyl)-2-hydroxy-4-phenylbutanenitrile (**2g**)

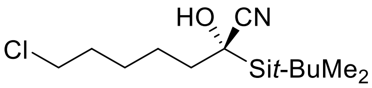

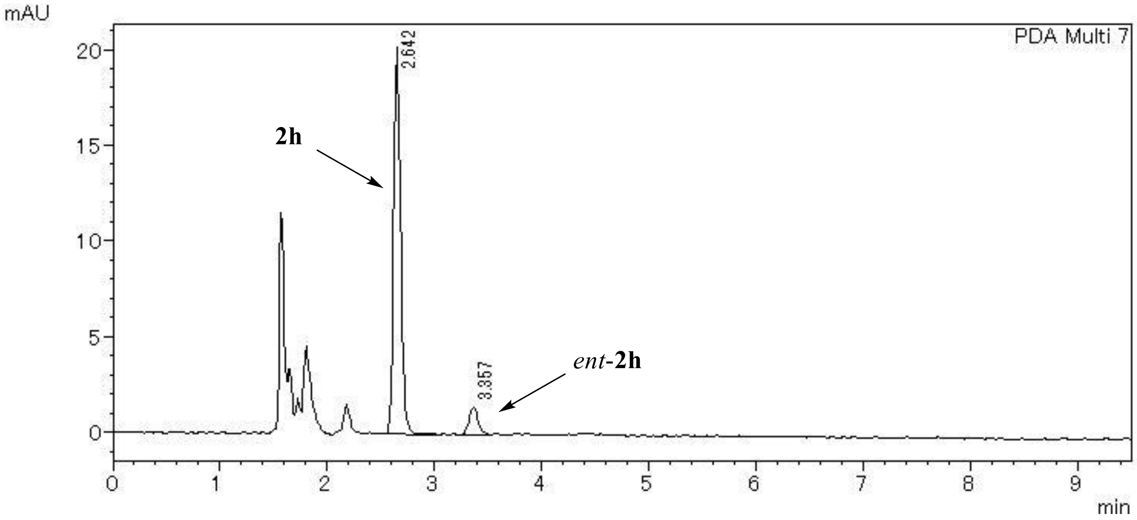

peak	retention time (min)	area %
1	2.642	91.116
2	3.357	8.884

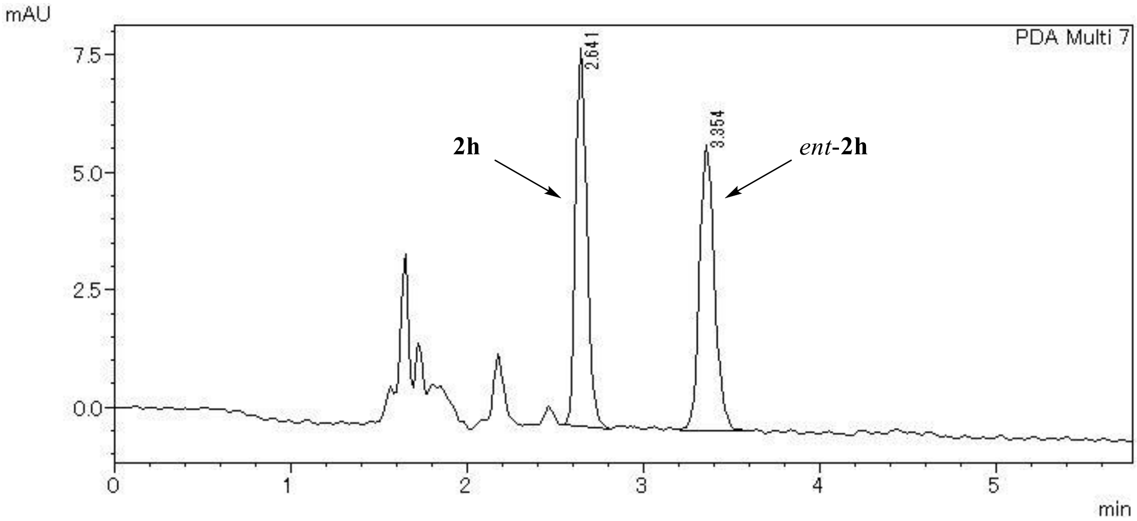

peak	retention time (min)	area %
1	2.641	49.864
2	3.354	50.136

Supplementary Figure 112. HPLC Chromatogram Profiles of 2-(*tert*-Butyldimethylsilyl)-7-chloro-2-hydroxyheptanenitrile (**2h**)

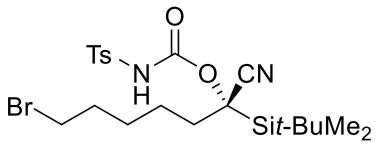

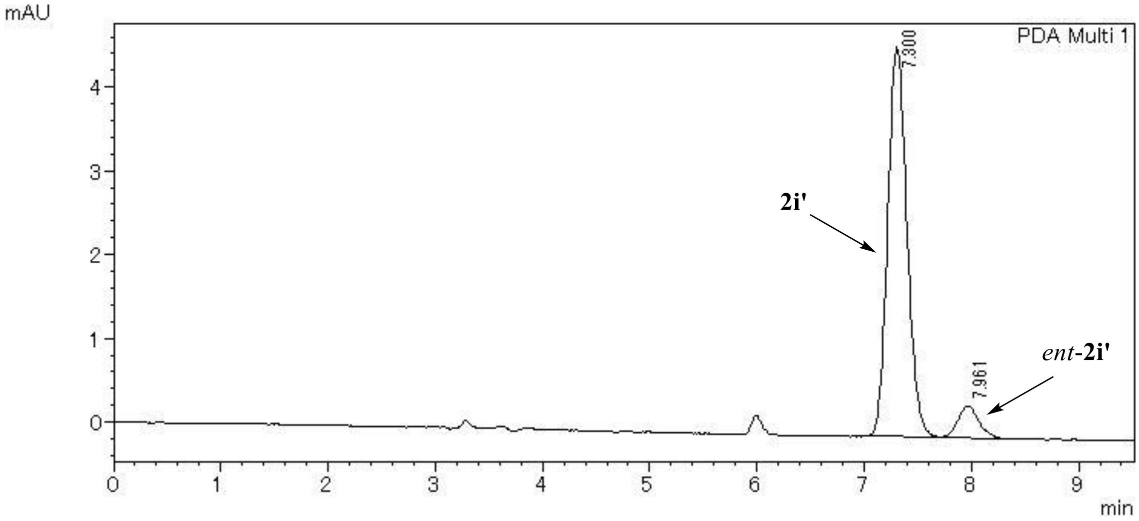

peak	retention time (min)	area %
1	7.300	91.901
2	7.961	8.099

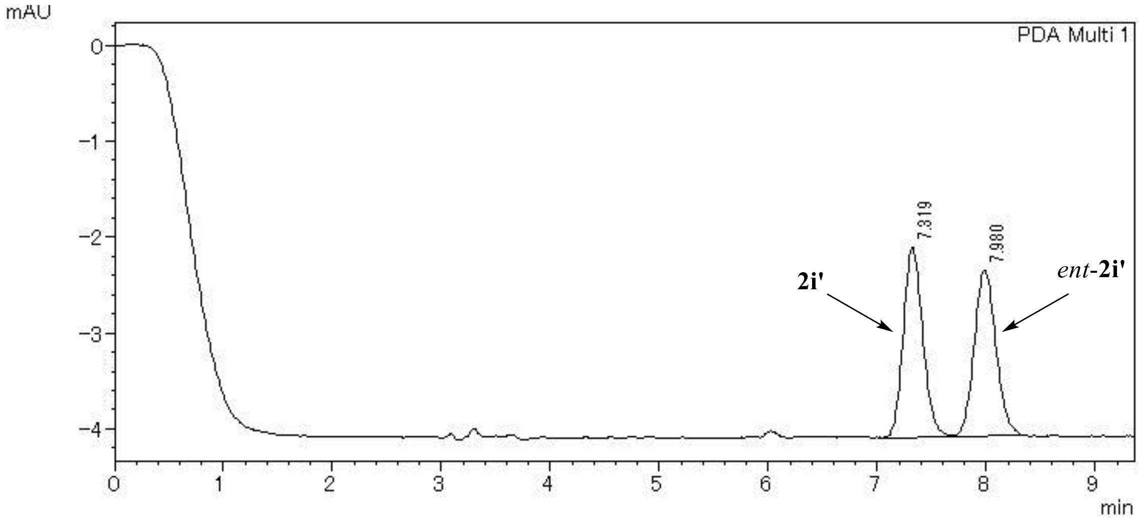

peak	retention time (min)	area %
1	7.319	50.582
2	7.980	49.726

Supplementary Figure 113. HPLC Chromatogram Profiles of 6-Bromo-1-(*tert*-butyldimethylsilyl)-1-cyanohexyl tosylcarbamate (**2i'**)

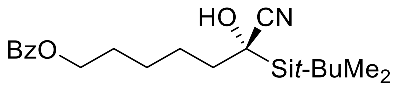

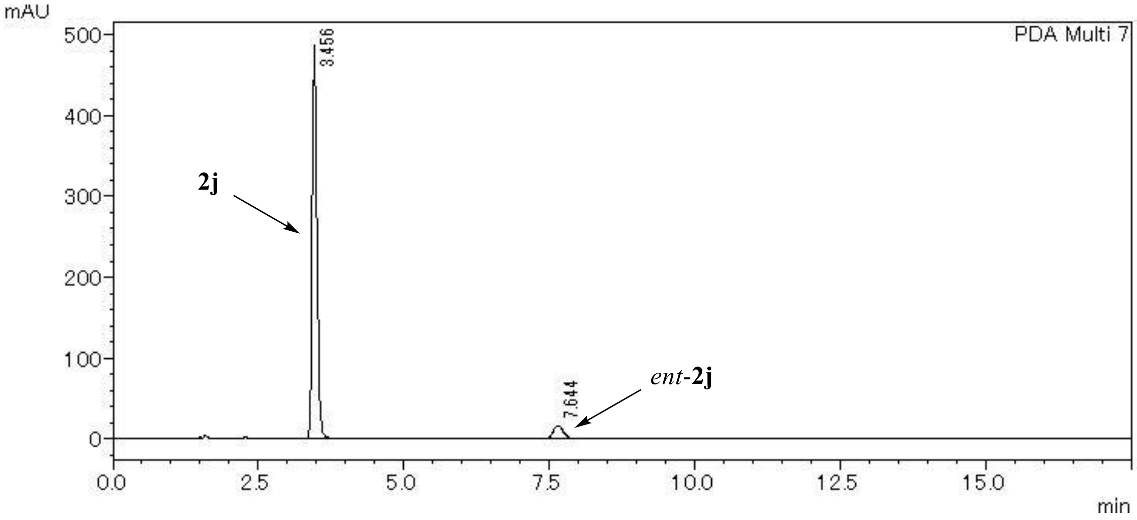

peak	retention time (min)	area %
1	3.456	93.053
2	7.644	6.947

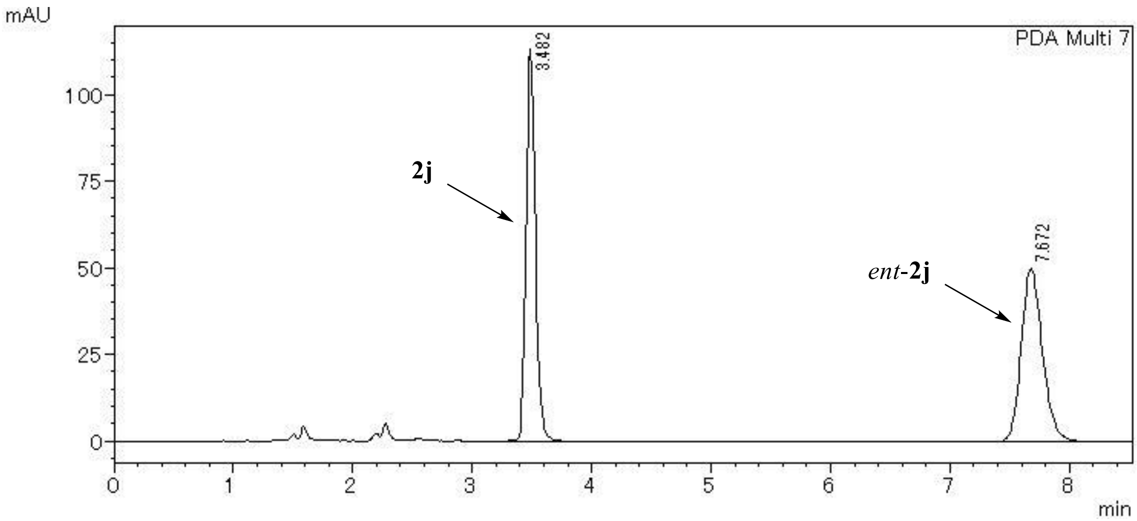

peak	retention time (min)	area %
1	3.482	49.733
2	7.672	50.267

Supplementary Figure 114. HPLC Chromatogram Profiles of 6-(*tert*-Butyldimethylsilyl)-6-cyano-6-hydroxyhexyl benzoate (**2j**)

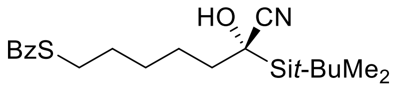

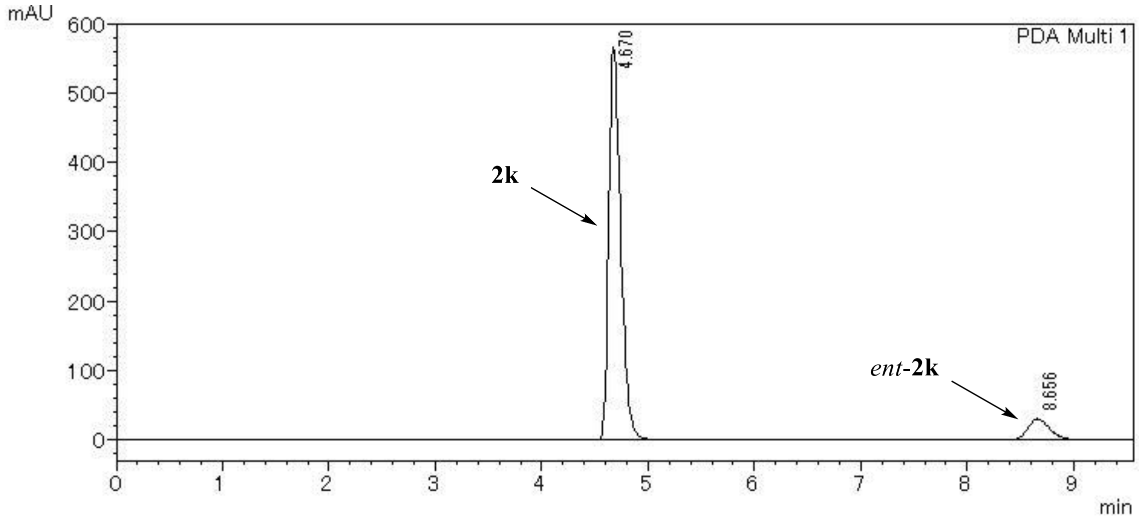

peak	retention time (min)	area %
1	4.670	91.019
2	8.656	8.981

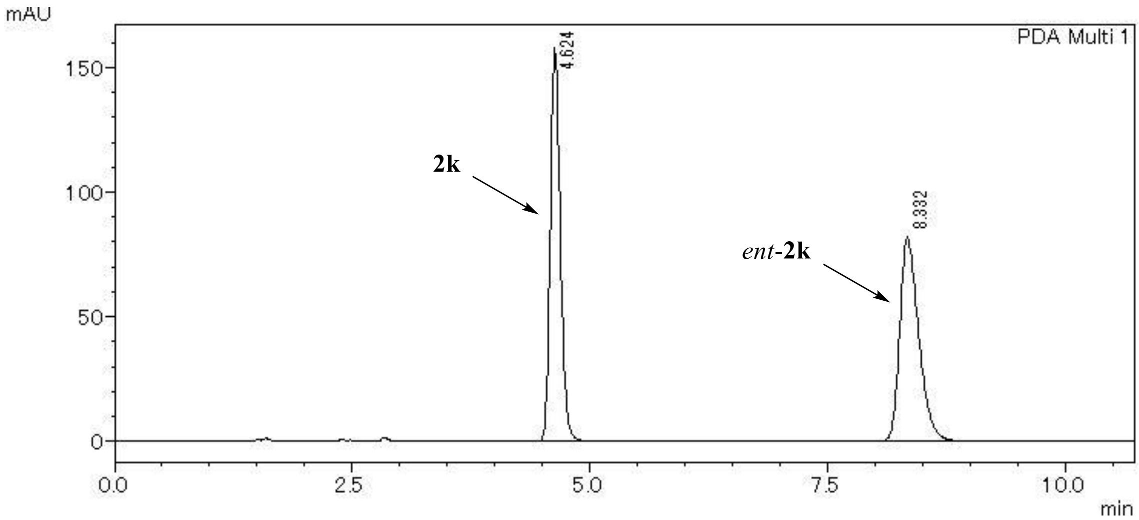

peak	retention time (min)	area %
1	4.624	50.084
2	8.332	49.916

Supplementary Figure 115. HPLC Chromatogram Profiles of *S*-(6-(*tert*-Butyldimethylsilyl)-6-cyano-6-hydroxyhexyl) benzothioate (**2k**)

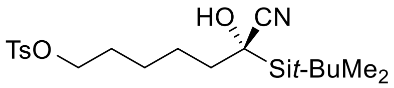

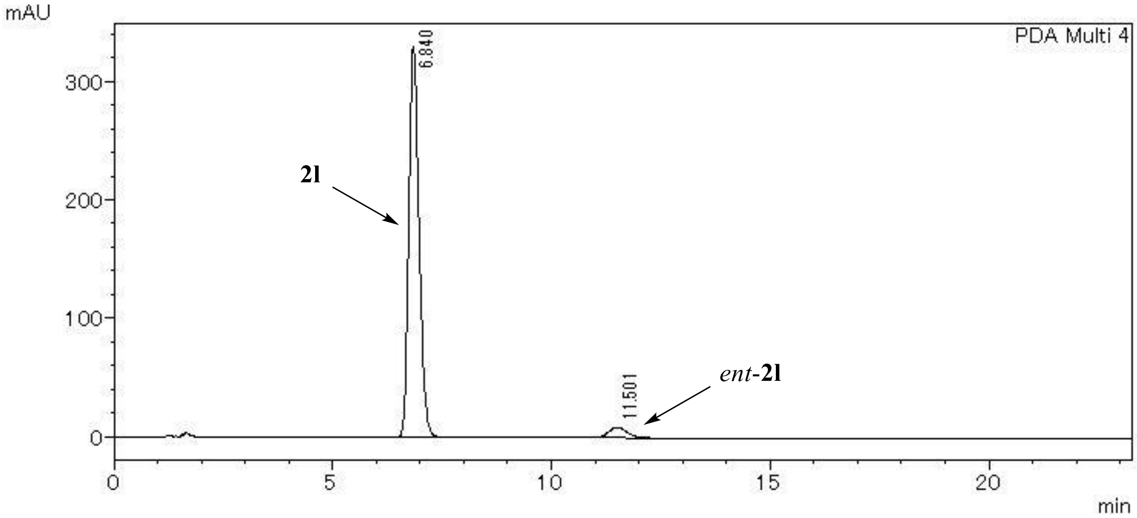

peak	retention time (min)	area %
1	6.840	95.467
2	11.501	4.533

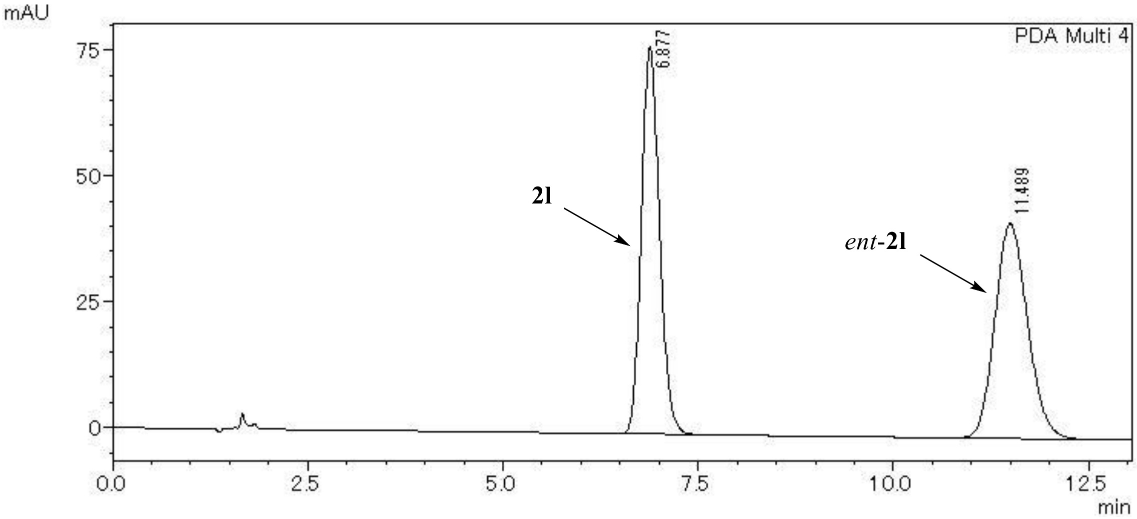

peak	retention time (min)	area %
1	6.877	50.093
2	11.489	49.907

Supplementary Figure 116. HPLC Chromatogram Profiles of 6-(*tert*-Butyldimethylsilyl)-6-cyano-6-hydroxyhexyl 4-methylbenzenesulfonate (**2l**)

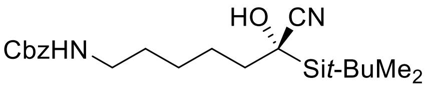

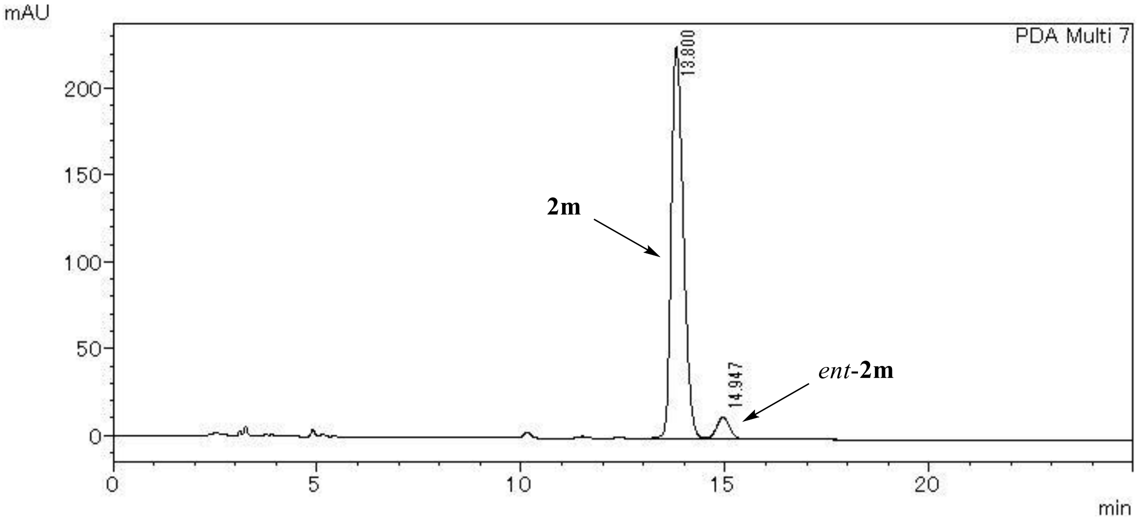

peak	retention time (min)	area %
1	13.800	94.418
2	14.947	5.582

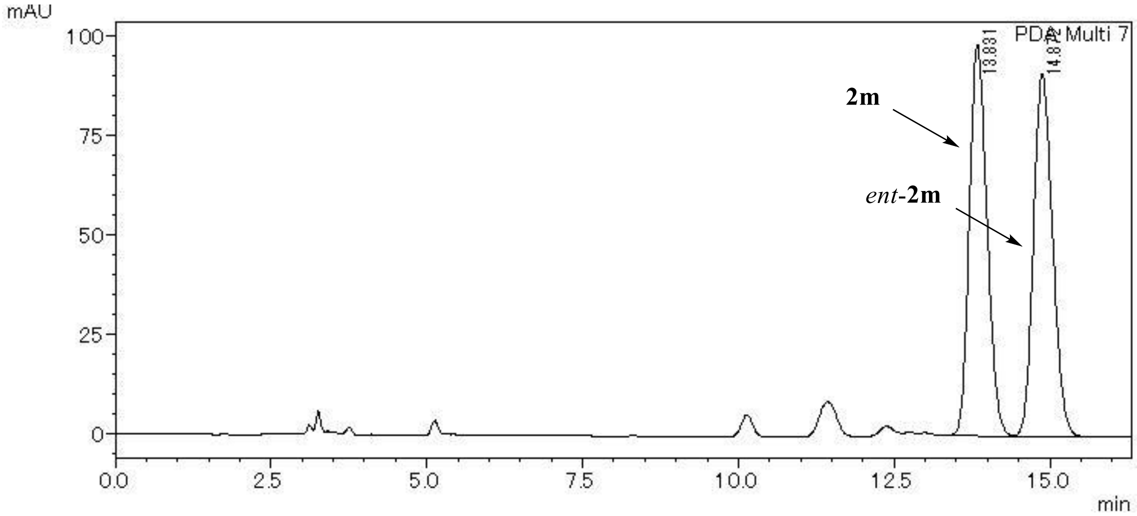

peak	retention time (min)	area %
1	13.831	49.902
2	14.872	50.098

Supplementary Figure 117. HPLC Chromatogram Profiles of Benzyl (6-(*tert*-butyldimethylsilyl)-6-cyano-6-hydroxyhexyl)carbamate (**2m**)

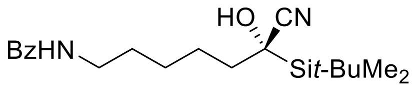

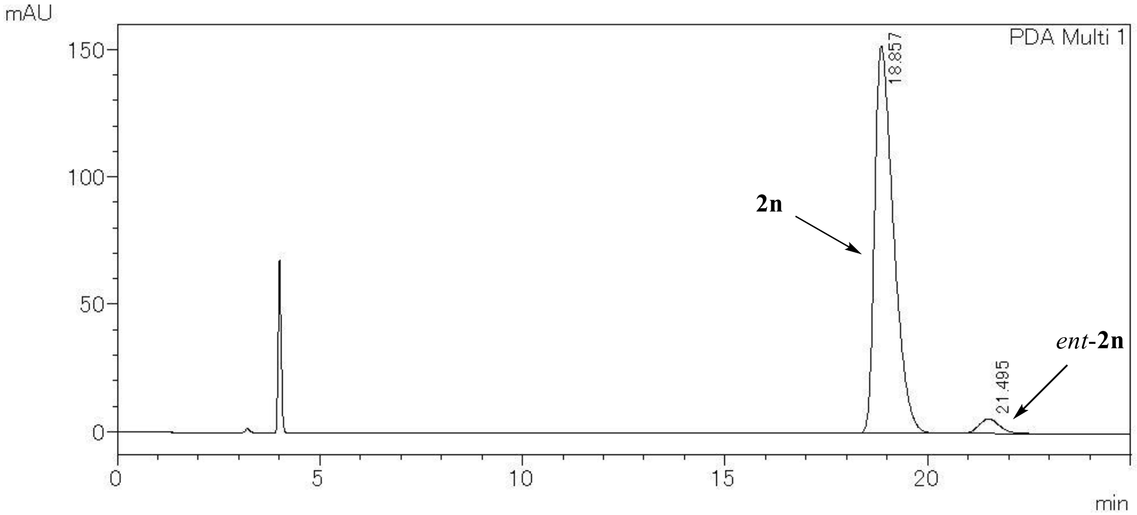

peak	retention time (min)	area %
1	18.857	96.080
2	21.495	3.920

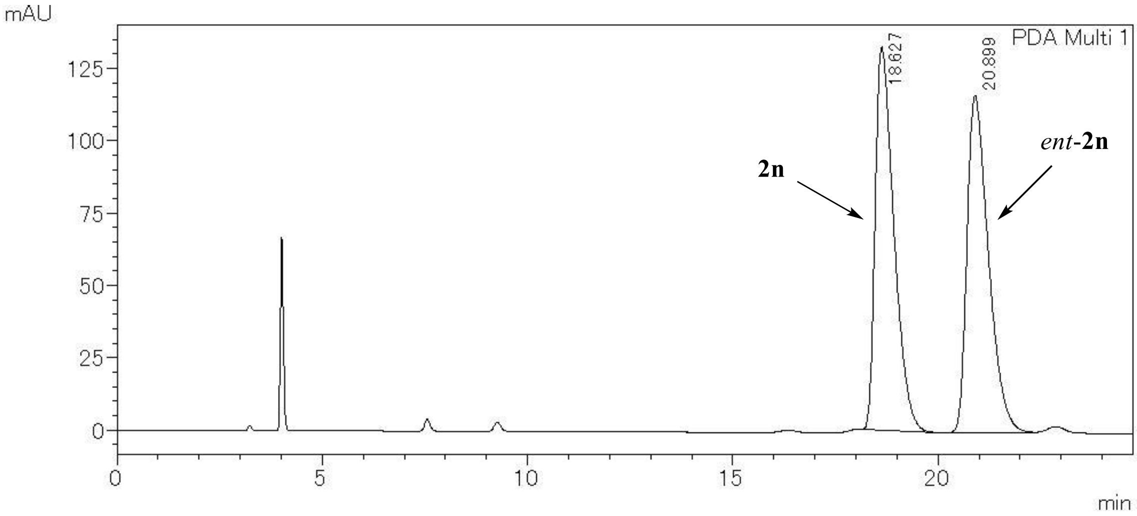

peak	retention time (min)	area %
1	18.627	49.871
2	20.899	50.129

Supplementary Figure 118. HPLC Chromatogram Profiles of *N*-(6-(*tert*-Butyldimethylsilyl)-6-cyano-6-hydroxyhexyl)benzamide (**2n**)

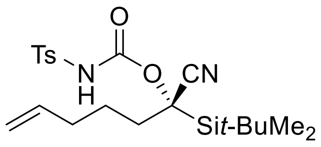

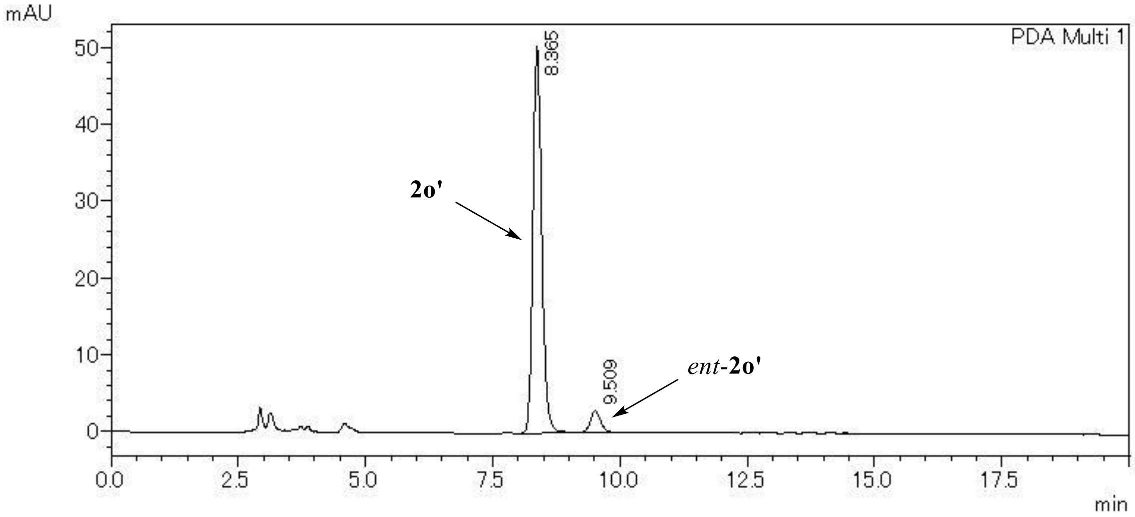

peak	retention time (min)	area %
1	8.365	93.985
2	9.509	6.015

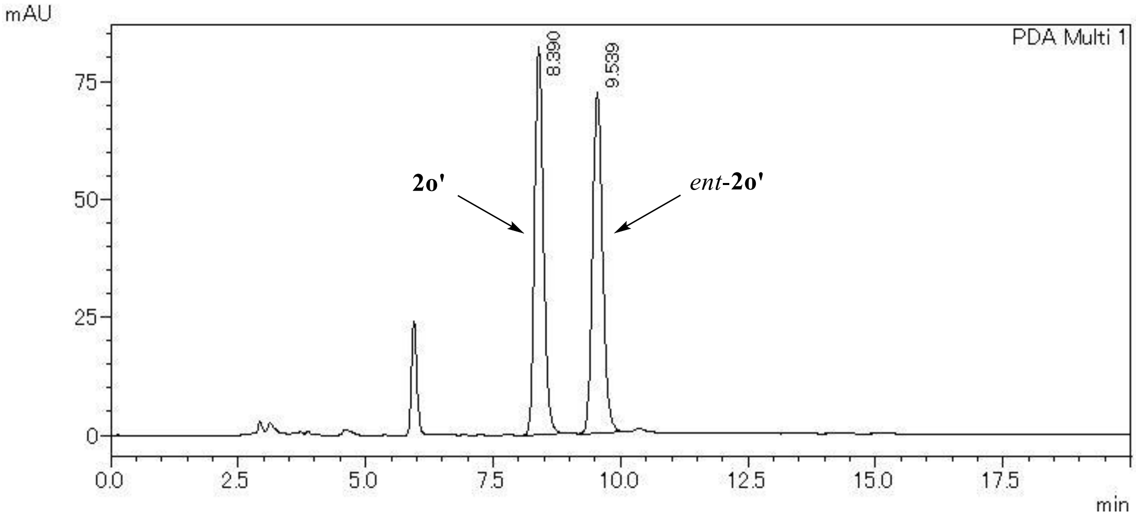

peak	retention time (min)	area %
1	8.390	49.912
2	9.539	50.088

Supplementary Figure 119. HPLC Chromatogram Profiles of 1-(*tert*-Butyldimethylsilyl)-1-cyanohex-5-en-1-yl tosylcarbamate (**2o'**)

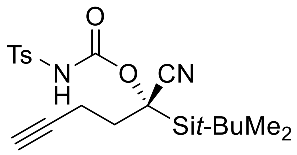

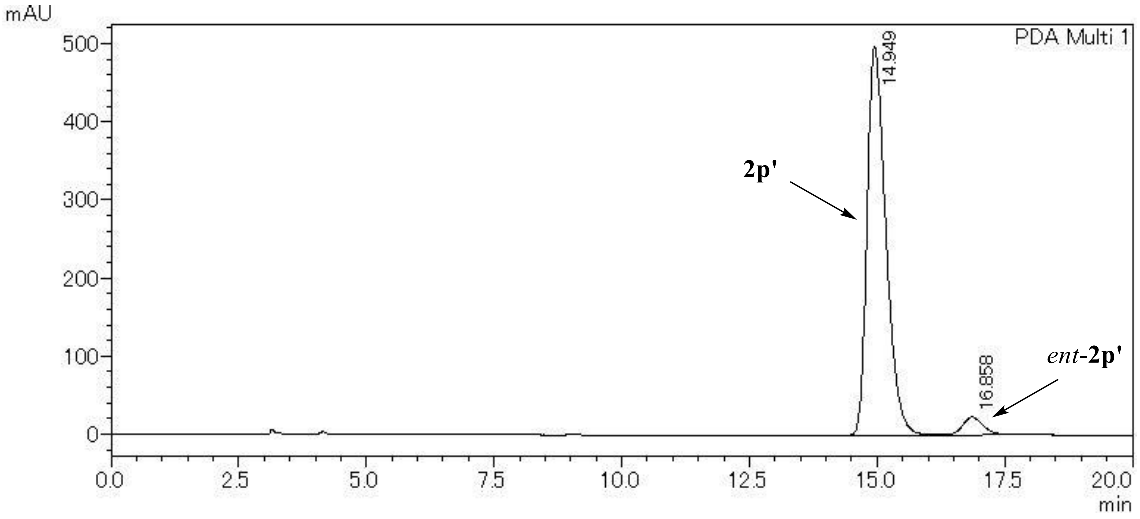

peak	retention time (min)	area %
1	14.949	95.604
2	16.858	4.396

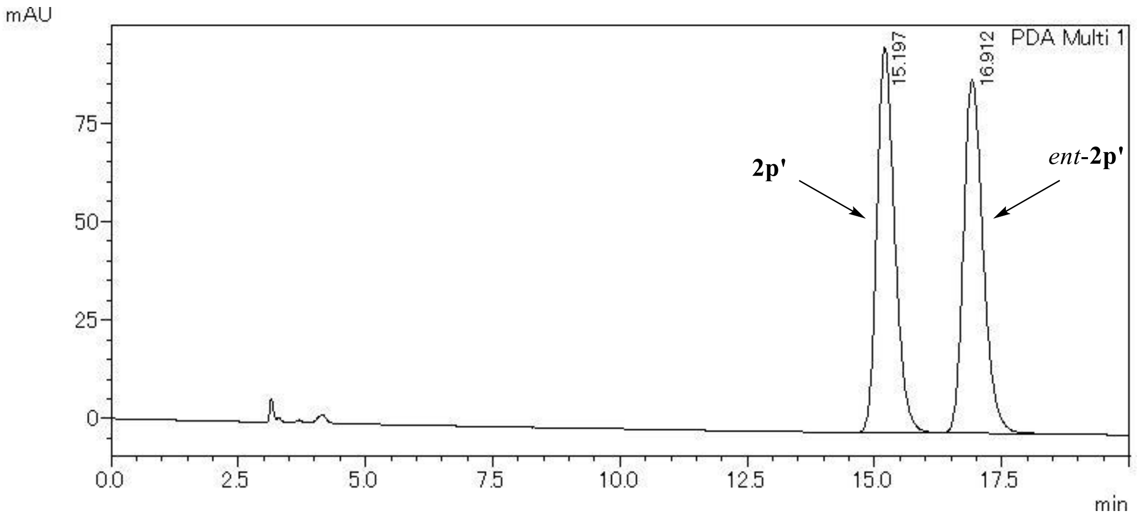

peak	retention time (min)	area %
1	15.197	49.844
2	16.912	50.156

Supplementary Figure 120. HPLC Chromatogram Profiles of 1-(*tert*-Butyldimethylsilyl)-1-cyanopent-4-yn-1-yl tosylcarbamate (**2p'**)

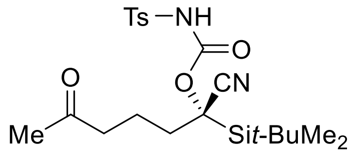

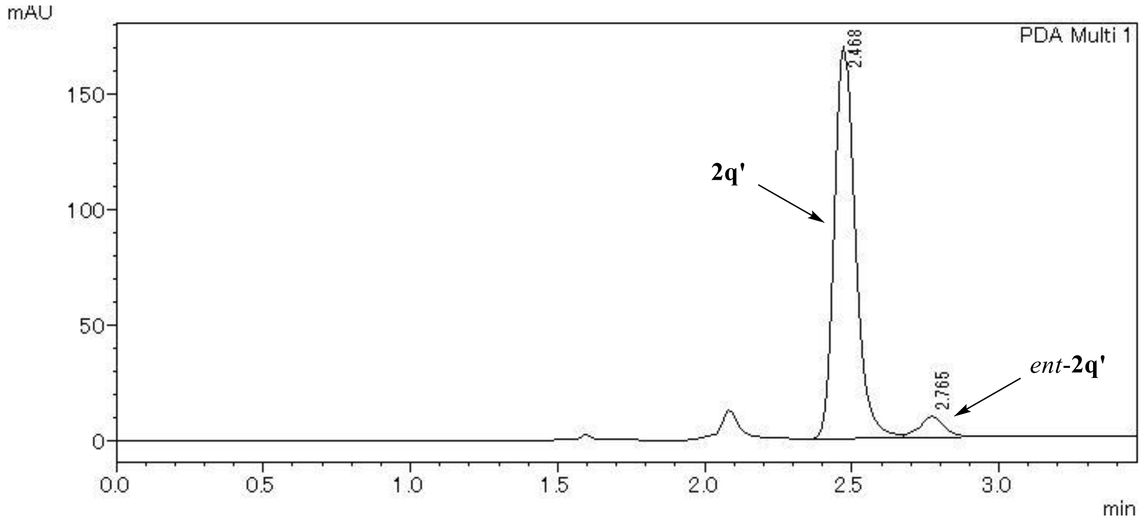

peak	retention time (min)	area %
1	2.468	93.646
2	2.765	6.354

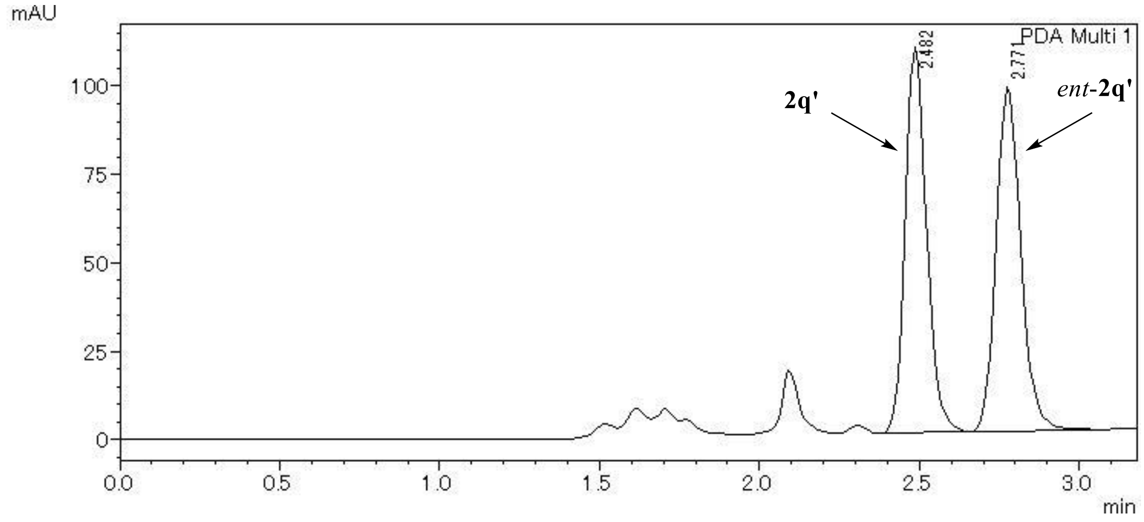

peak	retention time (min)	area %
1	2.482	49.417
2	2.771	50.583

Supplementary Figure 121. HPLC Chromatogram Profiles of 1-(*tert*-Butyldimethylsilyl)-1-cyano-5-oxohexyl tosylcarbamate (**2q'**)

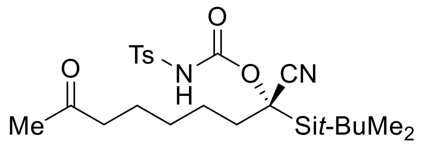

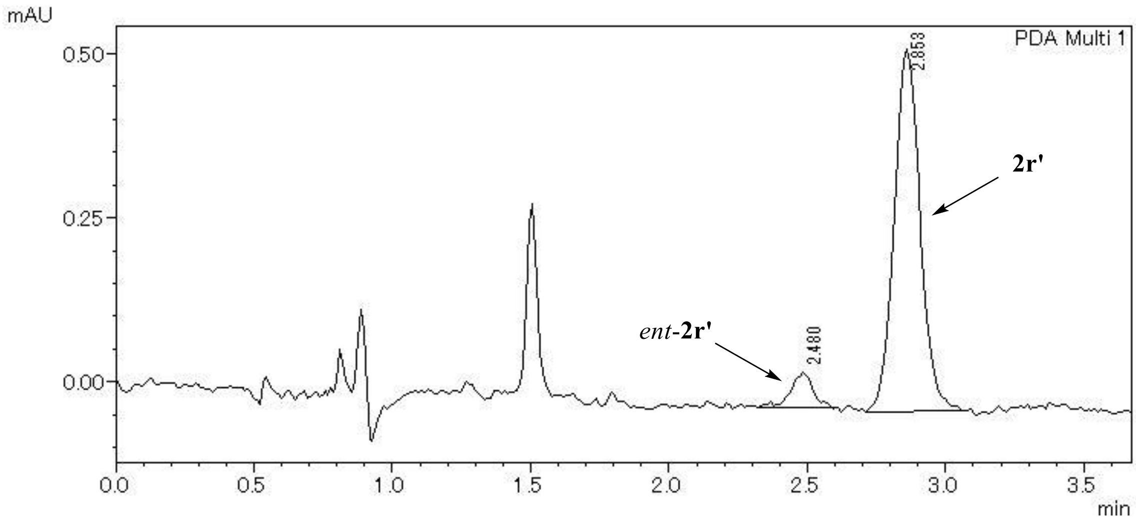

peak	retention time (min)	area %
1	2.480	7.542
2	2.853	92.458

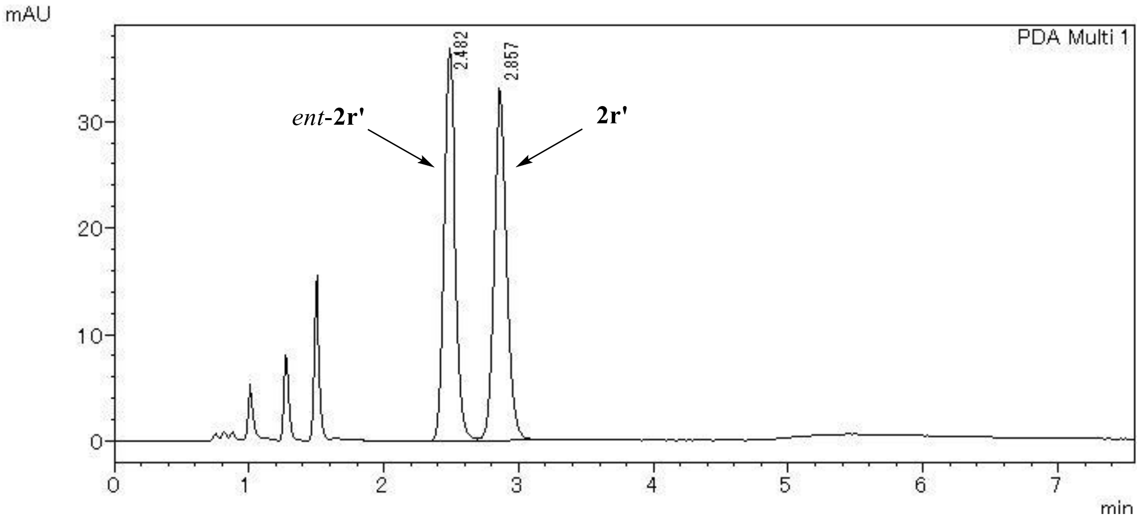

peak	retention time (min)	area %
1	2.482	49.555
2	2.857	50.445

Supplementary Figure 122. HPLC Chromatogram Profiles of 1-(*tert*-Butyldimethylsilyl)-1-cyano-7-oxooctyl tosylcarbamate (**2r'**)

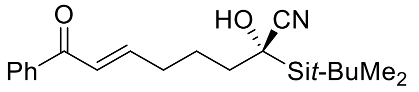

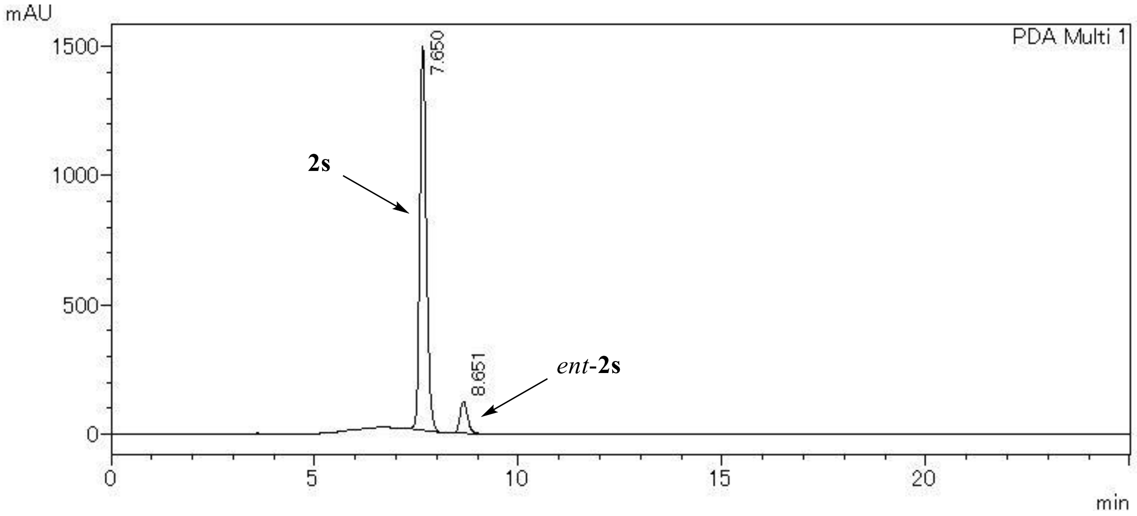

peak	retention time (min)	area %
1	7.650	91.313
2	8.651	8.687

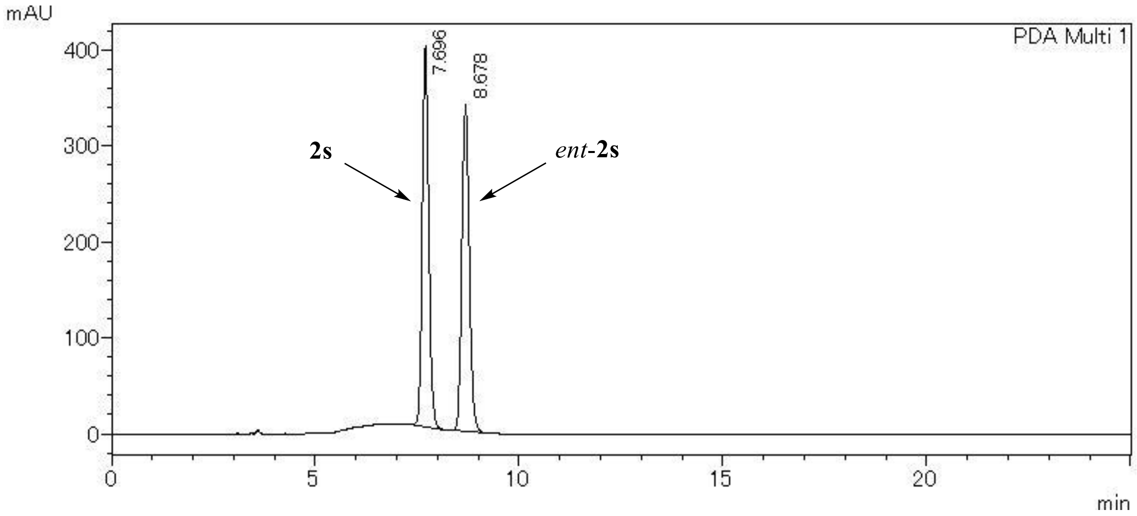

peak	retention time (min)	area %
1	7.696	50.359
2	8.678	49.641

Supplementary Figure 123. HPLC Chromatogram Profiles of (*E*)-2-(*tert*-Butyldimethylsilyl)-2-hydroxy-8-oxo-8-phenyloct-6-enenitrile (**2s**)

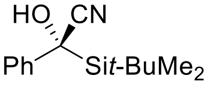

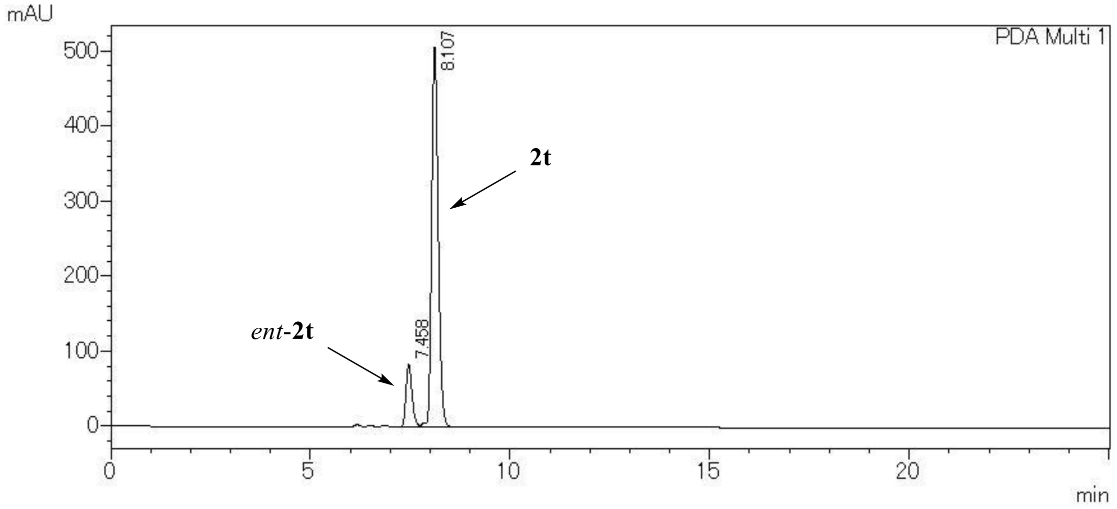

peak	retention time (min)	area %
1	7.458	14.153
2	8.107	85.847

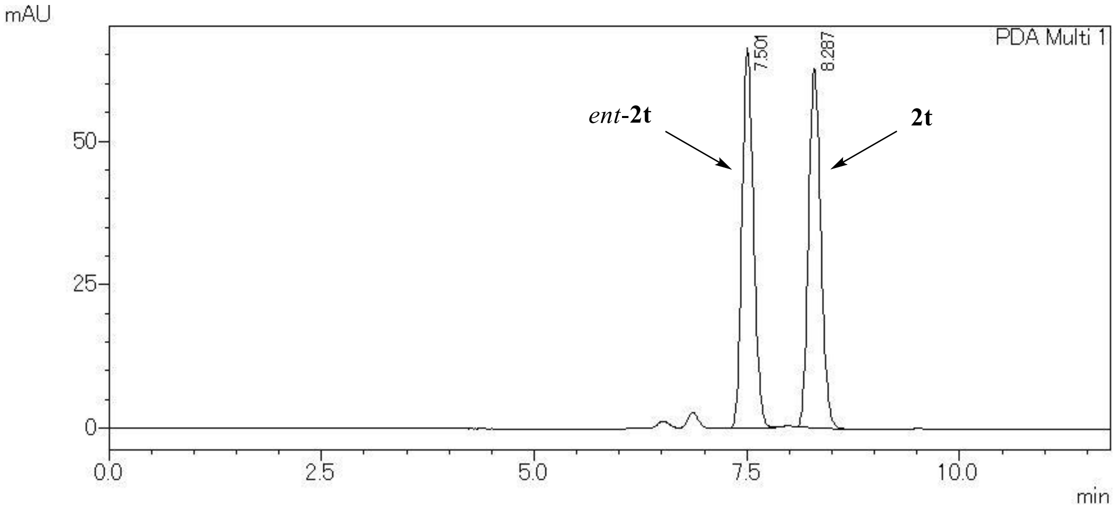

peak	retention time (min)	area %
1	7.501	51.458
2	8.287	48.542

Supplementary Figure 124. HPLC Chromatogram Profiles of 2-(*tert*-Butyldimethylsilyl)-2-hydroxy-2-phenylacetonitrile (**2t**)

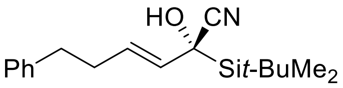

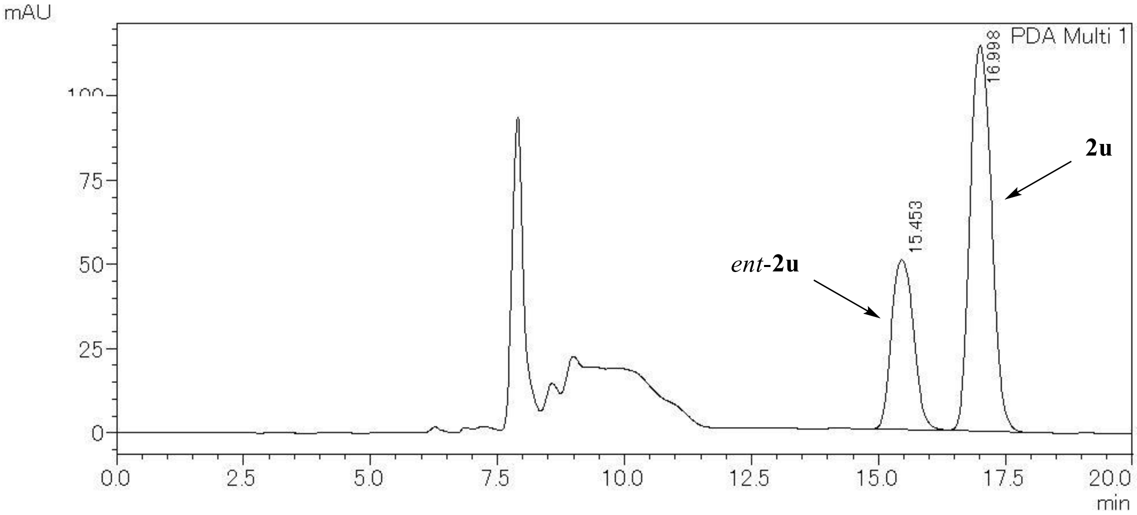

peak	retention time (min)	area %
1	15.453	31.234
2	16.998	68.766

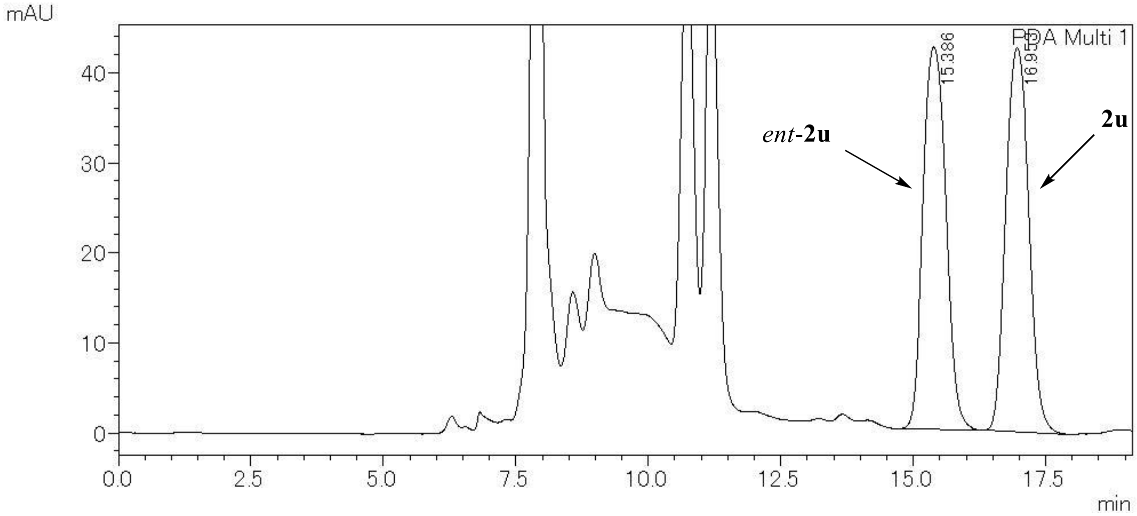

peak	retention time (min)	area %
1	15.386	49.603
2	16.953	50.397

Supplementary Figure 125. HPLC Chromatogram Profiles of (*E*)-2-(*tert*-Butyldimethylsilyl)-2-hydroxy-6-phenylhex-3-enenitrile (**2u**)

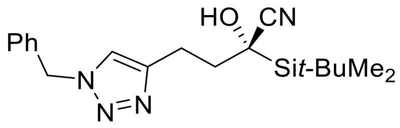

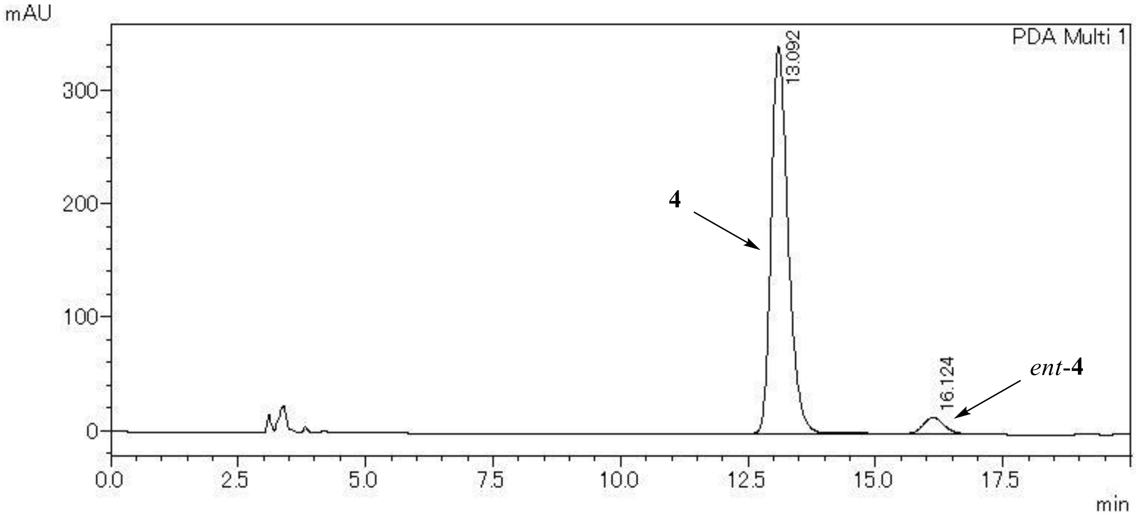

peak	retention time (min)	area %
1	13.092	95.108
2	16.124	4.892

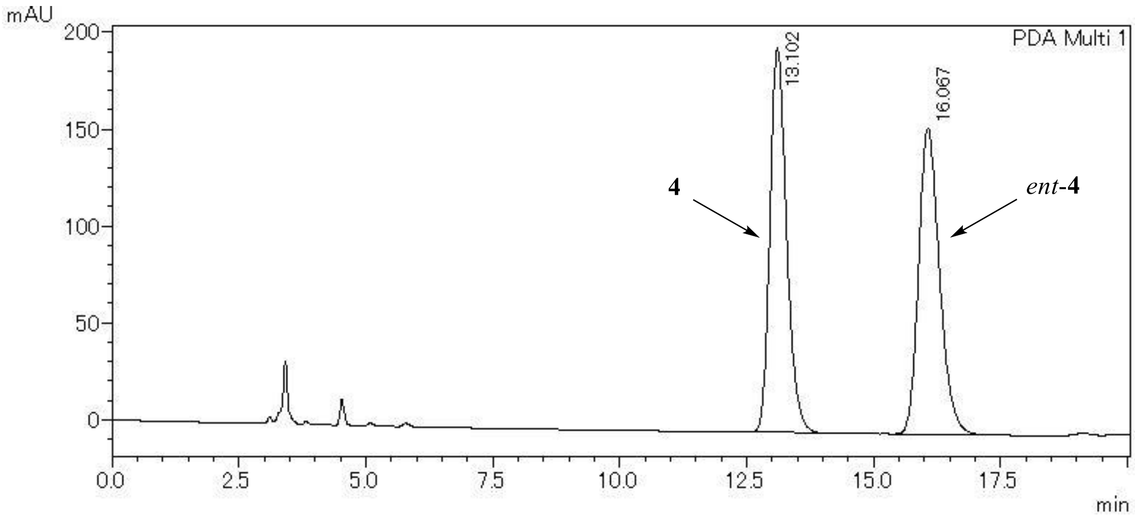

peak	retention time (min)	area %
1	13.102	49.982
2	16.067	50.018

Supplementary Figure 126. HPLC Chromatogram Profiles of 4-(1-Benzyl-1*H*-1,2,3-triazol-4-yl)-2-(*tert*-butyldimethylsilyl)-2-hydroxybutanenitrile (**4**)

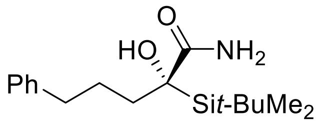

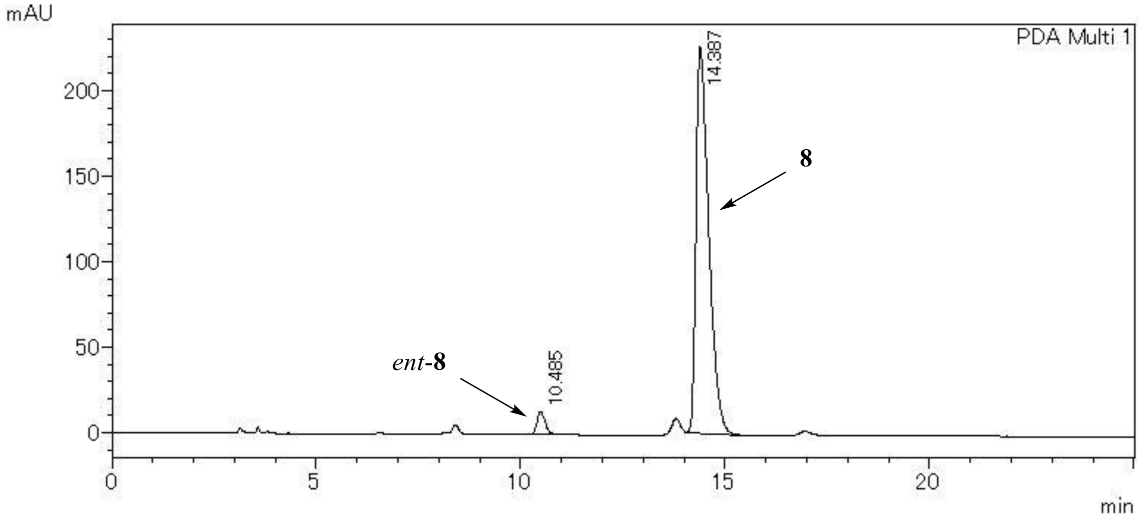

peak	retention time (min)	area %
1	10.485	3.641
2	14.387	96.359

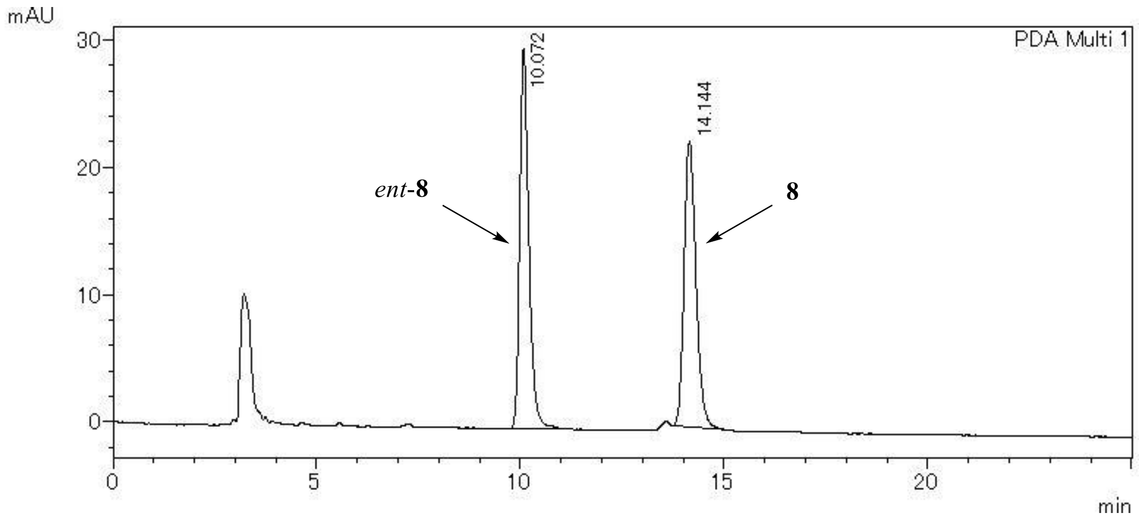

peak	retention time (min)	area %
1	10.072	50.662
2	14.144	49.338

Supplementary Figure 127. HPLC Chromatogram Profiles of 2-(*tert*-Butyldimethylsilyl)-2-hydroxy-5-phenylpentanamide (**8**)

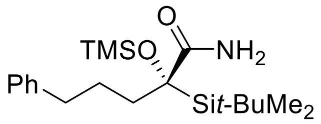

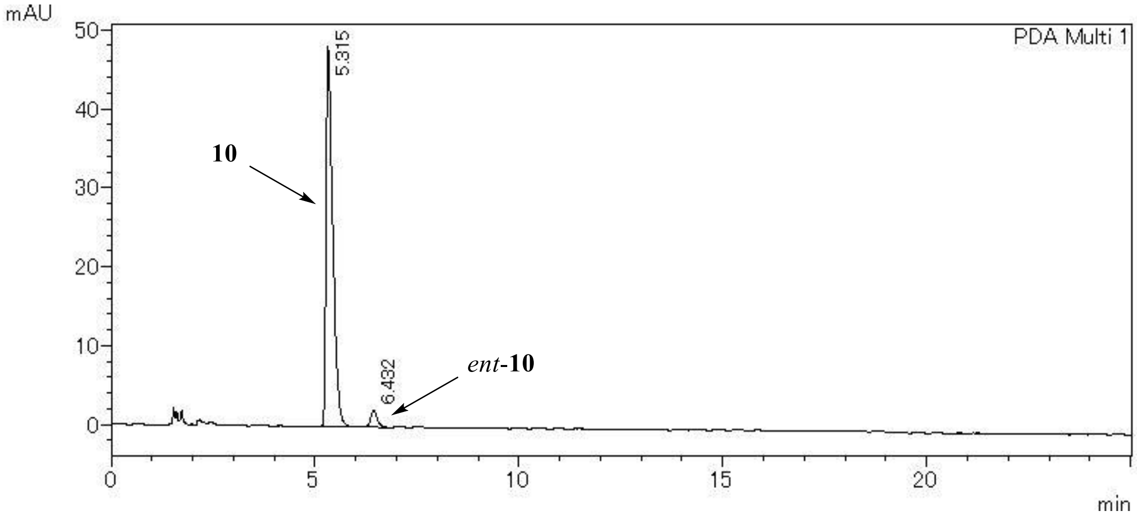

peak	retention time (min)	area %
1	5.315	95.824
2	6.432	4.176

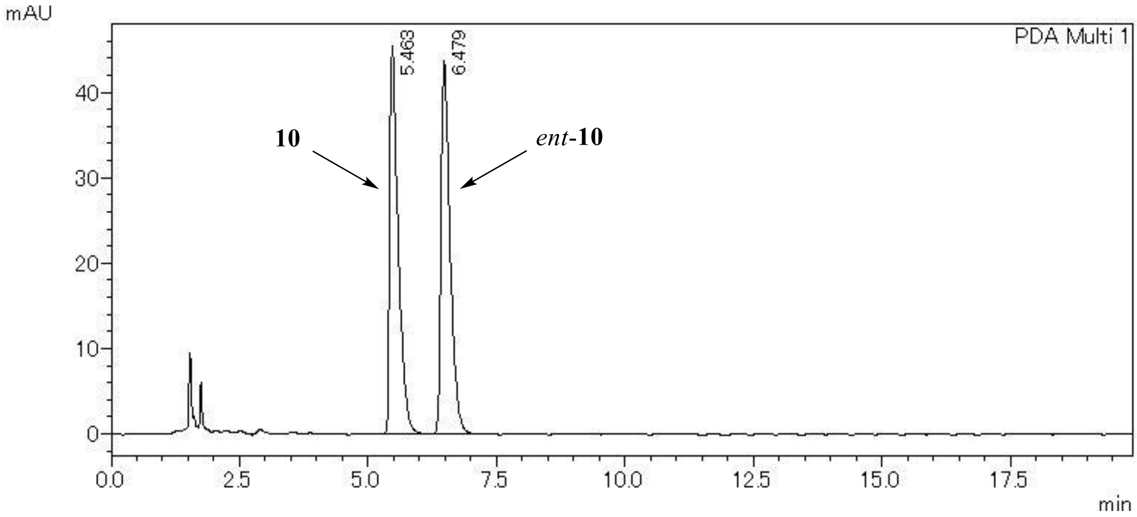

peak	retention time (min)	area %
1	5.463	49.945
2	6.479	50.055

Supplementary Figure 128. HPLC Chromatogram Profiles of 2-(*tert*-Butyldimethylsilyl)-5-phenyl-2-((trimethylsilyl)oxy)pentanamide (**10**)

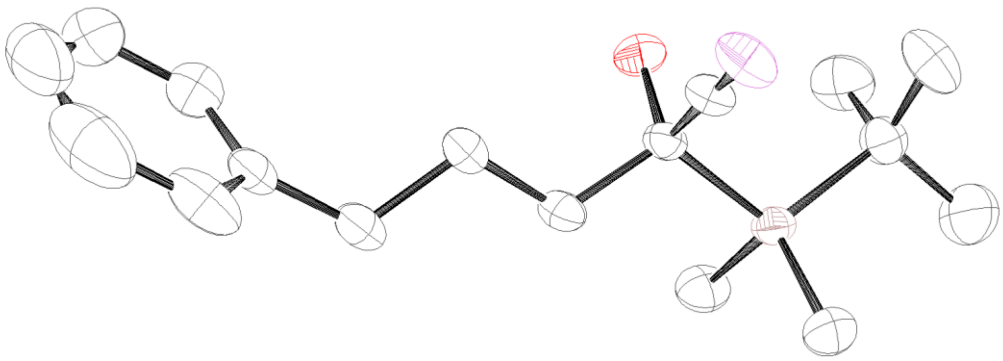

Supplementary Figure 129. ORTEP Drawing of **2a**

Supplementary References

1. Matsumoto, A.; Asano, K.; Matsubara, S. *Chem.—Asian J.* **2019**, *14*, 116.
2. Tsukano, C.; Nakajima, M.; Hande, S. M.; Takemoto, Y. *Org. Biomol. Chem.* **2019**, *17*, 1731.
3. Kurosawa, W.; Kan, T.; Fukuyama, T. *Org. Synth.* **2002**, *79*, 186.
4. Schultz, E. E.; Sarpong, R. *J. Am. Chem. Soc.* **2013**, *135*, 4696.
5. Narayanappa, A.; Huren, D.; McNulty, J. *Synlett* **2017**, *28*, 2961.
6. Zhang, W.-C.; Li, C.-J. *J. Org. Chem.* **2000**, *65*, 5831.
7. Sakaguchi, K.; Yamamoto, M.; Kawamoto, T.; Yamada, T.; Shinada, T.; Shimamoto, K.; Ohfunea, Y. *Tetrahedron Lett.* **2004**, *45*, 5869.
8. Becker, J.; Bergander, K.; Fröhlich, R.; Hoppe, D. *Angew. Chem., Int. Ed.* **2008**, *47*, 1654.
9. Anderson, B. K.; Livinghouse, T. *J. Org. Chem.* **2015**, *80*, 9847.
10. Karimi, B.; Golshani, B. *J. Org. Chem.* **2000**, *65*, 7228.
11. Shao, C.; Wang, X.; Zhang, Q.; Luo, S.; Zhao, J.; Hu, Y. *J. Org. Chem.* **2011**, *76*, 6832.
12. Kanda, T.; Naraoka, A.; Naka, H. *J. Am. Chem. Soc.* **2019**, *141*, 825.
13. Kim, E. S.; Lee, H. S.; Kim, S. H.; Kim, J. N. *Tetrahedron Lett.* **2010**, *51*, 1589.
