# Supplementary material for: Non-enzymatic catalytic asymmetric cyanation of acylsilanes
Source: Commun Chem. 2022 Mar 31;5:45. doi: 10.1038/s42004-022-00662-y (PMC9814240; doi:10.1038/s42004-022-00662-y)
Supplement: Supplementary file 3 — Description of Additional Supplementary Files [file 42004_2022_662_MOESM3_ESM.pdf]

Description of Additional Supplementary Files

File Name: Supplementary Data 1

Description: cif of 2a
